# Supplementary material for: On the limits of fitting complex models of population history to f-statistics
Source: eLife. 2023 Jun 29;12:e85492. doi: 10.7554/eLife.85492 (PMC10310323; doi:10.7554/eLife.85492)

**Figure 3–source data 1.** The published (Bergström et al. 2020) and alternative admixture graphs for dogs found with *findGraphs*. Model parameters (graph edges) that were inferred to be unidentifiable are plotted in red.

**a**, published model

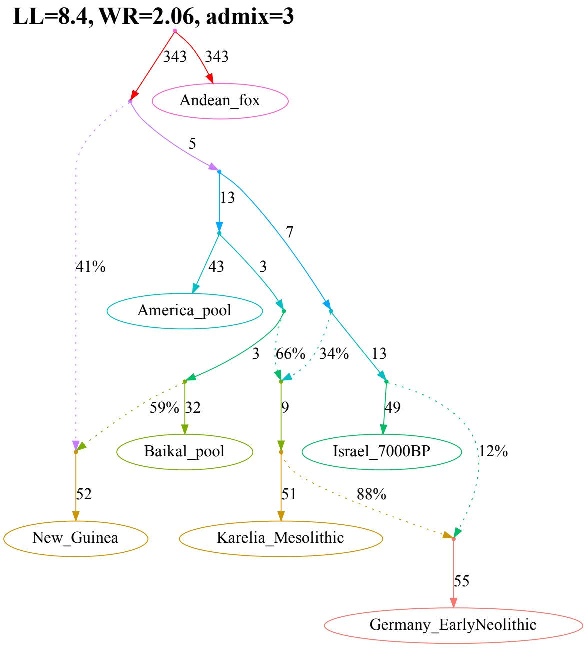

**b**, alternative models fitting nominally better than the published one, sorted by the fit score

the best model corresponding perfectly to the human history

**LL=2.4,WR=0.95, admix=3**

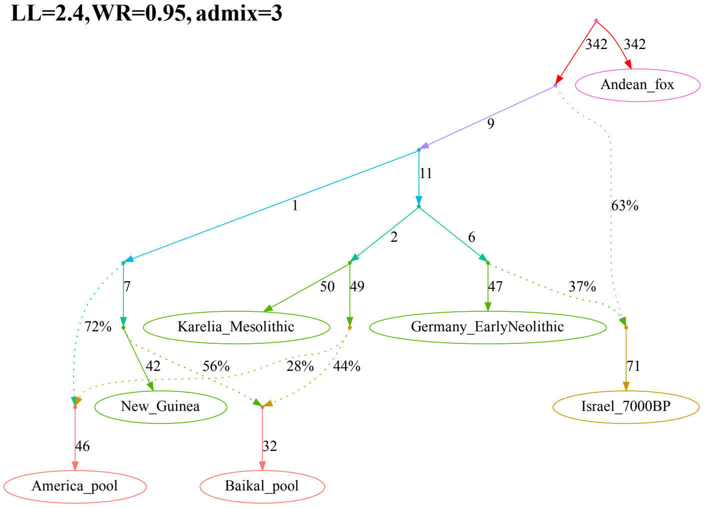


**LL=4.0, WR=1.48, admix=3**

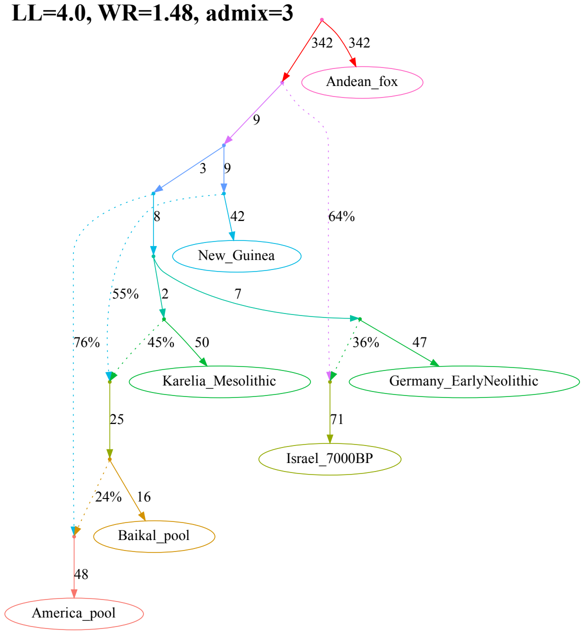


**LL=5.7, WR=2.04, admix=3**

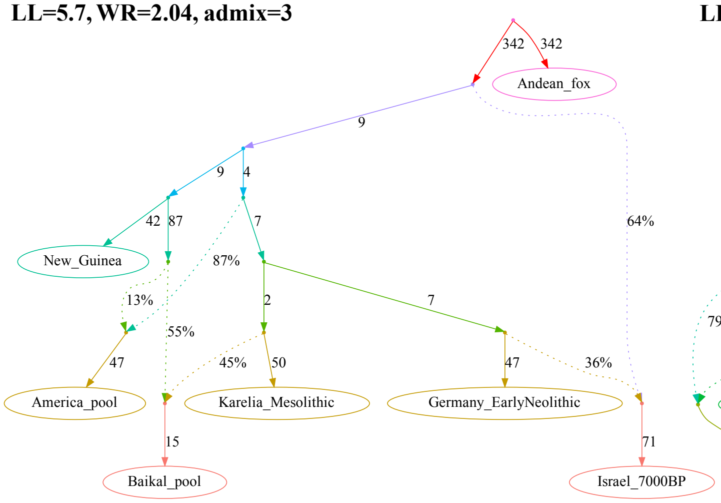


**LL=6.7, WR=2.04, admix=3**

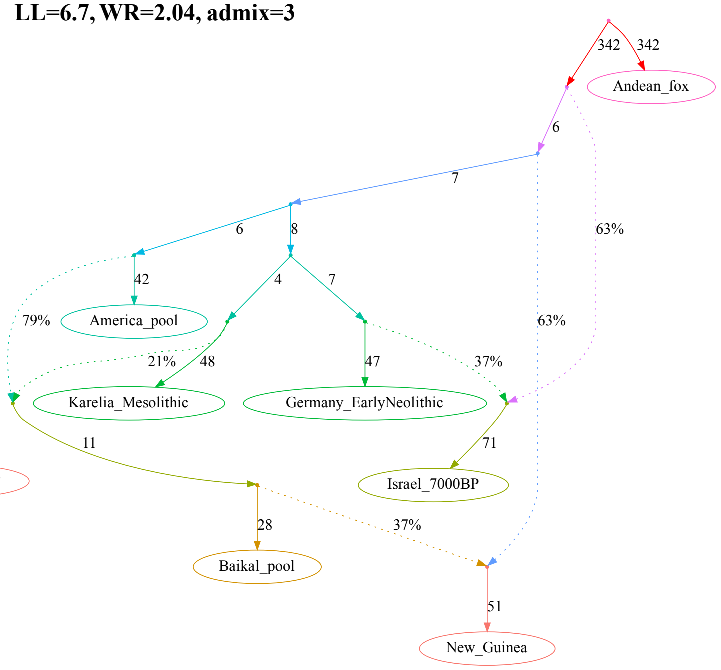

**c**, alternative models fitting nominally or significantly (the graph framed in blue) better than the published one, sorted by the fit score

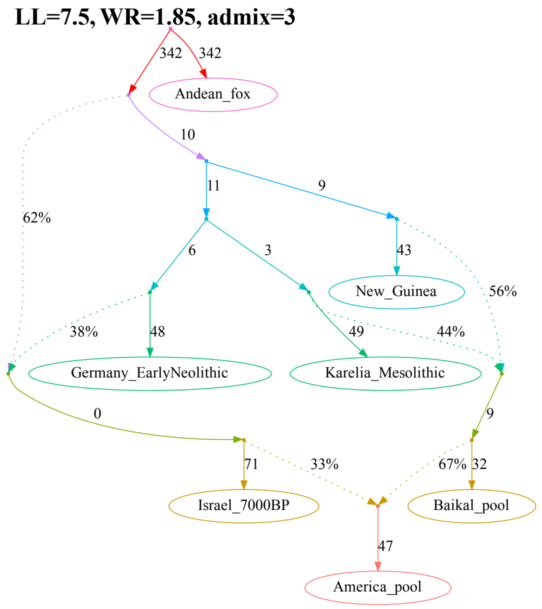


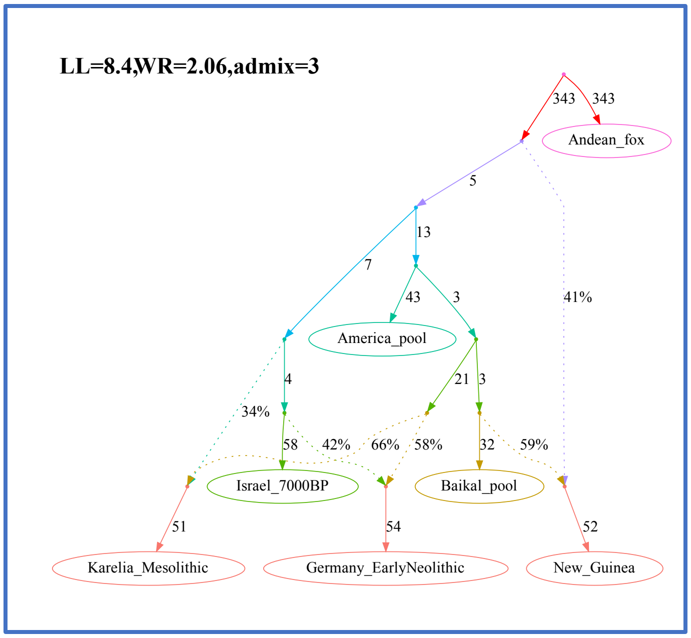

Supplement: Figure 3—source data 1. — Model parameters (graph edges) that were inferred to be unidentifiable are plotted in red. (a) The published model, (b) alternative models fitting nominally better than the published one, sorted by the fit score, and (c) alternative models fitting nominally or significantly (the graph framed in blue) better than the published one, sorted by the fit score. [file elife-85492-fig3-data1.pdf]
