# Supplementary material for: On the limits of fitting complex models of population history to f-statistics
Source: eLife. 2023 Jun 29;12:e85492. doi: 10.7554/eLife.85492 (PMC10310323; doi:10.7554/eLife.85492)

Figure 3–source data 2. Alternative admixture graphs for humans found with *findGraphs* for the dataset from Bergström *et al*. (2020). Model parameters (graph edges) that were inferred to be unidentifiable are plotted in red.

a, best-fitting models for humans sorted by the fit score

LL=1.0, WR=0.61, admix=3

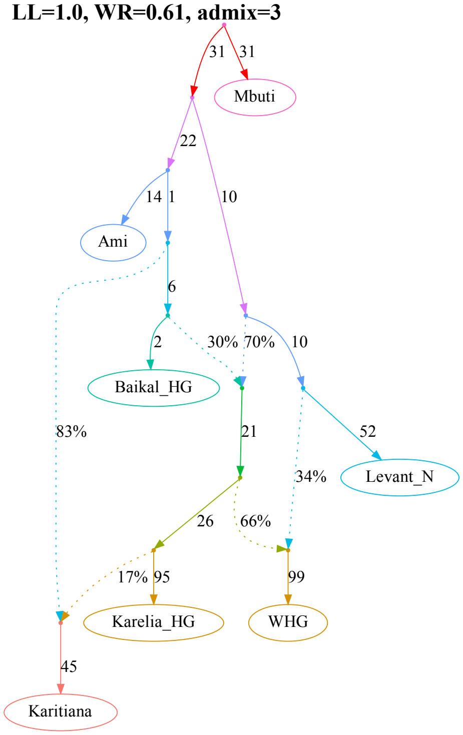

LL=1.8, WR=0.88, admix=3

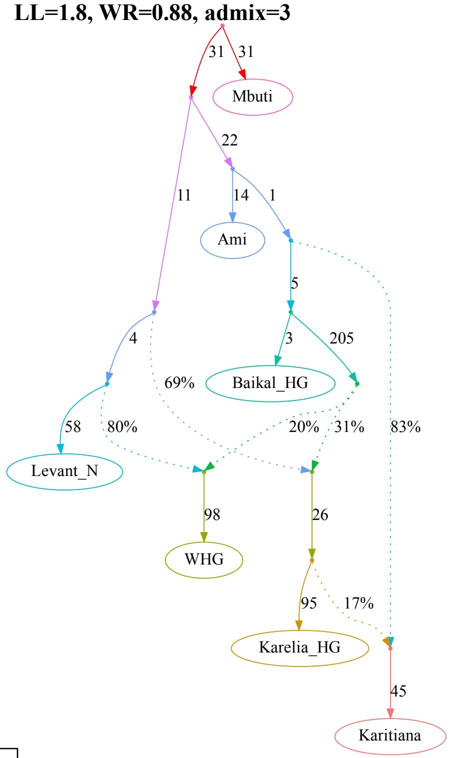

models below are very similar to the best-fitting model for dogs

LL=6.1, WR=2.05, admix=3

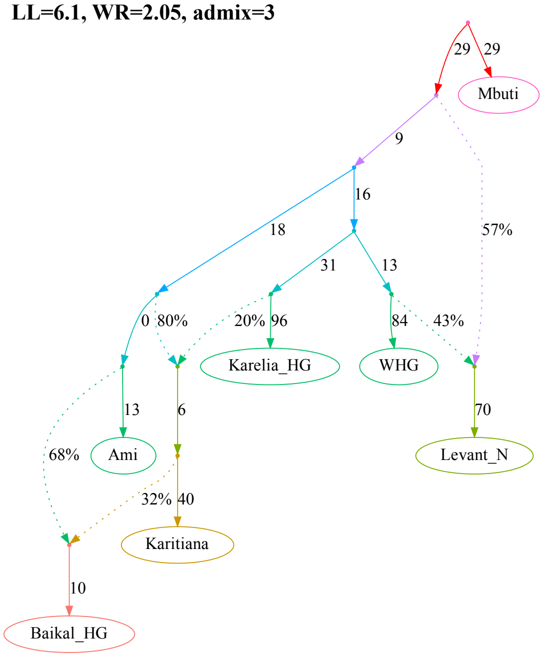

LL=6.1, WR=2.05, admix=3

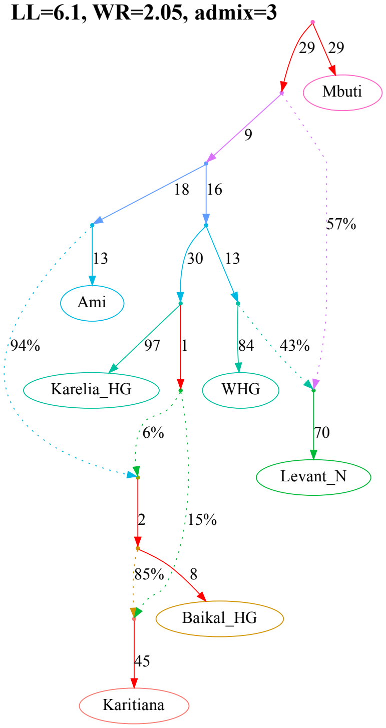

b, best-fitting models for humans sorted by the fit score

LL=6.1, WR=2.05, admix=3

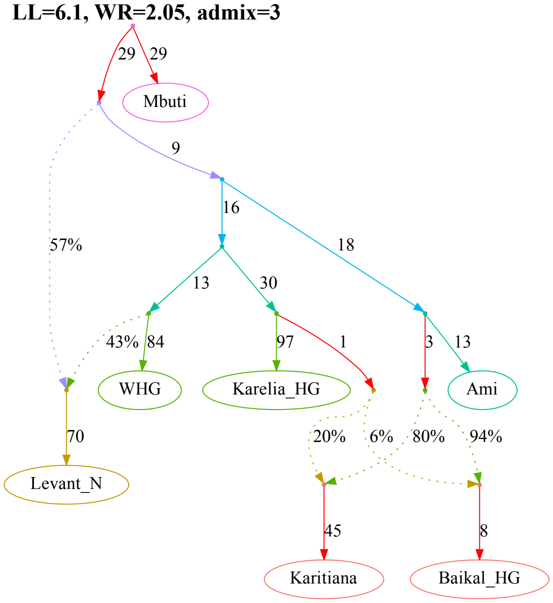

LL=6.1, WR=2.05, admix=3

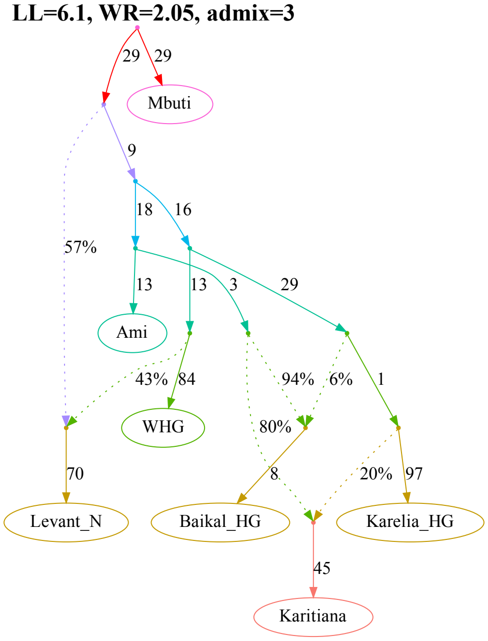

LL=6.2, WR=2.05, admix=3

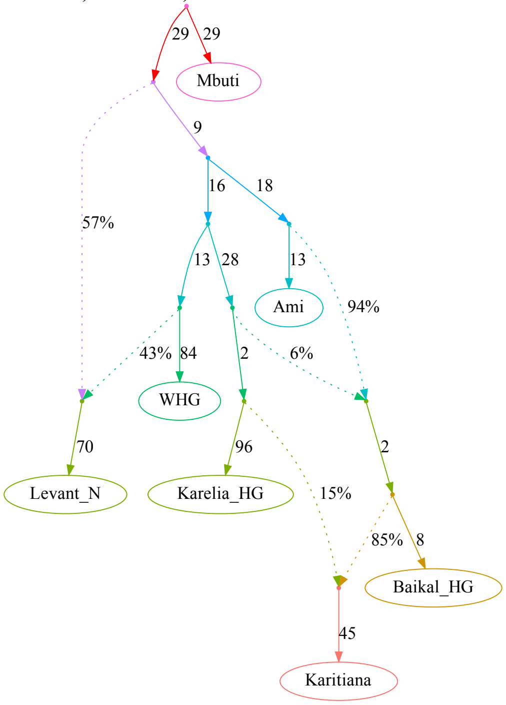

LL=6.2, WR=2.05, admix=3

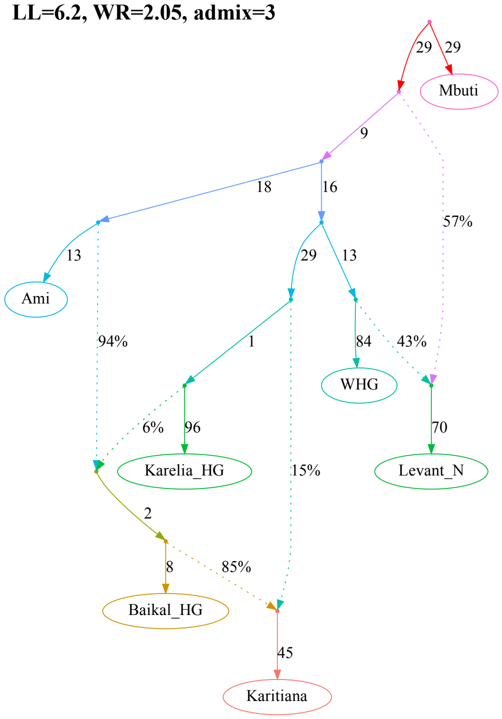

Supplement: Figure 3—source data 2. — Model parameters (graph edges) that were inferred to be unidentifiable are plotted in red. (a, b) Best-fitting models for humans sorted by the fit score. [file elife-85492-fig3-data2.pdf]
