# Supplementary material for: On the limits of fitting complex models of population history to f-statistics
Source: eLife. 2023 Jun 29;12:e85492. doi: 10.7554/eLife.85492 (PMC10310323; doi:10.7554/eLife.85492)

Figure 3–source data 3. Published admixture graph from Lazaridis *et al*. (2014) and alternative graphs found with *findGraphs* (7 populations, 4 admixture events). Model parameters (graph edges) that were inferred to be unidentifiable are plotted in red.

a, the re-fitted published graph

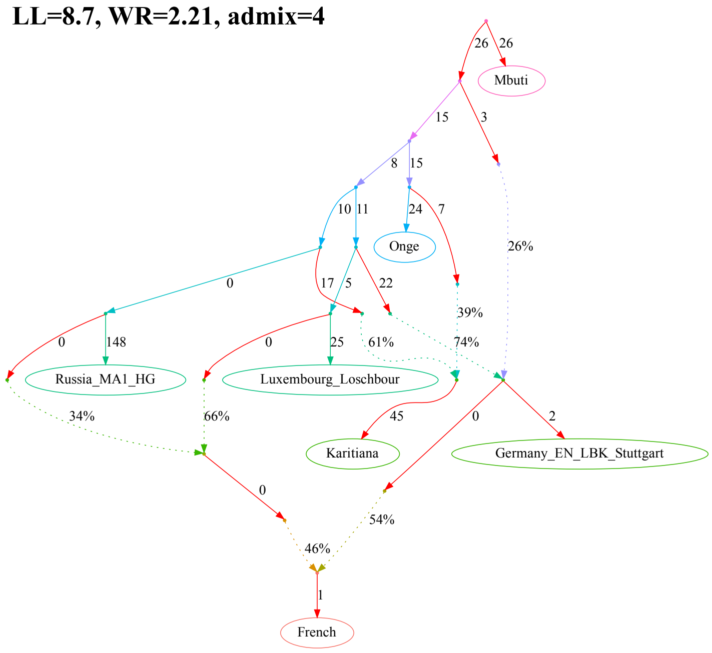

b, ten examples of graphs inferred by *findGraphs* (arranged according to LL score) and fitting significantly (the graph framed in blue) or nominally better than the published model.

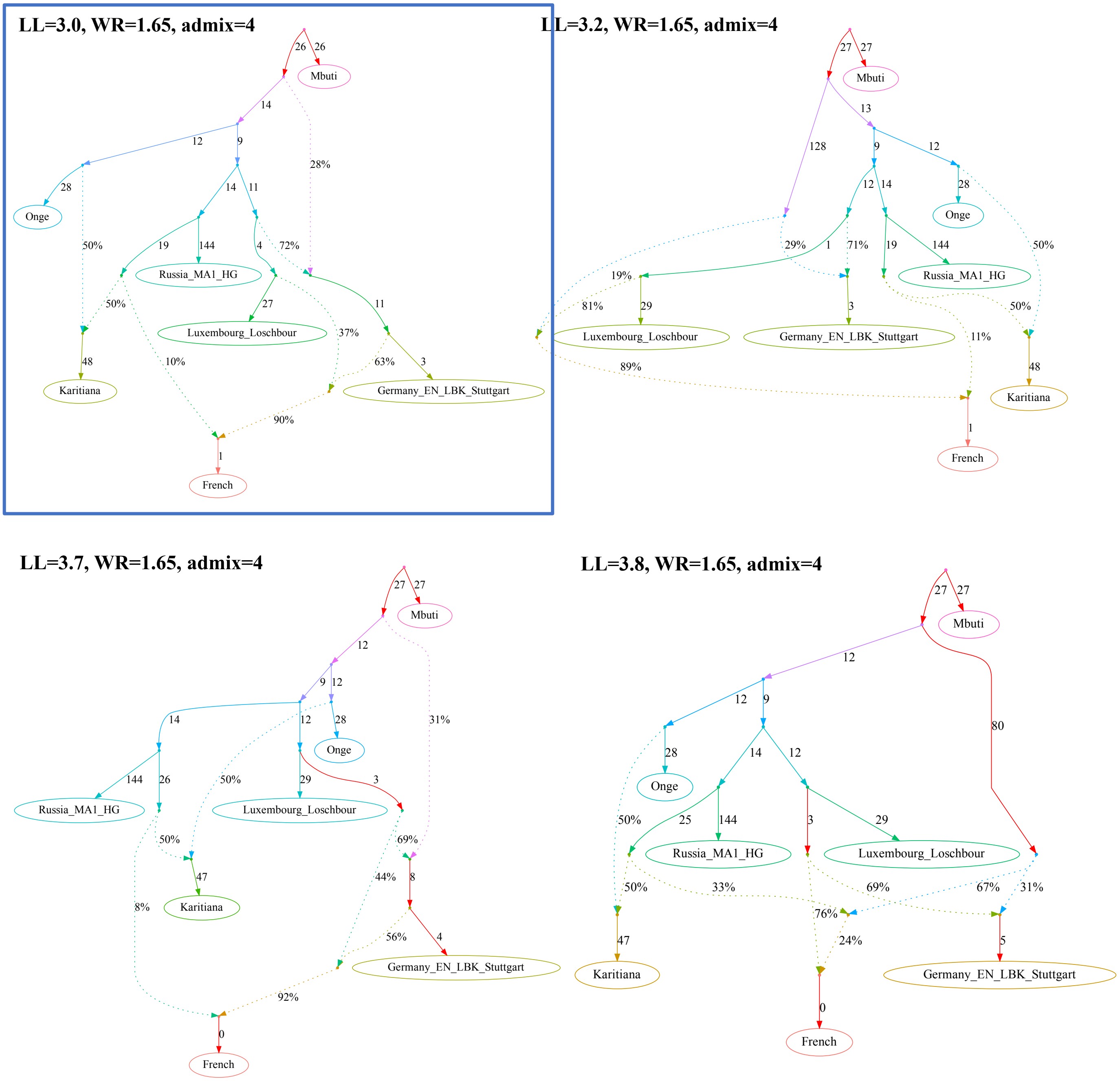

LL=4.5, WR=1.83, admix=4

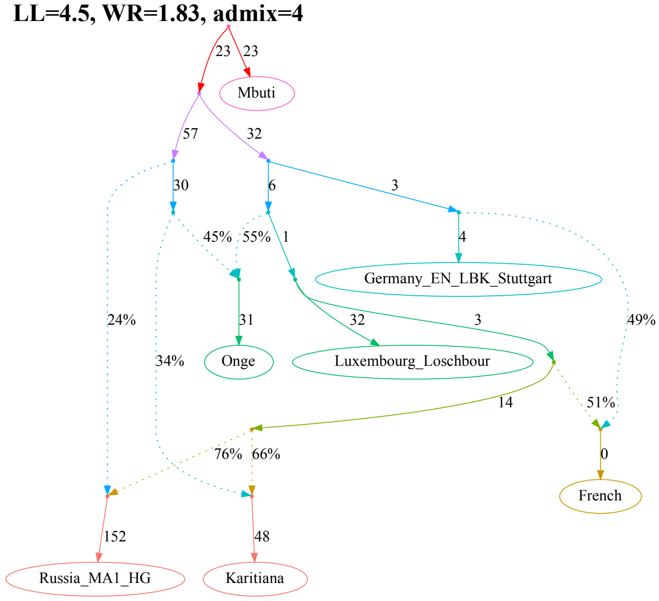

LL=4.5, WR=1.83, admix=4

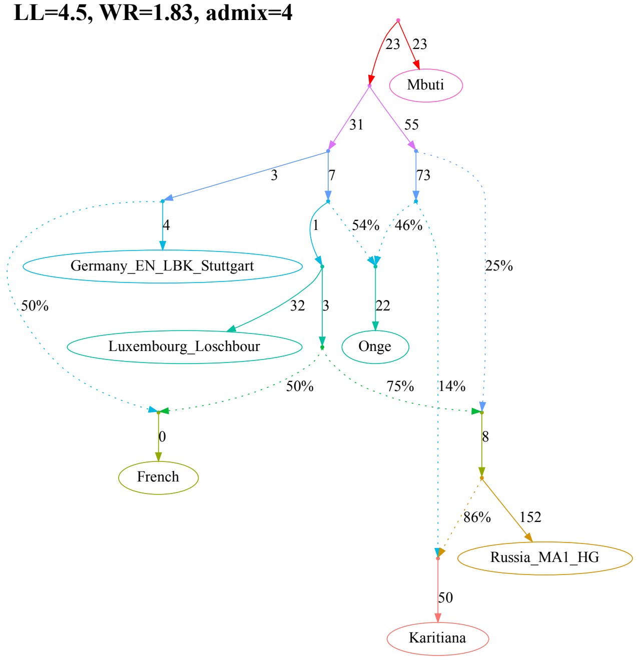

LL=5.3, WR=1.58, admix=4

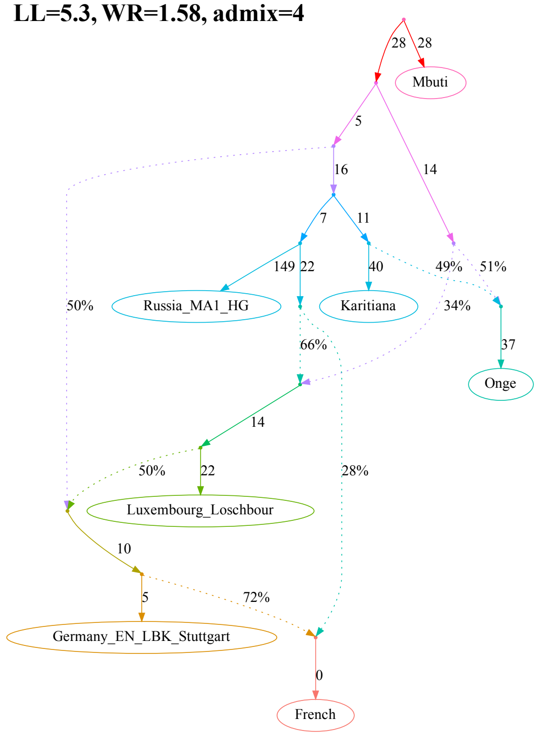

LL=5.7, WR=2.00, admix=4

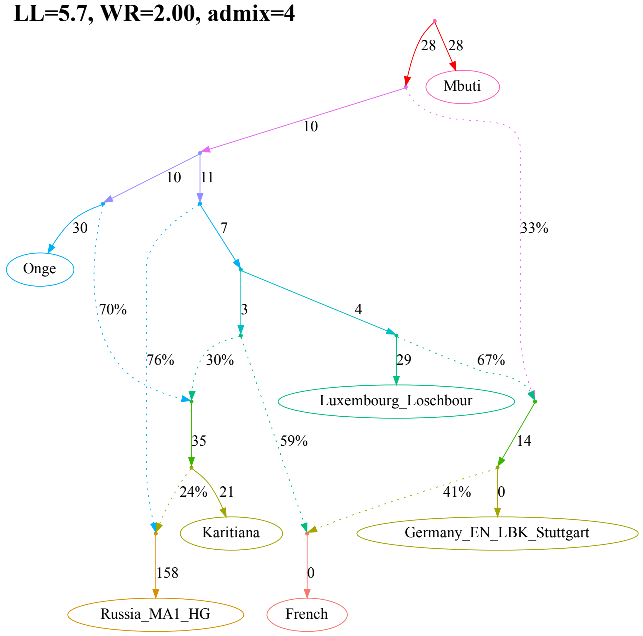

LL=6.2, WR=2.03, admix=4

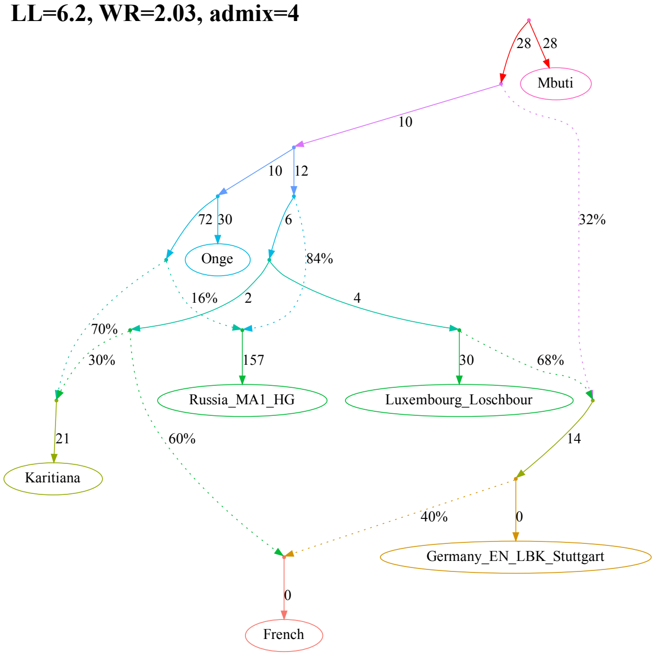

LL=7.2, WR=1.91, admix=4

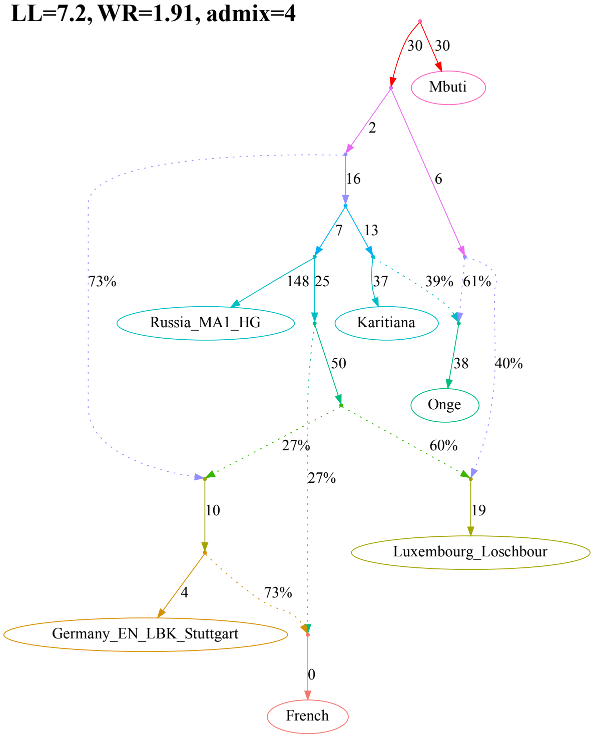

Supplement: Figure 3—source data 3. — Model parameters (graph edges) that were inferred to be unidentifiable are plotted in red. (a) The re-fitted published graph and (b) 10 examples of graphs inferred by findGraphs (arranged according to LL score) and fitting significantly (the graph framed in blue) or nominally better than the published model. [file elife-85492-fig3-data3.pdf]
