# Supplementary material for: On the limits of fitting complex models of population history to f-statistics
Source: eLife. 2023 Jun 29;12:e85492. doi: 10.7554/eLife.85492 (PMC10310323; doi:10.7554/eLife.85492)

Figure 3–source data 4. Published admixture graph from Shinde *et al*. (2019) and alternative graphs found with *findGraphs* (8 populations, 3 admixture events) relying on the original set of SNPs, original group composition, and original (incorrect) algorithm for calculating *f*-statistics. Model parameters (graph edges) that were inferred to be unidentifiable are plotted in red.

a, published model; the original set of SNPs and individuals, and the original algorithm for calculating f_3-statistics was used (470,389 variable site with no missing data at the group level available)

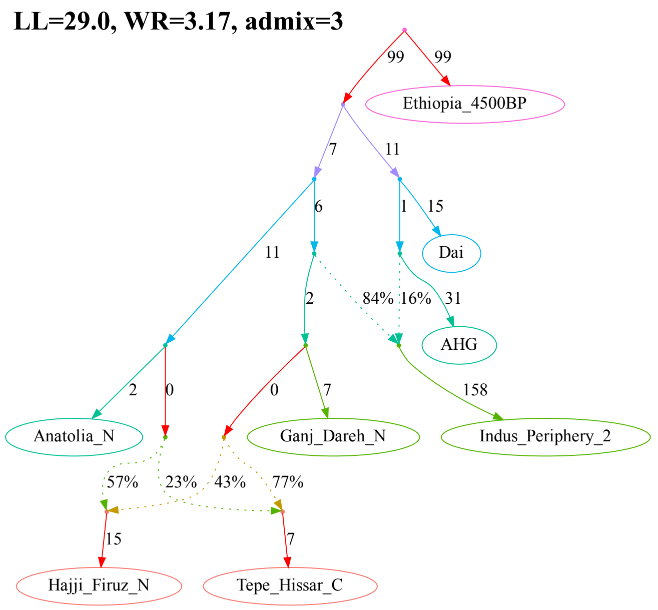

The claim by Shinde et al. 2019 relying on the admixture graph:

Primary ancestry in the Indus Periphery group forms the deepest branch in the Iranian Neolithic clade composed of the Indus Periphery, Ganj Dareh Neolithic, Hajji Firuz Neolithic, and Tepe Hissar Chalcolithic groups.

b, selected alternative models fitting significantly better (graphs framed in blue), nominally better (graphs without frames), or not significantly worse (graphs framed in red) than the published one

Indus Periphery is not the deepest branch in the Iranian Neolithic clade

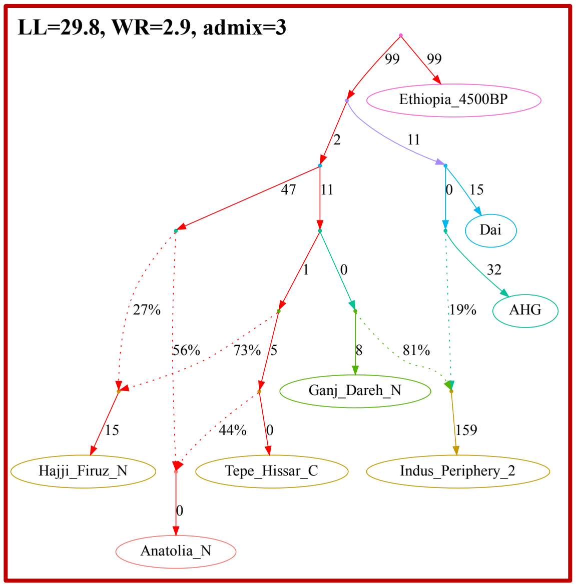

Indus Periphery is not the deepest branch in the Iranian Neolithic clade

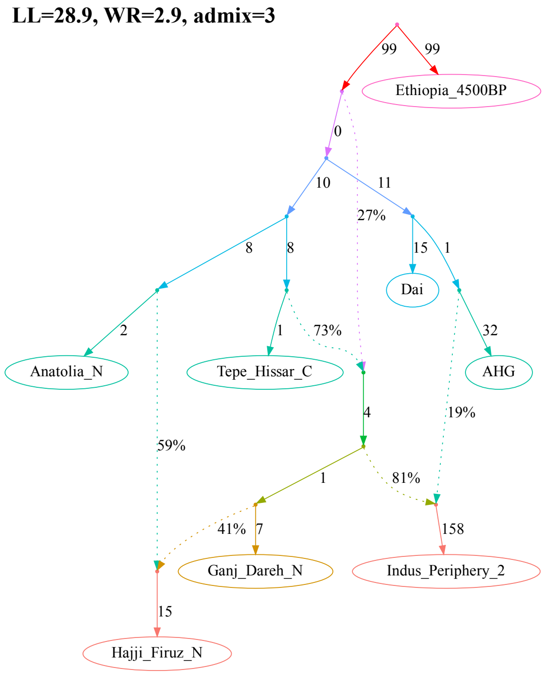

Indus Periphery is not the deepest branch in the Iranian Neolithic clade
Indus Periphery is not the deepest branch in the Iranian Neolithic clade

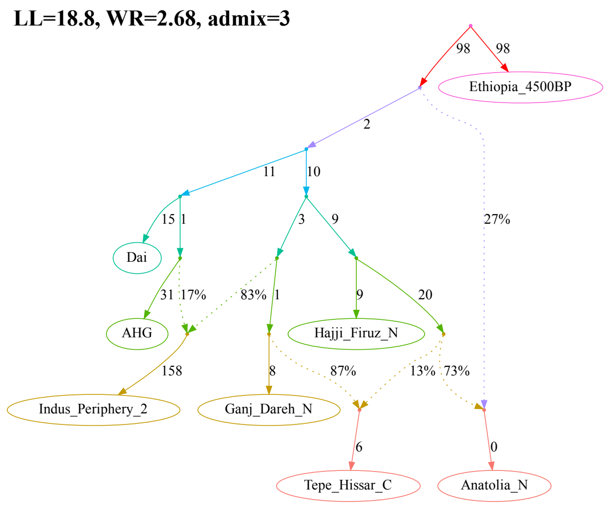

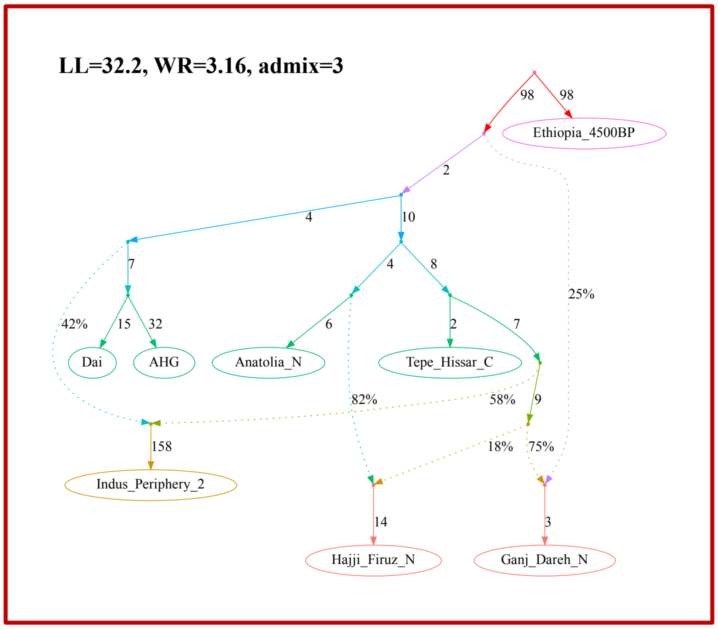

c, selected alternative models fitting significantly better (graphs framed in blue), nominally better (graphs without frames), or not significantly worse (graphs framed in red) than the published one

Indus Periphery is not the deepest branch in the Iranian Neolithic clade

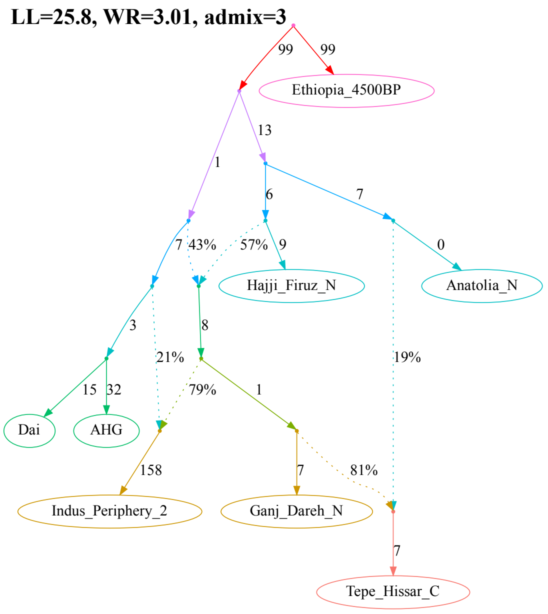

Indus Periphery is not the deepest branch in the Iranian Neolithic clade

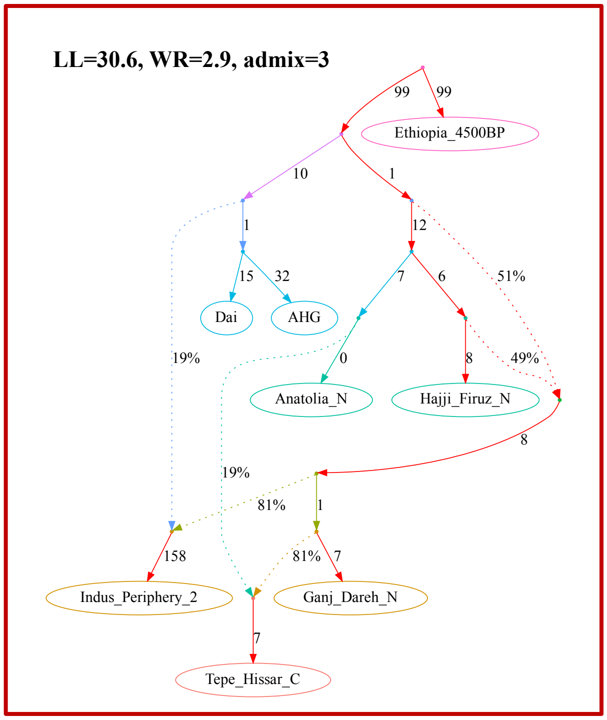

Indus Periphery is not the deepest branch in the Iranian Neolithic clade

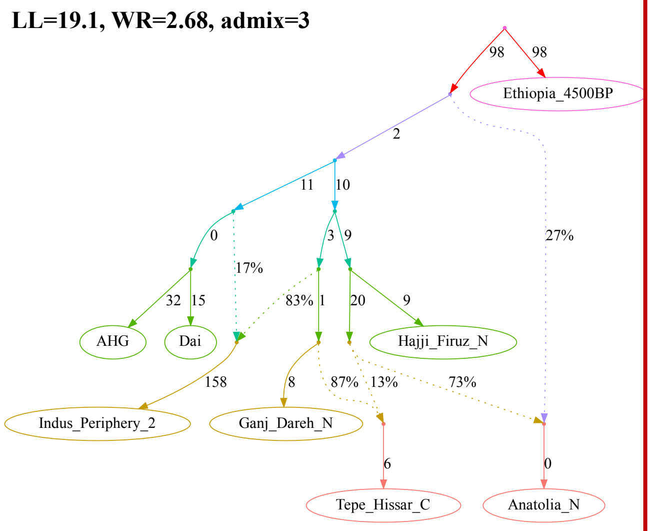

Indus Periphery is not the deepest branch in the Iranian Neolithic clade

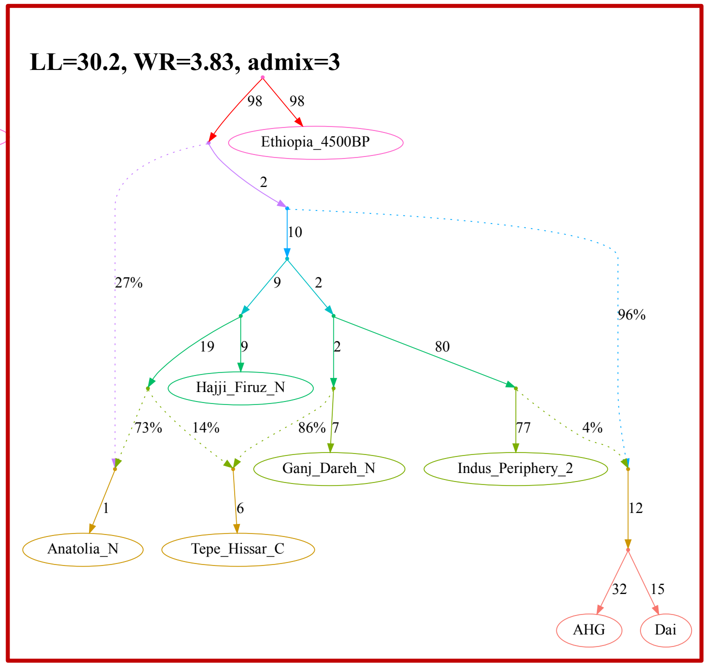

d, selected alternative models fitting significantly better (graphs framed in blue), nominally better (graphs without frames), or not significantly worse (graphs framed in red) than the published one

Primary ancestry in Indus Periphery is a basal Asian branch

LL=25.7, WR=3.01, admix=3

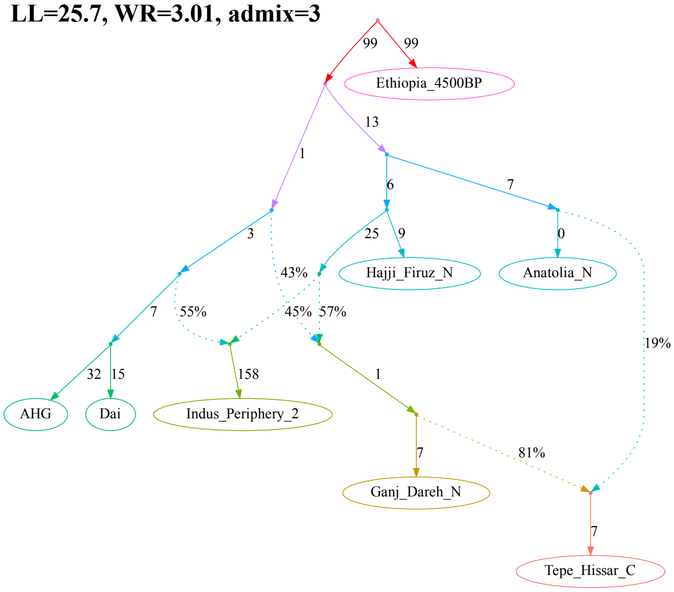

Primary ancestry in Indus Periphery is a basal Asian branch

LL=15.2, WR=2.68, admix=3

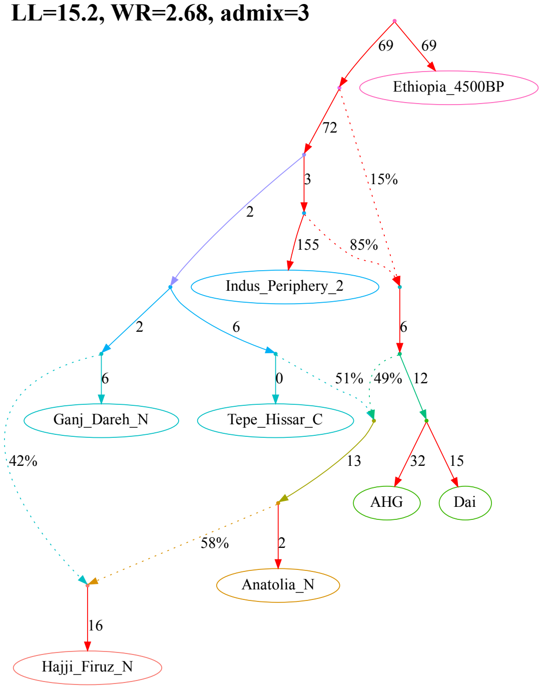

Primary ancestry in Indus Periphery is a basal Asian branch

LL=22.3, WR=2.68, admix=3

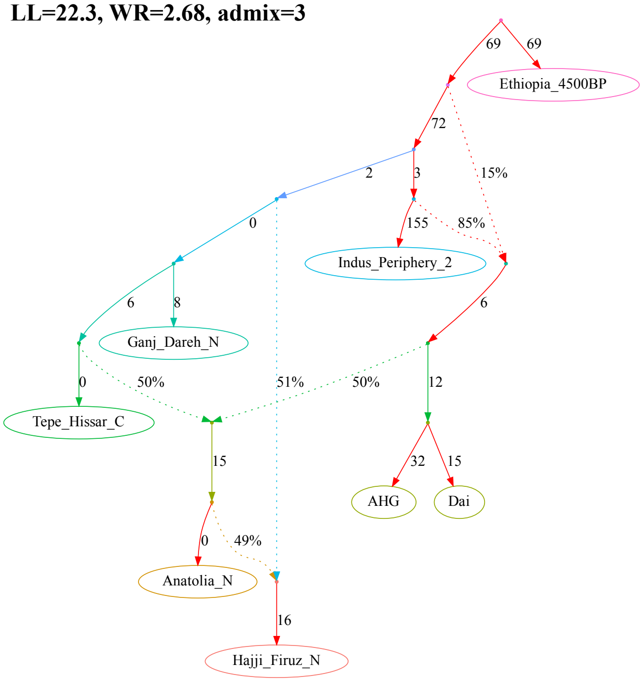

Primary ancestry in Indus Periphery is a basal West Eurasian branch

LL=12.4, WR=2.68, admix=3

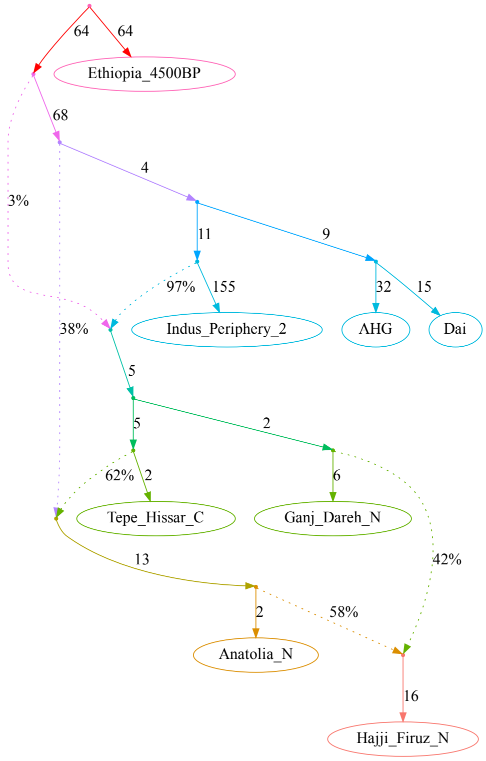

e, selected alternative models fitting significantly better (graphs framed in blue), nominally better (graphs without frames), or not significantly worse (graphs framed in red) than the published one

Indus Periphery is the deepest branch in the Iranian Neolithic clade

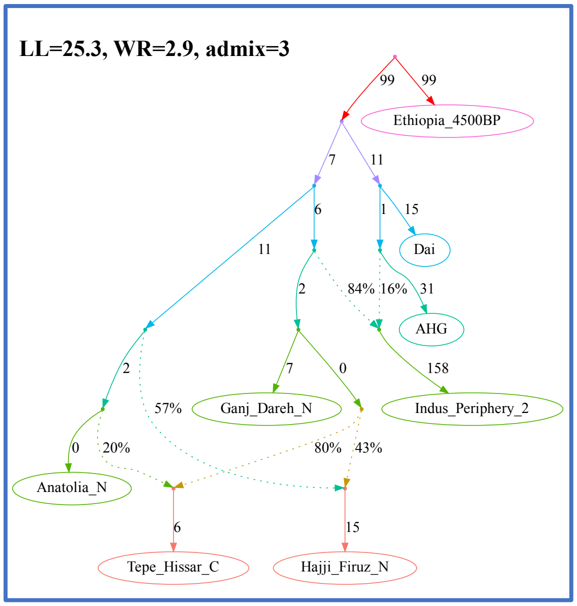

Indus Periphery is the deepest branch in the Iranian Neolithic clade

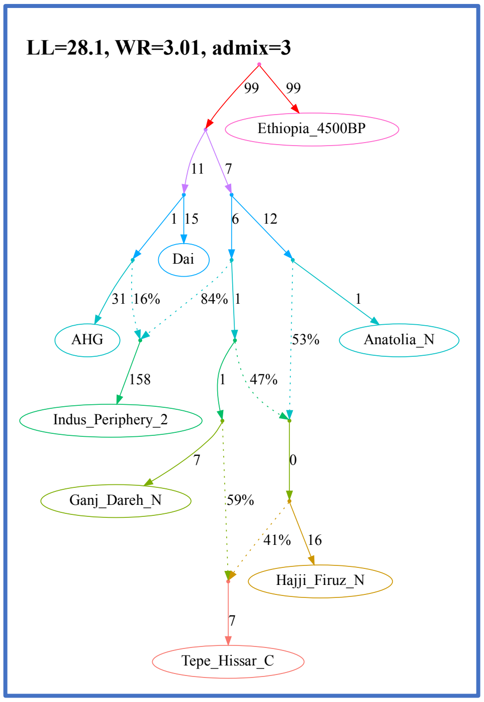

Indus Periphery is the deepest branch in the Iranian Neolithic clade

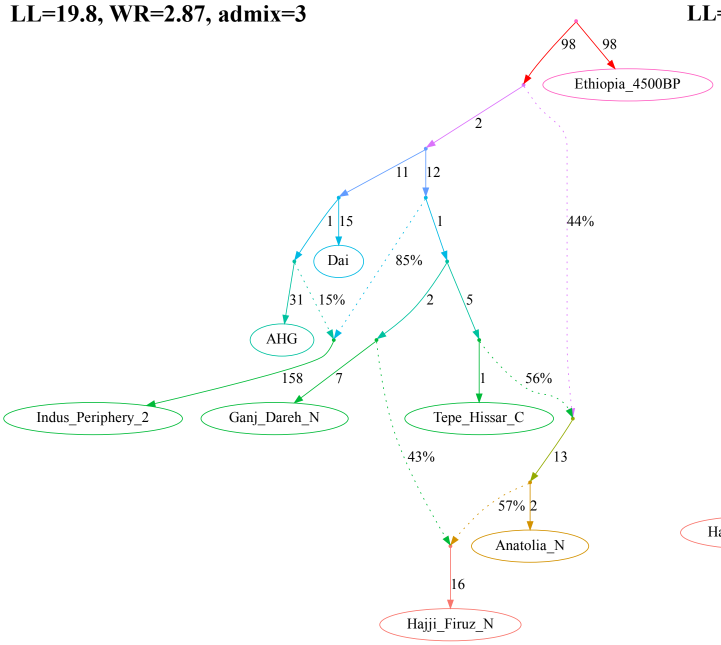

Indus Periphery is the deepest branch in the Iranian Neolithic clade

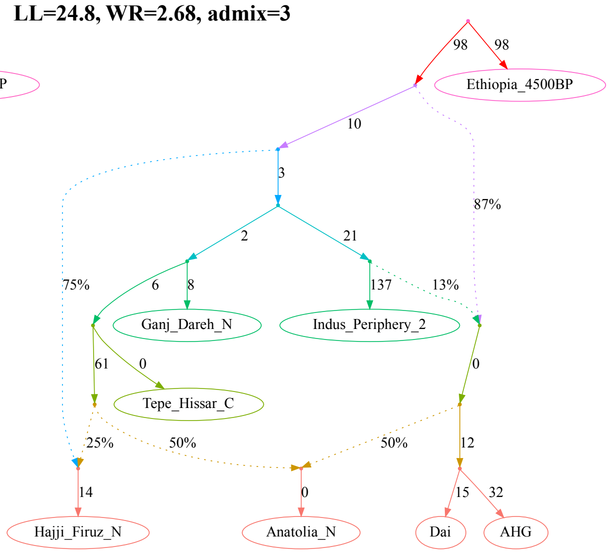

f, selected alternative models fitting significantly better (graphs framed in blue), nominally better (graphs without frames), or not significantly worse (graphs framed in red) than the published one

Indus Periphery is the deepest branch in the Iranian Neolithic clade

LL=15.1, WR=2.68, admix=3

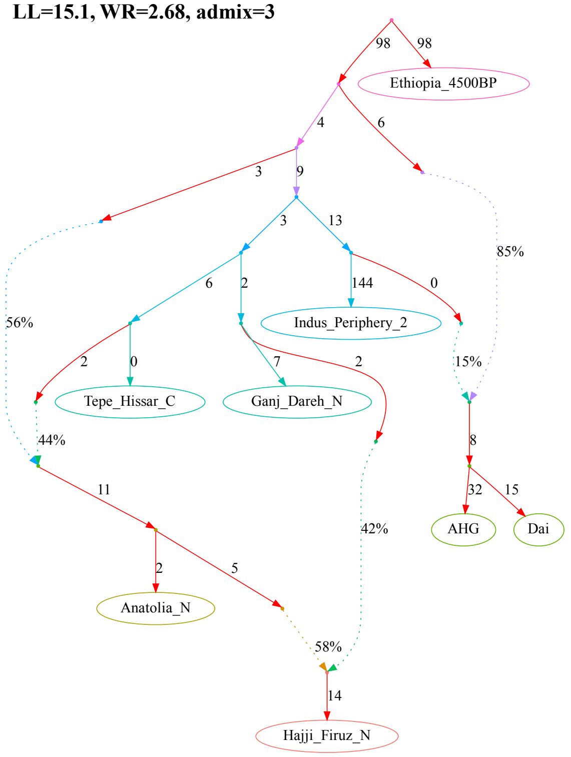

Indus Periphery is the deepest branch in the Iranian Neolithic clade

LL=23.6, WR=3.12, admix=3

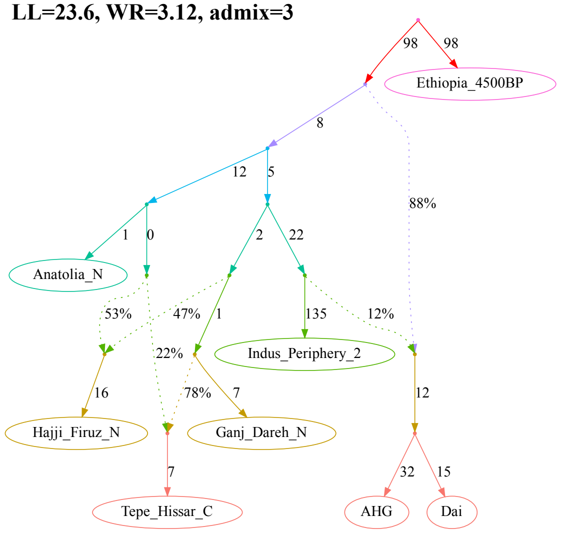

Supplement: Figure 3—source data 4. — Model parameters (graph edges) that were inferred to be unidentifiable are plotted in red. (a) The published model; the original set of SNPs and individuals, and the original algorithm for calculating f3-statistics was used (470,389 variable sites with no missing data at the group level available). The following claim in Shinde et al., 2019 relies on the admixture graph: Primary ancestry in the Indus Periphery group forms the deepest branch in the Iranian Neolithic clade composed of the Indus Periphery, Ganj Dareh Neolithic, Hajji Firuz Neolithic, and Tepe Hissar Chalcolithic groups. (b-f) Selected alternative models fitting significantly better (graphs framed in blue), nominally better (graphs without frames), or not significantly worse (graphs framed in red) than the published one. [file elife-85492-fig3-data4.pdf]
