# Supplementary material for: On the limits of fitting complex models of population history to f-statistics
Source: eLife. 2023 Jun 29;12:e85492. doi: 10.7554/eLife.85492 (PMC10310323; doi:10.7554/eLife.85492)

Figure 3–source data 6. Alternative graphs allowing for an additional admixture event found with *findGraphs* for the dataset from Shinde *et al*. (2019): 8 populations, 4 admixture events, the modified group composition and the updated algorithm for calculating *f*-statistics. The graphs were also re-fitted on the original set of SNPs/individuals and using the original algorithm for calculating *f*-statistics. Model parameters (graph edges) that were inferred to be unidentifiable are plotted in red.

a, highest-ranking model with 4 admixture events; confirms all important features of the published model with 3 admixture events

Relatives (2nd and 3rd degree) and potentially contaminated individuals removed, the Indus Periphery group expanded to 4 ind., the corrected algorithm for calculating f_3-statistics (249,009 variable site with no missing data at the group level)	The original set of SNPs and individuals, and the original algorithm for calculating f_3-statistics (470,389 variable site with no missing data at the group level)
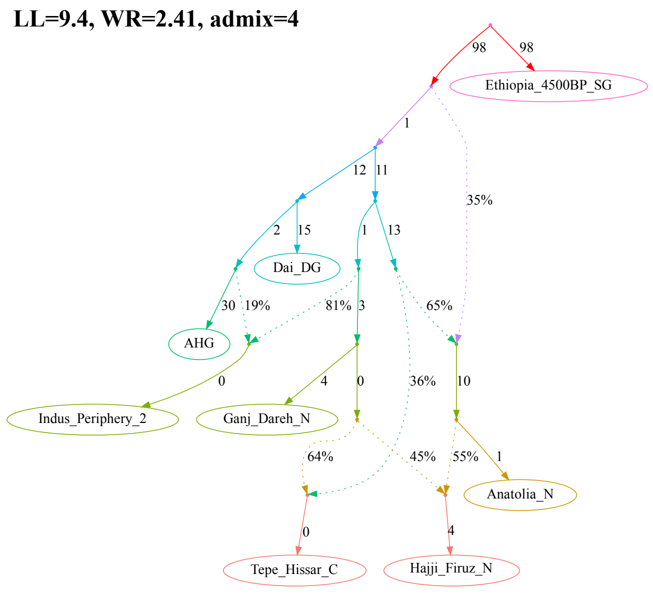	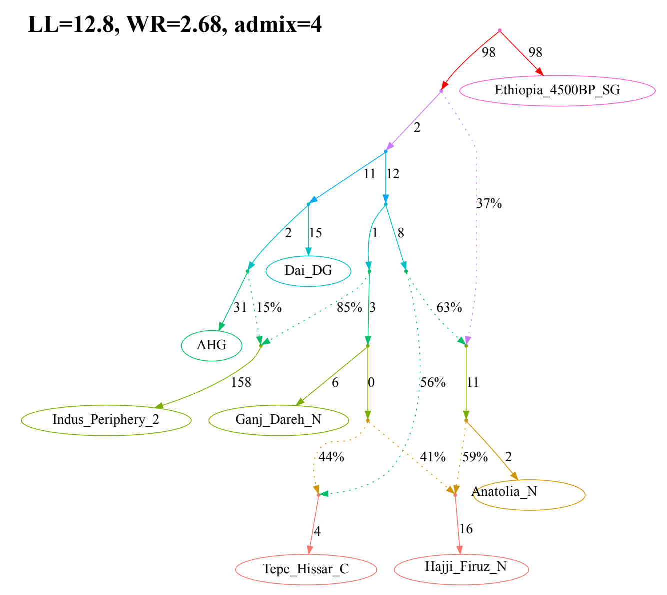

A claim by Shinde *et al*. 2019 relying on the admixture graph:

Primary ancestry in the Indus Periphery group forms the deepest branch in the Iranian Neolithic clade composed of the Indus Periphery, Ganj Dareh Neolithic, Hajji Firuz Neolithic, and Tepe Hissar Chalcolithic groups.

b, alternative models fitting not significantly worse than the highest-ranking one and contradicting the historical interpretation of the admixture graph results by Shinde *et al*. 2019

<table>
<tr>
<td>Relatives (2nd and 3rd degree) and potentially contaminated individuals removed, the Indus Periphery group expanded to 4 ind., the corrected algorithm for calculating f_3-statistics (249,009 variable site with no missing data at the group level)</td>
<td>The original set of SNPs and individuals, and the original algorithm for calculating f_3-statistics (470,389 variable site with no missing data at the group level)</td>
</tr>
<tr>
<td>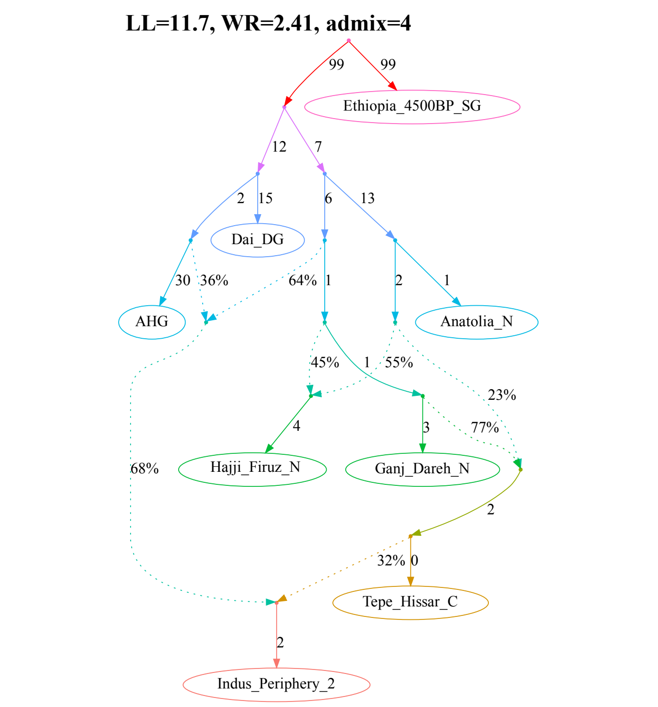
</td>
<td>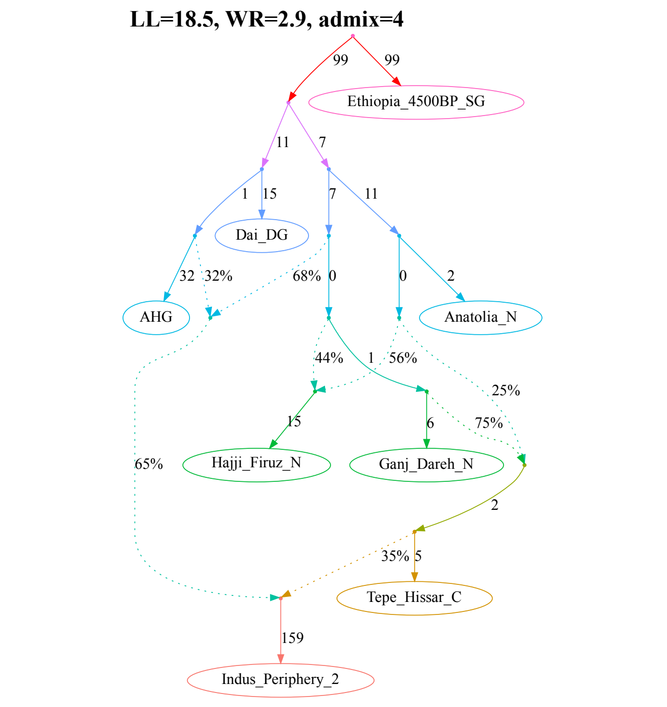
</td>
</tr>
<tr>
<td></td>
<td></td>
</tr>
<tr>
<td>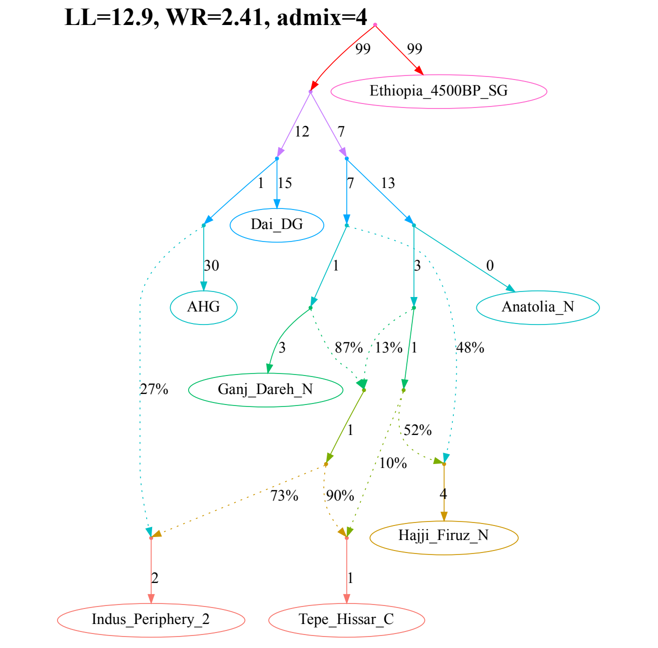
</td>
<td>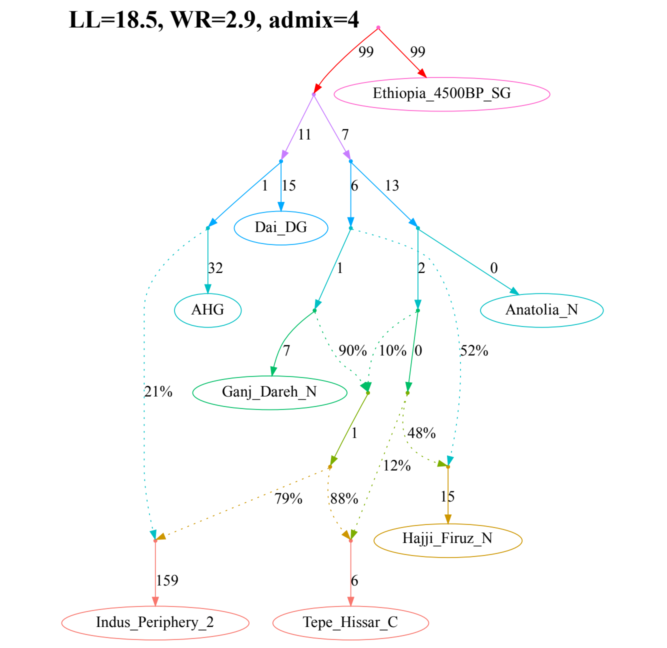
</td>
</tr>
</table>

Relatives (2nd and 3rd degree) and potentially contaminated individuals removed, the Indus Periphery group expanded to 4 ind., the corrected algorithm for calculating f_3-statistics (249,009 variable site with no missing data at the group level)	The original set of SNPs and individuals, and the original algorithm for calculating f_3-statistics (470,389 variable site with no missing data at the group level)

LL=13.3, WR=2.41, admix=4

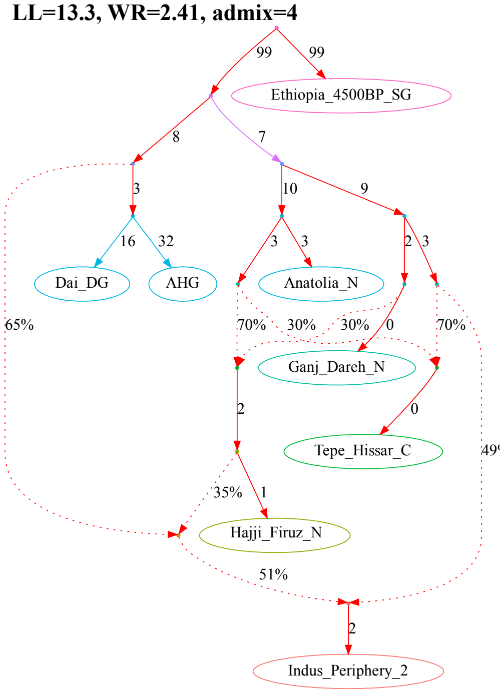

LL=18.6, WR=2.9, admix=4

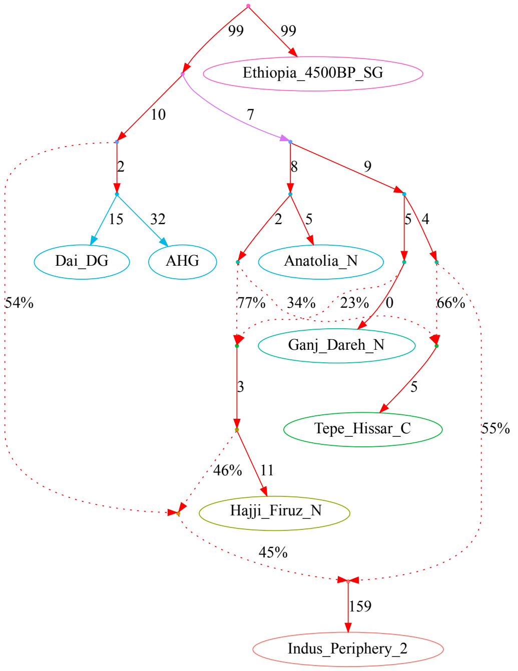

LL=13.1, WR=2.41, admix=4

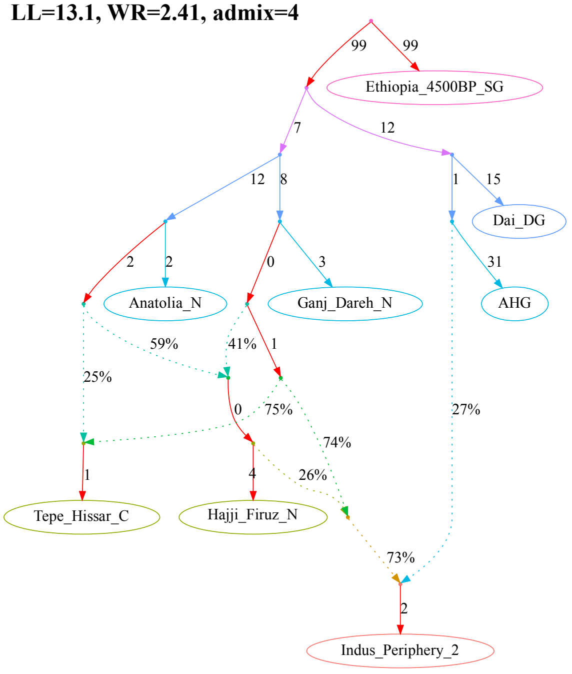

LL=18.5, WR=2.9, admix=4

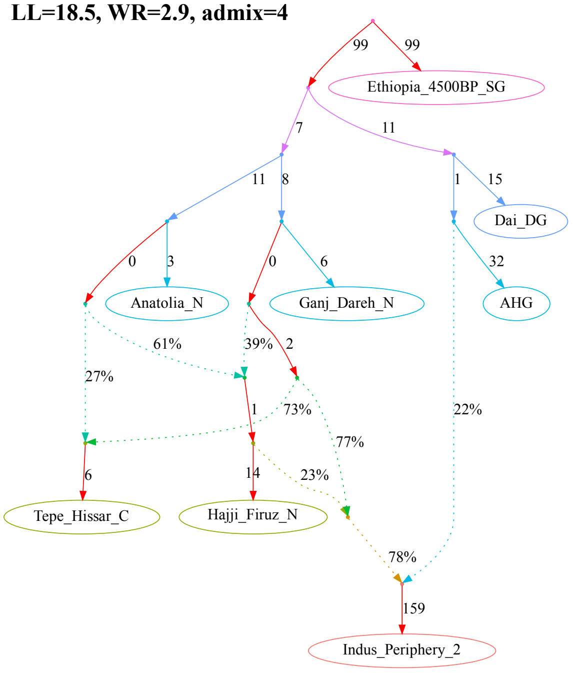

Supplement: Figure 3—source data 6. — The graphs were also re-fitted on the original set of single-nucleotide polymorphisms (SNPs)/individuals and using the original algorithm for calculating f-statistics. Model parameters (graph edges) that were inferred to be unidentifiable are plotted in red. (a) The highest-ranking model with four admixture events that confirms all important features of the published model with three admixture events. The following claim in Shinde et al., 2019 relies on the admixture graph: Primary ancestry in the Indus Periphery group forms the deepest branch in the Iranian Neolithic clade composed of the Indus Periphery, Ganj Dareh Neolithic, Hajji Firuz Neolithic, and Tepe Hissar Chalcolithic groups. (b) Alternative models fitting not significantly worse than the highest-ranking one and contradicting the historical interpretation of the admixture graph results by Shinde et al., 2019. [file elife-85492-fig3-data6.pdf]
