# Supplementary material for: On the limits of fitting complex models of population history to f-statistics
Source: eLife. 2023 Jun 29;12:e85492. doi: 10.7554/eLife.85492 (PMC10310323; doi:10.7554/eLife.85492)

Figure 3–source data 7. Published admixture graphs from Librado *et al*. (2019) and alternative graphs found with *findGraphs* (10 populations, 3 to 5 admixture events) for the modified group composition and using the updated algorithm for calculating *f*-statistics. The graphs were also re-fitted on the original set of SNPs/individuals and using the original algorithm for calculating *f*-statistics. Selected alternative graphs found with *findGraphs* when more admixture events were allowed (from 6 to 9) are also shown. Model parameters (graph edges) that were inferred to be unidentifiable are plotted in red.

a, published model, 3 admixture events

**claim 1** yes<br>**claim 2** yes<br>**claim 3** yes<br>**claim 4** yes<br>**claim 5** yes	**claim 1** yes<br>**claim 2** yes<br>**claim 3** yes<br>**claim 4** yes<br>**claim 5** yes
The modified set of individuals and the corrected algorithm for calculating f_3-statistics (1,767,419 variable site with no missing data at the group level)	The modified set of individuals and the original algorithm for calculating f_3-statistics (6,343,116 variable site with no missing data at the group level)

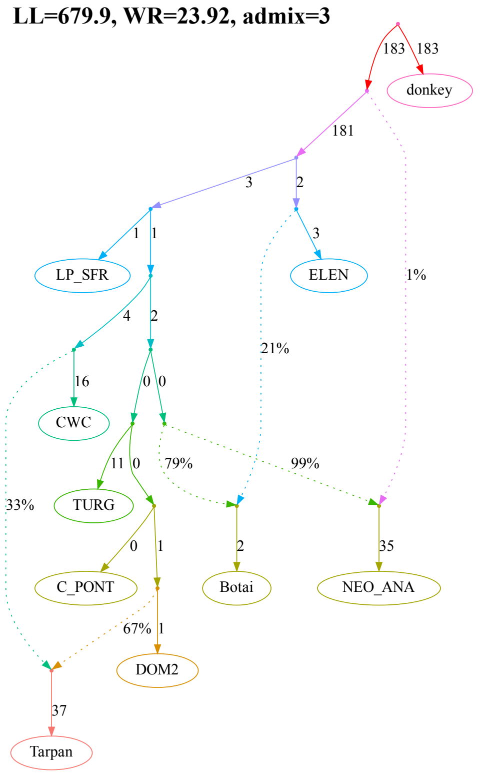

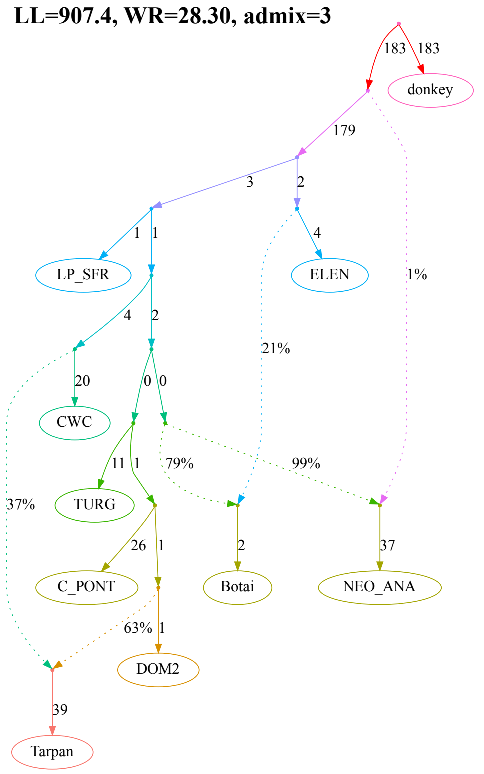

Claims by Librado *et al*. 2021 relying on the admixture graph:

1) NEO-ANA-related admixture is absent in DOM2;
2) DOM2 and C-PONT are sister groups;
3) there is no gene flow connecting the CWC and the cluster associated with Yamnaya horses and horses of the later Sintashta culture whose ancestry is maximized in the Western Steppe (DOM2, C-PONT, TURG);
4) there was a gene flow from a deep-branching ghost group to NEO-ANA;
5) Tarpan is a mixture of a CWC-related and a DOM2-related lineage.

b, an alternative model with 3 admixture events fitting significantly better than the published one

<table>
<tr>
<td>

**claim 1**	yes
**claim 2**	yes
**claim 3**	yes
**claim 4**	yes
**claim 5**	no

</td>
<td>

**claim 1**	yes
**claim 2**	yes
**claim 3**	yes
**claim 4**	yes
**claim 5**	no

</td>
</tr>
<tr>
<td>The modified set of individuals and the corrected algorithm for calculating f_3-statistics (1,767,419 variable site with no missing data at the group level)</td>
<td>The modified set of individuals and the original algorithm for calculating f_3-statistics (6,343,116 variable site with no missing data at the group level)</td>
</tr>
<tr>
<td>

LL=508.3, WR=16.99, admix=3

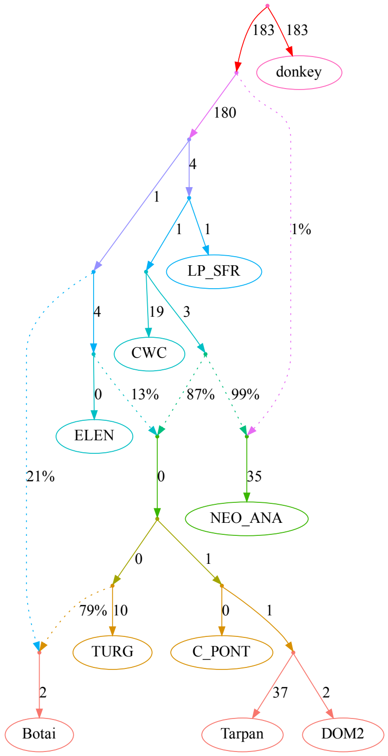

</td>
<td>

LL=773.9, WR=19.85, admix=3

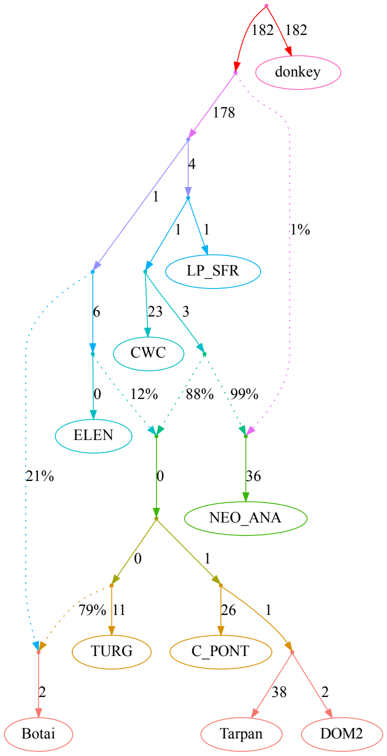

</td>
</tr>
</table>

c, published model, 4 admixture events

**claim 1** yes<br>**claim 2** yes<br>**claim 3** yes<br>**claim 4** yes<br>**claim 5** yes	**claim 1** yes<br>**claim 2** yes<br>**claim 3** yes<br>**claim 4** yes<br>**claim 5** yes
The modified set of individuals and the corrected algorithm for calculating f_3-statistics (1,767,419 variable site with no missing data at the group level)	The modified set of individuals and the original algorithm for calculating f_3-statistics (6,343,116 variable site with no missing data at the group level)

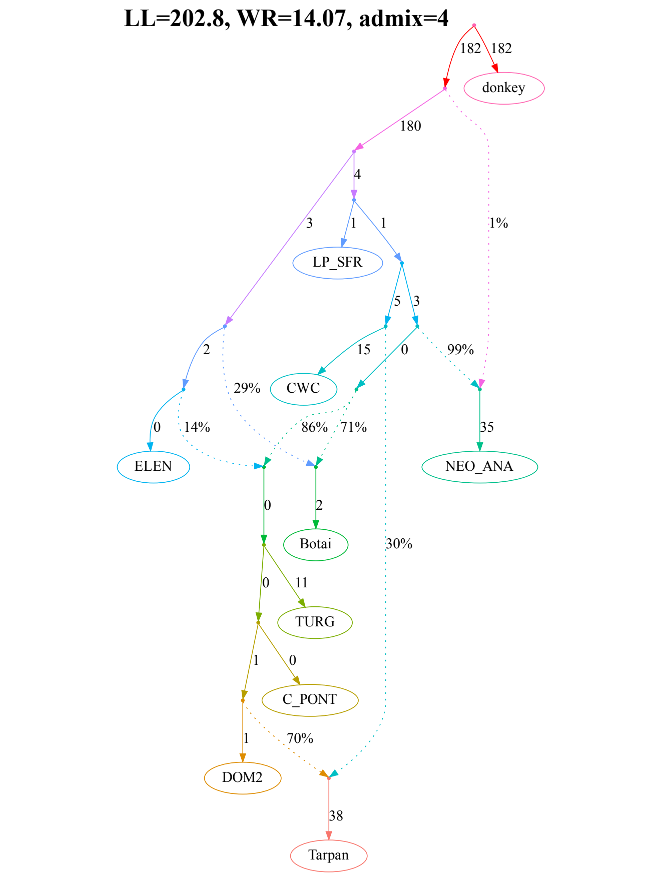

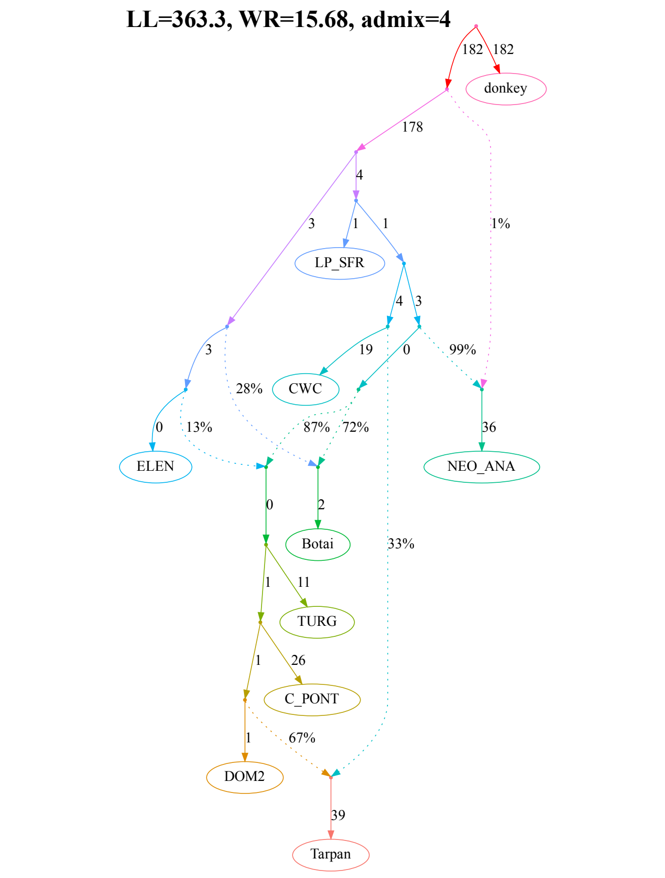

d, an alternative model with 4 admixture events fitting not significantly worse than the published one

**claim 1** yes<br>**claim 2** yes<br>**claim 3** yes<br>**claim 4** yes<br>**claim 5** yes	**claim 1** yes<br>**claim 2** yes<br>**claim 3** yes<br>**claim 4** yes<br>**claim 5** yes
The modified set of individuals and the corrected algorithm for calculating f_3-statistics (1,767,419 variable site with no missing data at the group level)	The modified set of individuals and the original algorithm for calculating f_3-statistics (6,343,116 variable site with no missing data at the group level)
**LL=204.9, WR=10.16, admix=4**<br>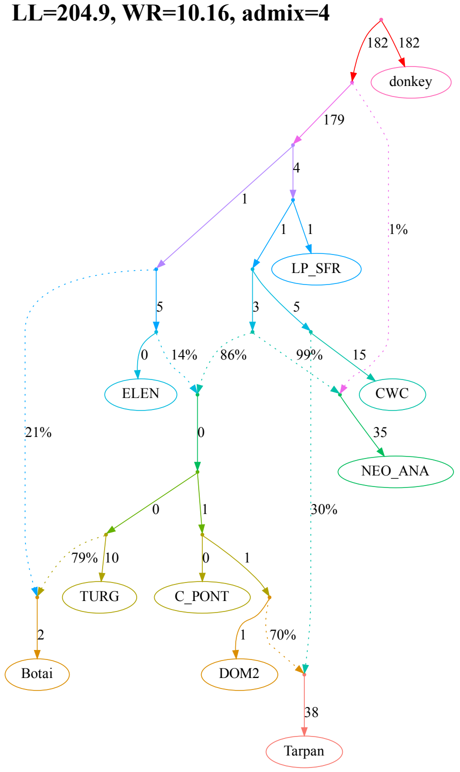 	**LL=378.1, WR=12.42, admix=4**<br>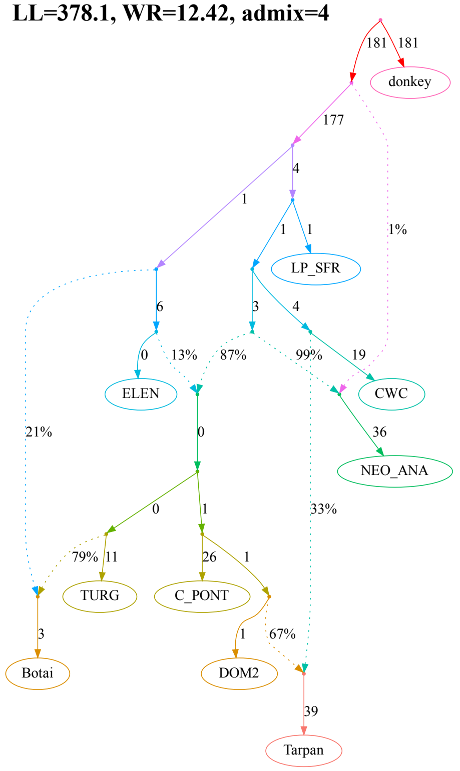

e, published model, 5 admixture events

**claim 1** yes<br>**claim 2** yes<br>**claim 3** yes<br>**claim 4** yes<br>**claim 5** yes	**claim 1** yes<br>**claim 2** yes<br>**claim 3** yes<br>**claim 4** yes<br>**claim 5** yes
The modified set of individuals and the corrected algorithm for calculating f_3-statistics (1,767,419 variable site with no missing data at the group level)	The modified set of individuals and the original algorithm for calculating f_3-statistics (6,343,116 variable site with no missing data at the group level)
**LL=120.9, WR=6.86, admix=5** 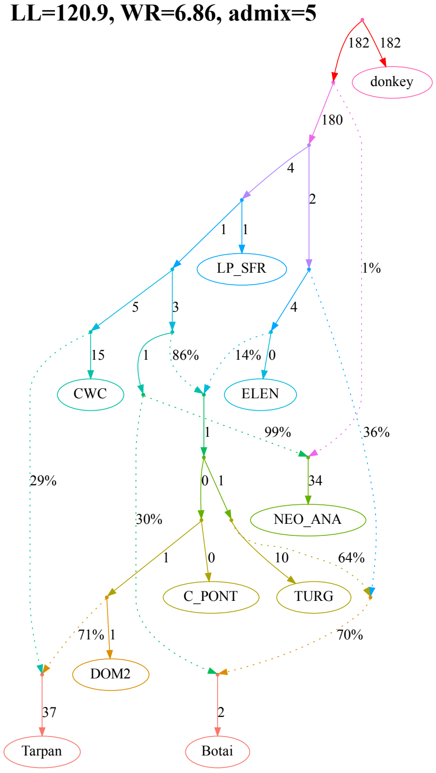	**LL=267.7, WR=10.40, admix=5** 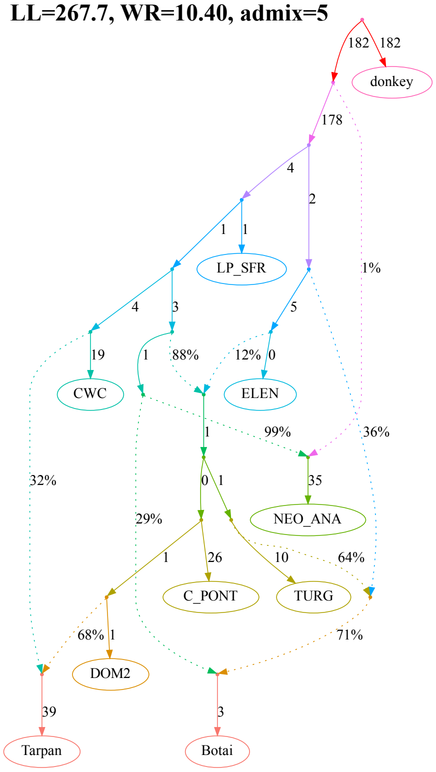

f, an alternative model with 5 admixture events fitting not significantly worse than the published one

<table>
<tr>
<td>claim 1 yes
claim 2 yes
claim 3 no
claim 4 yes
claim 5 yes</td>
<td>claim 1 yes
claim 2 yes
claim 3 no
claim 4 yes
claim 5 yes</td>
</tr>
<tr>
<td>The modified set of individuals and the corrected algorithm for calculating f_3-statistics (1,767,419 variable site with no missing data at the group level)</td>
<td>The modified set of individuals and the original algorithm for calculating f_3-statistics (6,343,116 variable site with no missing data at the group level)</td>
</tr>
<tr>
<td>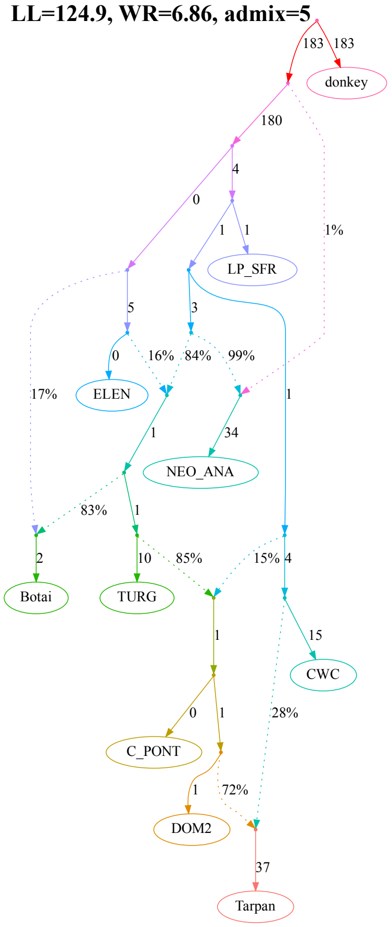
</td>
<td>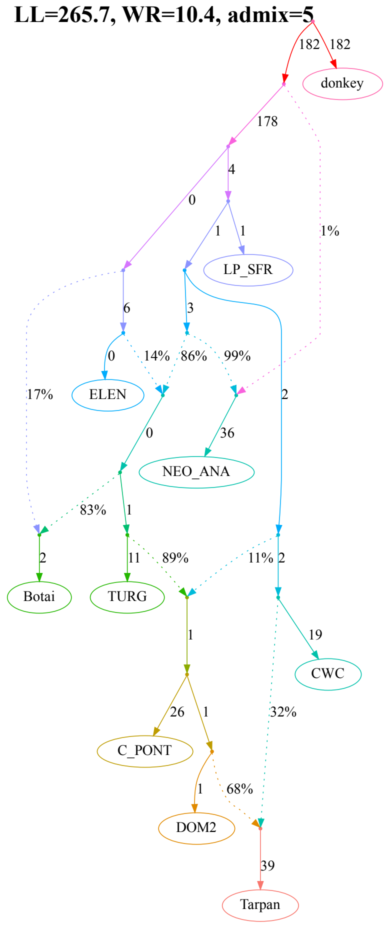
</td>
</tr>
</table>

g, selected models with 6 admixture events

**claim 1** yes<br>**claim 2** yes<br>**claim 3** no<br>**claim 4** yes<br>**claim 5** yes	**claim 1** yes<br>**claim 2** yes<br>**claim 3** no<br>**claim 4** yes<br>**claim 5** yes
The modified set of individuals and the corrected algorithm for calculating f_3-statistics (1,767,419 variable site with no missing data at the group level)	The modified set of individuals and the original algorithm for calculating f_3-statistics (6,343,116 variable site with no missing data at the group level)

LL=66.2, WR=5.03, admix=6

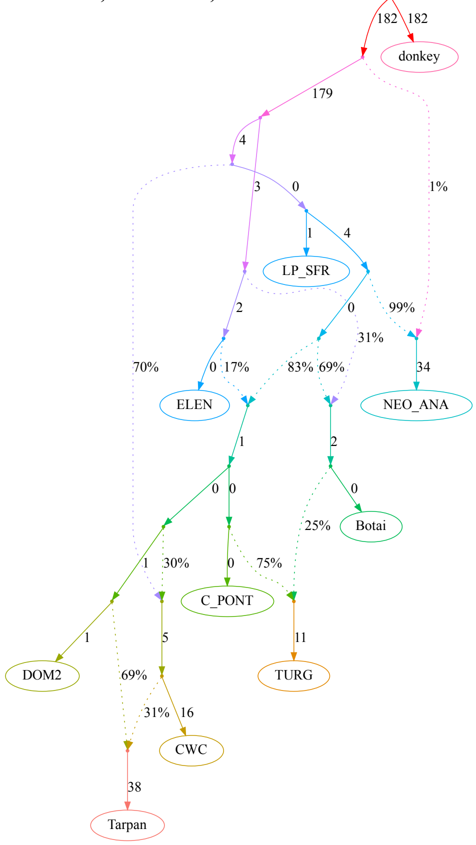

LL=109.1, WR=7.76, admix=6

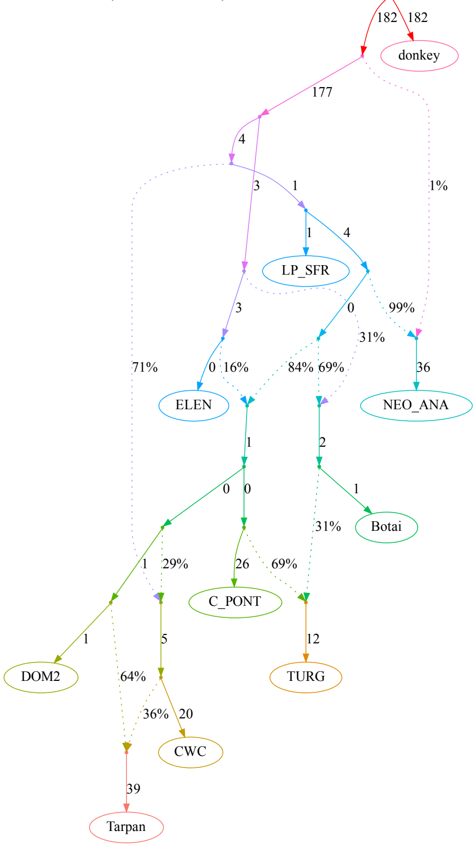

<table>
<tr>
<td>claim 1 no
claim 2 yes
claim 3 no
claim 4 no
claim 5 yes</td>
<td>claim 1 no
claim 2 yes
claim 3 no
claim 4 no
claim 5 yes</td>
</tr>
<tr>
<td>The modified set of individuals and the corrected algorithm for calculating f_3-statistics (1,767,419 variable site with no missing data at the group level)</td>
<td>The modified set of individuals and the original algorithm for calculating f_3-statistics (6,343,116 variable site with no missing data at the group level)</td>
</tr>
<tr>
<td>

LL=68, WR=5.28, admix=6

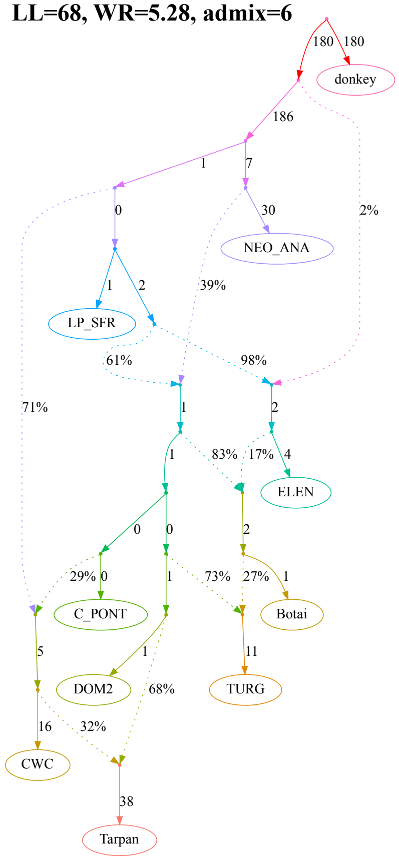

</td>
<td>

LL=131.6, WR=8.1, admix=6

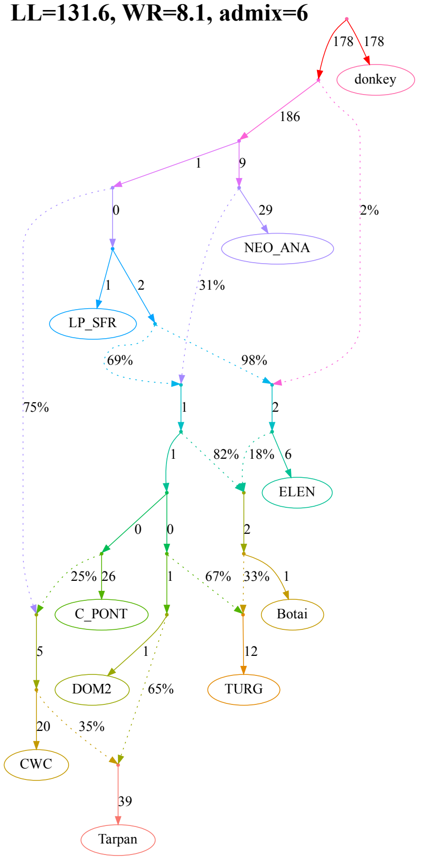

</td>
</tr>
</table>

h, selected models with 7 admixture events (all plausible models with WR < 5 SE)

**claim 1** yes<br>**claim 2** yes<br>**claim 3** no<br>**claim 4** yes<br>**claim 5** no	**claim 1** yes<br>**claim 2** yes<br>**claim 3** no<br>**claim 4** yes<br>**claim 5** no
The modified set of individuals and the corrected algorithm for calculating f_3-statistics (1,767,419 variable site with no missing data at the group level)	The modified set of individuals and the original algorithm for calculating f_3-statistics (6,343,116 variable site with no missing data at the group level)
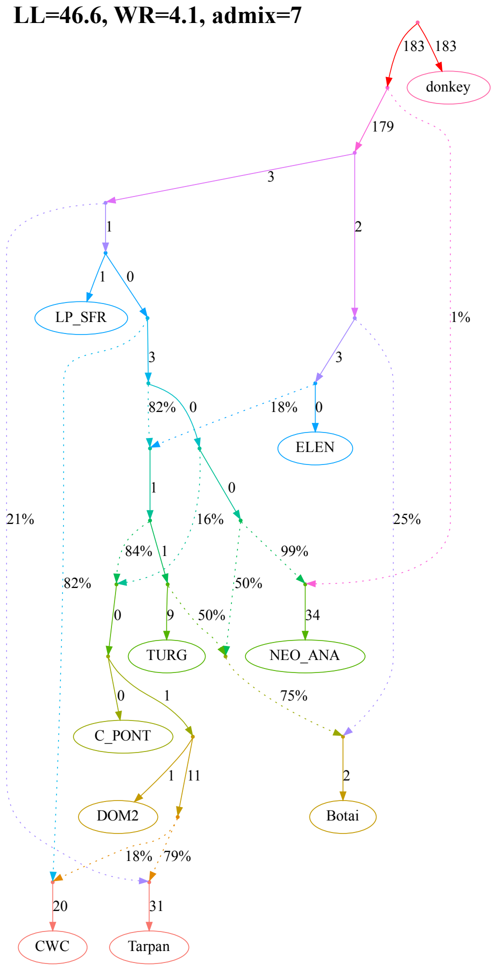	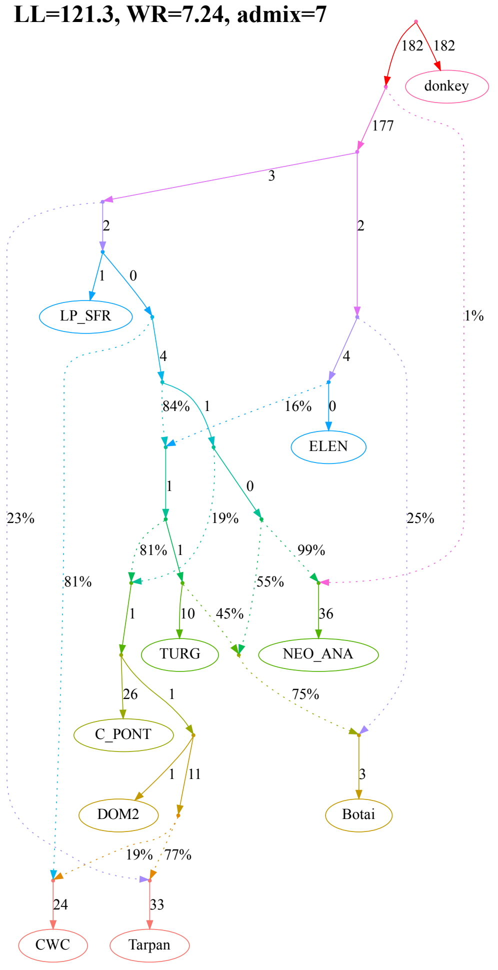

**claim 1** yes<br>**claim 2** yes<br>**claim 3** no<br>**claim 4** yes<br>**claim 5** yes	**claim 1** yes<br>**claim 2** yes<br>**claim 3** no<br>**claim 4** yes<br>**claim 5** yes
The modified set of individuals and the corrected algorithm for calculating f_3-statistics (1,767,419 variable site with no missing data at the group level)	The modified set of individuals and the original algorithm for calculating f_3-statistics (6,343,116 variable site with no missing data at the group level)
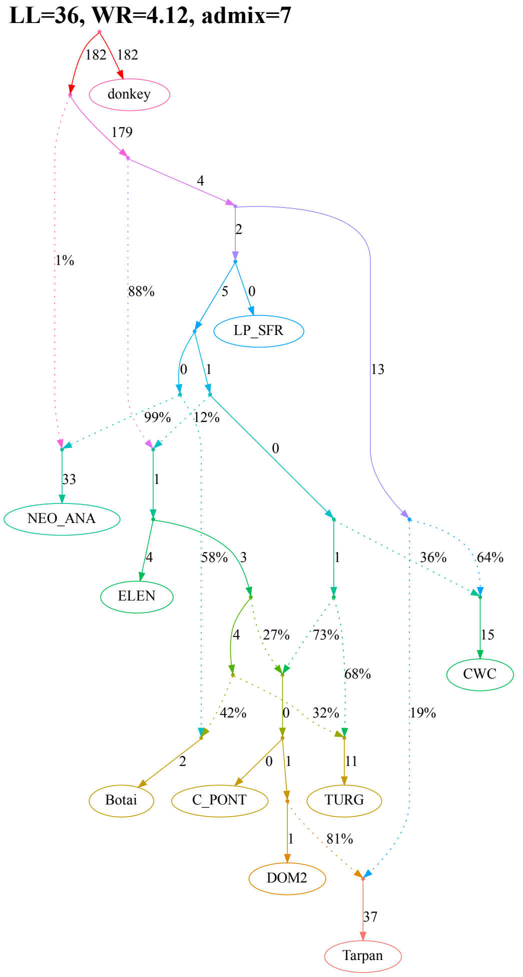	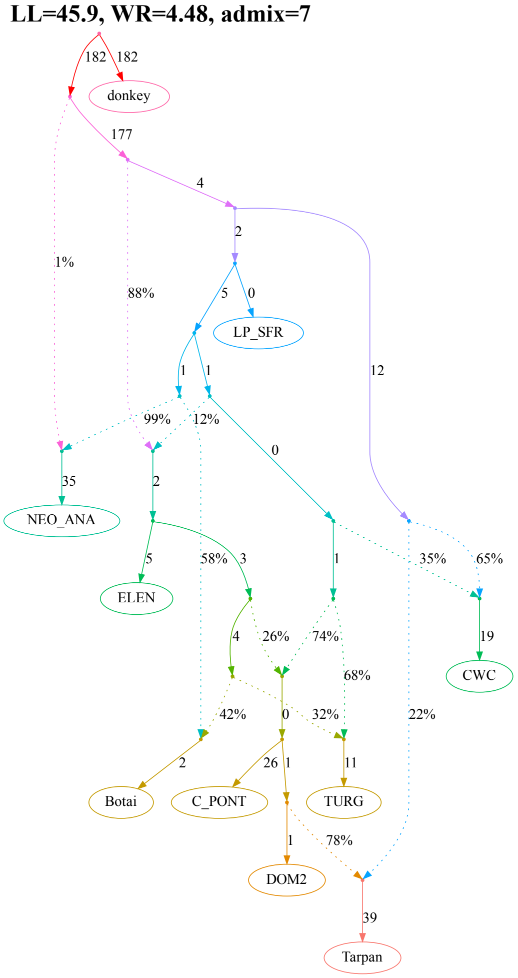

i, selected models with 7 admixture events (all plausible models with WR < 5 SE)

<table>
<tr>
<td>claim 1 yes
claim 2 yes
claim 3 no
claim 4 yes
claim 5 yes</td>
<td>claim 1 yes
claim 2 yes
claim 3 no
claim 4 yes
claim 5 yes</td>
</tr>
<tr>
<td>The modified set of individuals and the corrected algorithm for calculating f$_3$-statistics (1,767,419 variable site with no missing data at the group level)</td>
<td>The modified set of individuals and the original algorithm for calculating f$_3$-statistics (6,343,116 variable site with no missing data at the group level)</td>
</tr>
<tr>
<td>LL=53.3, WR=4.49, admix=7
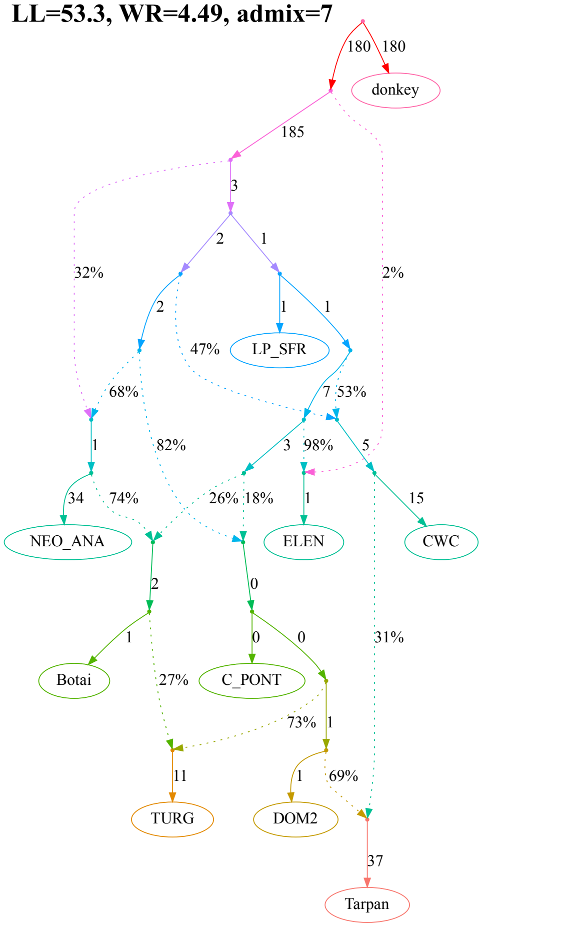
</td>
<td>LL=76.7, WR=7.03, admix=7
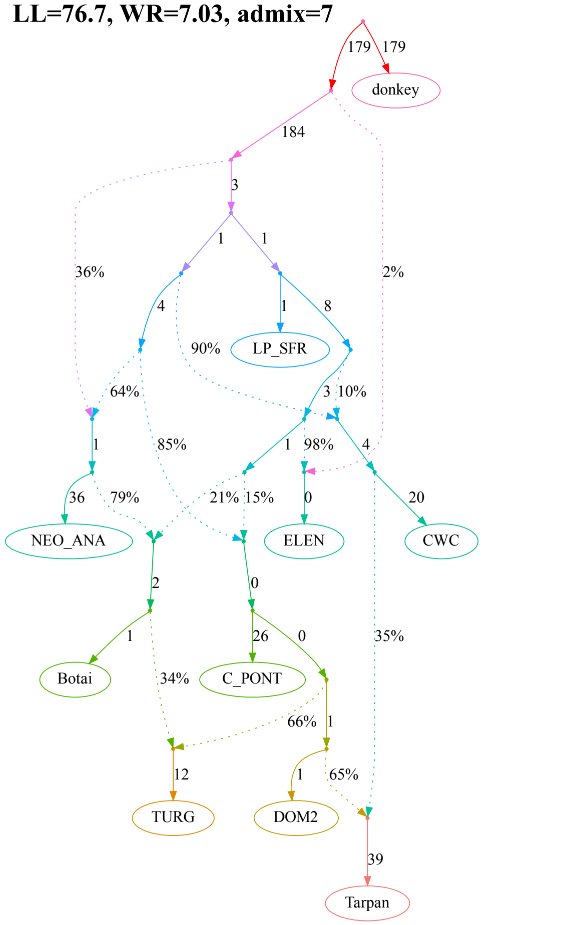
</td>
</tr>
</table>

<table>
<tr>
<td>claim 1 no
claim 2 yes
claim 3 no
claim 4 yes
claim 5 no</td>
<td>claim 1 no
claim 2 yes
claim 3 no
claim 4 yes
claim 5 no</td>
</tr>
<tr>
<td>The modified set of individuals and the corrected algorithm for calculating f_3-statistics (1,767,419 variable site with no missing data at the group level)</td>
<td>The modified set of individuals and the original algorithm for calculating f_3-statistics (6,343,116 variable site with no missing data at the group level)</td>
</tr>
<tr>
<td>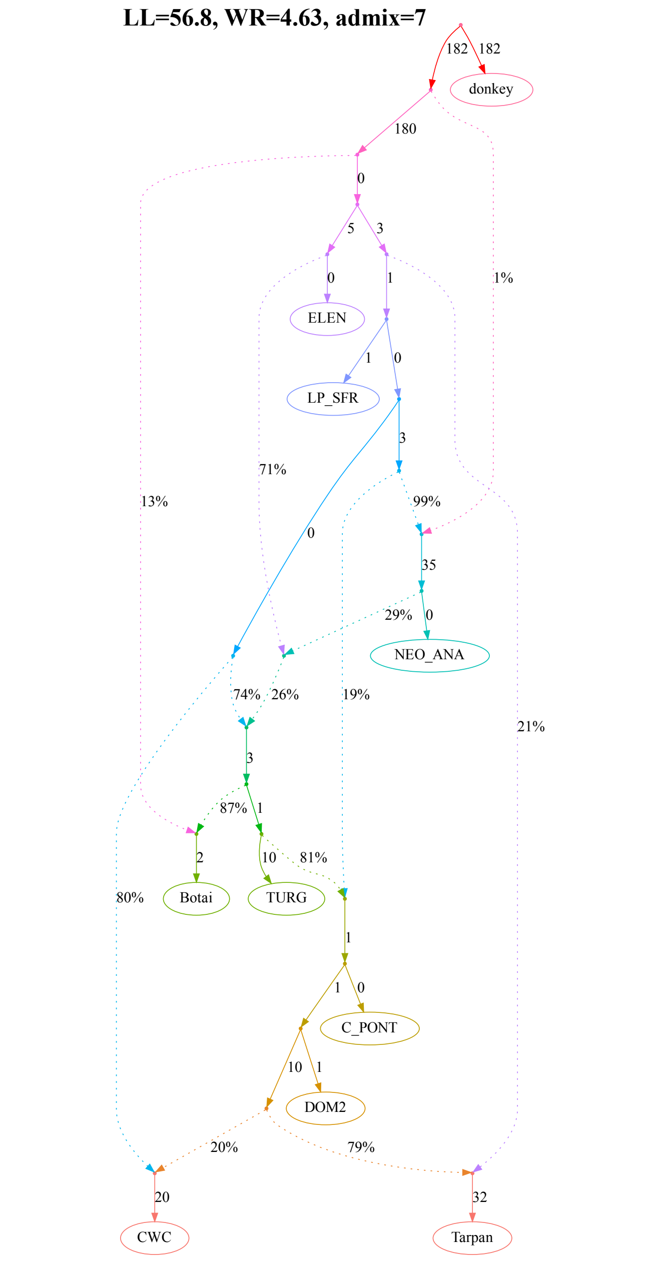
</td>
<td>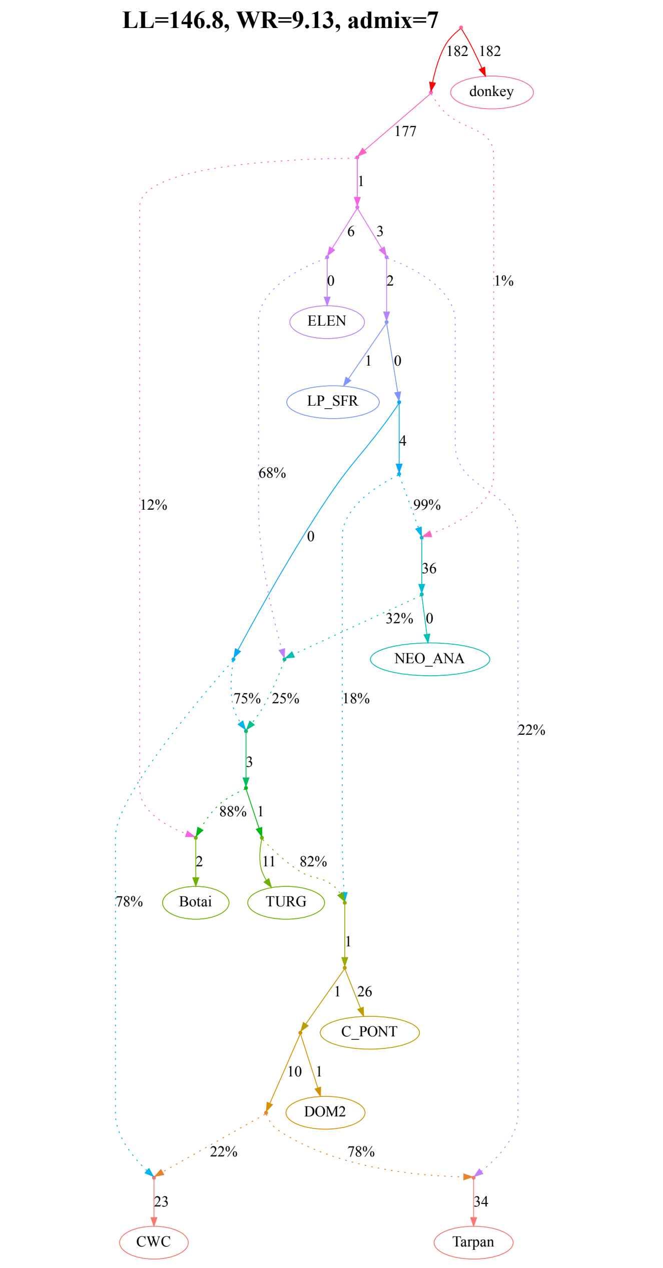
</td>
</tr>
</table>

j, selected models with 8 admixture events (all plausible models with WR < 4 SE)

**claim 1** no<br>**claim 2** yes<br>**claim 3** no<br>**claim 4** yes<br>**claim 5** no	**claim 1** no<br>**claim 2** yes<br>**claim 3** no<br>**claim 4** yes<br>**claim 5** no
The modified set of individuals and the corrected algorithm for calculating f_3-statistics (1,767,419 variable site with no missing data at the group level)	The modified set of individuals and the original algorithm for calculating f_3-statistics (6,343,116 variable site with no missing data at the group level)

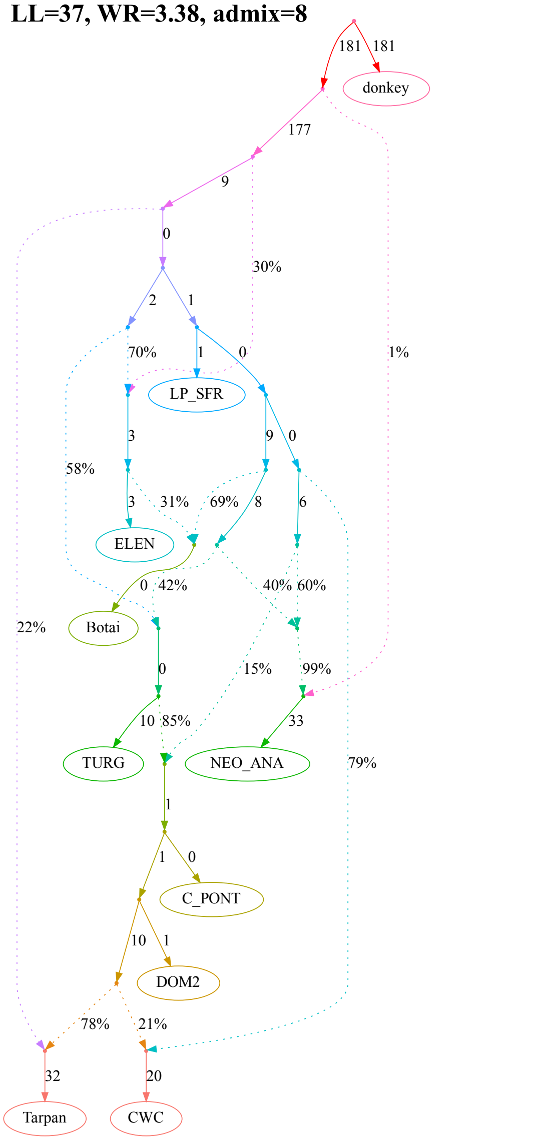

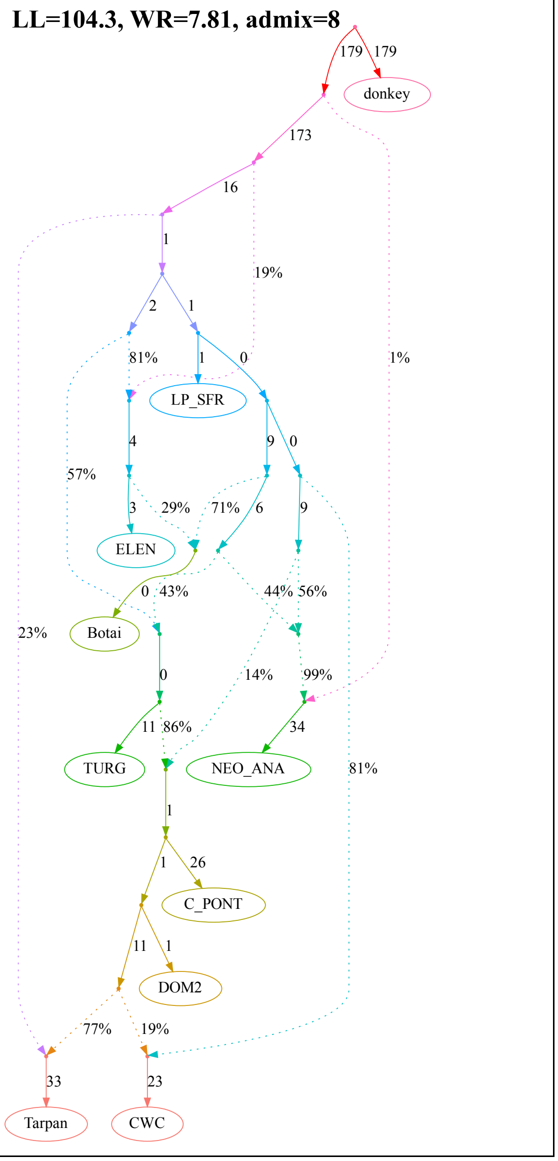

**claim 1**	no
**claim 2**	yes
**claim 3**	yes
**claim 4**	yes
**claim 5**	no

The modified set of individuals and the corrected algorithm for calculating f_3-statistics (1,767,419 variable site with no missing data at the group level)

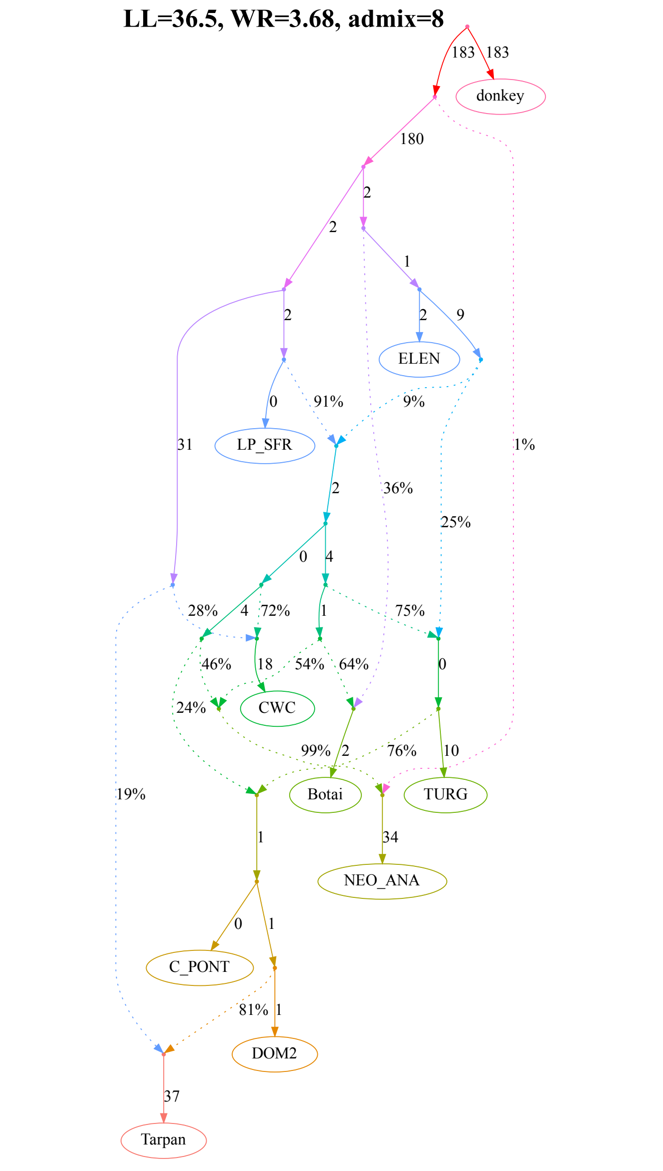

**claim 1**	no
**claim 2**	yes
**claim 3**	yes
**claim 4**	yes
**claim 5**	no

The modified set of individuals and the original algorithm for calculating f_3-statistics (6,343,116 variable site with no missing data at the group level)

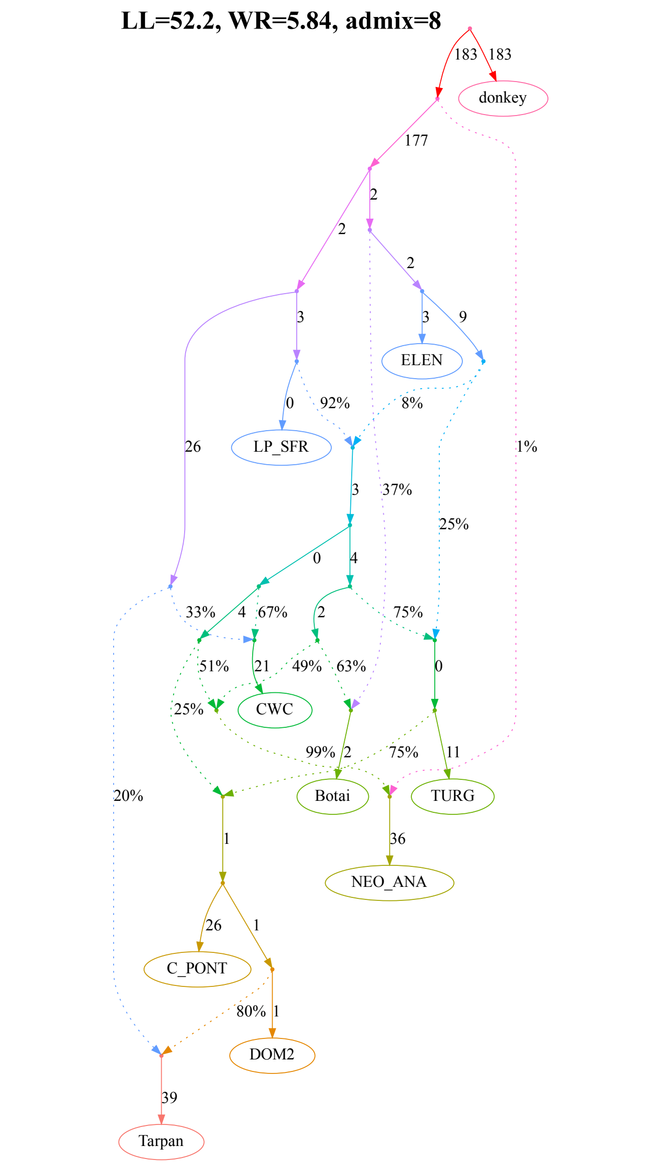

k, selected models with 8 admixture events (all plausible models with WR < 4 SE)

**claim 1** yes<br>**claim 2** yes<br>**claim 3** no<br>**claim 4** yes<br>**claim 5** yes	**claim 1** yes<br>**claim 2** yes<br>**claim 3** no<br>**claim 4** yes<br>**claim 5** yes
The modified set of individuals and the corrected algorithm for calculating f_3-statistics (1,767,419 variable site with no missing data at the group level)	The modified set of individuals and the original algorithm for calculating f_3-statistics (6,343,116 variable site with no missing data at the group level)

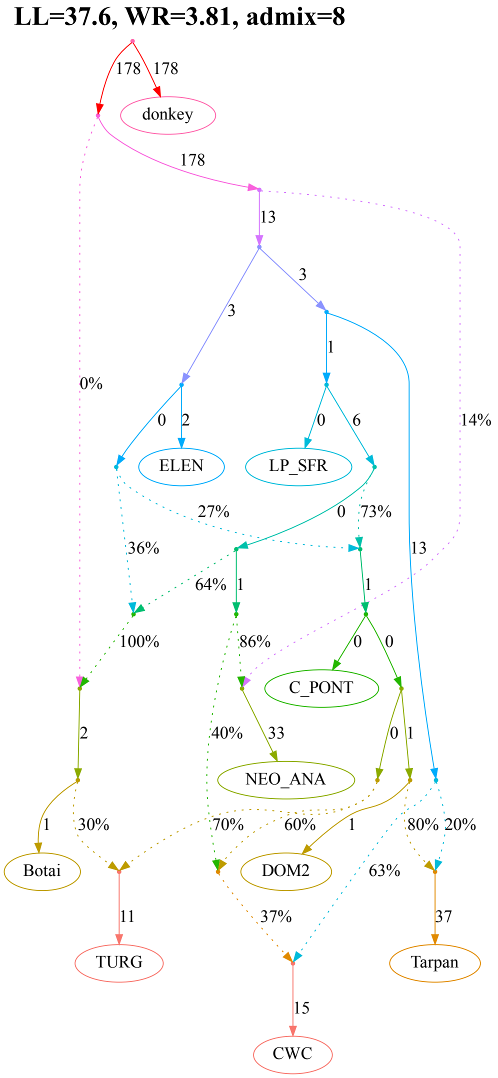

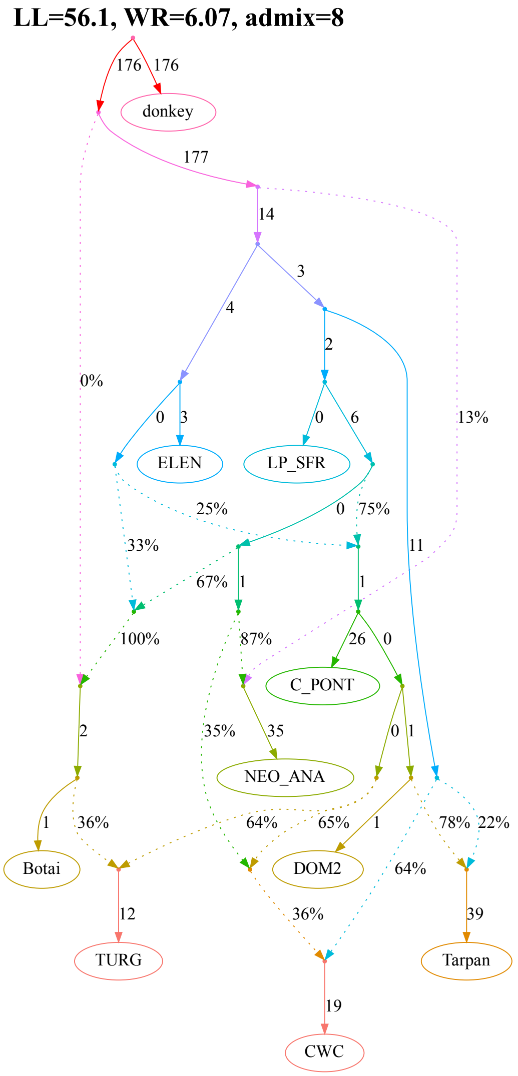

<table>
<tr>
<td>

**claim 1**	yes
**claim 2**	yes
**claim 3**	yes
**claim 4**	yes
**claim 5**	yes

</td>
<td>

**claim 1**	yes
**claim 2**	yes
**claim 3**	yes
**claim 4**	yes
**claim 5**	yes

</td>
</tr>
<tr>
<td>The modified set of individuals and the corrected algorithm for calculating f_3-statistics (1,767,419 variable site with no missing data at the group level)</td>
<td>The modified set of individuals and the original algorithm for calculating f_3-statistics (6,343,116 variable site with no missing data at the group level)</td>
</tr>
<tr>
<td>

LL=48.4, WR=3.85, admix=8

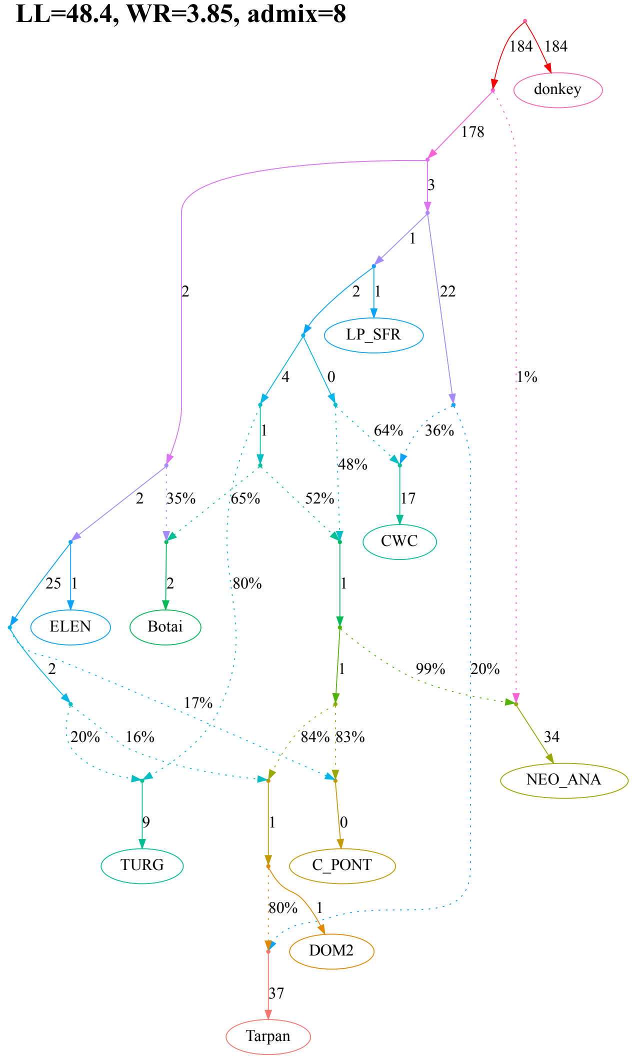

</td>
<td>

LL=71.9, WR=5.84, admix=8

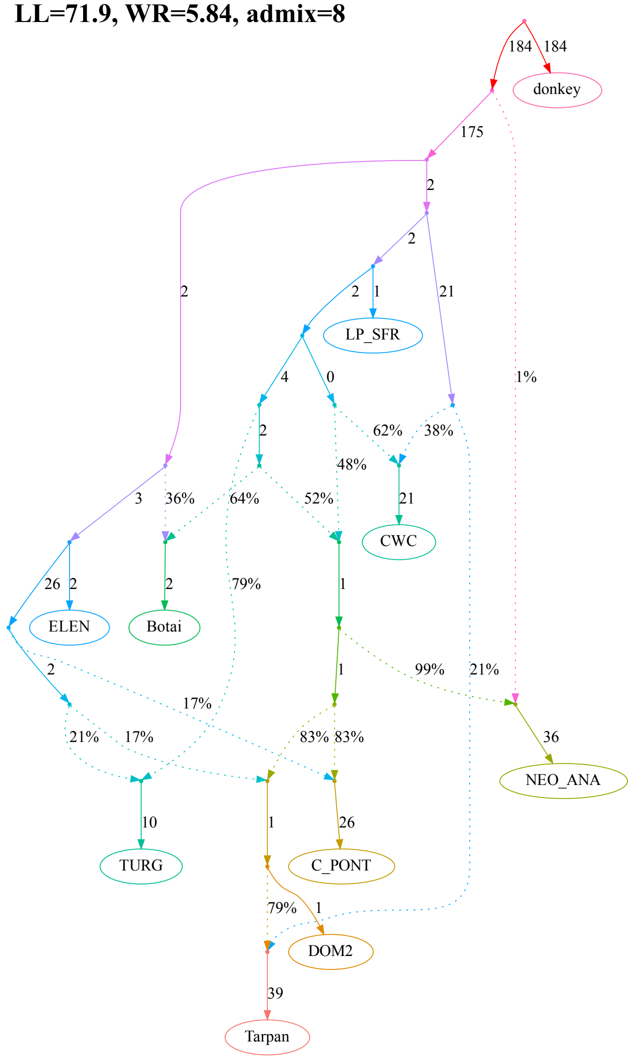

</td>
</tr>
</table>

I, selected models with 8 admixture events (all plausible models with WR < 4 SE)

**claim 1** yes<br>**claim 2** yes<br>**claim 3** no<br>**claim 4** yes<br>**claim 5** yes	**claim 1** yes<br>**claim 2** yes<br>**claim 3** no<br>**claim 4** yes<br>**claim 5** yes
The modified set of individuals and the corrected algorithm for calculating f_3-statistics (1,767,419 variable site with no missing data at the group level)	The modified set of individuals and the original algorithm for calculating f_3-statistics (6,343,116 variable site with no missing data at the group level)

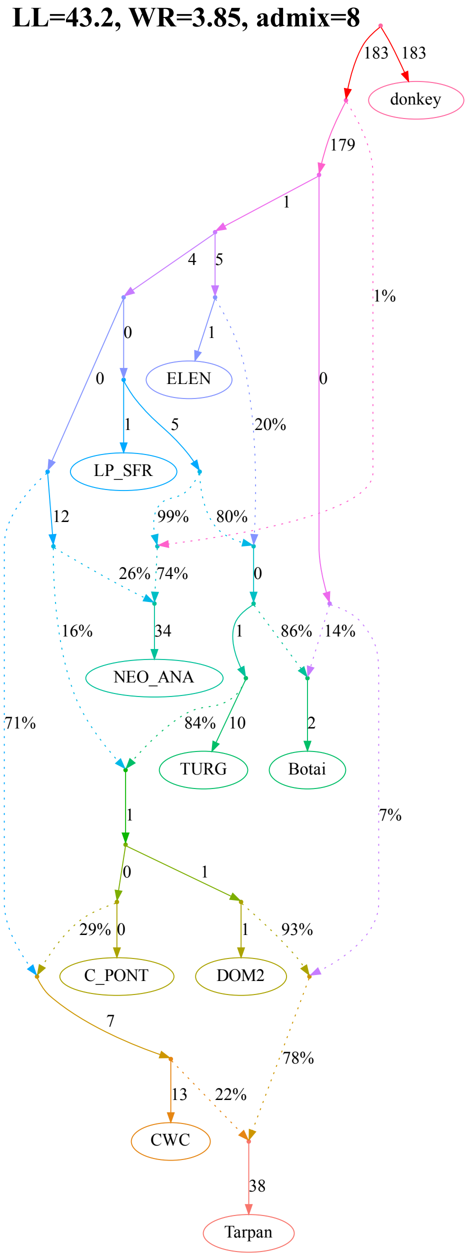

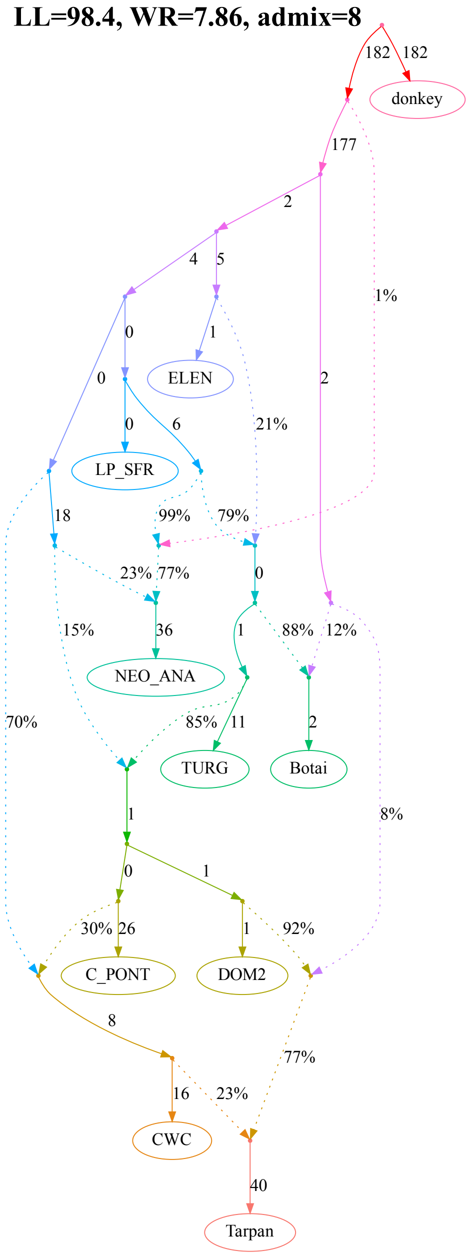

m, selected models with 9 admixture events (all plausible models with WR < 4 SE)

**claim 1** no<br>**claim 2** yes<br>**claim 3** no<br>**claim 4** no<br>**claim 5** yes	**claim 1** no<br>**claim 2** yes<br>**claim 3** no<br>**claim 4** no<br>**claim 5** yes
The modified set of individuals and the corrected algorithm for calculating f_3-statistics (1,767,419 variable site with no missing data at the group level)	The modified set of individuals and the original algorithm for calculating f_3-statistics (6,343,116 variable site with no missing data at the group level)

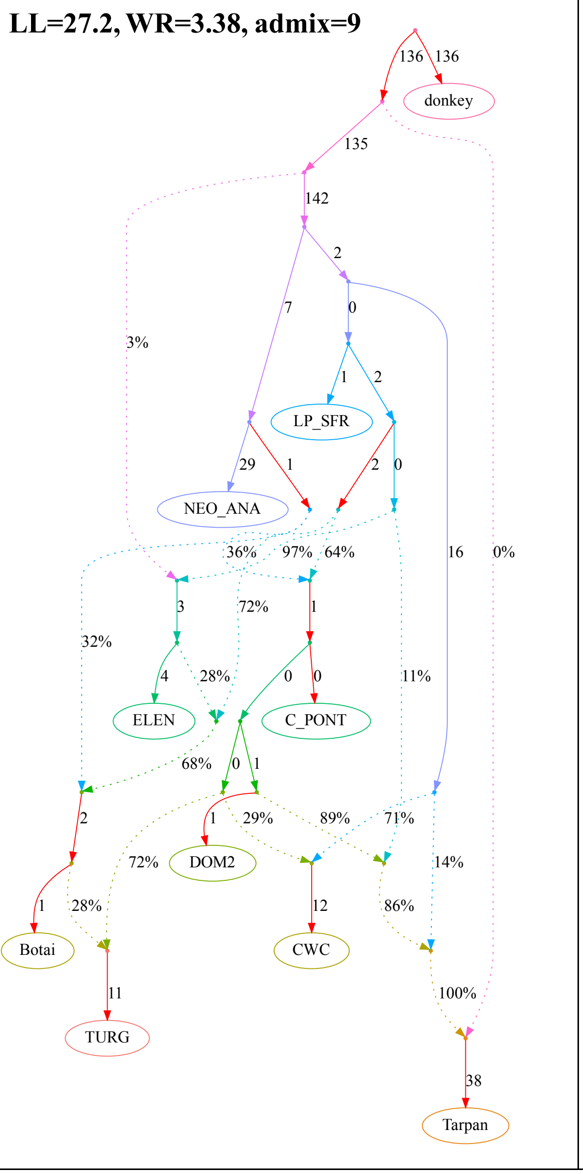

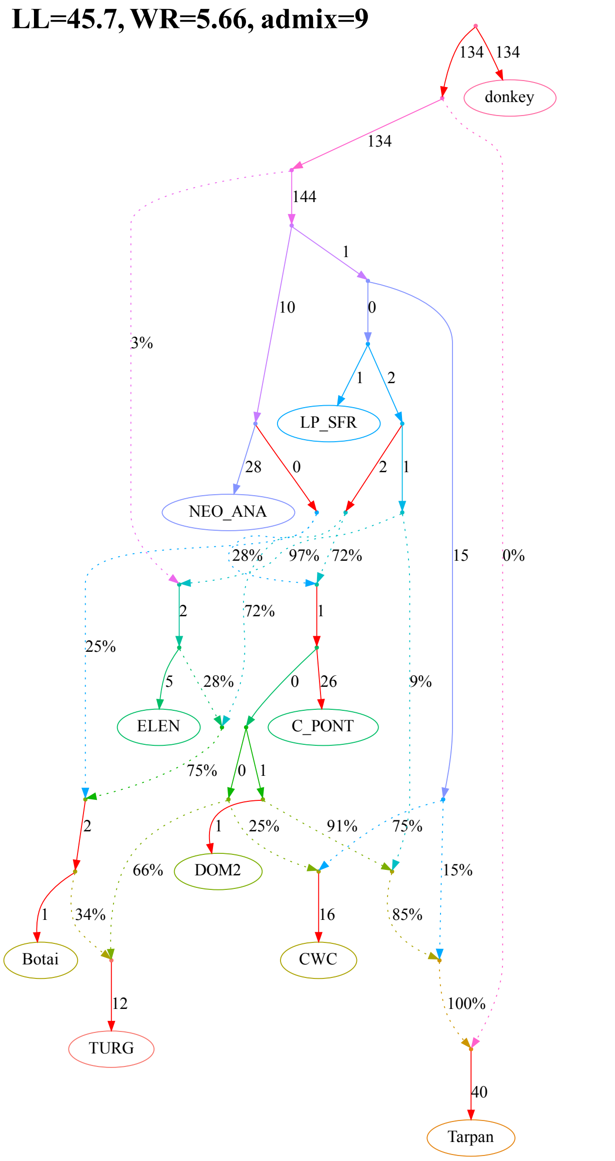

claim 1 no<br>claim 2 yes<br>claim 3 no<br>claim 4 yes<br>claim 5 no	claim 1 no<br>claim 2 yes<br>claim 3 no<br>claim 4 yes<br>claim 5 no
The modified set of individuals and the corrected algorithm for calculating f_3-statistics (1,767,419 variable site with no missing data at the group level)	The modified set of individuals and the original algorithm for calculating f_3-statistics (6,343,116 variable site with no missing data at the group level)

LL=26.6, WR=3.38, admix=9

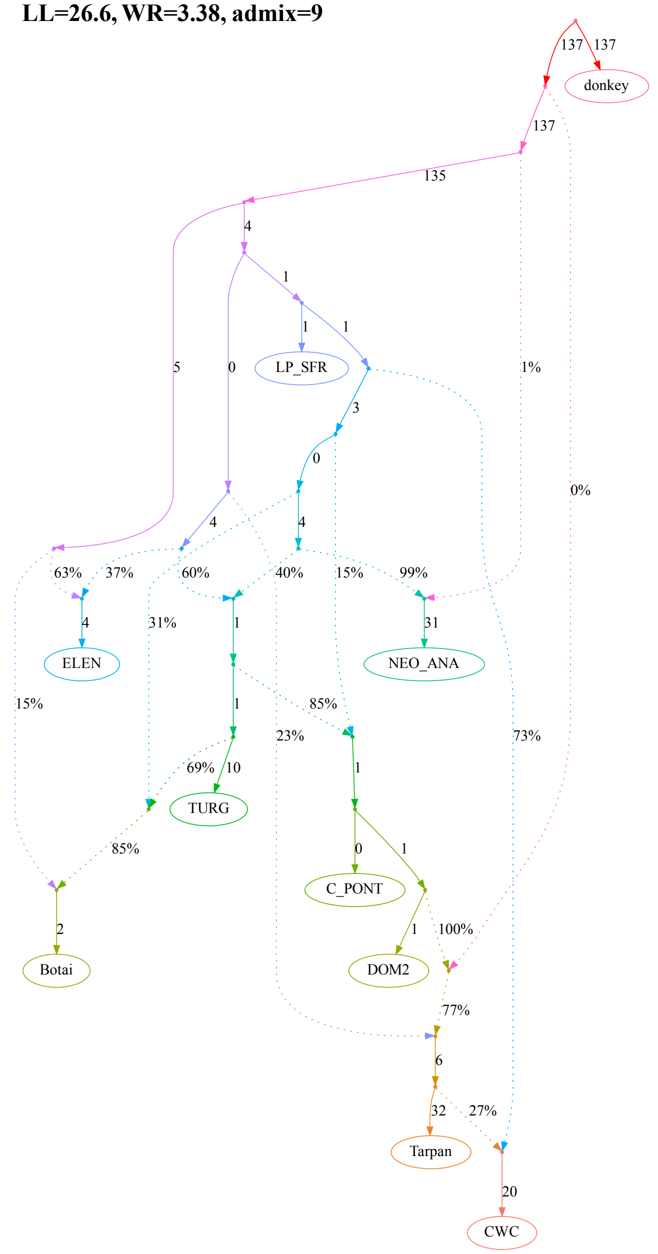

LL=81.4, WR=6.39, admix=9

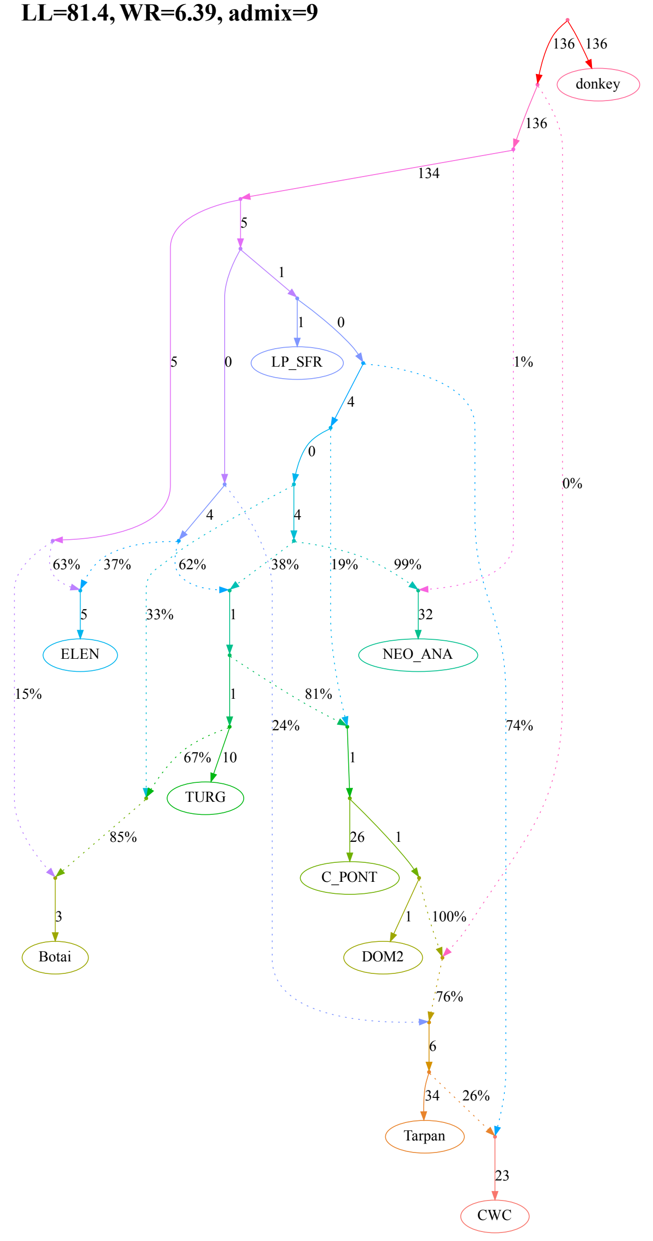

n, selected models with 9 admixture events (all plausible models with WR < 4 SE)

**claim 1** yes<br>**claim 2** yes<br>**claim 3** no<br>**claim 4** yes<br>**claim 5** no	**claim 1** yes<br>**claim 2** yes<br>**claim 3** no<br>**claim 4** yes<br>**claim 5** no
The modified set of individuals and the corrected algorithm for calculating f_3-statistics (1,767,419 variable site with no missing data at the group level)	The modified set of individuals and the original algorithm for calculating f_3-statistics (6,343,116 variable site with no missing data at the group level)
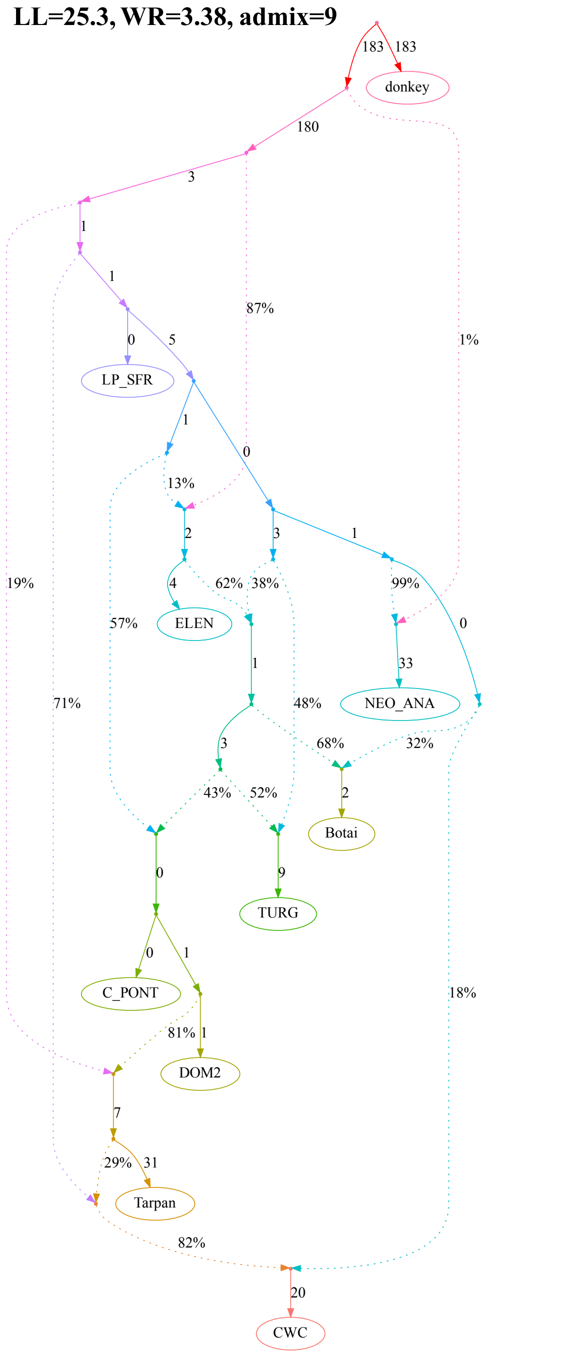	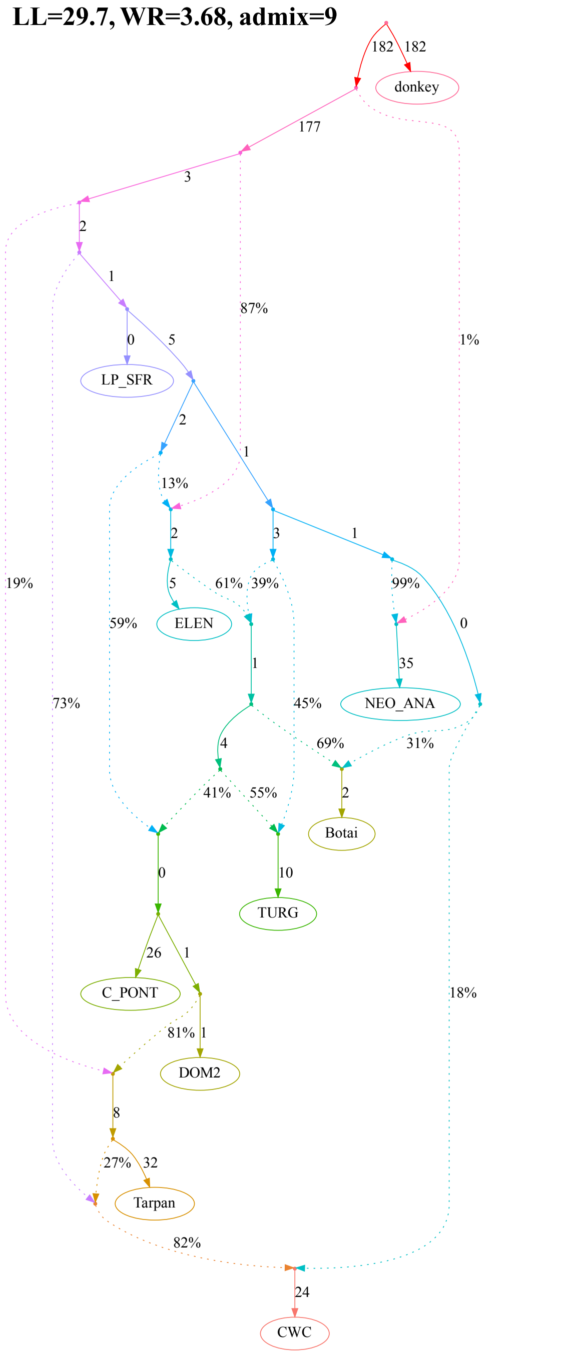

claim 1 no<br>claim 2 yes<br>claim 3 no<br>claim 4 yes<br>claim 5 no	claim 1 no<br>claim 2 yes<br>claim 3 no<br>claim 4 yes<br>claim 5 no
The modified set of individuals and the corrected algorithm for calculating f_3-statistics (1,767,419 variable site with no missing data at the group level)	The modified set of individuals and the original algorithm for calculating f_3-statistics (6,343,116 variable site with no missing data at the group level)
**LL=26.5, WR=3.38, admix=9**<br>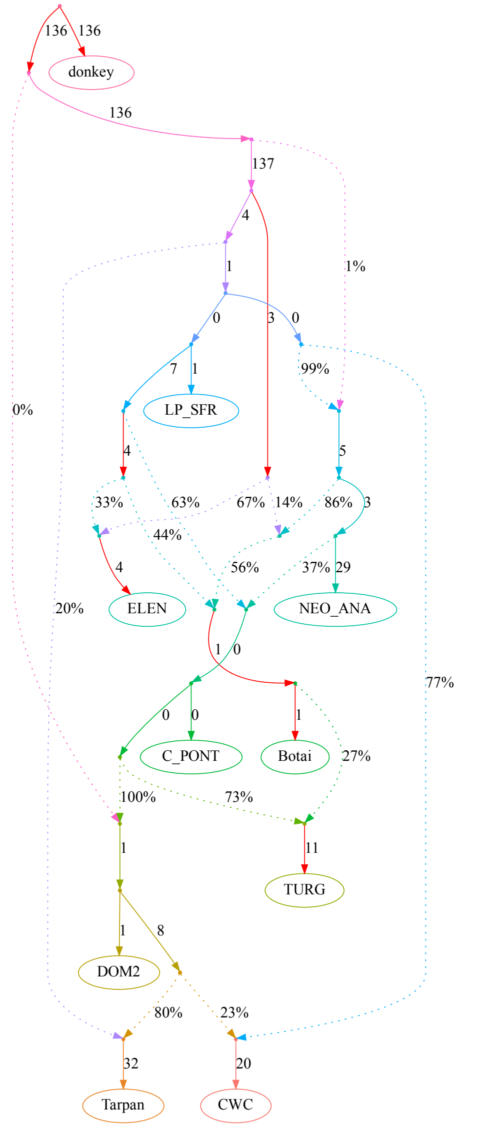 	**LL=56.8, WR=5.64, admix=9**<br>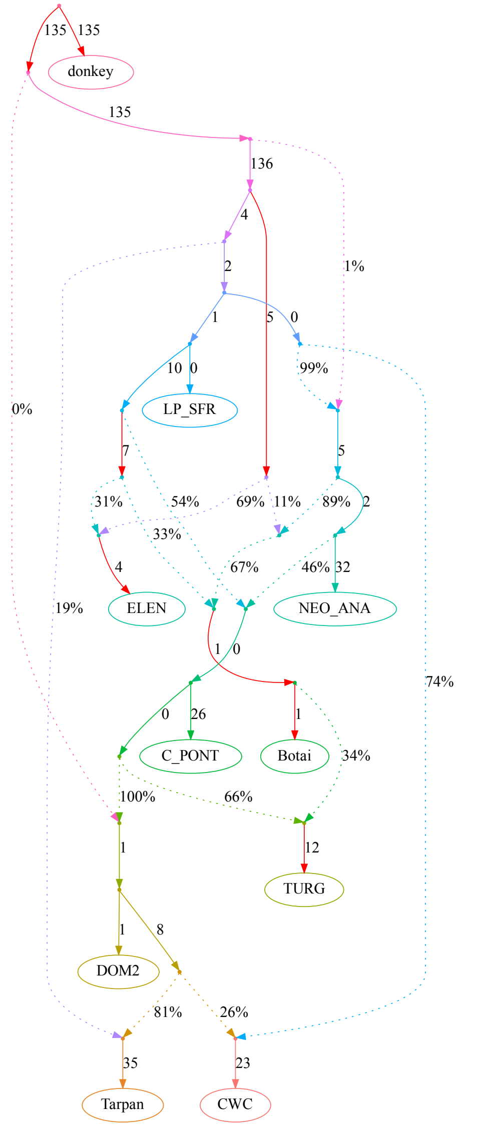

o, selected models with 9 admixture events (all plausible models with WR < 4 SE)

<table>
<tr>
<td>claim 1 yes
claim 2 yes
claim 3 no
claim 4 no
claim 5 yes</td>
<td>claim 1 yes
claim 2 yes
claim 3 no
claim 4 no
claim 5 yes</td>
</tr>
<tr>
<td>The modified set of individuals and the corrected algorithm for calculating f_3-statistics (1,767,419 variable site with no missing data at the group level)</td>
<td>The modified set of individuals and the original algorithm for calculating f_3-statistics (6,343,116 variable site with no missing data at the group level)</td>
</tr>
<tr>
<td>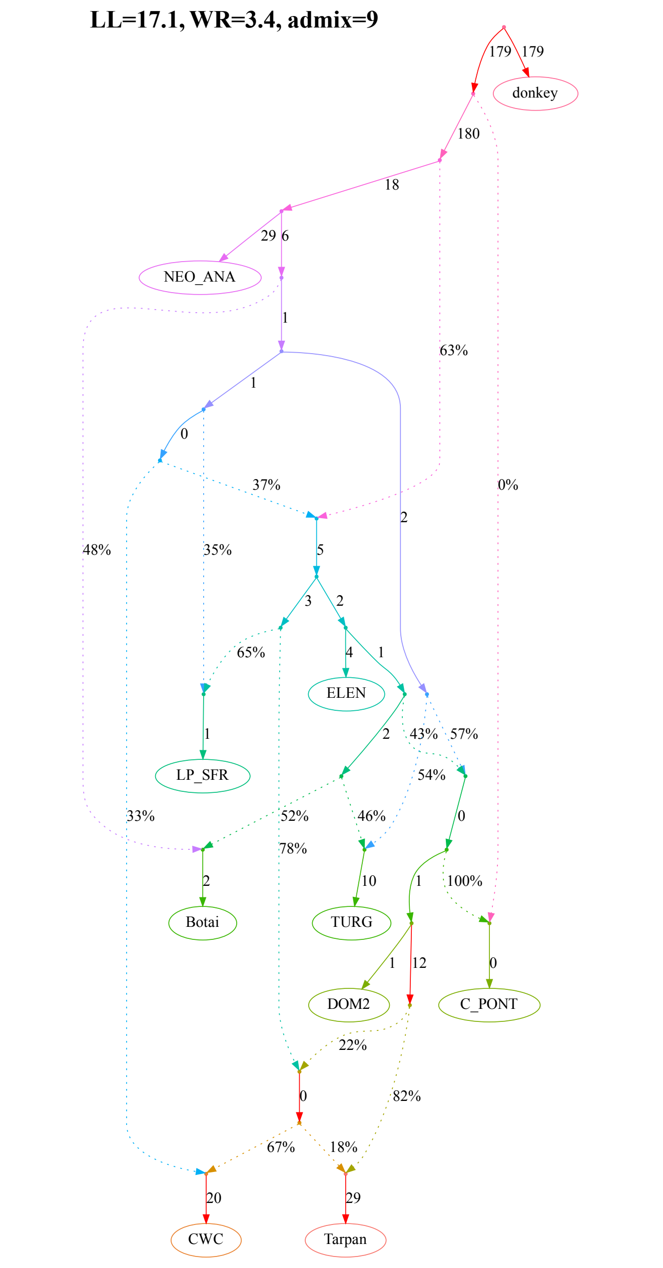
</td>
<td>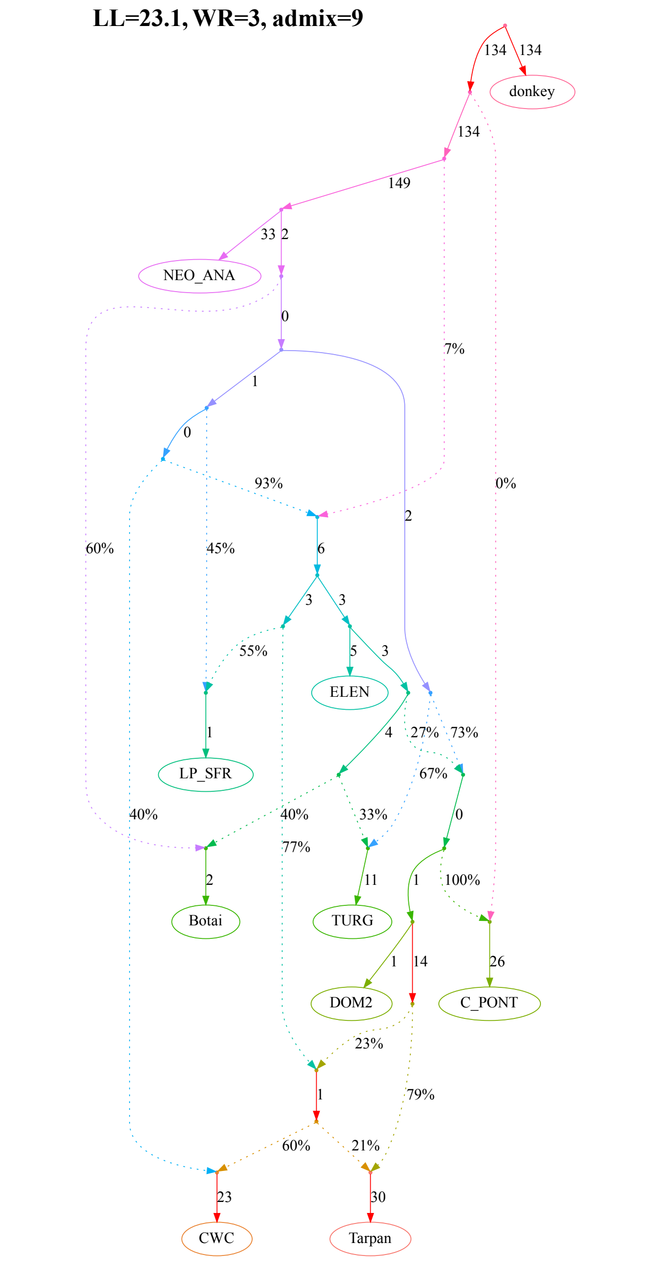
</td>
</tr>
</table>

claim 1 yes<br>claim 2 yes<br>claim 3 no<br>claim 4 yes<br>claim 5 no	claim 1 yes<br>claim 2 yes<br>claim 3 no<br>claim 4 yes<br>claim 5 no
The modified set of individuals and the corrected algorithm for calculating f_3-statistics (1,767,419 variable site with no missing data at the group level)	The modified set of individuals and the original algorithm for calculating f_3-statistics (6,343,116 variable site with no missing data at the group level)

LL=26.3, WR=3.66, admix=9

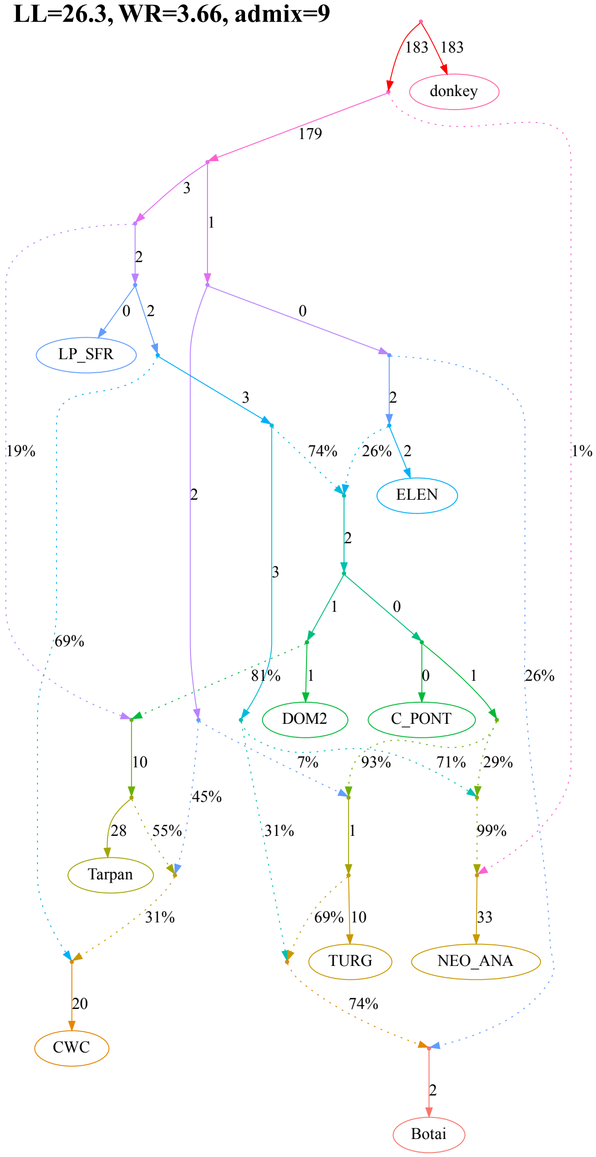

LL=33.8, WR=4.18, admix=9

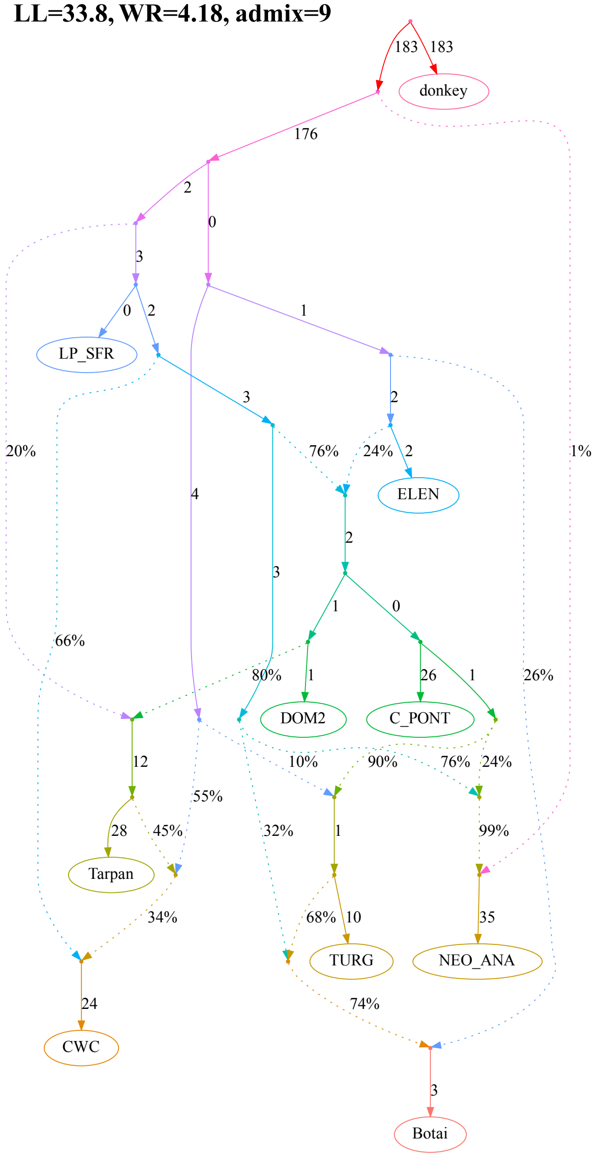

p, selected models with 9 admixture events (all plausible models with WR < 4 SE)

**claim 1** yes<br>**claim 2** yes<br>**claim 3** yes<br>**claim 4** yes<br>**claim 5** no	**claim 1** yes<br>**claim 2** yes<br>**claim 3** yes<br>**claim 4** yes<br>**claim 5** no
The modified set of individuals and the corrected algorithm for calculating *f*$_3$-statistics (1,767,419 variable site with no missing data at the group level)	The modified set of individuals and the original algorithm for calculating *f*$_3$-statistics (6,343,116 variable site with no missing data at the group level)
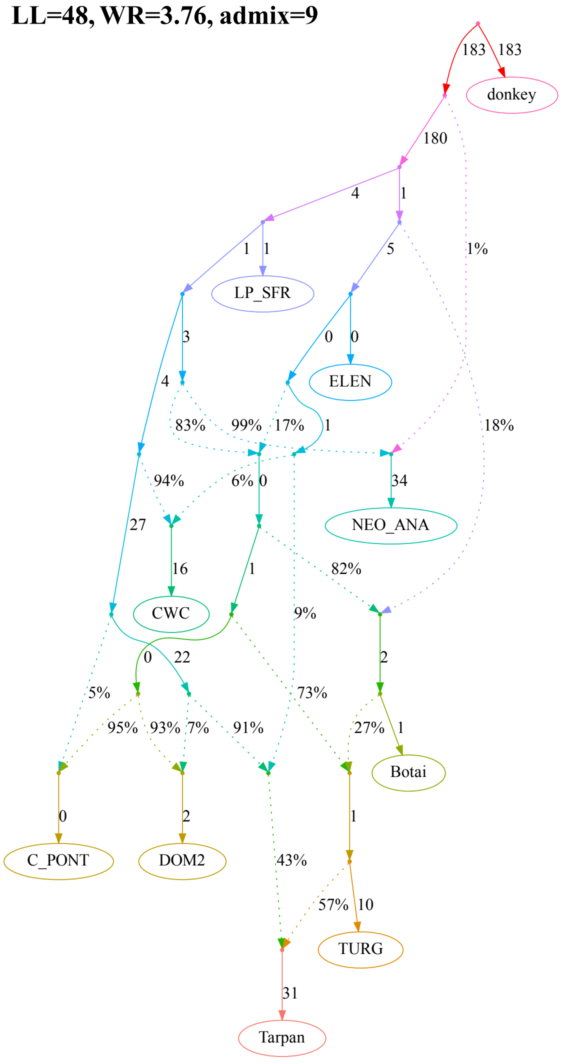	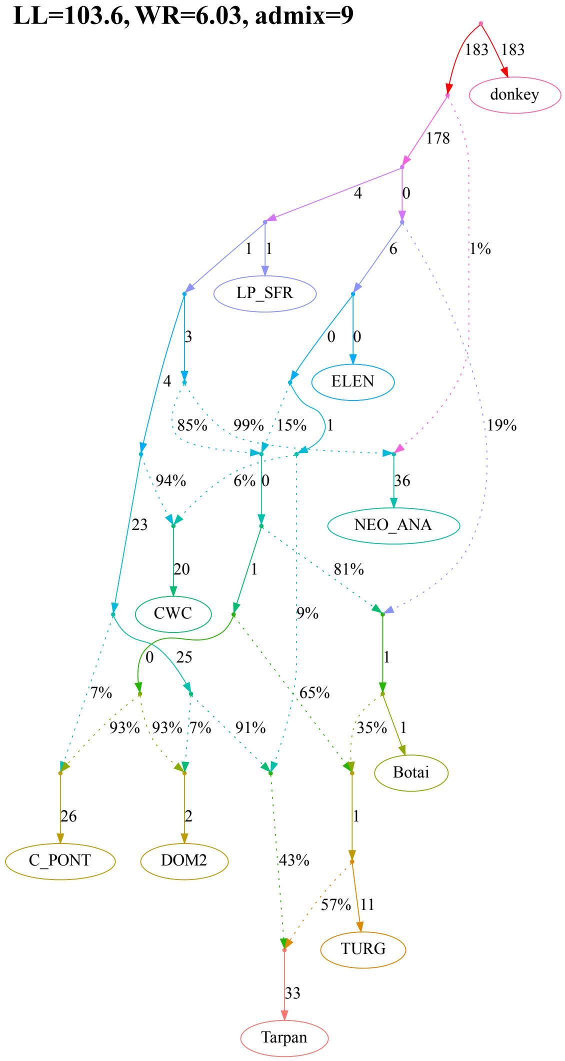

<table>
<tr><td>claim 1 yes
claim 2 yes
claim 3 no
claim 4 yes
claim 5 yes</td><td>claim 1 yes
claim 2 yes
claim 3 no
claim 4 yes
claim 5 yes</td></tr>
<tr><td>The modified set of individuals and the corrected algorithm for calculating f_3-statistics (1,767,419 variable site with no missing data at the group level)</td><td>The modified set of individuals and the original algorithm for calculating f_3-statistics (6,343,116 variable site with no missing data at the group level)</td></tr>
<tr><td>

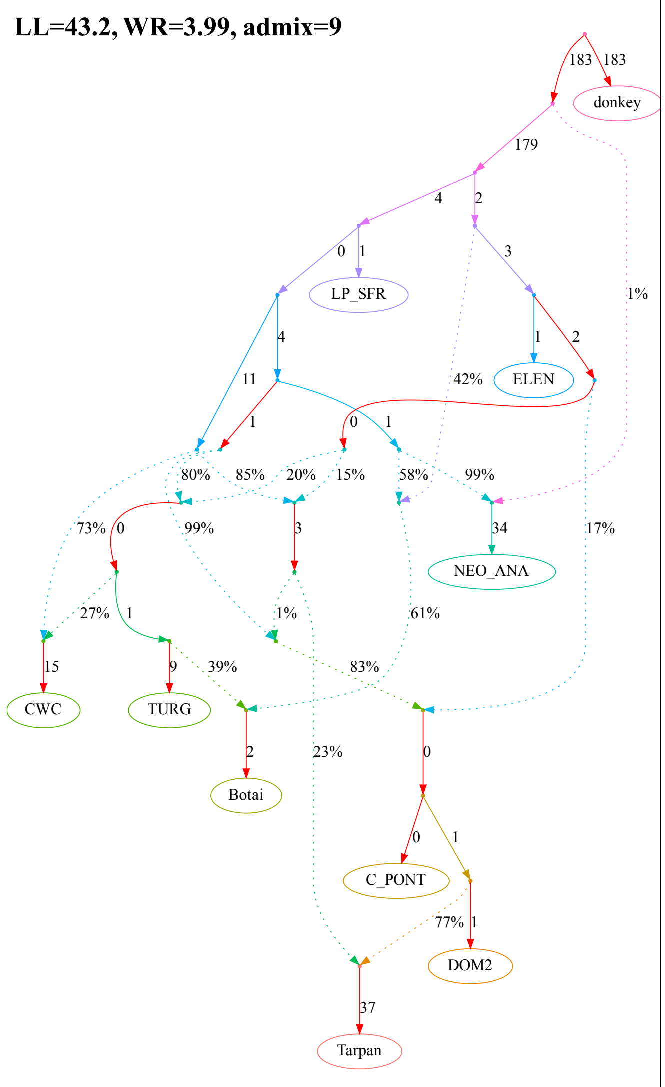

</td><td>

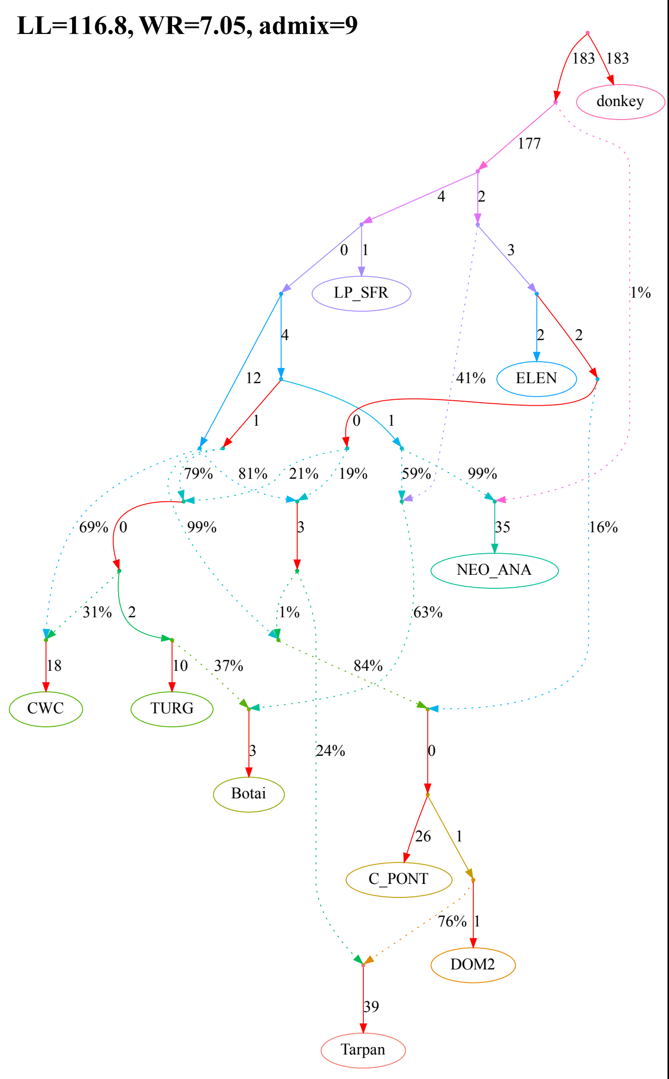

</td></tr>
</table>

q, selected models with 9 admixture events (all plausible models with WR < 4 SE)

**claim 1** yes<br>**claim 2** yes<br>**claim 3** no<br>**claim 4** yes<br>**claim 5** yes	**claim 1** yes<br>**claim 2** yes<br>**claim 3** no<br>**claim 4** yes<br>**claim 5** yes
The modified set of individuals and the corrected algorithm for calculating f_3-statistics (1,767,419 variable site with no missing data at the group level)	The modified set of individuals and the original algorithm for calculating f_3-statistics (6,343,116 variable site with no missing data at the group level)

LL=37.6, WR=3.76, admix=9

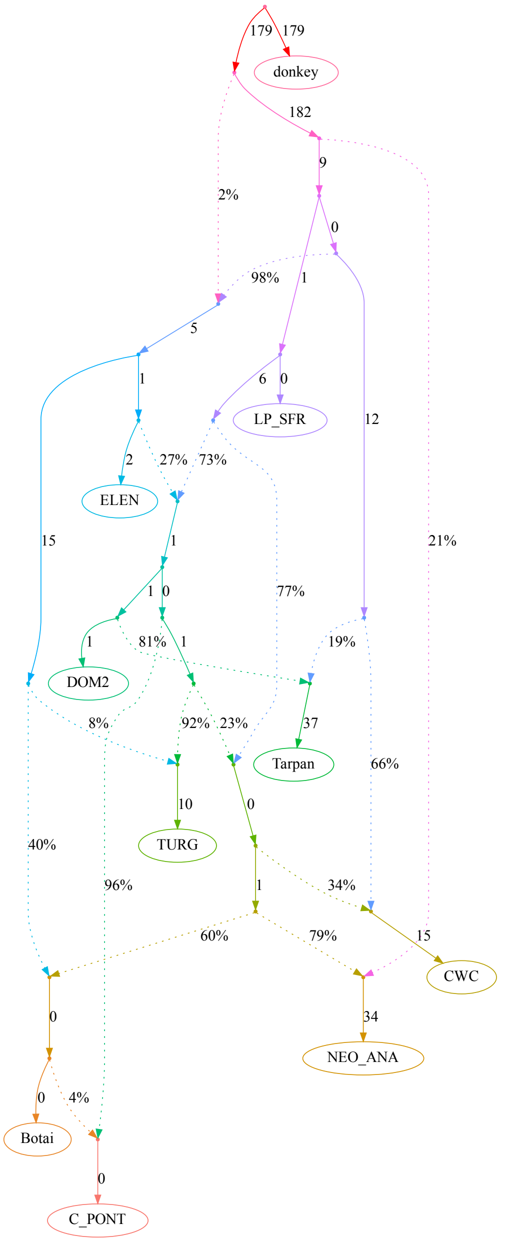

LL=57.1, WR=4.65, admix=9

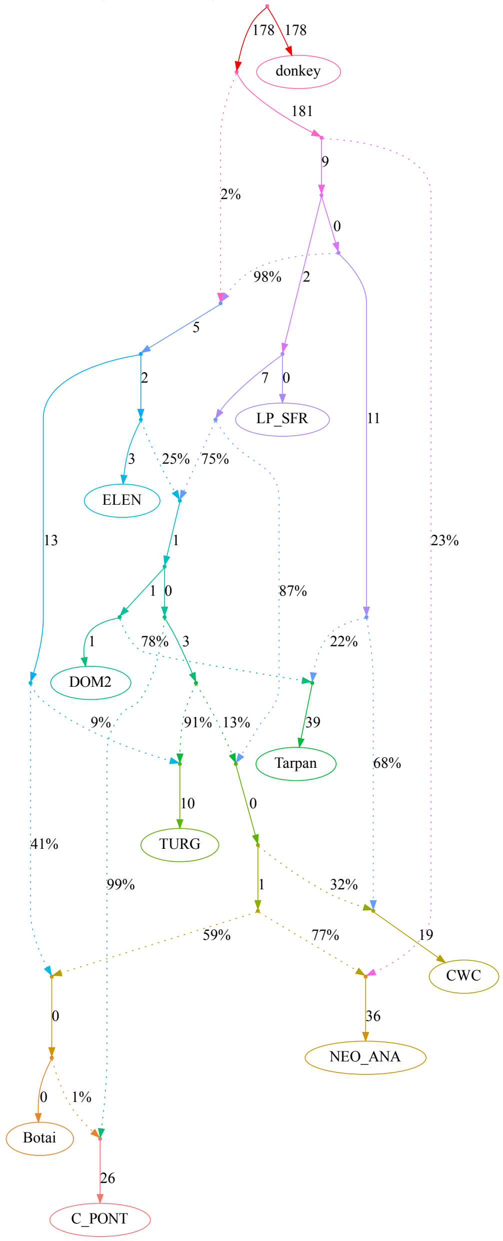

claim 1 yes<br>claim 2 yes<br>claim 3 no<br>claim 4 yes<br>claim 5 no	claim 1 yes<br>claim 2 yes<br>claim 3 no<br>claim 4 yes<br>claim 5 no
The modified set of individuals and the corrected algorithm for calculating f_3-statistics (1,767,419 variable site with no missing data at the group level)	The modified set of individuals and the original algorithm for calculating f_3-statistics (6,343,116 variable site with no missing data at the group level)

LL=31.3, WR=3.78, admix=9

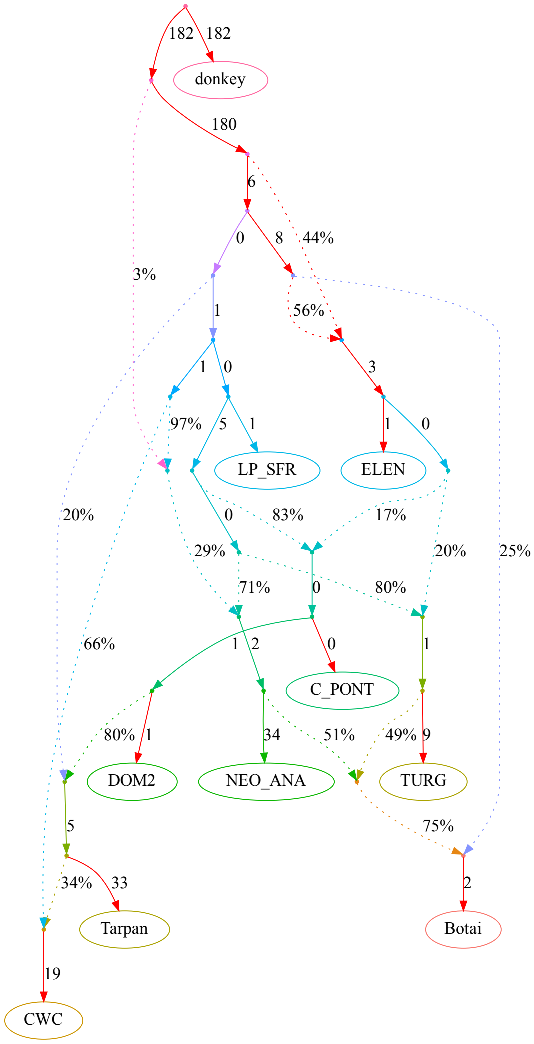

LL=66, WR=5.61, admix=9

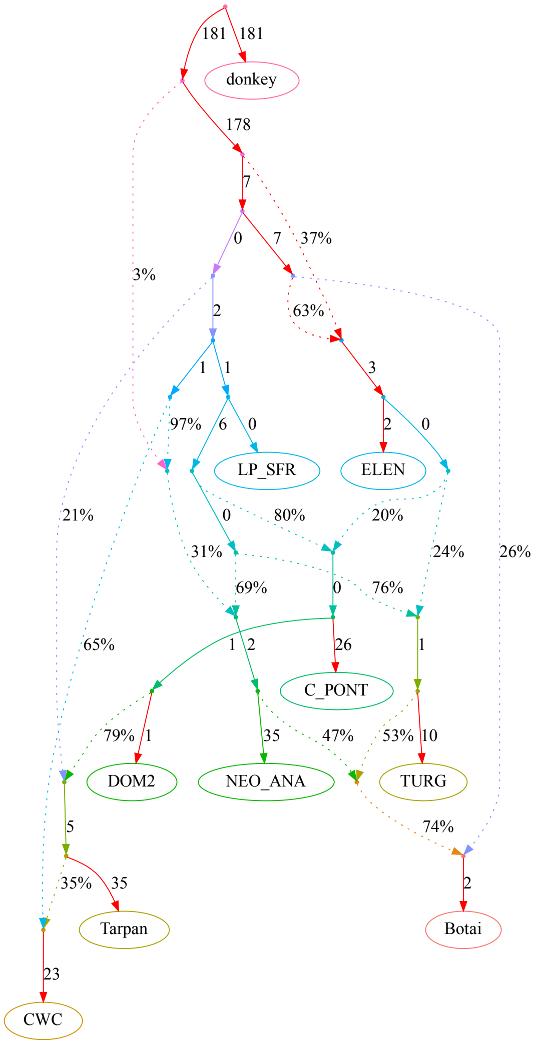

r, selected models with 9 admixture events (all plausible models with WR < 4 SE)

**claim 1** no<br>**claim 2** no<br>**claim 3** no<br>**claim 4** no<br>**claim 5** yes	**claim 1** no<br>**claim 2** no<br>**claim 3** no<br>**claim 4** no<br>**claim 5** yes
The modified set of individuals and the corrected algorithm for calculating f_3-statistics (1,767,419 variable site with no missing data at the group level)	The modified set of individuals and the original algorithm for calculating f_3-statistics (6,343,116 variable site with no missing data at the group level)

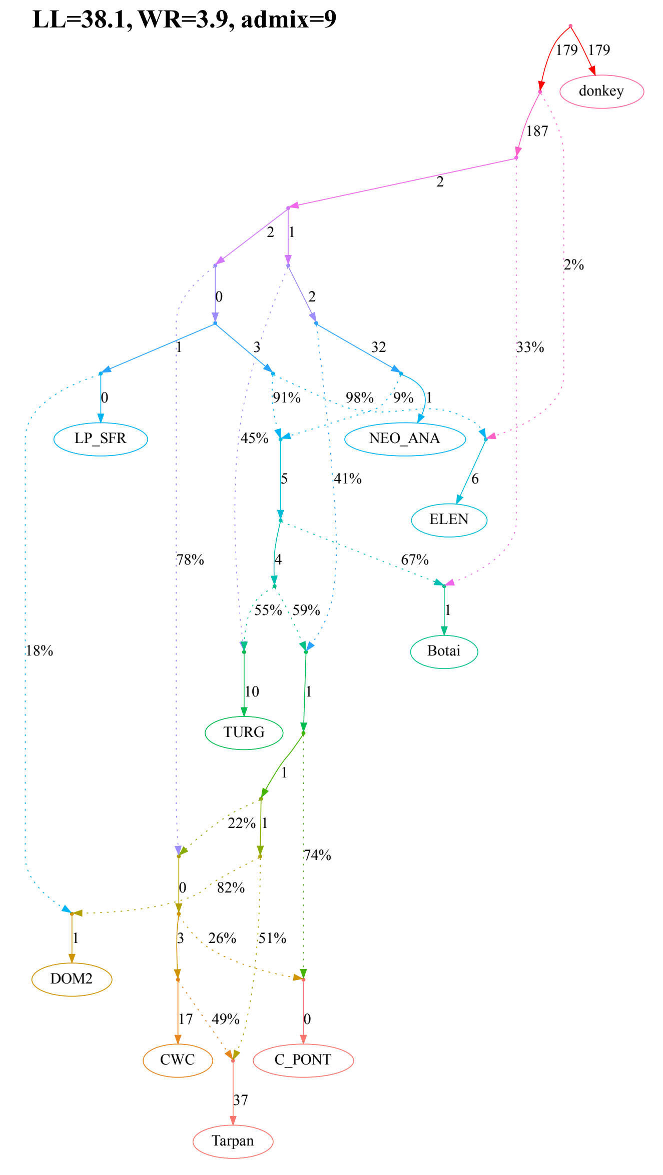

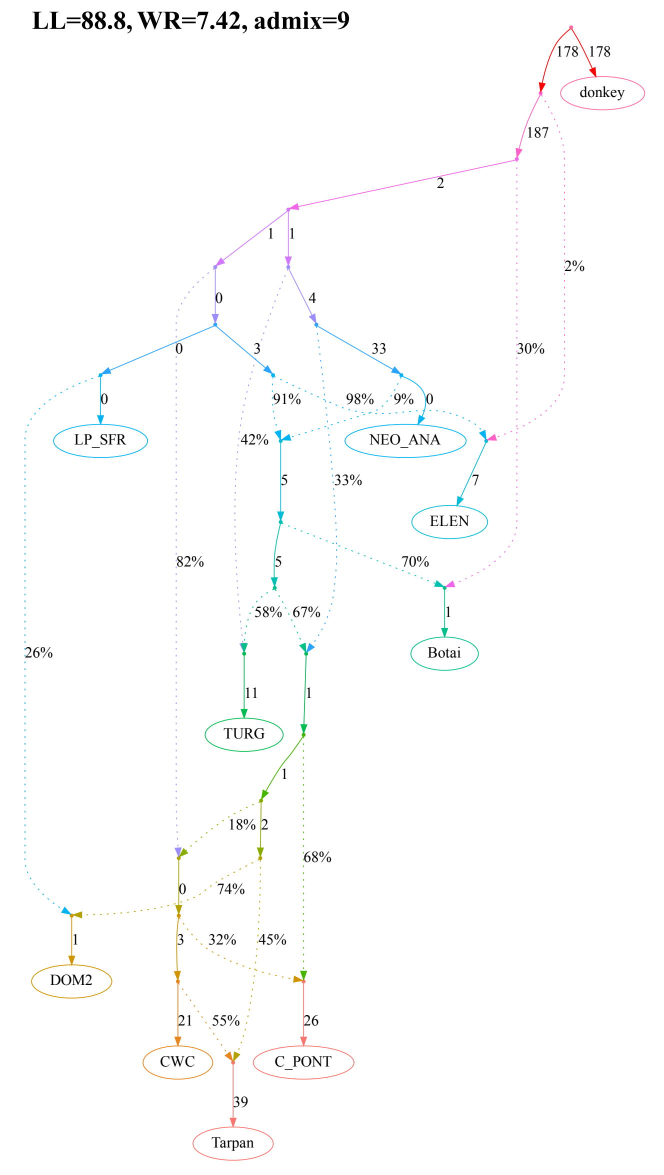

Supplement: Figure 3—source data 7. — The graphs were also re-fitted on the original set of single-nucleotide polymorphisms (SNPs)/individuals and using the original algorithm for calculating f-statistics. Selected alternative graphs found with findGraphs when more admixture events were allowed (from 6 to 9) are also shown. Model parameters (graph edges) that were inferred to be unidentifiable are plotted in red. (a) The published model, three admixture events. The following claims in Librado et al., 2021 rely on the admixture graph: (1) NEO-ANA-related admixture is absent in DOM2; (2) DOM2 and C-PONT are sister groups; (3) there is no gene flow connecting the CWC group and the cluster associated with Yamnaya horses and horses of the later Sintashta culture whose ancestry is maximized in the Western Steppe (DOM2, C-PONT, and TURG); (4) there was a gene flow from a deep-branching ghost group to NEO-ANA; (5) Tarpan is a mixture of a CWC-related and a DOM2-related lineage. (b) An alternative model with three admixture events fitting significantly better than the published one, (c) the published model, four admixture events, (d) an alternative model with four admixture events fitting not significantly worse than the published one, (e) the published model, five admixture events, (f) an alternative model with five admixture events fitting not significantly worse than the published one, (g) selected models with six admixture events, (h,i) selected models with seven admixture events (all plausible models with WR < 5 SE), (j-l) selected models with eight admixture events (all plausible models with WR < 4 SE), (m-r) selected models with nine admixture events (all plausible models with WR < 4 SE). [file elife-85492-fig3-data7.pdf]
