# Supplementary material for: On the limits of fitting complex models of population history to f-statistics
Source: eLife. 2023 Jun 29;12:e85492. doi: 10.7554/eLife.85492 (PMC10310323; doi:10.7554/eLife.85492)

Figure 3–source data 8. Published admixture graph from Hajdinjak *et al*. (2021) and alternative graphs found with *findGraphs* (12 populations, 8 admixture events). Model parameters (graph edges) that were inferred to be unidentifiable are plotted in red.

a, published model

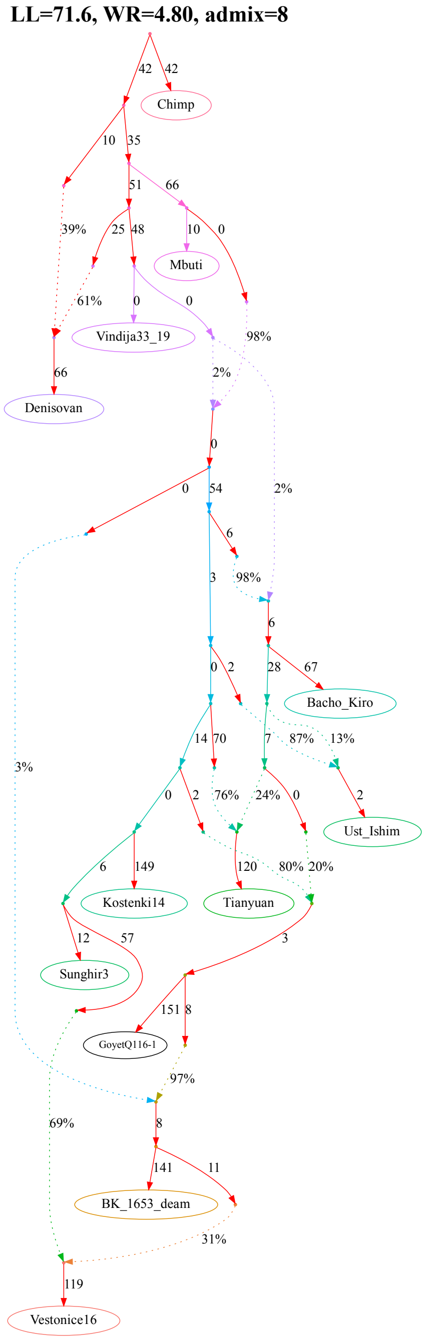

b, published model, simplified

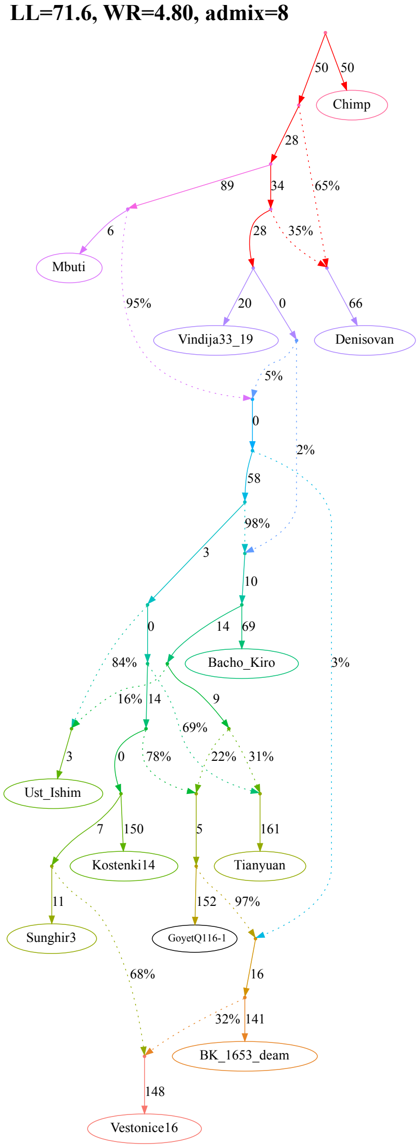

Claims by Hajdinjak *et al*. 2021 relying on the admixture graph:

1) Gene flow from the Bacho Kiro lineage to Ust'-Ishim, Tianyuan, and GoyetQ116-1;
2) the BK1653 individual belonged to a population that was related, but not identical, to that of the GoyetQ116-1 individual;
3) Vestonice is a mixture of a Sunghir-related and a BK1653-related lineage.

c, selected alternative models fitting significantly better than the published one

claim 1 partially supported, claims 2&3 supported

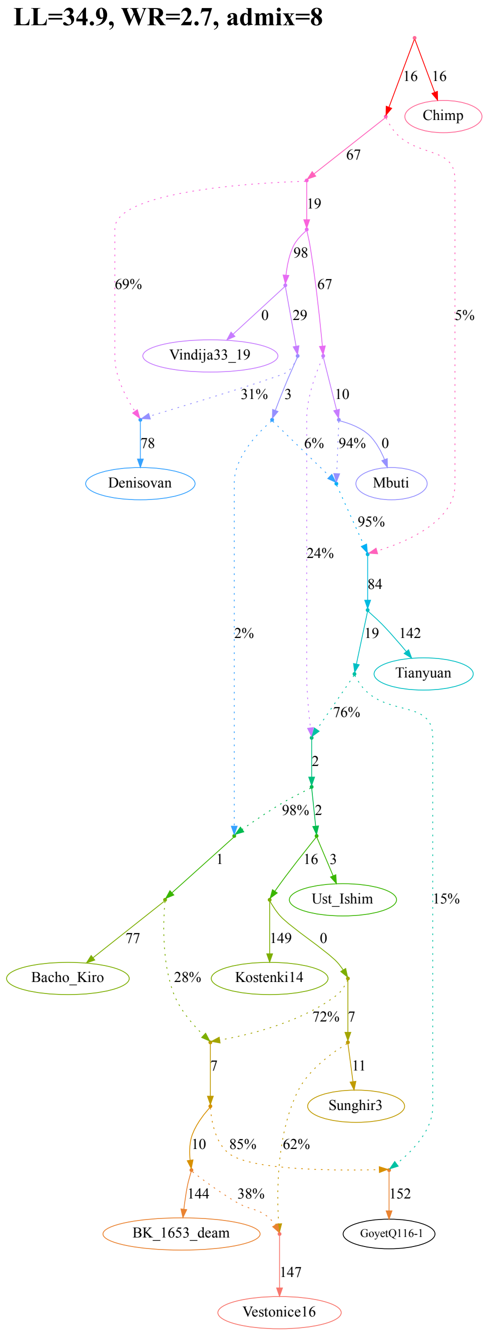

claim 1 not supported, claims 2&3 supported

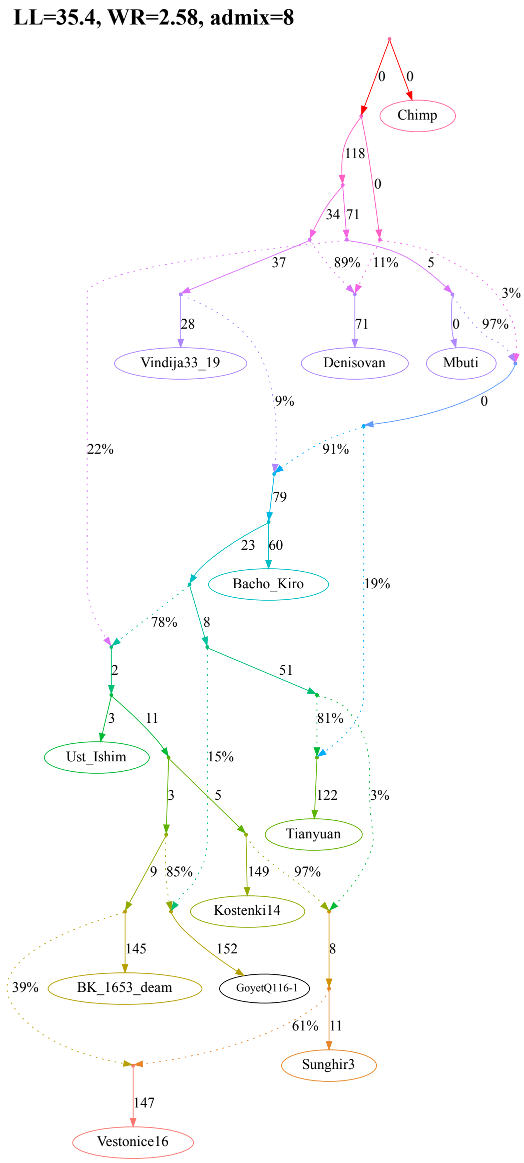

claim 1 not supported, claims 2&3 supported

LL=35.4, WR=2.7, admix=8

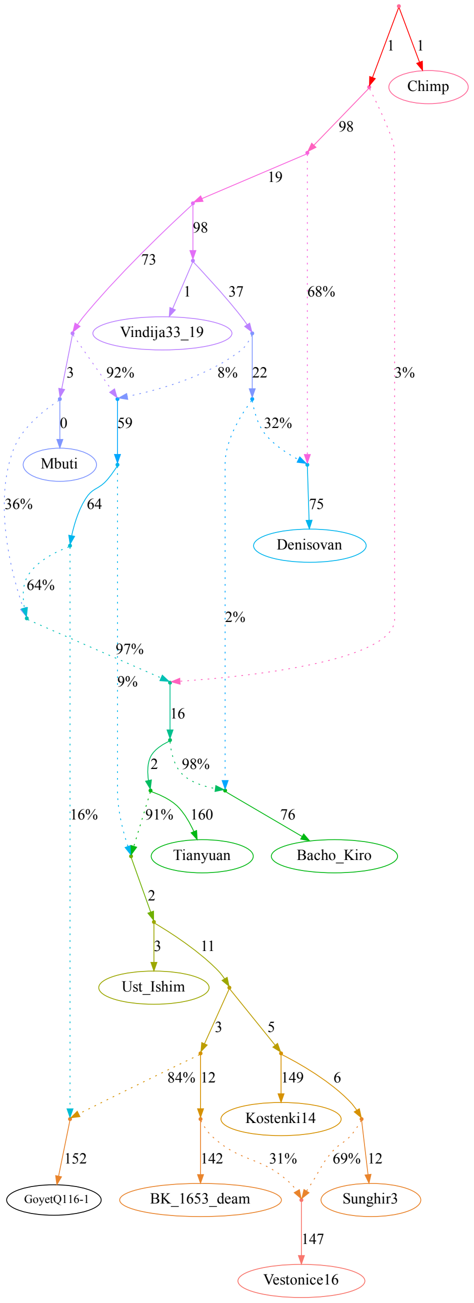

claim 1 not supported, claims 2&3 supported

LL=35.6, WR=2.7, admix=8

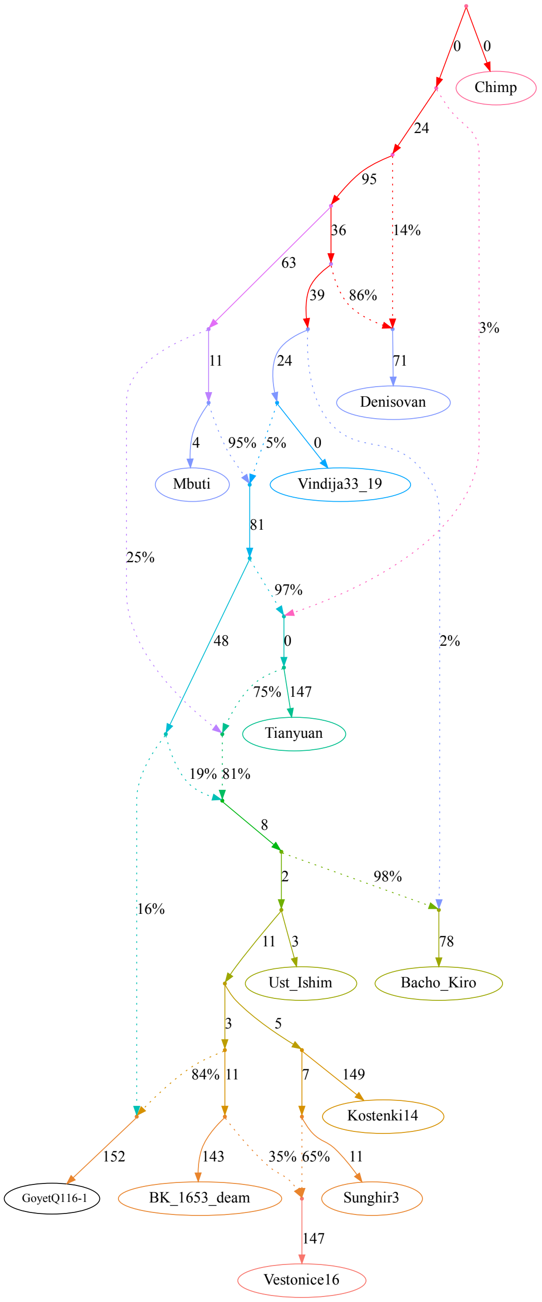

d, selected alternative models fitting significantly better than the published one

claim 1 not supported, claims 2&3 supported

LL=35.8, WR=2.7, admix=8

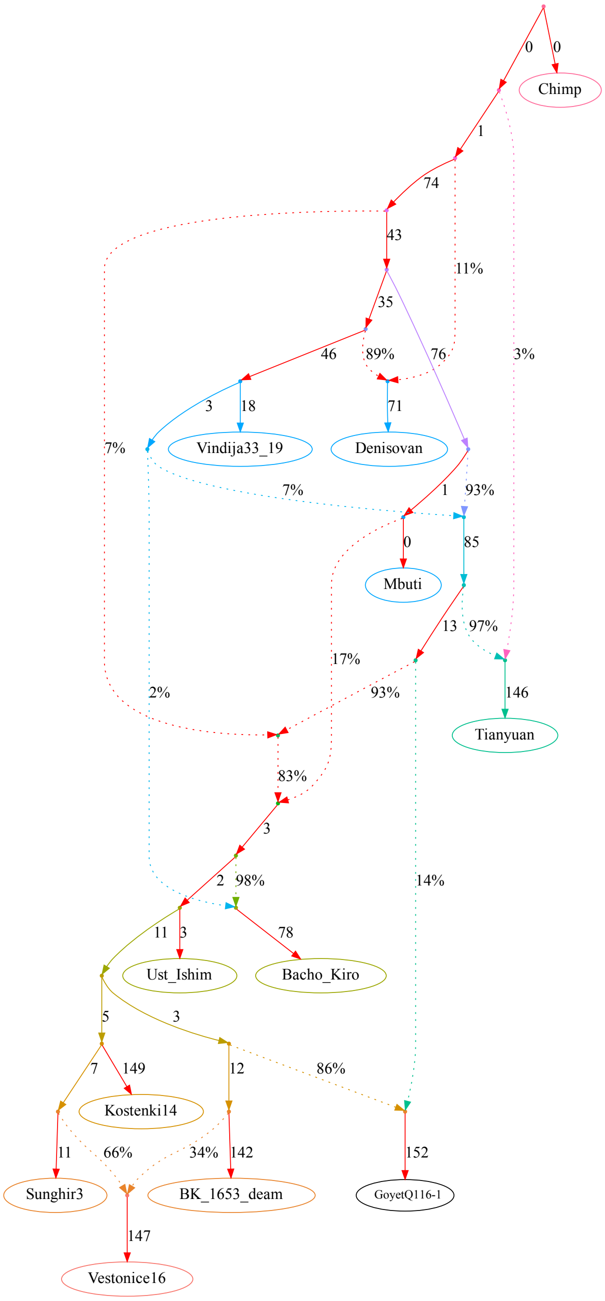

claim 1 not supported, claims 2&3 supported

LL=35.9, WR=2.75, admix=8

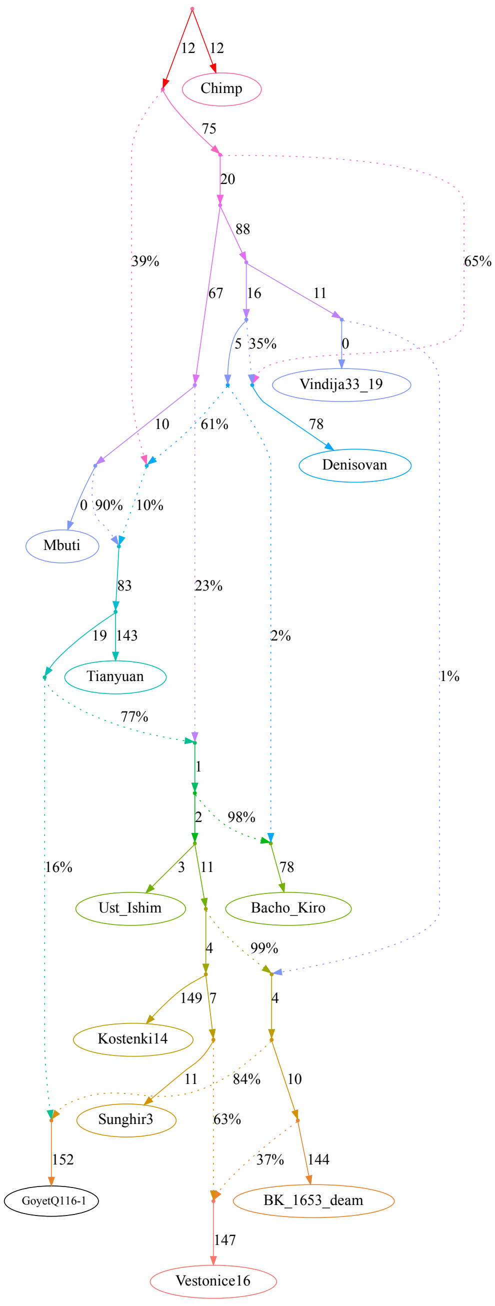

claims 1&2 not supported, claim 3 supported

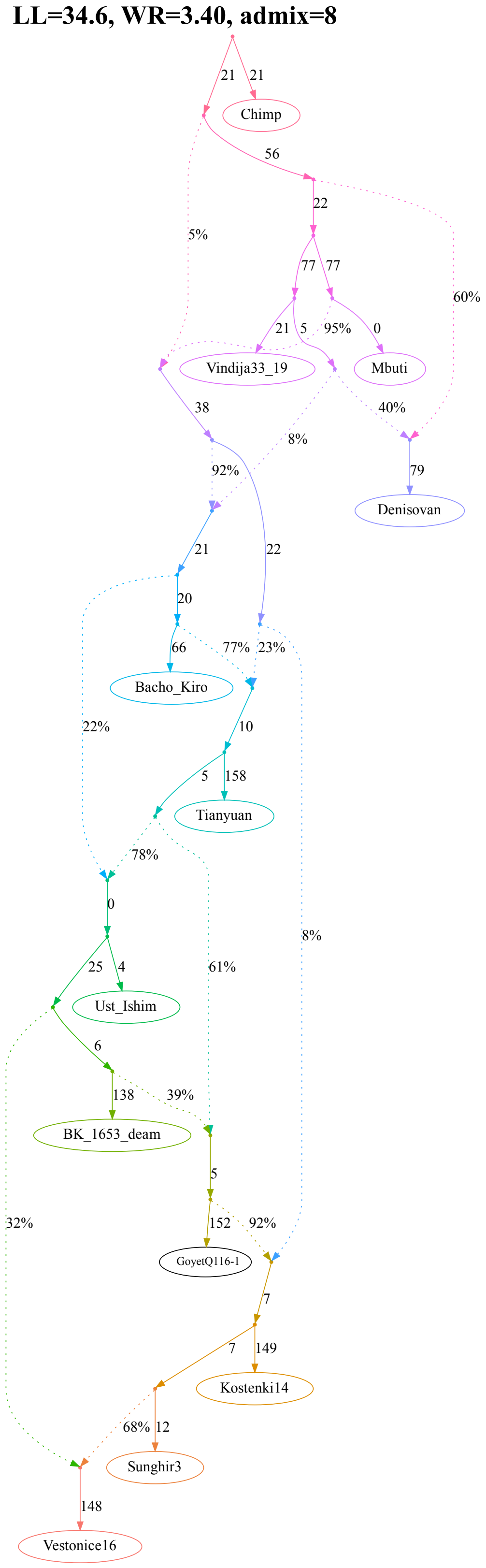

claims 1&2 not supported, claim 3 supported

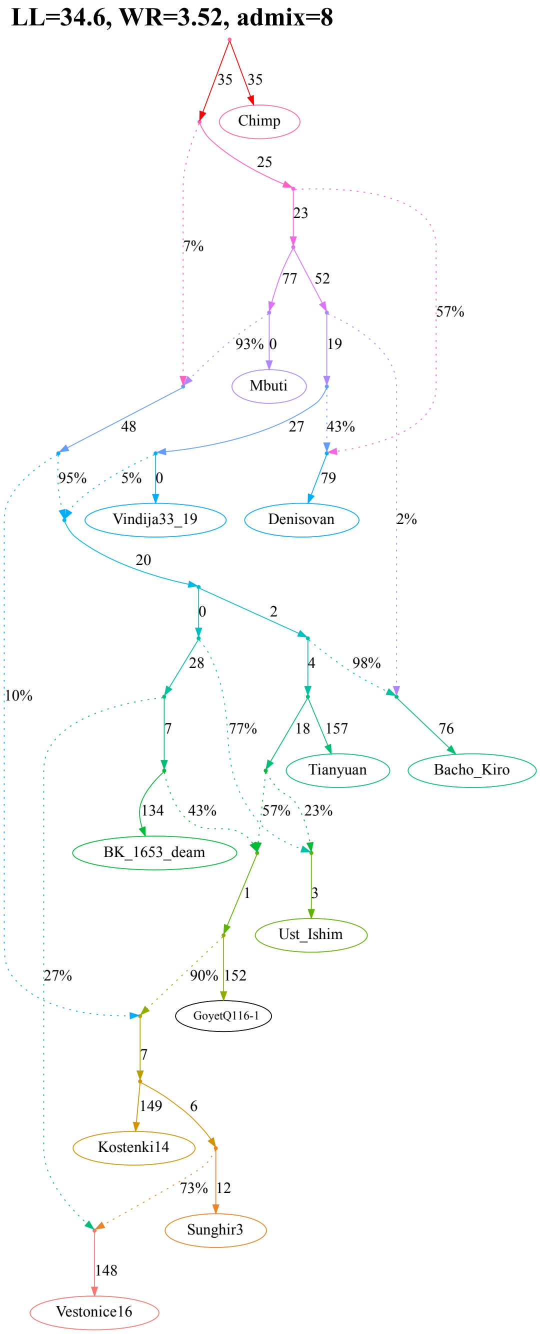

e, selected alternative models fitting significantly better than the published one

claims 1&2 not supported, claim 3 supported

LL=34.7, WR=3.52, admix=8

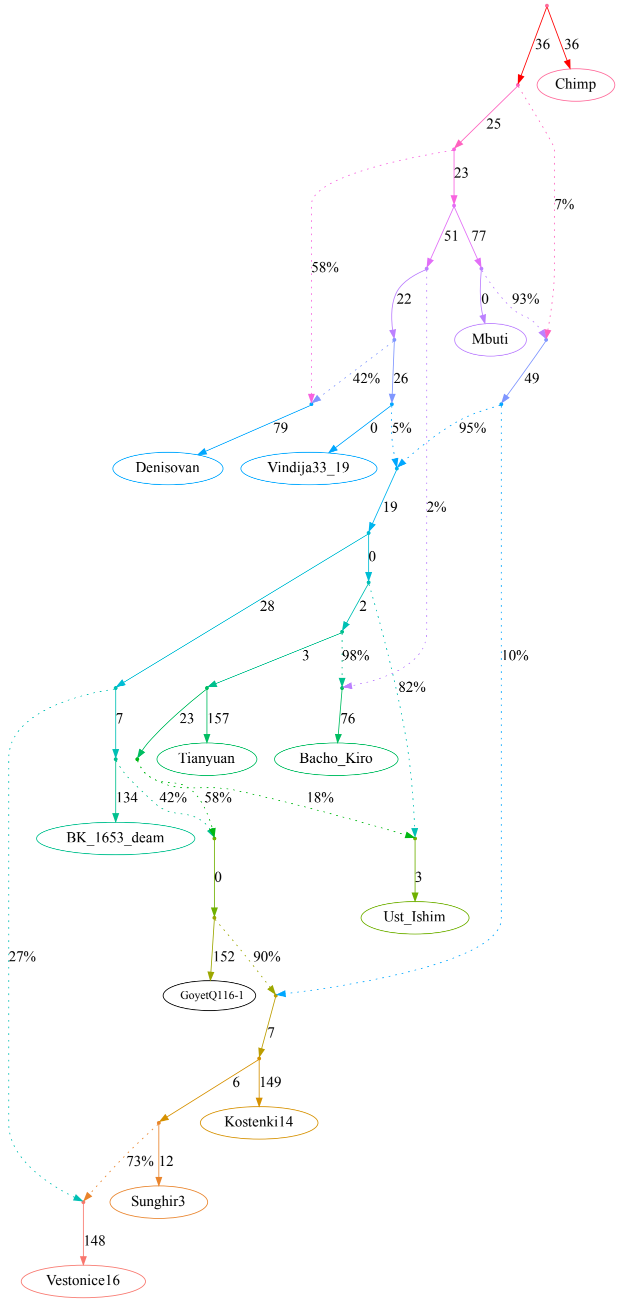

claims 1&2 not supported, claim 3 supported

LL=35.7, WR=3.52, admix=8

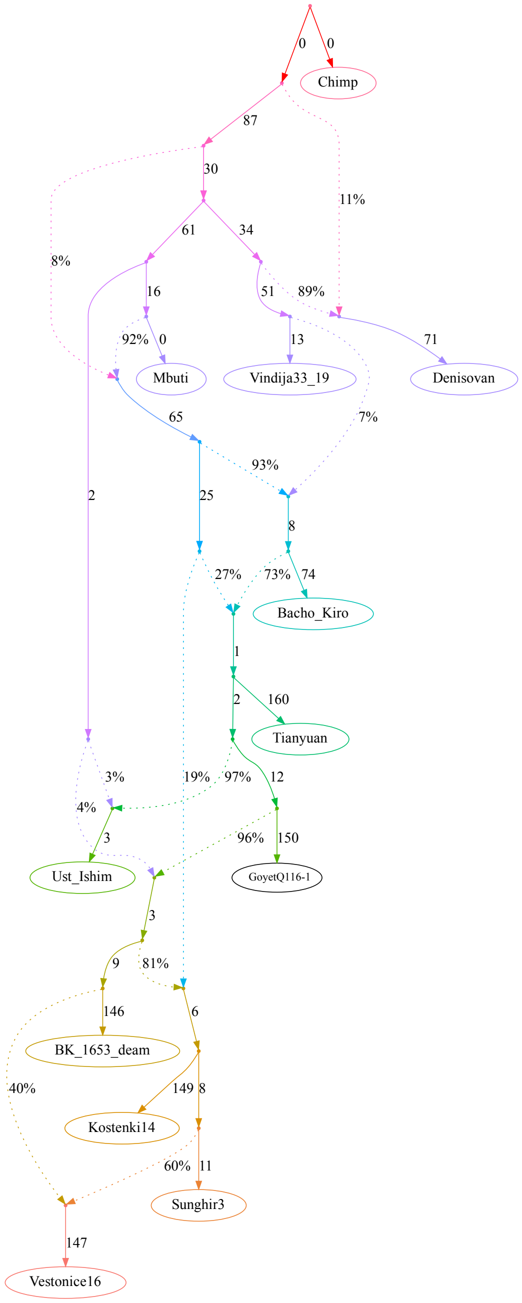

claims 1&2 not supported, claim 3 supported

LL=36.2, WR=3.52, admix=8

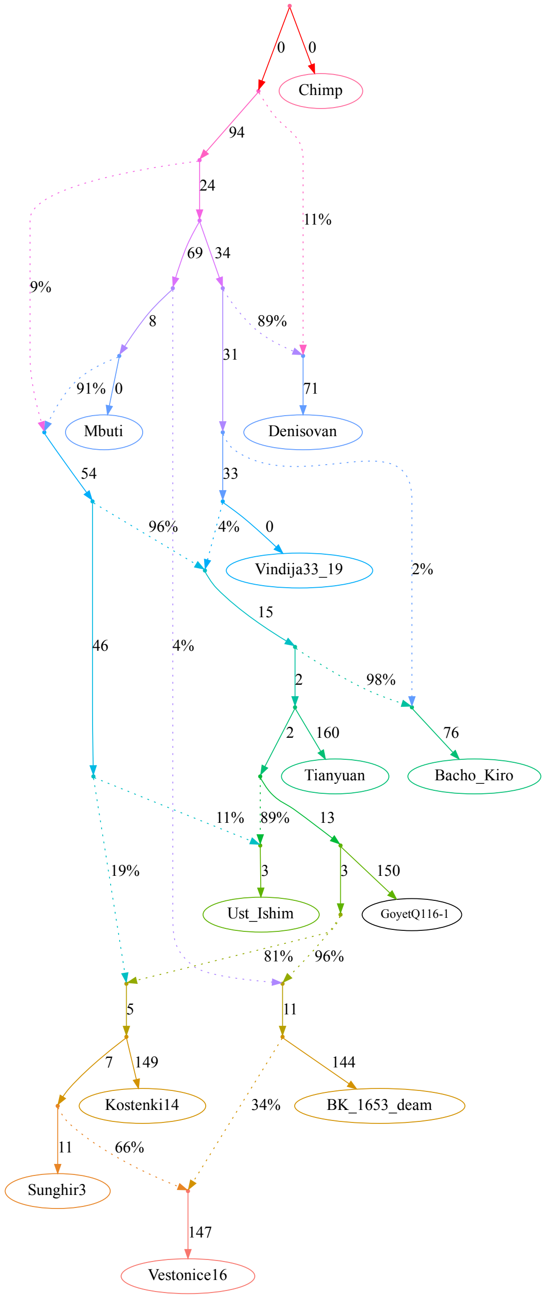

Supplement: Figure 3—source data 8. — Model parameters (graph edges) that were inferred to be unidentifiable are plotted in red. (a, b) The published model and its simplified representation. The following claims in Hajdinjak et al., 2021 rely on the admixture graph: (1) gene flow from the Bacho Kiro lineage to Ust’-Ishim, Tianyuan, and GoyetQ116-1; (2) the BK1653 individual belonged to a population that was related, but not identical, to that of the GoyetQ116-1 individual; (3) Vestonice is a mixture of a Sunghir-related and a BK1653-related lineage. (c-e) Selected alternative models fitting significantly better than the published one. [file elife-85492-fig3-data8.pdf]
