# Supplementary material for: On the limits of fitting complex models of population history to f-statistics
Source: eLife. 2023 Jun 29;12:e85492. doi: 10.7554/eLife.85492 (PMC10310323; doi:10.7554/eLife.85492)

Figure 4–source data 1. Published admixture graph from Lipson *et al*. (2020) and alternative graphs found with *findGraphs* (12 populations, 11 admixture events) using the updated algorithm for calculating *f*-statistics. The graphs were also re-fitted using the original algorithm for calculating *f*-statistics. Model parameters (graph edges) that were inferred to be unidentifiable are plotted in red.

a, published model

no. of East Asian groups with Onge-related ancestry	6	no. of East Asian groups with Onge-related ancestry	6
no. of East Asian groups with European-related ancestry	1	no. of East Asian groups with European-related ancestry	1
direction of gene flow China_Island_EN > Taiwan_IA	forward	direction of gene flow China_Island_EN > Taiwan_IA	forward
Mongolia_East is basal with respect to China_Upper_YR and Nepal_Chokhopani	yes	Mongolia_East is basal with respect to China_Upper_YR and Nepal_Chokhopani	yes
Taiwan_IA is basal with respect to Mongolia_East and Nepal_Chokhopani	yes	Taiwan_IA is basal with respect to Mongolia_East and Nepal_Chokhopani	yes
The original set of SNPs and individuals and the corrected algorithm for calculating f_3-statistics (211,738 variable site with no missing data at the group level)		The original set of SNPs and individuals and the original algorithm for calculating f_3-statistics (363,131 variable site with no missing data at the group level)	

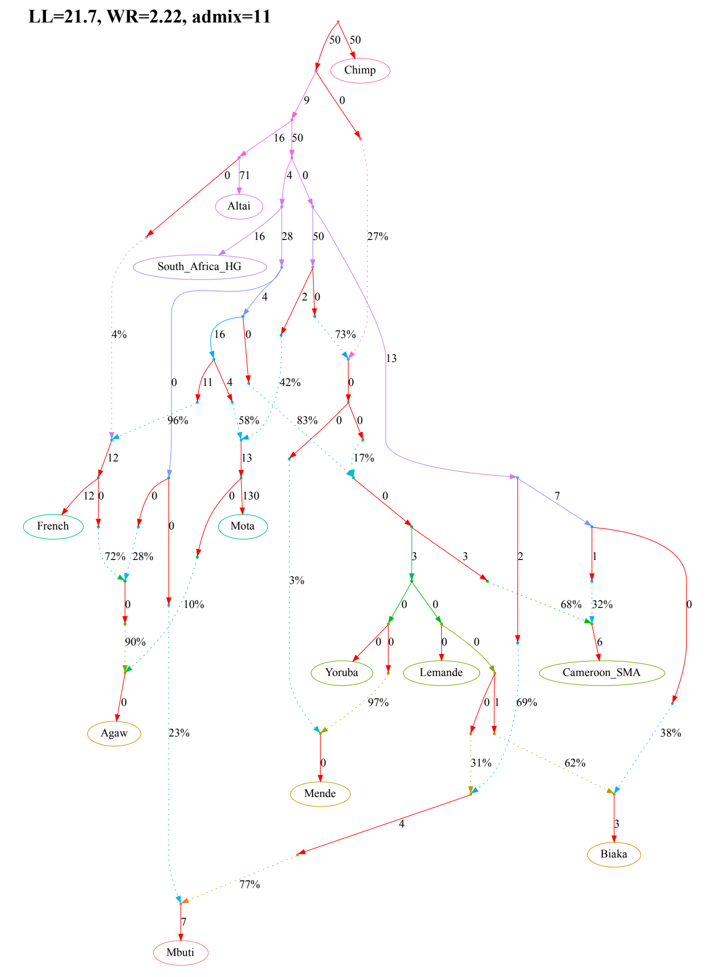

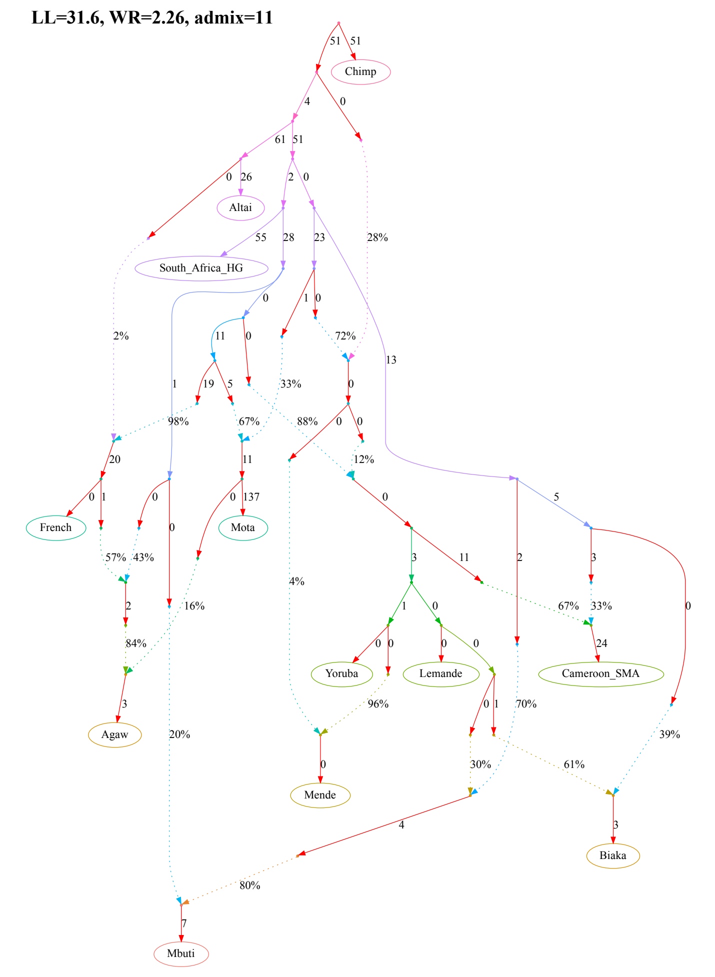

b, published model, simplified

no. of East Asian groups with Onge-related ancestry	6
no. of East Asian groups with European-related ancestry	1
direction of gene flow China_Island_EN > Taiwan_IA	forward
Mongolia_East is basal with respect to China_Upper_YR and Nepal_Chokhopani	yes
Taiwan_IA is basal with respect to Mongolia_East and Nepal_Chokhopani	yes

The original set of SNPs and individuals and the corrected algorithm for calculating f_3-statistics (211,738 variable site with no missing data at the group level)

LL=21.8, WR=2.27, admix=11

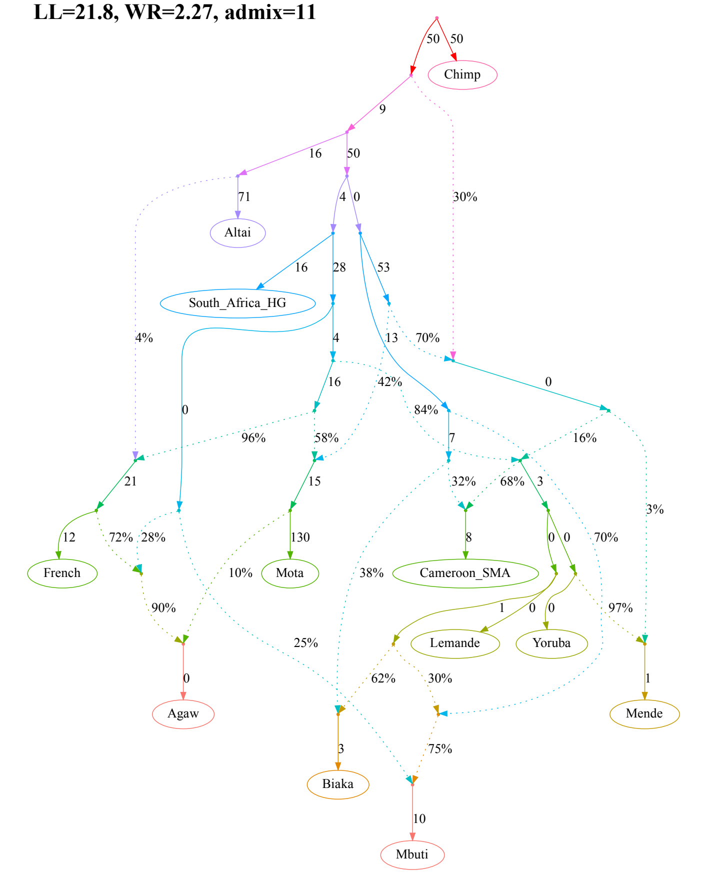

no. of East Asian groups with Onge-related ancestry	6
no. of East Asian groups with European-related ancestry	1
direction of gene flow China_Island_EN > Taiwan_IA	forward
Mongolia_East is basal with respect to China_Upper_YR and Nepal_Chokhopani	yes
Taiwan_IA is basal with respect to Mongolia_East and Nepal_Chokhopani	yes

The original set of SNPs and individuals and the original algorithm for calculating f_3-statistics (363,131 variable site with no missing data at the group level)

LL=32.2, WR=2.27, admix=11

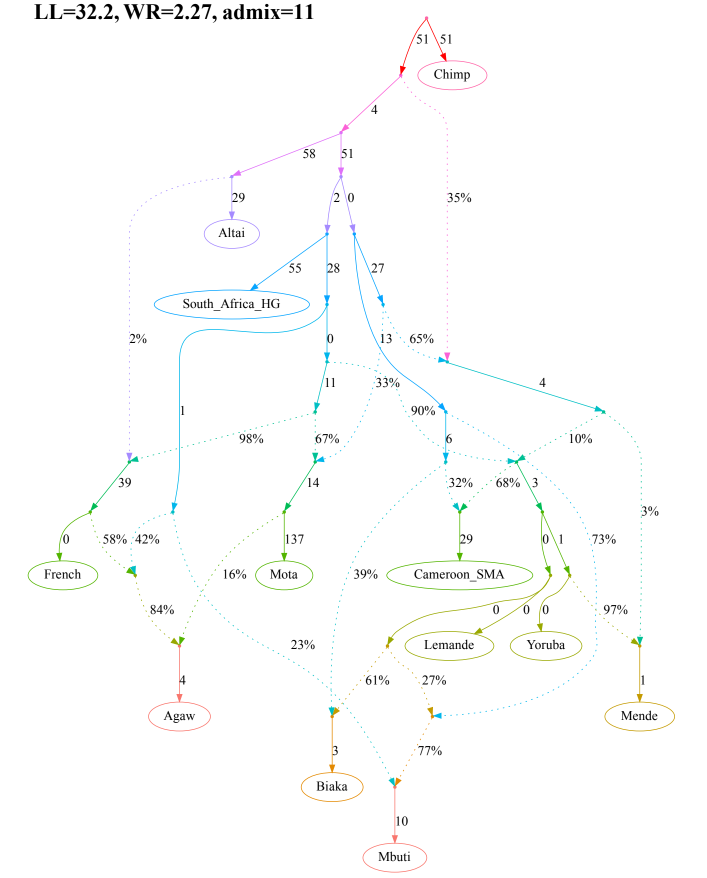

Selected claims by Lipson *et al*. 2020 relying on the admixture graph:

1) A lineage maximized in present-day West African groups (Lemande, Mende, and Yoruba) also contributed some ancestry to the ancient Shum Laka individual, and present-day Biaka and Mbuti;
2) another ancestry component in Shum Laka is a deep-branching lineage maximized in rainforest hunter-gatherers Biaka and Mbuti;
3) “super-archaic” ancestry (i.e., diverging at the modern human/Neanderthal split point or deeper) contributed to Biaka, Shum Laka, Mbuti, Lemande, Mende, and Yoruba;
4) a ghost modern human lineage (or lineages) contributed to Agaw, Mota, Biaka, Shum Laka, Mbuti, Lemande, Mende, and Yoruba.

c, selected alternative models fitting nominally better than the published one

**support for claim 1** partial<br>**support for claim 2** not supported<br>**support for claim 3** not supported<br>**support for claim 4** not supported	**support for claim 1** partial<br>**support for claim 2** not supported<br>**support for claim 3** not supported<br>**support for claim 4** not supported
The original set of SNPs and individuals and the corrected algorithm for calculating f_3-statistics (211,738 variable site with no missing data at the group level)	The original set of SNPs and individuals and the original algorithm for calculating f_3-statistics (363,131 variable site with no missing data at the group level)
**LL=13.1, WR=1.57, admix=11**<br>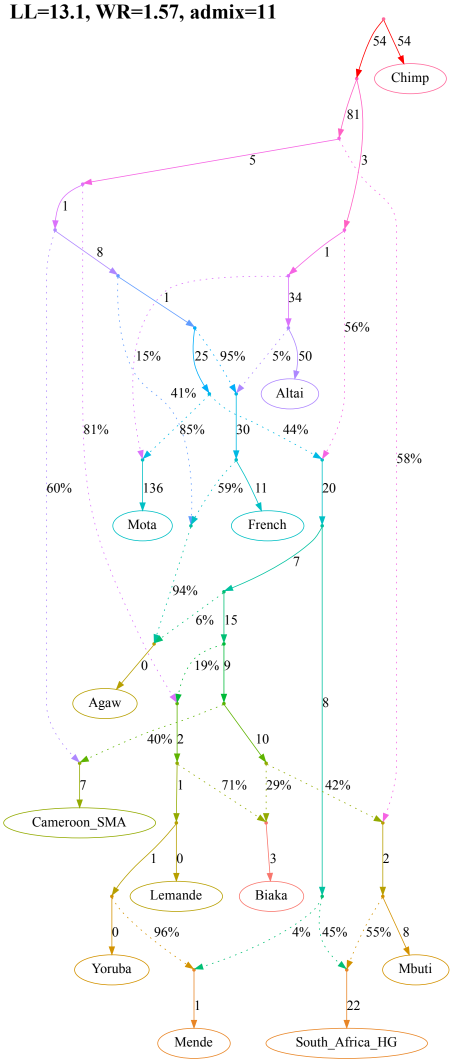 	**LL=20.9, WR=2.05, admix=11**<br>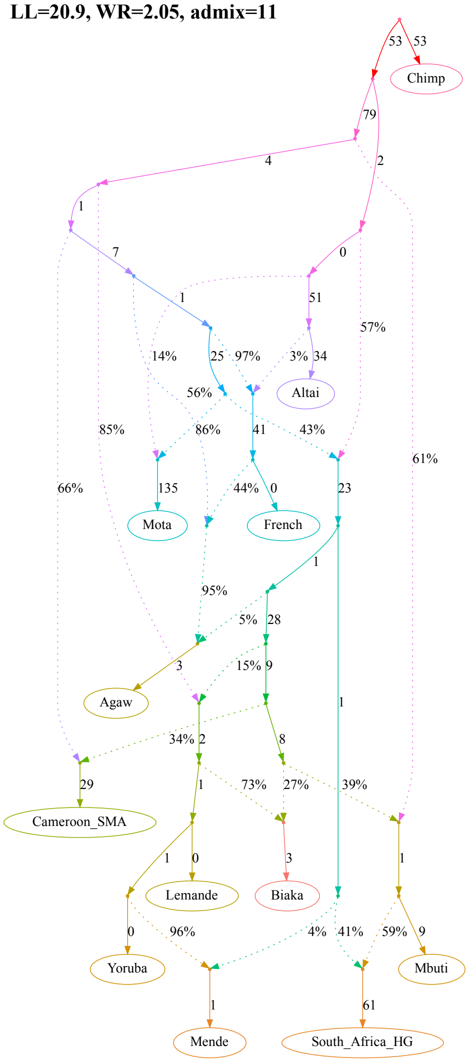

**support for claim 1** not supported<br>**support for claim 2** not supported<br>**support for claim 3** partial<br>**support for claim 4** not supported	**support for claim 1** not supported<br>**support for claim 2** not supported<br>**support for claim 3** partial<br>**support for claim 4** not supported
The original set of SNPs and individuals and the corrected algorithm for calculating f_3-statistics (211,738 variable site with no missing data at the group level)	The original set of SNPs and individuals and the original algorithm for calculating f_3-statistics (363,131 variable site with no missing data at the group level)

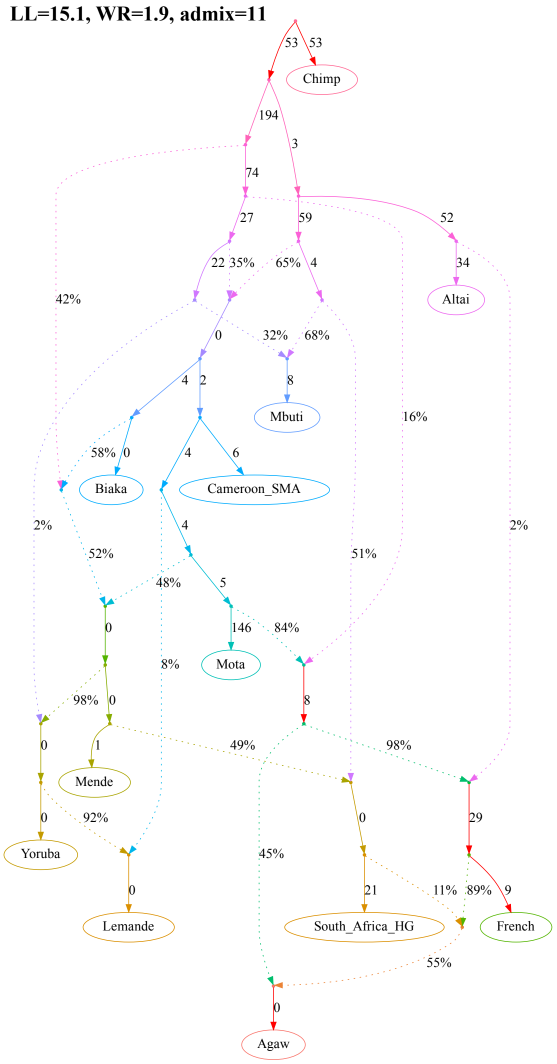

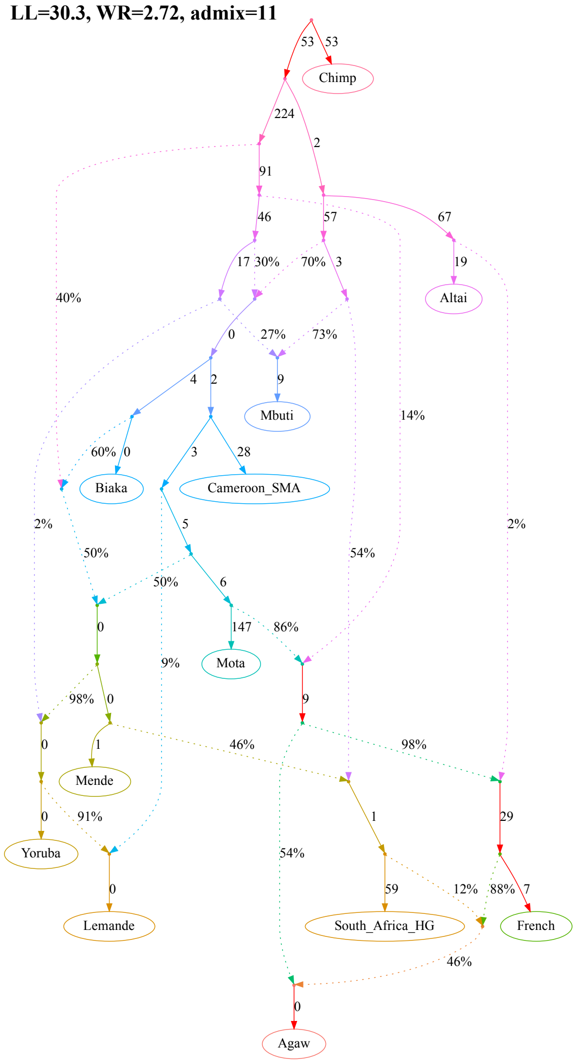

d, selected alternative models fitting nominally better than the published one

**support for claim 1** not supported<br>**support for claim 2** partial<br>**support for claim 3** partial<br>**support for claim 4** not supported	**support for claim 1** not supported<br>**support for claim 2** partial<br>**support for claim 3** partial<br>**support for claim 4** not supported
The original set of SNPs and individuals and the corrected algorithm for calculating f_3-statistics (211,738 variable site with no missing data at the group level)	The original set of SNPs and individuals and the original algorithm for calculating f_3-statistics (363,131 variable site with no missing data at the group level)
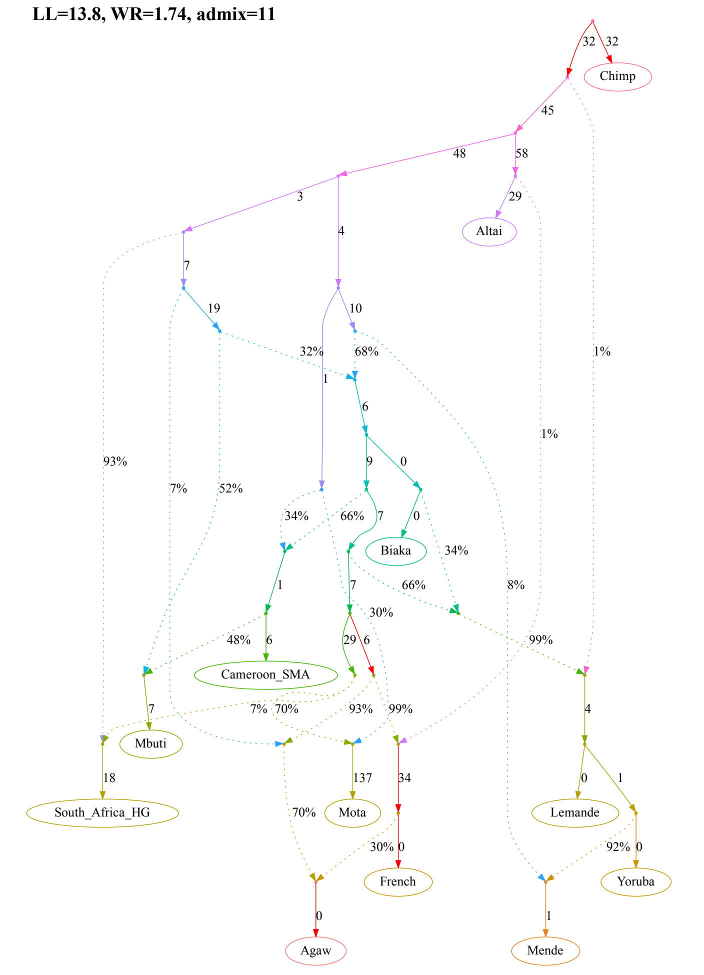	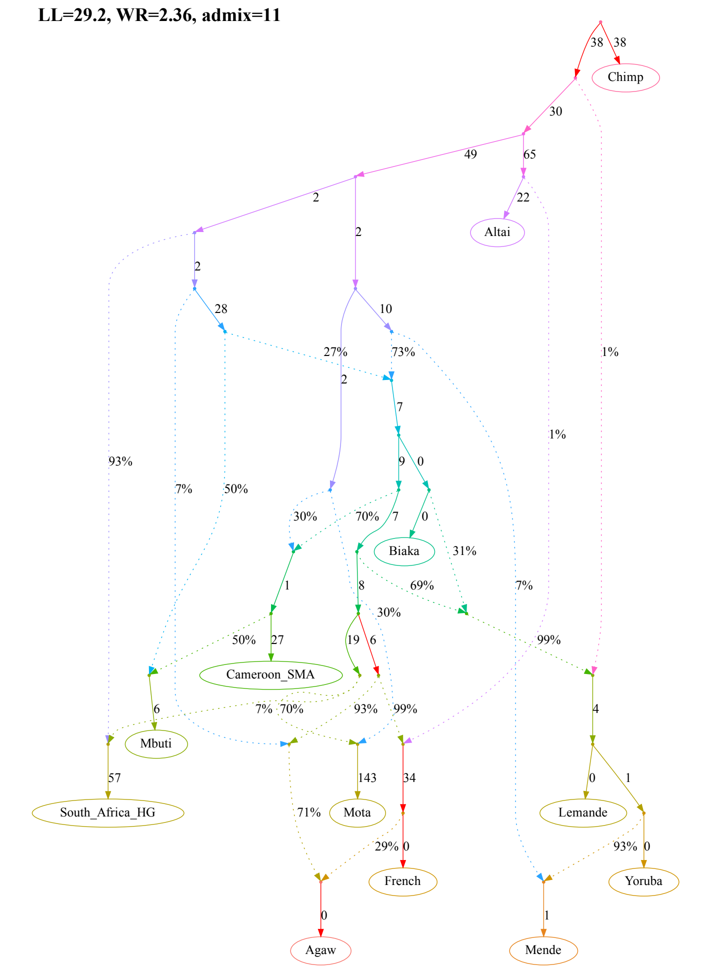

**support for claim 1**	partial
**support for claim 2**	not supported
**support for claim 3**	not supported
**support for claim 4**	partial

The original set of SNPs and individuals and the corrected algorithm for calculating f_3-statistics (211,738 variable site with no missing data at the group level)

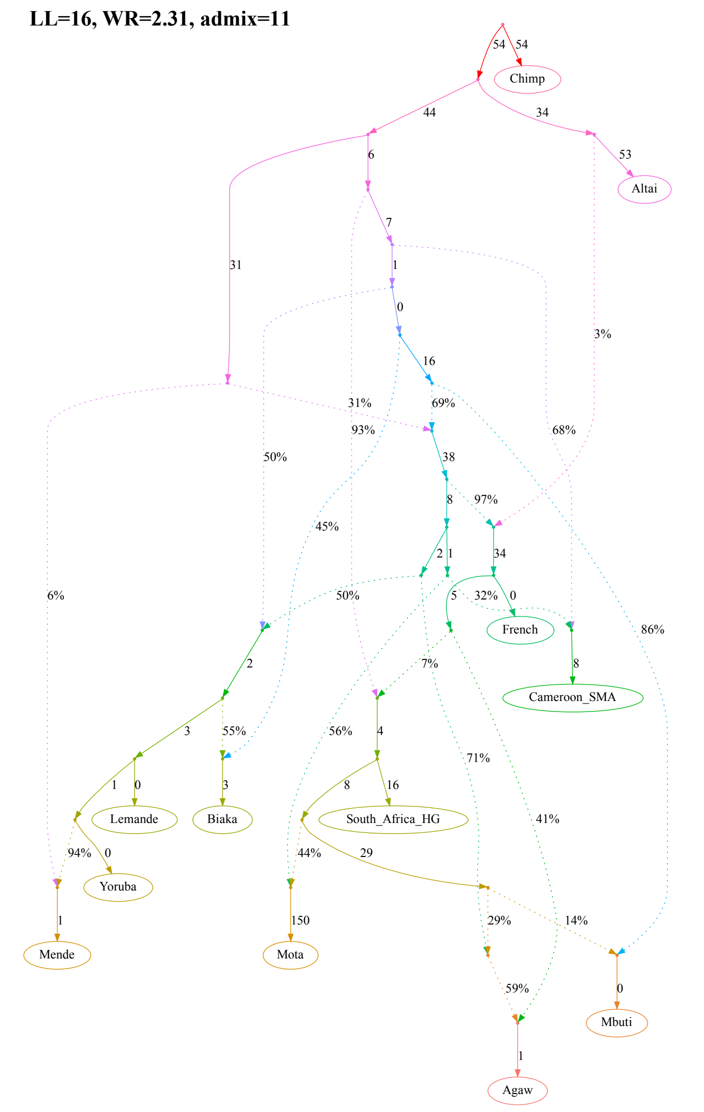

**support for claim 1**	partial
**support for claim 2**	not supported
**support for claim 3**	not supported
**support for claim 4**	partial

The original set of SNPs and individuals and the original algorithm for calculating f_3-statistics (363,131 variable site with no missing data at the group level)

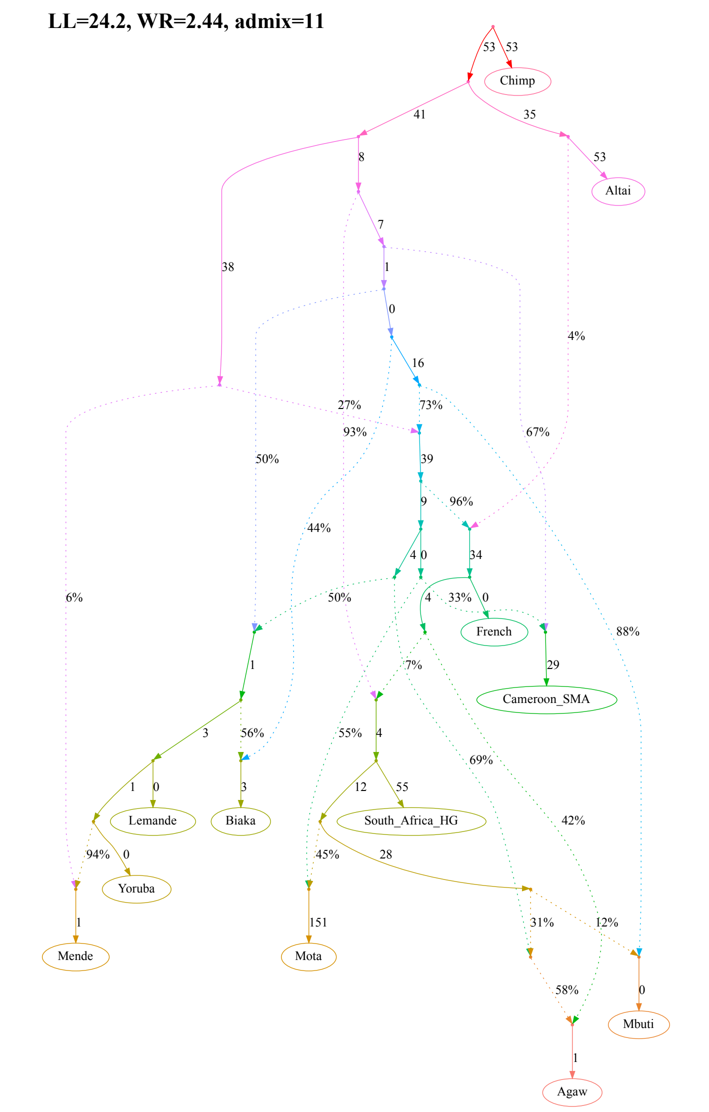

e, selected alternative models fitting nominally better than the published one

**support for claim 1** supported<br>**support for claim 2** not supported<br>**support for claim 3** partial<br>**support for claim 4** not supported	**support for claim 1** supported<br>**support for claim 2** not supported<br>**support for claim 3** partial<br>**support for claim 4** not supported
The original set of SNPs and individuals and the corrected algorithm for calculating *f*3-statistics (211,738 variable site with no missing data at the group level)	The original set of SNPs and individuals and the original algorithm for calculating *f*3-statistics (363,131 variable site with no missing data at the group level)

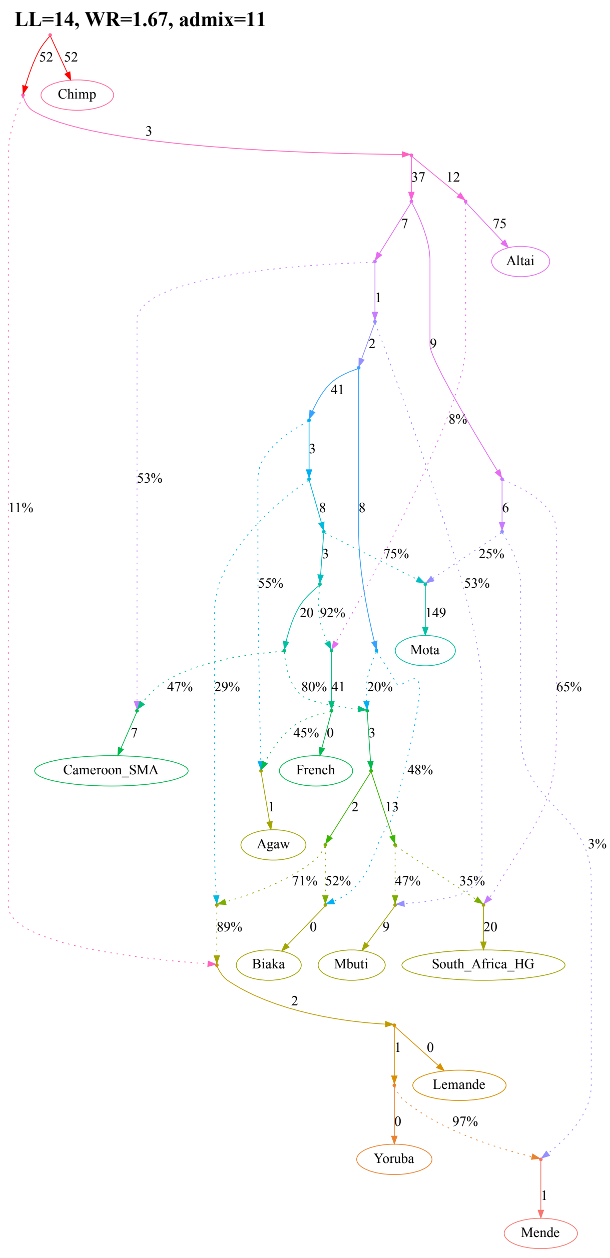

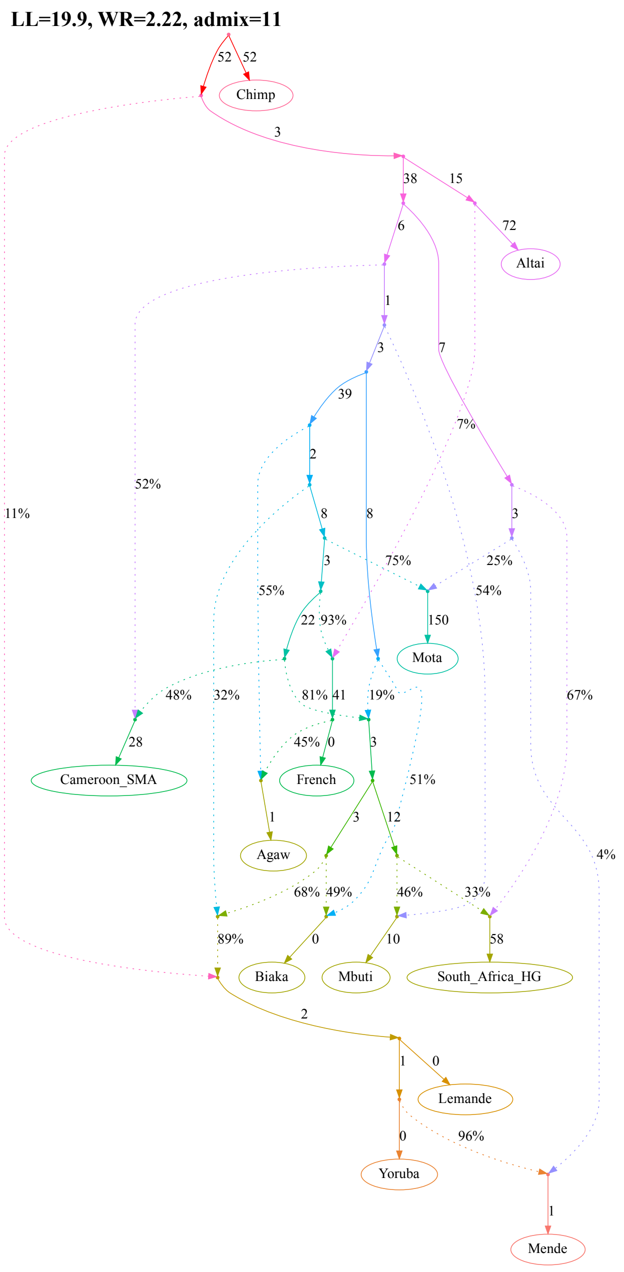

**support for claim 1** not supported<br>**support for claim 2** partial<br>**support for claim 3** partial<br>**support for claim 4** partial	**support for claim 1** not supported<br>**support for claim 2** partial<br>**support for claim 3** partial<br>**support for claim 4** partial
The original set of SNPs and individuals and the corrected algorithm for calculating f_3-statistics (211,738 variable site with no missing data at the group level)	The original set of SNPs and individuals and the original algorithm for calculating f_3-statistics (363,131 variable site with no missing data at the group level)

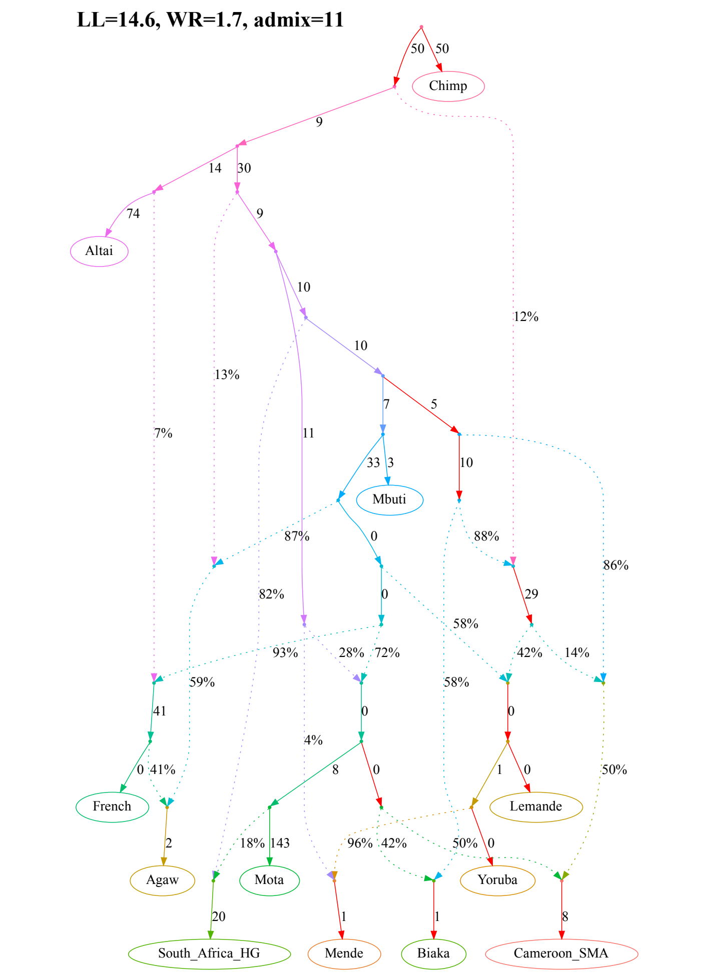

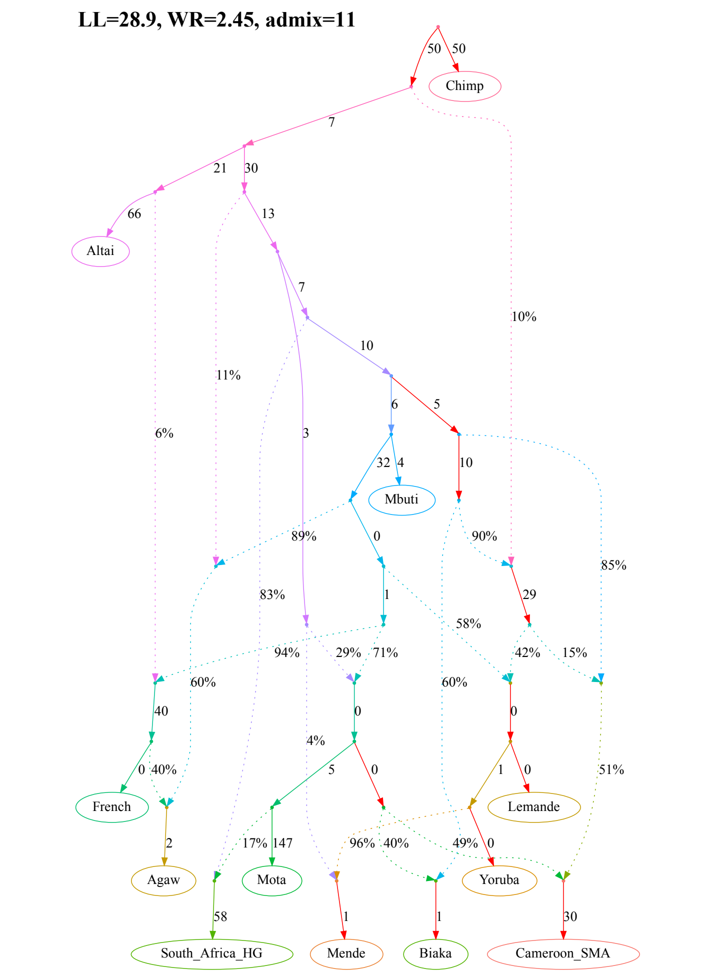

f, selected alternative models fitting nominally better than the published one

**support for claim 1** partial<br>**support for claim 2** partial<br>**support for claim 3** not supported<br>**support for claim 4** partial	**support for claim 1** partial<br>**support for claim 2** partial<br>**support for claim 3** not supported<br>**support for claim 4** partial
The original set of SNPs and individuals and the corrected algorithm for calculating f_3-statistics (211,738 variable site with no missing data at the group level)	The original set of SNPs and individuals and the original algorithm for calculating f_3-statistics (363,131 variable site with no missing data at the group level)

LL=15.9, WR=2.17, admix=11

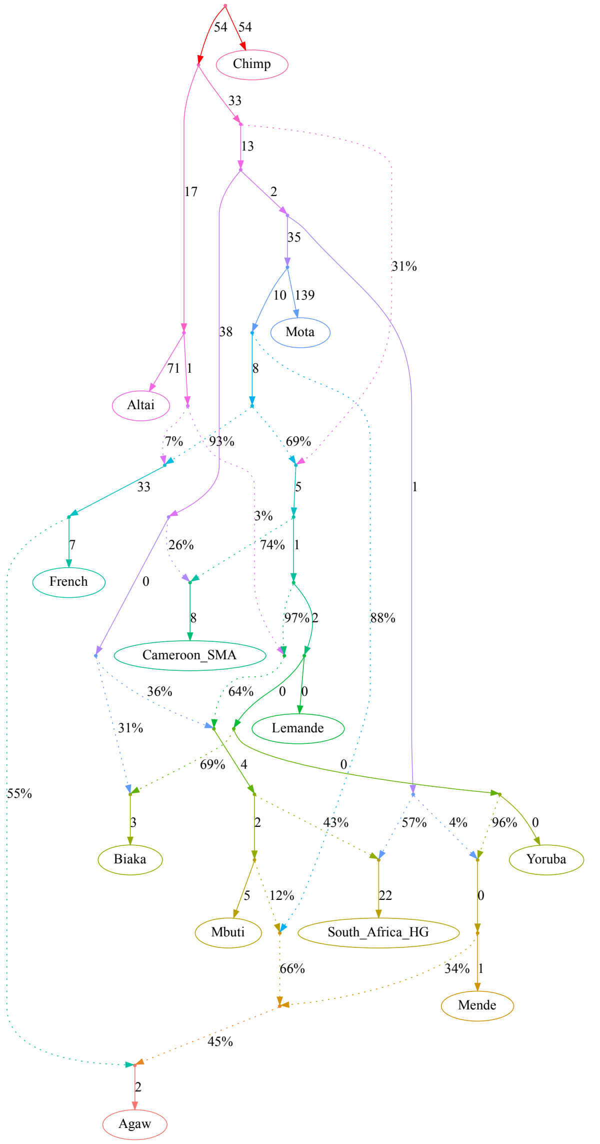

LL=27, WR=2.44, admix=11

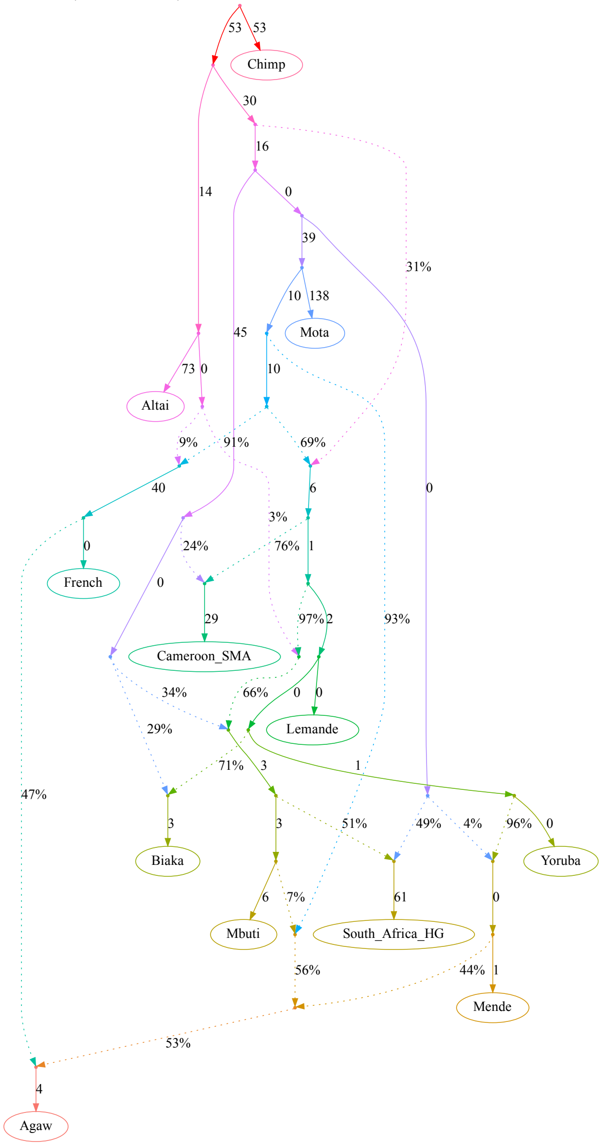

**support for claim 1**	partial
**support for claim 2**	not supported
**support for claim 3**	partial
**support for claim 4**	partial

The original set of SNPs and individuals and the corrected algorithm for calculating *f*₃-statistics (211,738 variable site with no missing data at the group level)

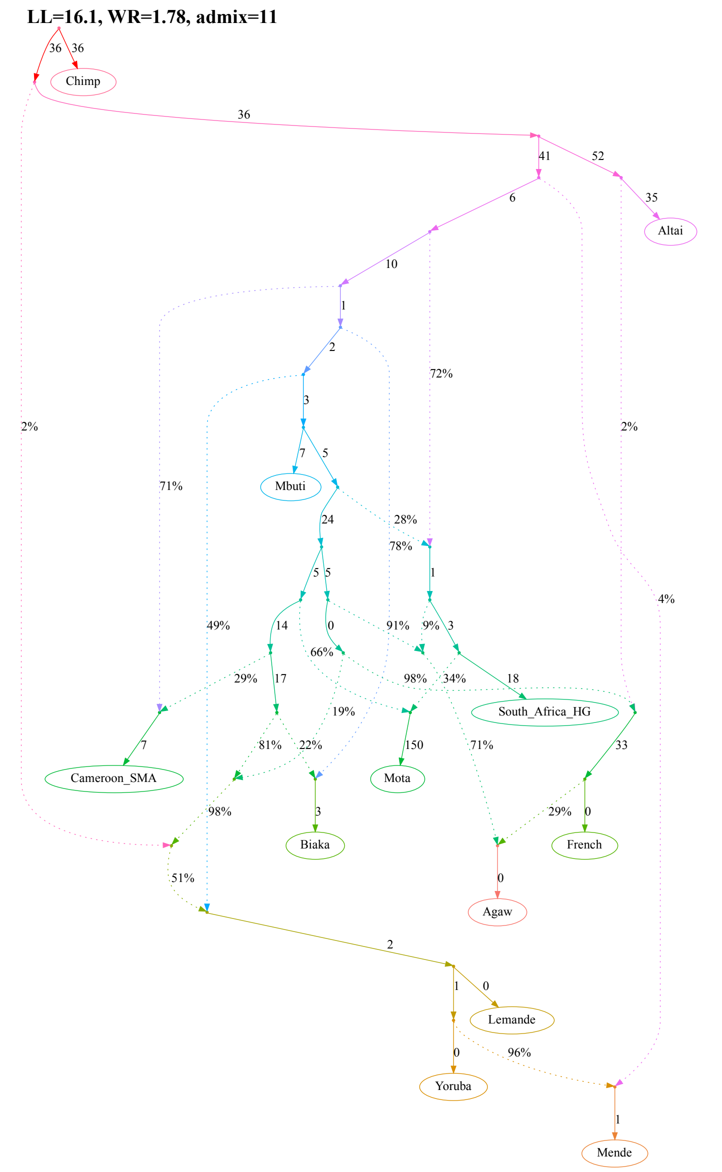

**support for claim 1**	partial
**support for claim 2**	not supported
**support for claim 3**	partial
**support for claim 4**	partial

The original set of SNPs and individuals and the original algorithm for calculating *f*₃-statistics (363,131 variable site with no missing data at the group level)

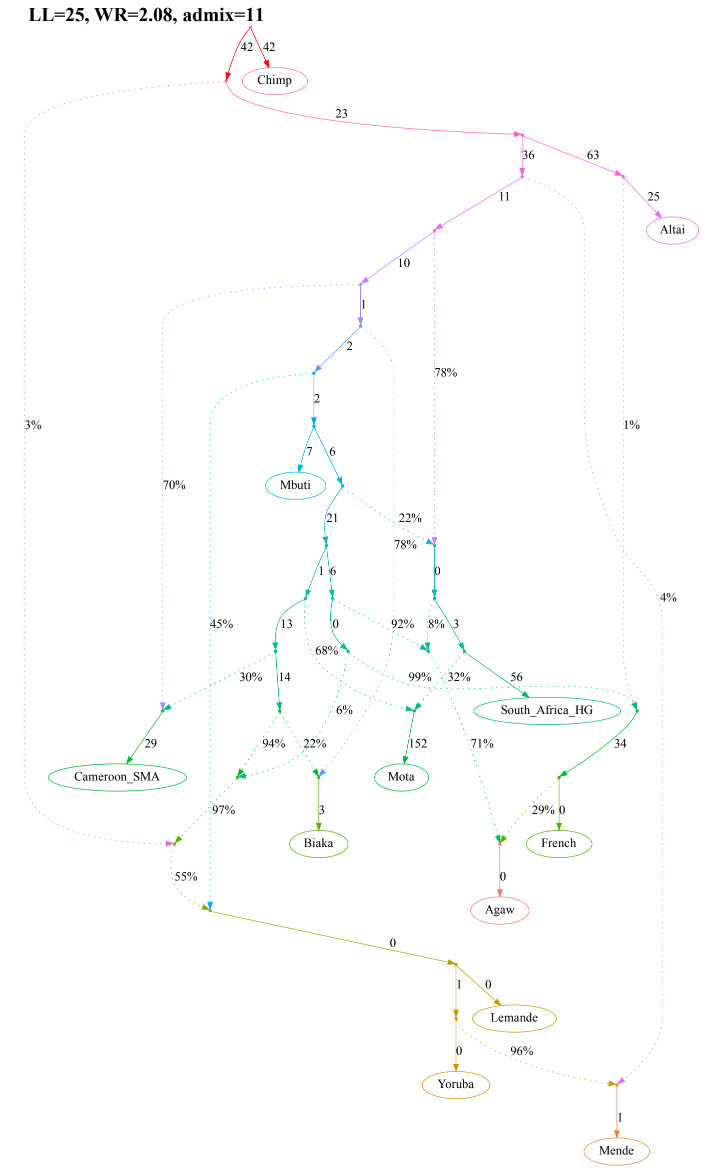

g, selected alternative models fitting nominally better than the published one

**support for claim 1** supported<br>**support for claim 2** partial<br>**support for claim 3** not supported<br>**support for claim 4** partial	**support for claim 1** supported<br>**support for claim 2** partial<br>**support for claim 3** not supported<br>**support for claim 4** partial
The original set of SNPs and individuals and the corrected algorithm for calculating f_3-statistics (211,738 variable site with no missing data at the group level)	The original set of SNPs and individuals and the original algorithm for calculating f_3-statistics (363,131 variable site with no missing data at the group level)
**LL=10.5, WR=1.58, admix=11**<br>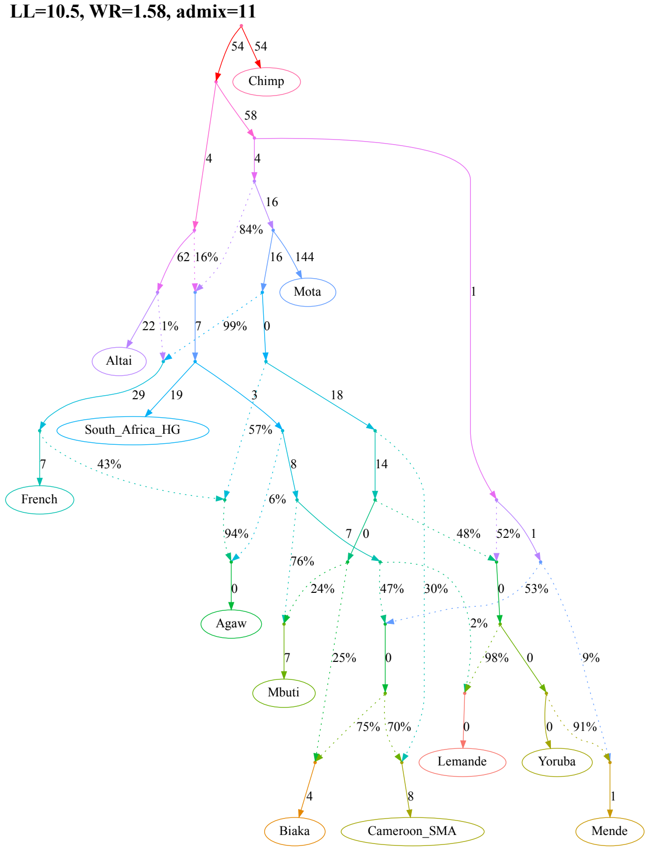	**LL=23.8, WR=1.89, admix=11**<br>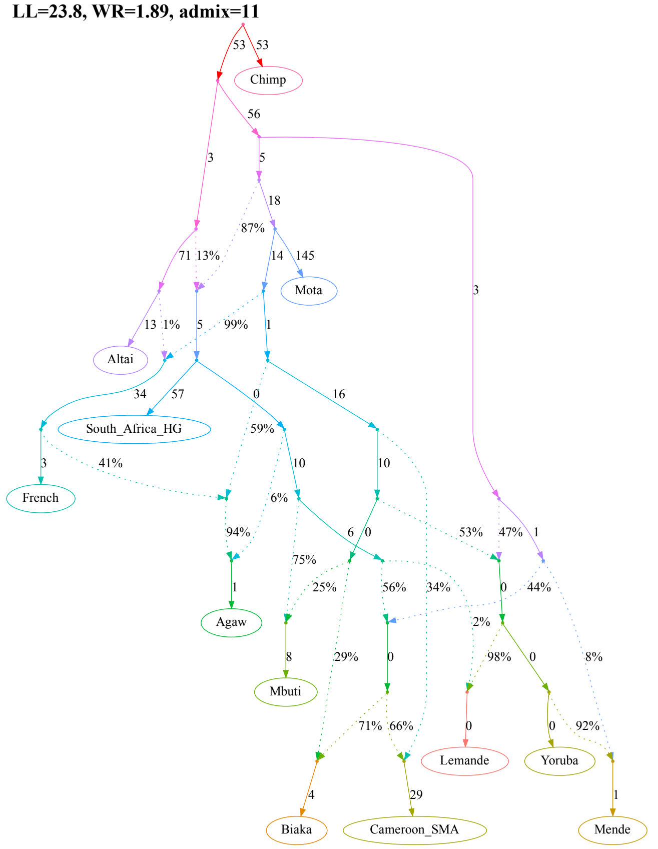

<table>
<tr>
<td>

**support for claim 1**	partial
**support for claim 2**	partial
**support for claim 3**	supported
**support for claim 4**	not supported

</td>
<td>

**support for claim 1**	partial
**support for claim 2**	partial
**support for claim 3**	supported
**support for claim 4**	not supported

</td>
</tr>
<tr>
<td>The original set of SNPs and individuals and the corrected algorithm for calculating f_3-statistics (211,738 variable site with no missing data at the group level)</td>
<td>The original set of SNPs and individuals and the original algorithm for calculating f_3-statistics (363,131 variable site with no missing data at the group level)</td>
</tr>
<tr>
<td>

LL=13, WR=1.78, admix=11

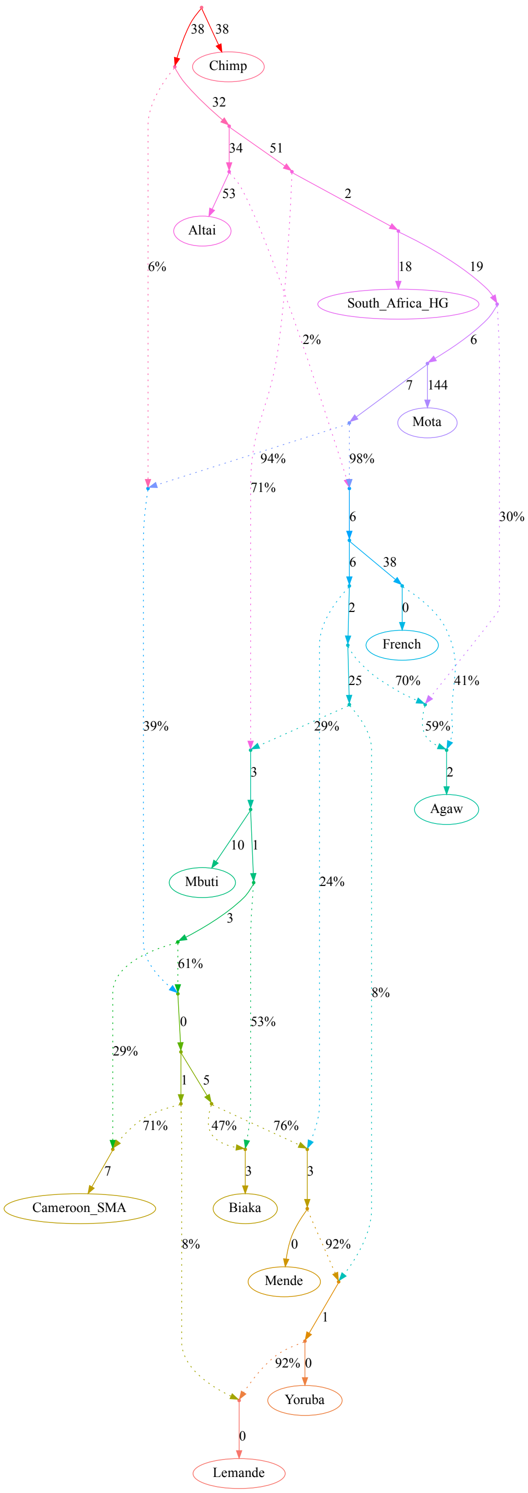

</td>
<td>

LL=36.4, WR=3, admix=11

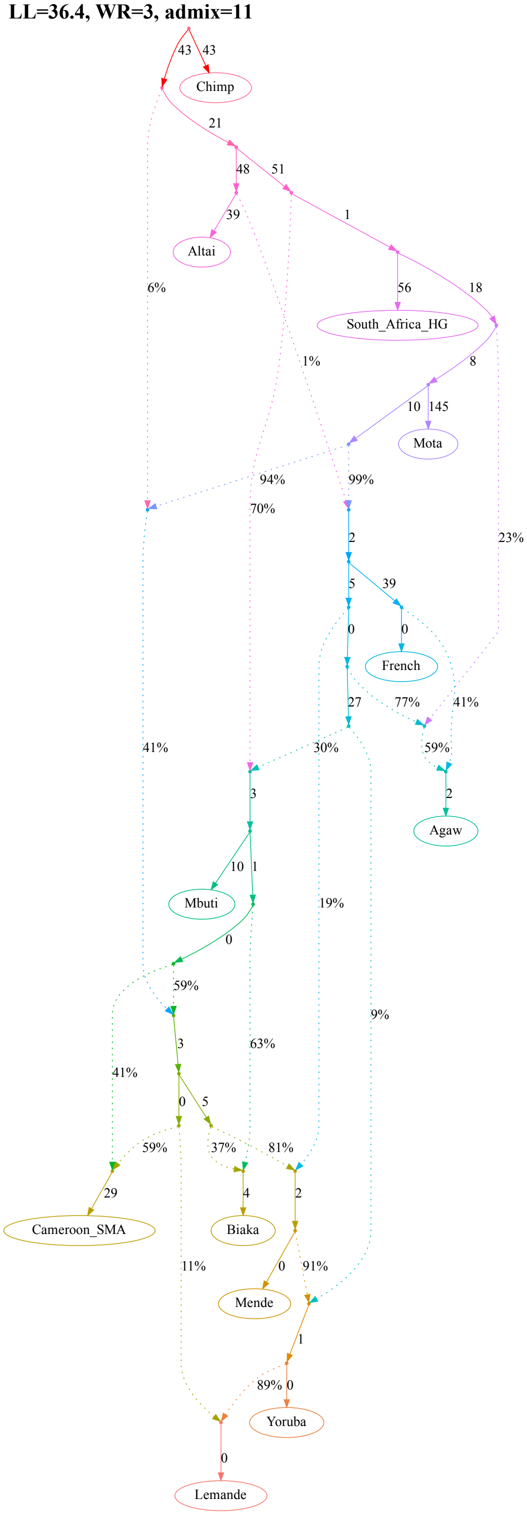

</td>
</tr>
</table>

h, selected alternative models fitting nominally better than the published one

**support for claim 1** partial<br>**support for claim 2** partial<br>**support for claim 3** supported<br>**support for claim 4** not supported	**support for claim 1** partial<br>**support for claim 2** partial<br>**support for claim 3** supported<br>**support for claim 4** not supported
The original set of SNPs and individuals and the corrected algorithm for calculating f_3-statistics (211,738 variable site with no missing data at the group level)	The original set of SNPs and individuals and the original algorithm for calculating f_3-statistics (363,131 variable site with no missing data at the group level)

LL=13.3, WR=1.74, admix=11

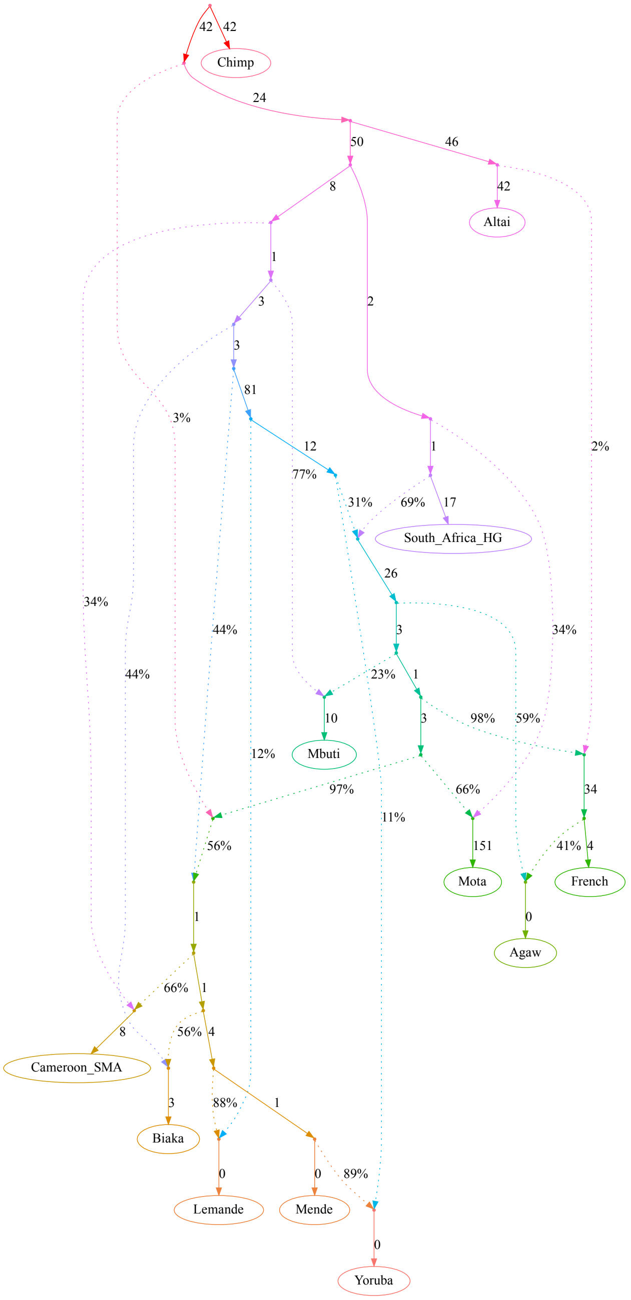

LL=24.4, WR=2.26, admix=11

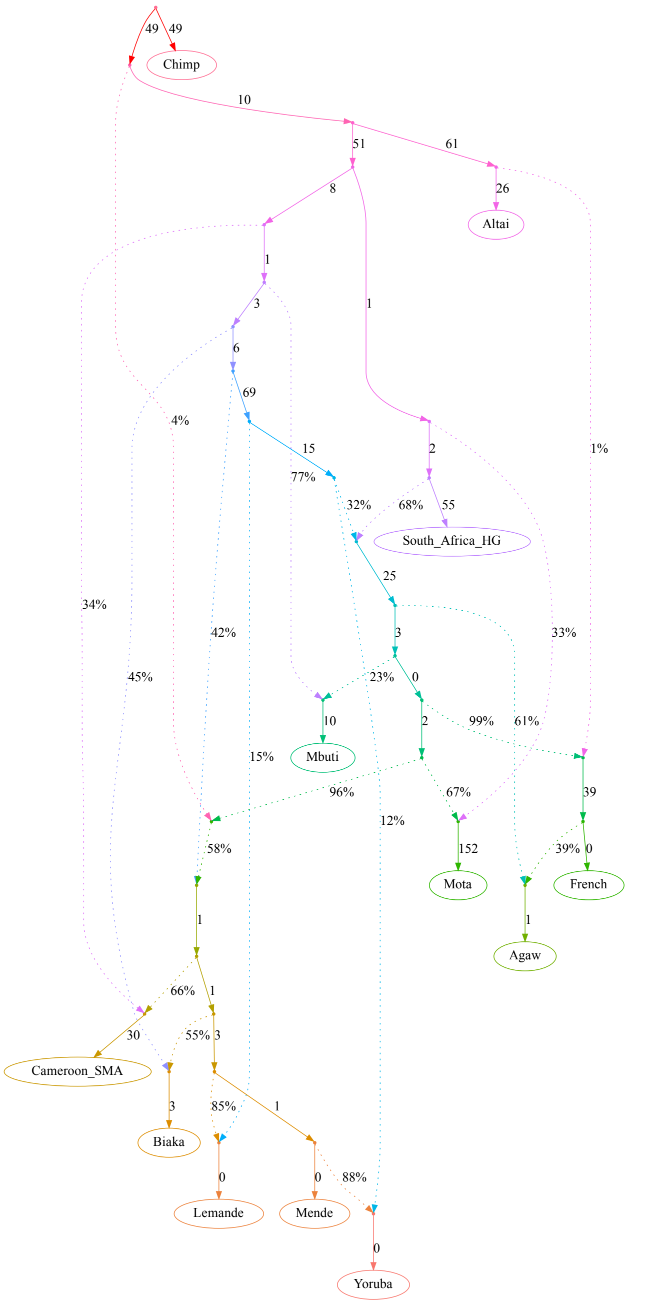

**support for claim 1**	partial
**support for claim 2**	partial
**support for claim 3**	supported
**support for claim 4**	not supported

The original set of SNPs and individuals and the corrected algorithm for calculating f_3-statistics (211,738 variable site with no missing data at the group level)

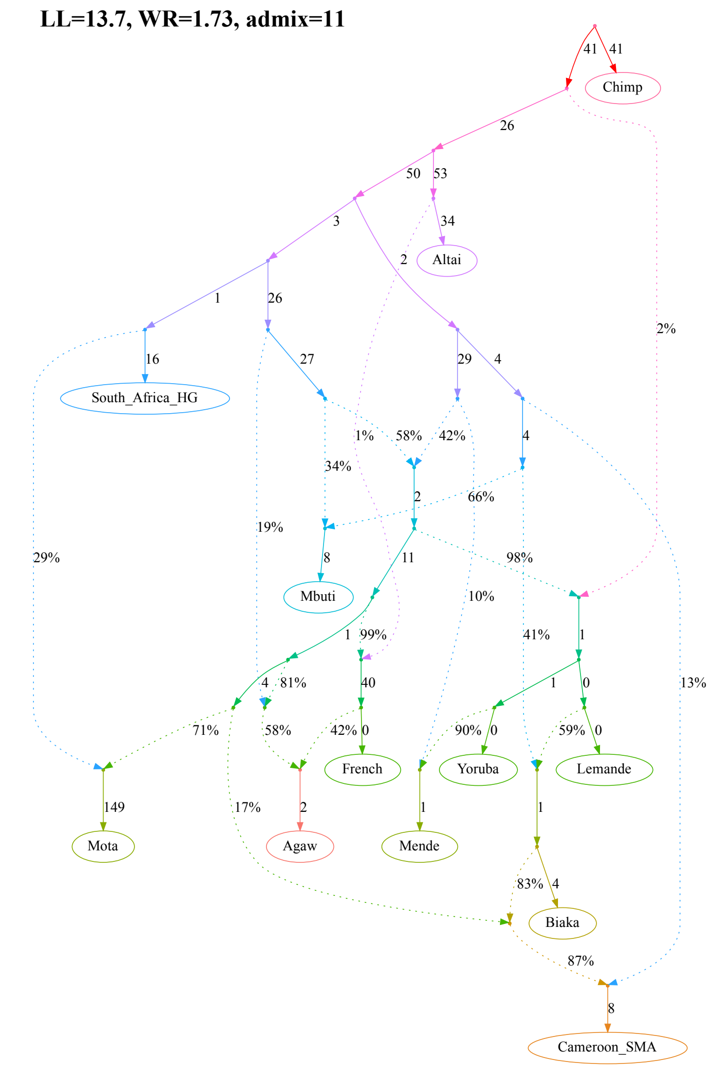

**support for claim 1**	partial
**support for claim 2**	partial
**support for claim 3**	supported
**support for claim 4**	not supported

The original set of SNPs and individuals and the original algorithm for calculating f_3-statistics (363,131 variable site with no missing data at the group level)

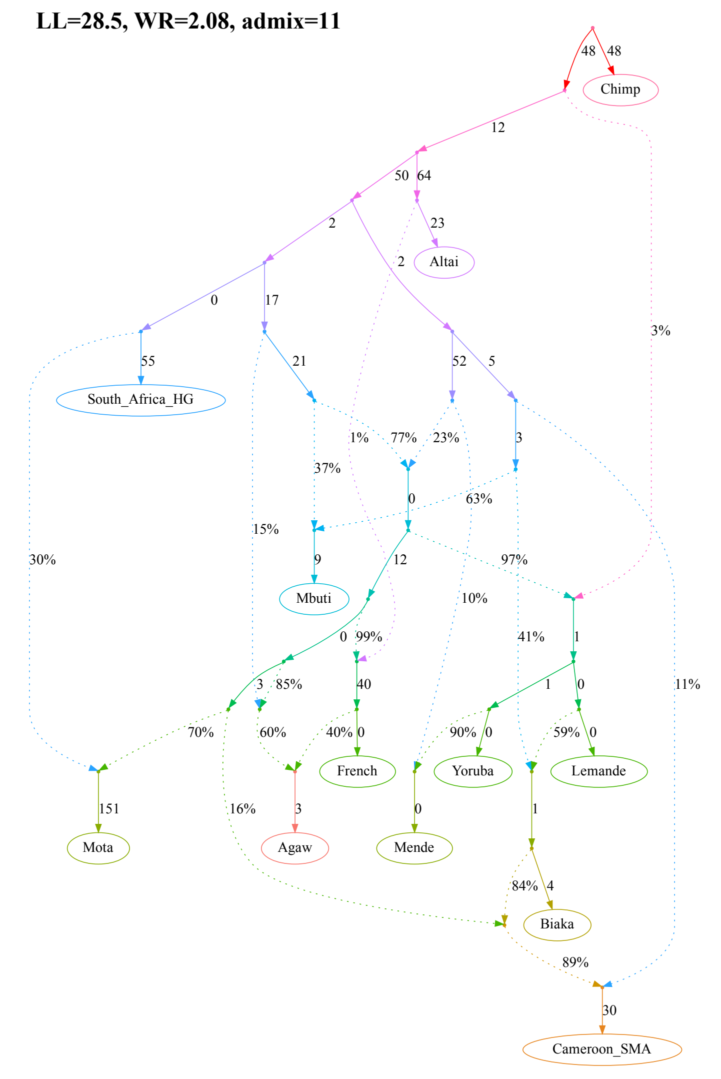

i, selected alternative models fitting nominally better than the published one

**support for claim 1** not supported<br>**support for claim 2** partial<br>**support for claim 3** partial<br>**support for claim 4** supported	**support for claim 1** not supported<br>**support for claim 2** partial<br>**support for claim 3** partial<br>**support for claim 4** supported
The original set of SNPs and individuals and the corrected algorithm for calculating f_3-statistics (211,738 variable site with no missing data at the group level)	The original set of SNPs and individuals and the original algorithm for calculating f_3-statistics (363,131 variable site with no missing data at the group level)
**LL=14.2, WR=1.95, admix=11**<br>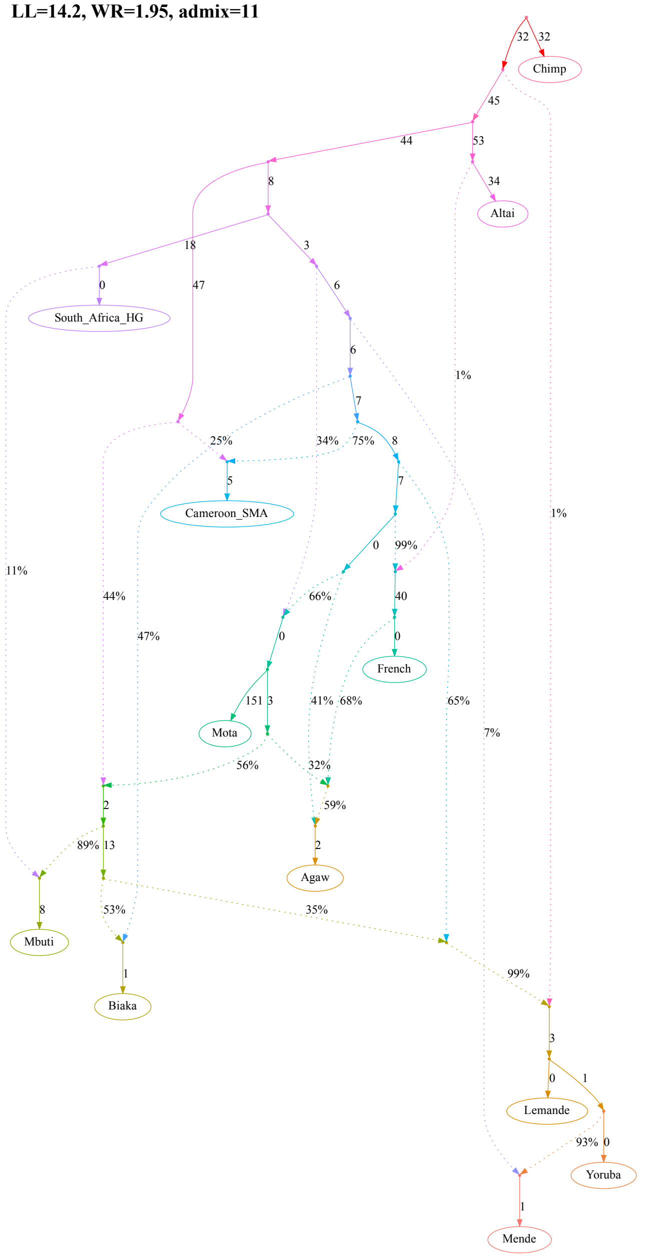	**LL=28.3, WR=2.63, admix=11**<br>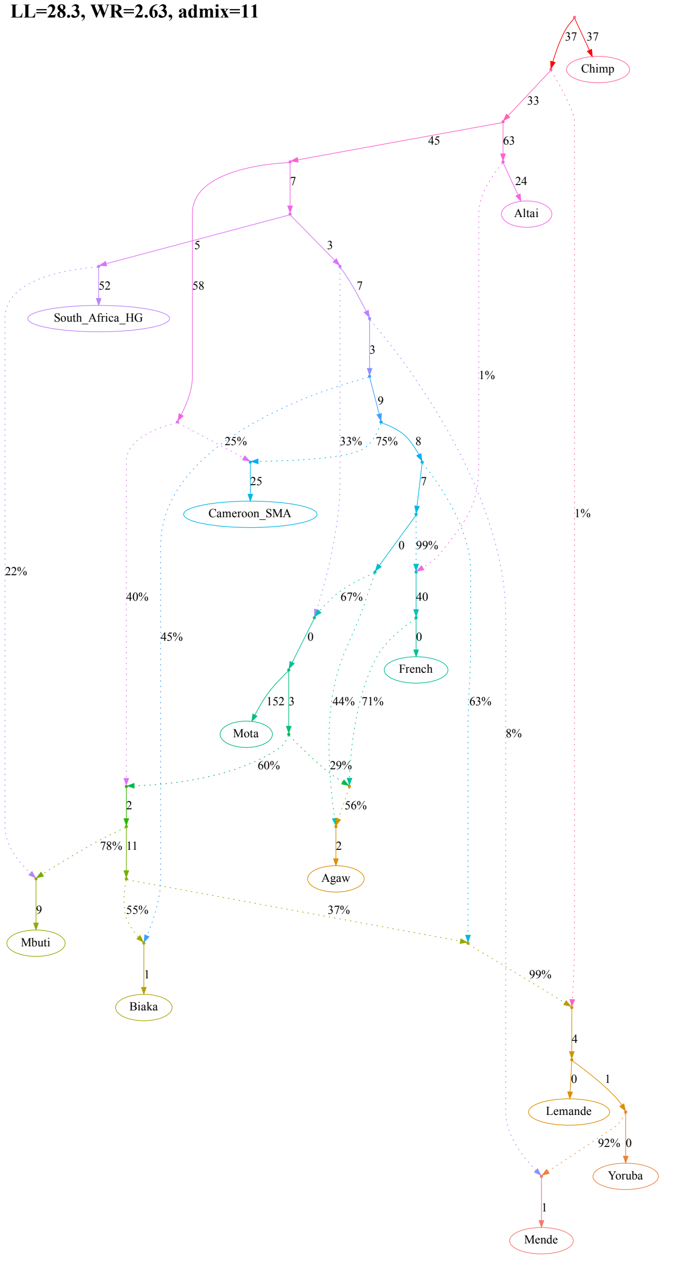

<table>
<tr>
<td>

support for claim 1 supported
support for claim 2 partial
support for claim 3 not supported
support for claim 4 partial

</td>
<td>

support for claim 1 supported
support for claim 2 partial
support for claim 3 not supported
support for claim 4 partial

</td>
</tr>
<tr>
<td>The original set of SNPs and individuals and the corrected algorithm for calculating f_3-statistics (211,738 variable site with no missing data at the group level)</td>
<td>The original set of SNPs and individuals and the original algorithm for calculating f_3-statistics (363,131 variable site with no missing data at the group level)</td>
</tr>
<tr>
<td>

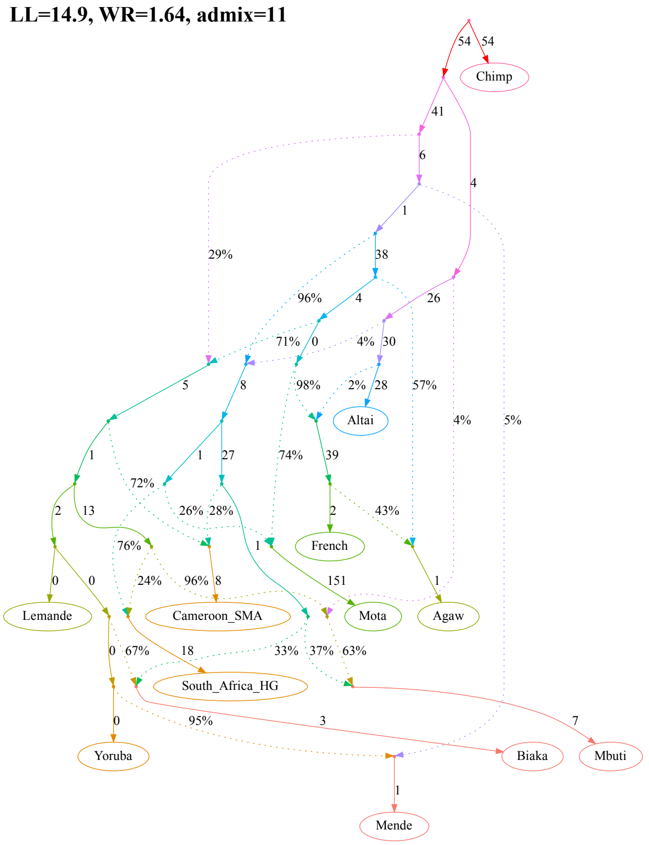

</td>
<td>

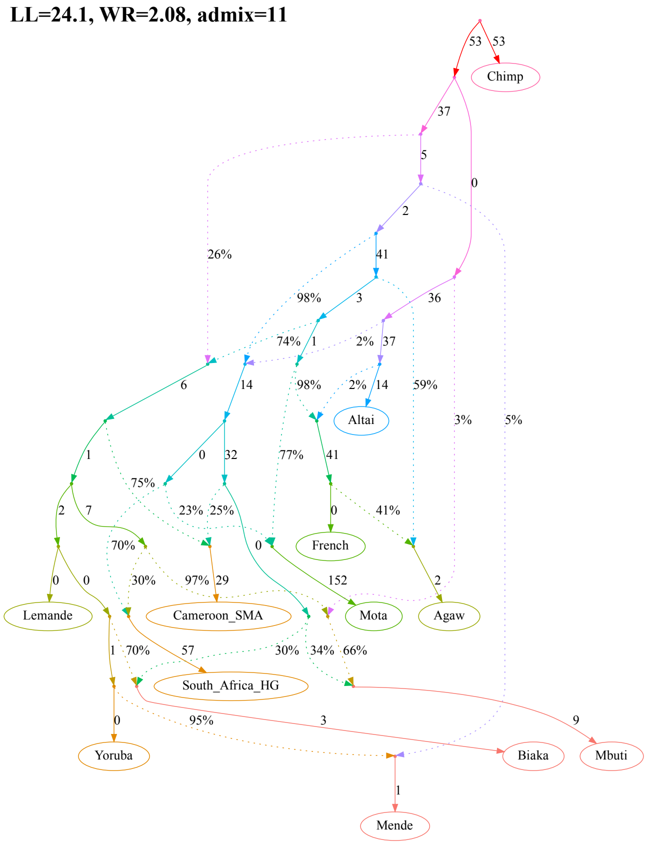

</td>
</tr>
</table>

j, selected alternative models fitting nominally better than the published one

<table>
<tr>
<td>

**support for claim 1**	supported
**support for claim 2**	partial
**support for claim 3**	partial
**support for claim 4**	not supported

</td>
<td>

**support for claim 1**	supported
**support for claim 2**	partial
**support for claim 3**	partial
**support for claim 4**	not supported

</td>
</tr>
<tr>
<td>The original set of SNPs and individuals and the corrected algorithm for calculating f_3-statistics (211,738 variable site with no missing data at the group level)</td>
<td>The original set of SNPs and individuals and the original algorithm for calculating f_3-statistics (363,131 variable site with no missing data at the group level)</td>
</tr>
<tr>
<td>

LL=15.2, WR=1.66, admix=11

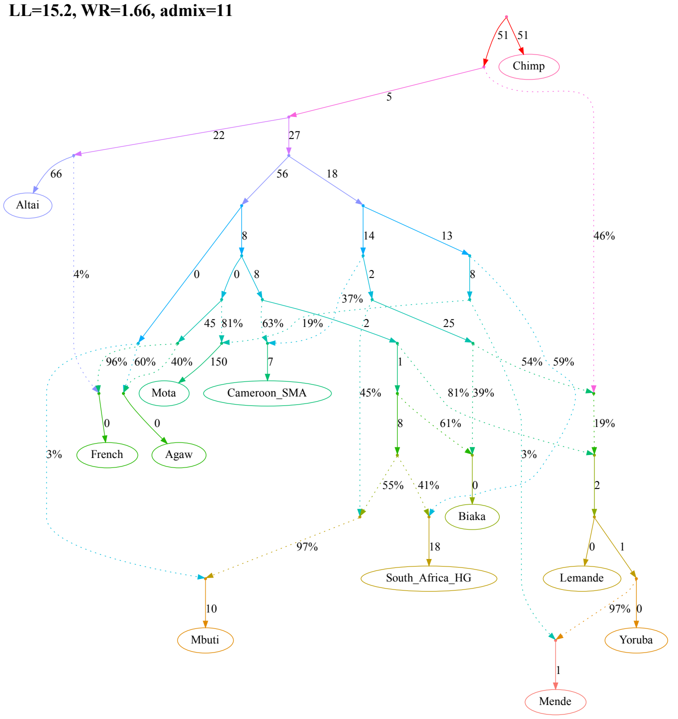

</td>
<td>

LL=26.5, WR=2.44, admix=11

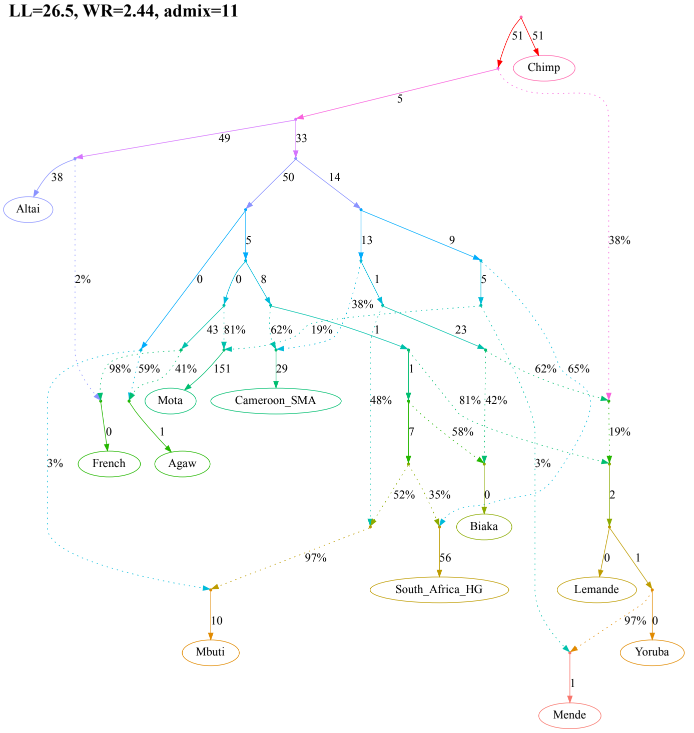

</td>
</tr>
</table>

**support for claim 1** supported<br>**support for claim 2** not supported<br>**support for claim 3** partial<br>**support for claim 4** partial	**support for claim 1** supported<br>**support for claim 2** not supported<br>**support for claim 3** partial<br>**support for claim 4** partial
The original set of SNPs and individuals and the corrected algorithm for calculating f_3-statistics (211,738 variable site with no missing data at the group level)	The original set of SNPs and individuals and the original algorithm for calculating f_3-statistics (363,131 variable site with no missing data at the group level)
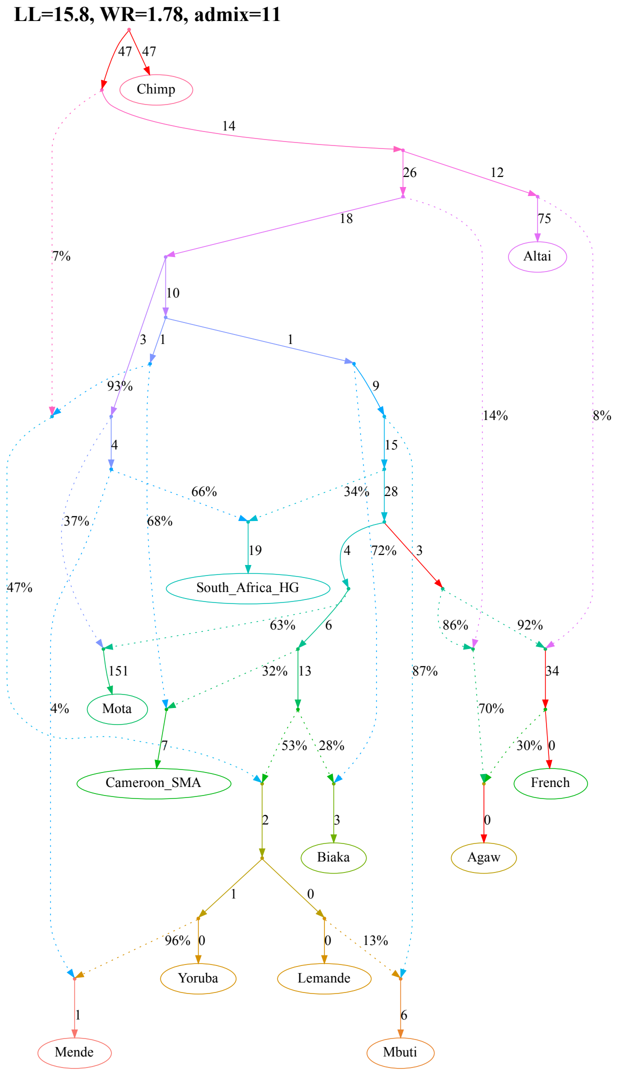	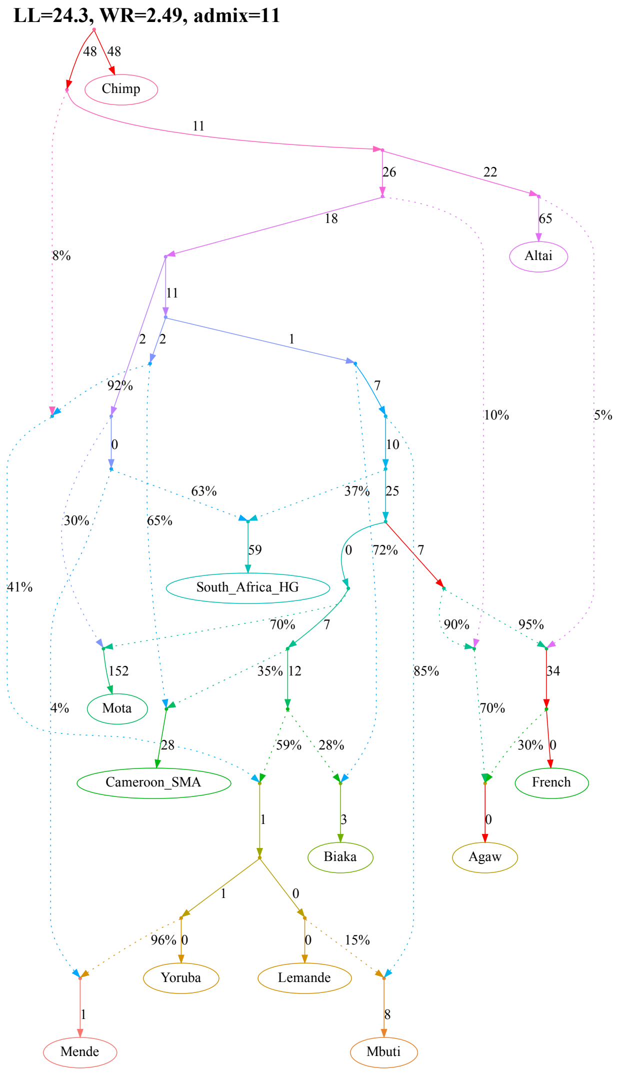

k, selected alternative models fitting nominally better than the published one

**support for claim 1** supported<br>**support for claim 2** supported<br>**support for claim 3** supported<br>**support for claim 4** not supported	**support for claim 1** supported<br>**support for claim 2** supported<br>**support for claim 3** supported<br>**support for claim 4** not supported
The original set of SNPs and individuals and the corrected algorithm for calculating f_3-statistics (211,738 variable site with no missing data at the group level)	The original set of SNPs and individuals and the original algorithm for calculating f_3-statistics (363,131 variable site with no missing data at the group level)

LL=16.1, WR=1.83, admix=11

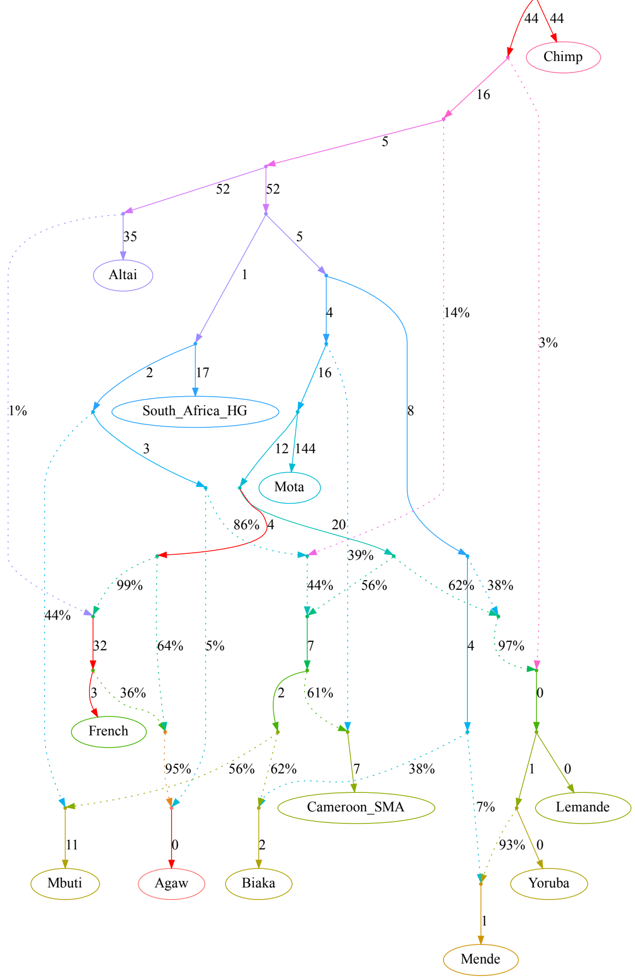

LL=34.5, WR=2.57, admix=11

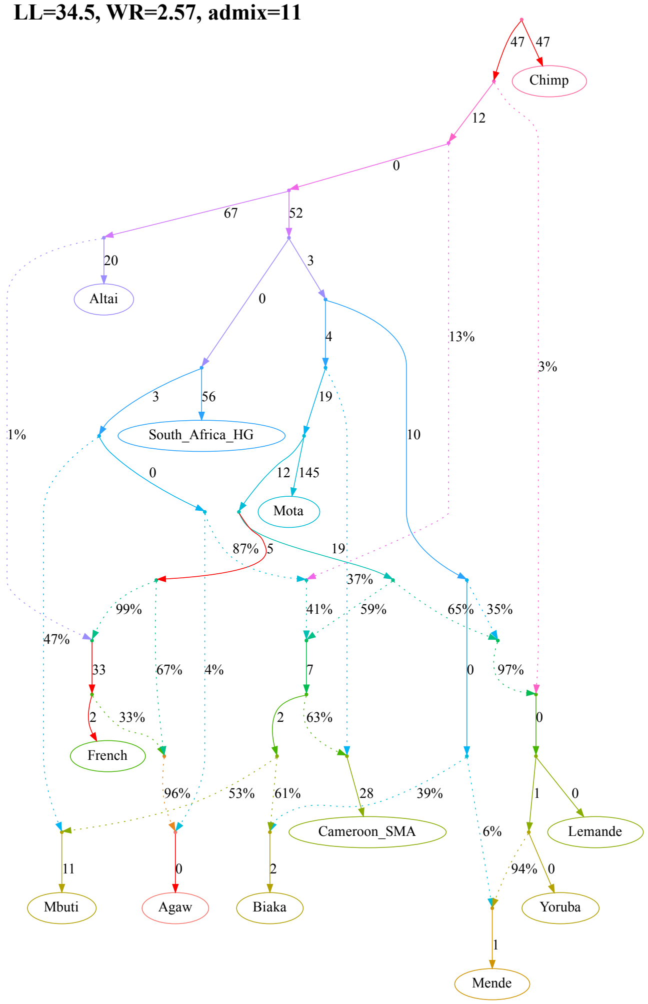

**support for claim 1** partial<br>**support for claim 2** partial<br>**support for claim 3** partial<br>**support for claim 4** partial	**support for claim 1** partial<br>**support for claim 2** partial<br>**support for claim 3** partial<br>**support for claim 4** partial
The original set of SNPs and individuals and the corrected algorithm for calculating f_3-statistics (211,738 variable site with no missing data at the group level)	The original set of SNPs and individuals and the original algorithm for calculating f_3-statistics (363,131 variable site with no missing data at the group level)

LL=13.3, WR=1.78, admix=11

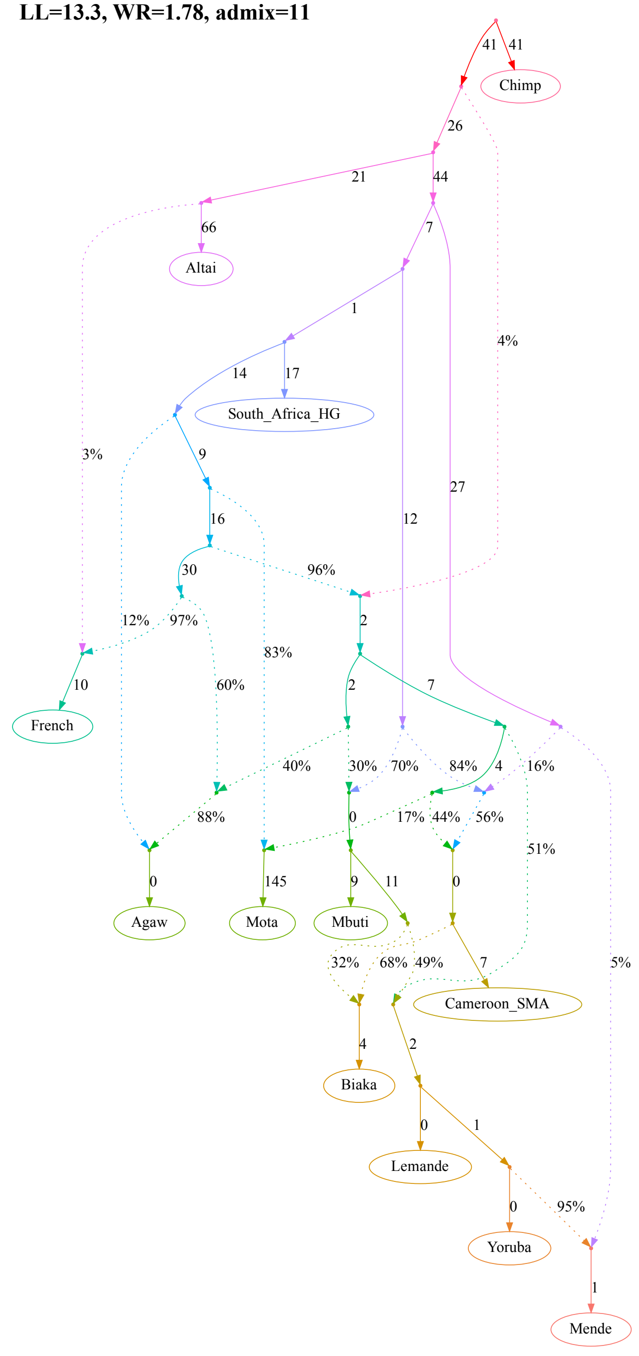

LL=23.3, WR=2.28, admix=11

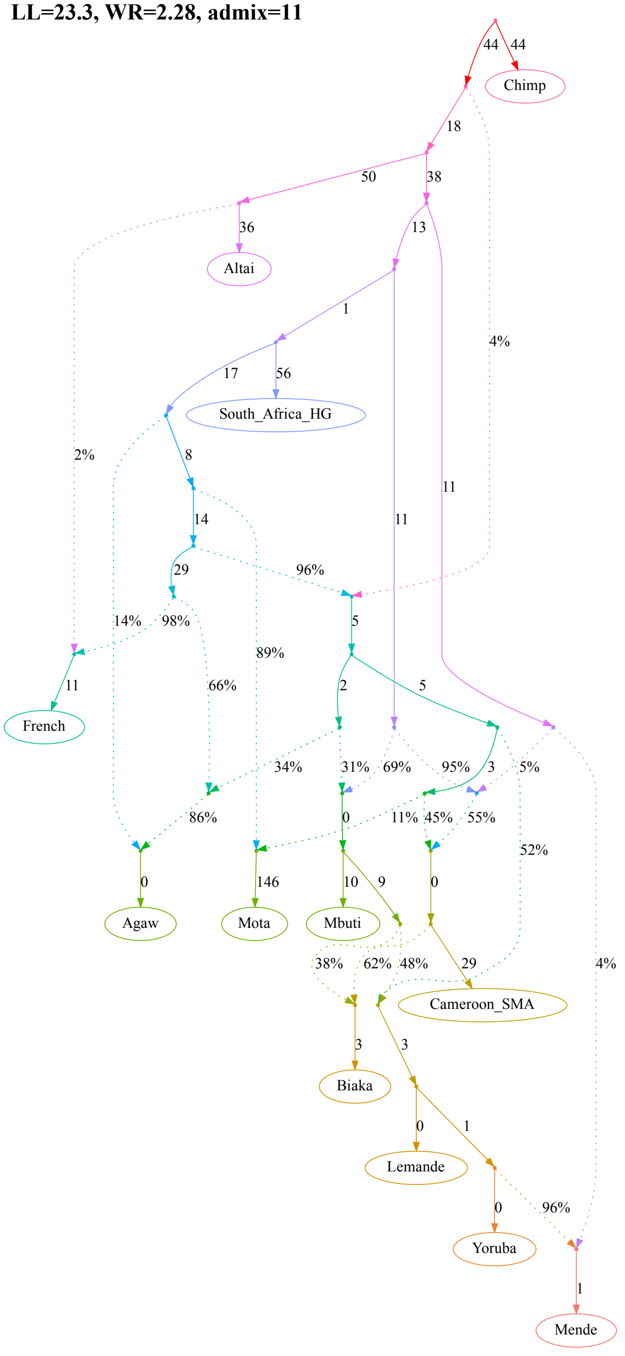

I, selected alternative models fitting nominally better than the published one

**support for claim 1** partial<br>**support for claim 2** partial<br>**support for claim 3** partial<br>**support for claim 4** partial	**support for claim 1** partial<br>**support for claim 2** partial<br>**support for claim 3** partial<br>**support for claim 4** partial
The original set of SNPs and individuals and the corrected algorithm for calculating *f*$_3$-statistics (211,738 variable site with no missing data at the group level)	The original set of SNPs and individuals and the original algorithm for calculating *f*$_3$-statistics (363,131 variable site with no missing data at the group level)

LL=13.4, WR=1.41, admix=11

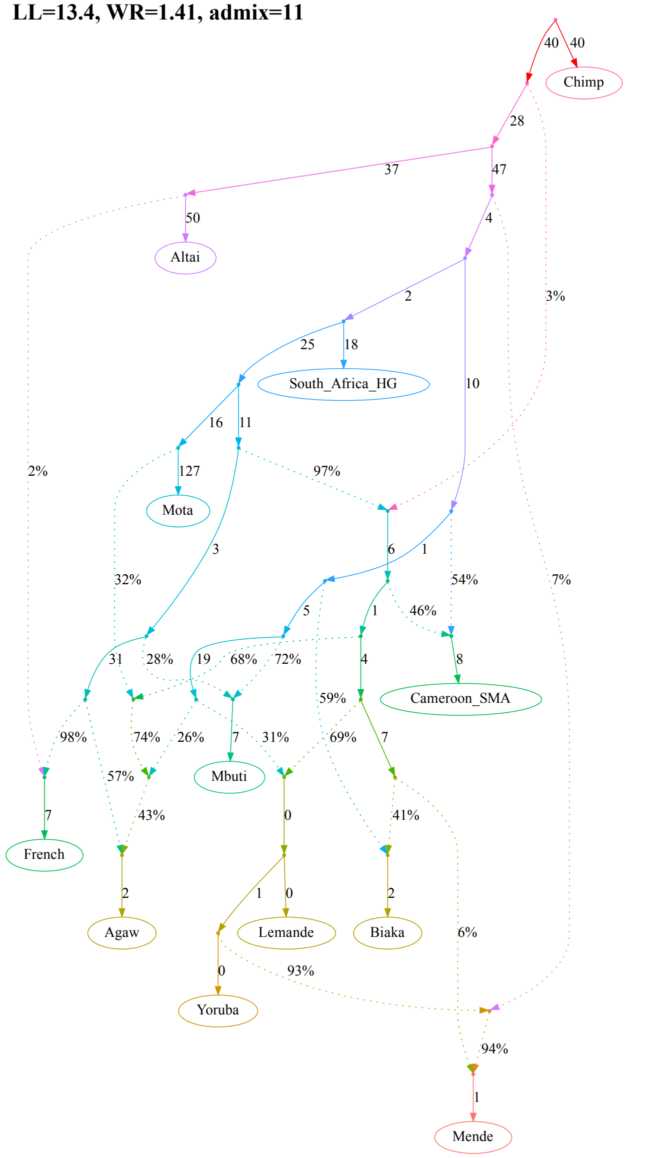

LL=19, WR=2.21, admix=11

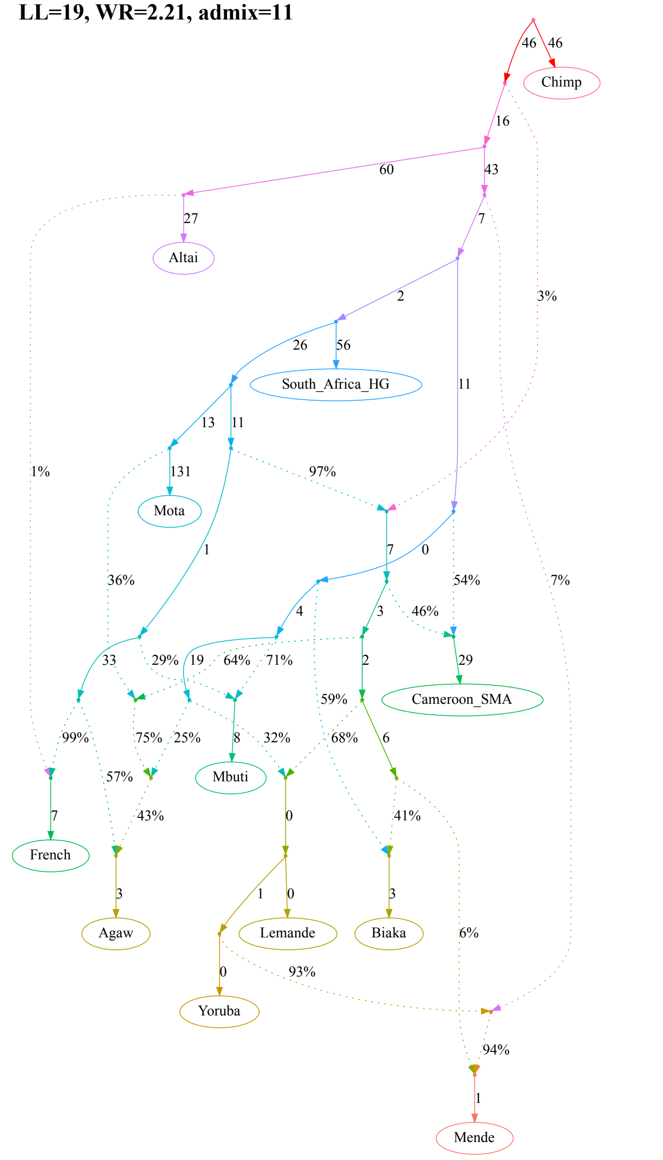

**support for claim 1** partial<br>**support for claim 2** partial<br>**support for claim 3** partial<br>**support for claim 4** partial	**support for claim 1** partial<br>**support for claim 2** partial<br>**support for claim 3** partial<br>**support for claim 4** partial
The original set of SNPs and individuals and the corrected algorithm for calculating *f_3*-statistics (211,738 variable site with no missing data at the group level)	The original set of SNPs and individuals and the original algorithm for calculating *f_3*-statistics (363,131 variable site with no missing data at the group level)
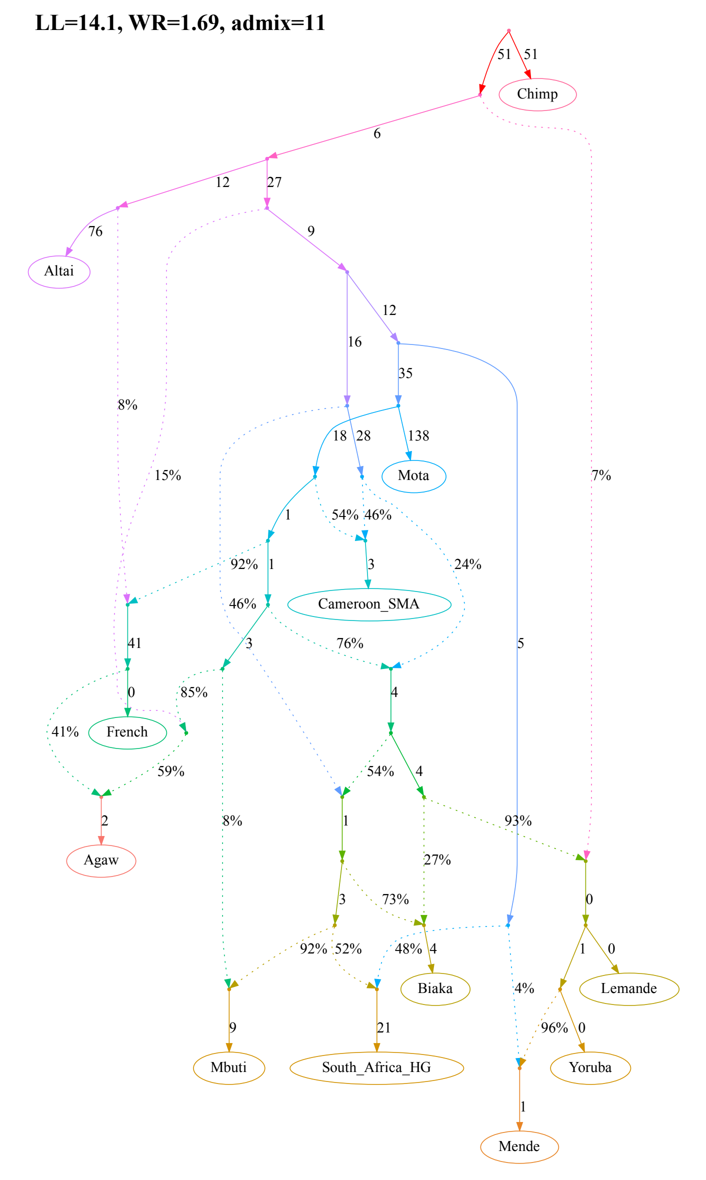	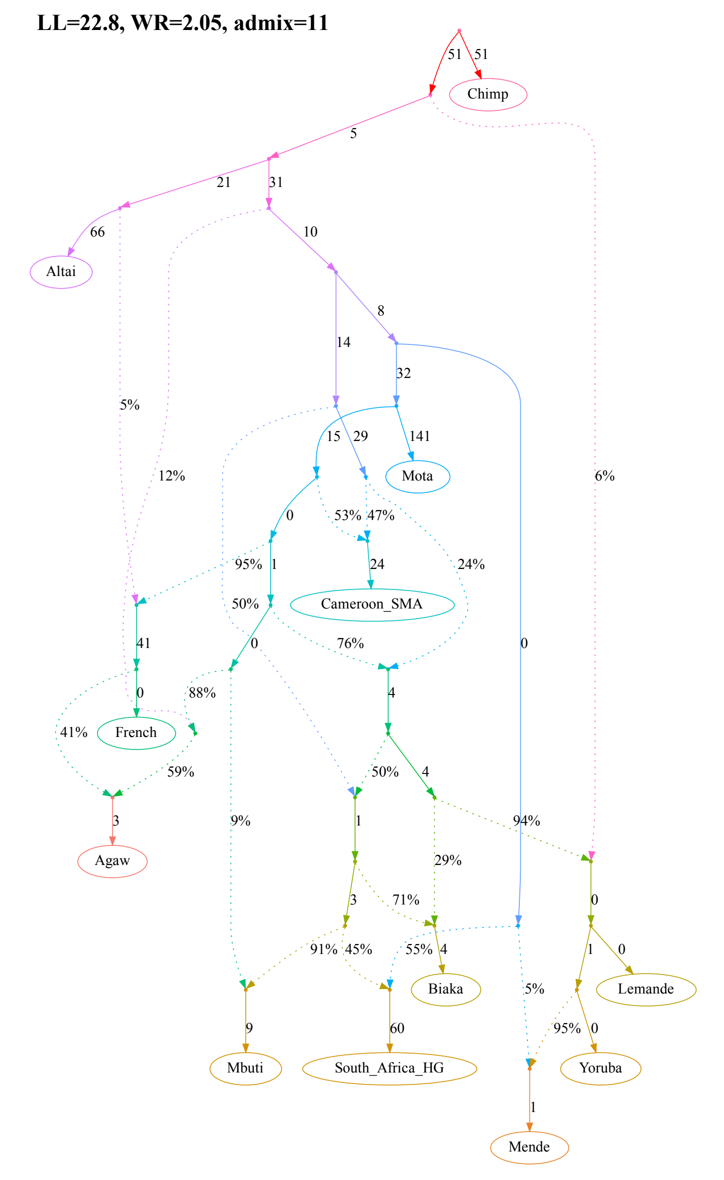

m, selected alternative models fitting nominally better than the published one

**support for claim 1** partial<br>**support for claim 2** partial<br>**support for claim 3** partial<br>**support for claim 4** partial	**support for claim 1** partial<br>**support for claim 2** partial<br>**support for claim 3** partial<br>**support for claim 4** partial
The original set of SNPs and individuals and the corrected algorithm for calculating f_3-statistics (211,738 variable site with no missing data at the group level)	The original set of SNPs and individuals and the original algorithm for calculating f_3-statistics (363,131 variable site with no missing data at the group level)

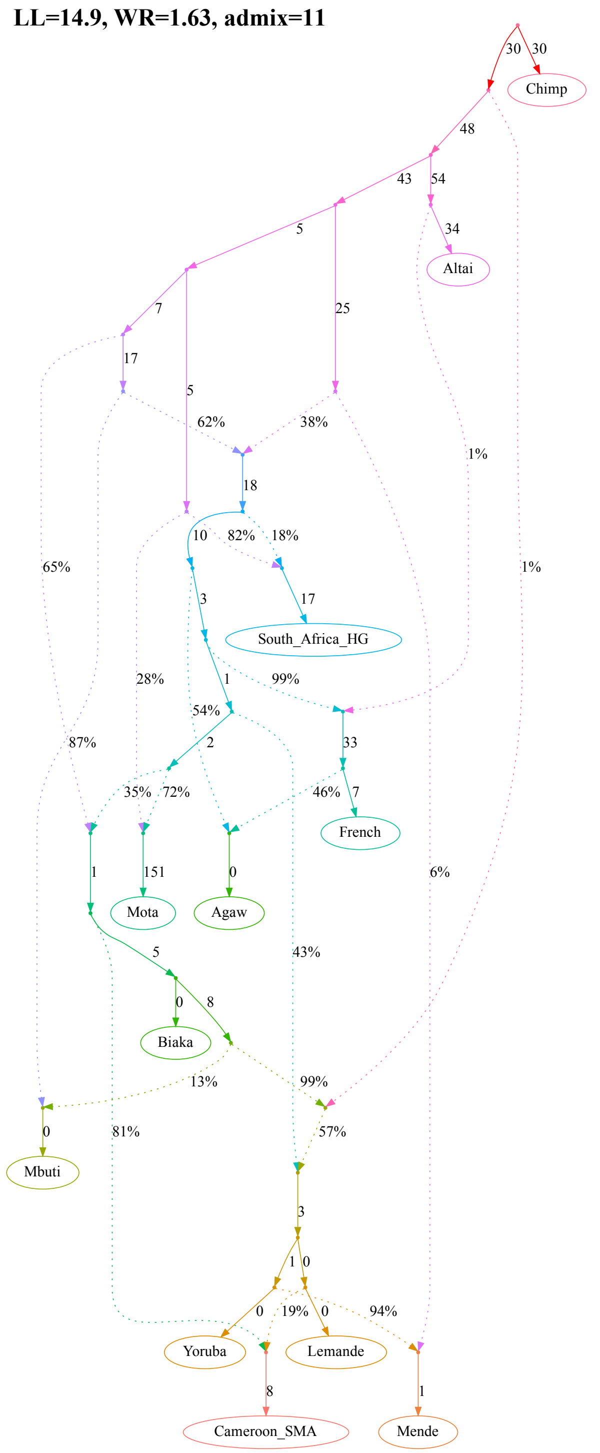

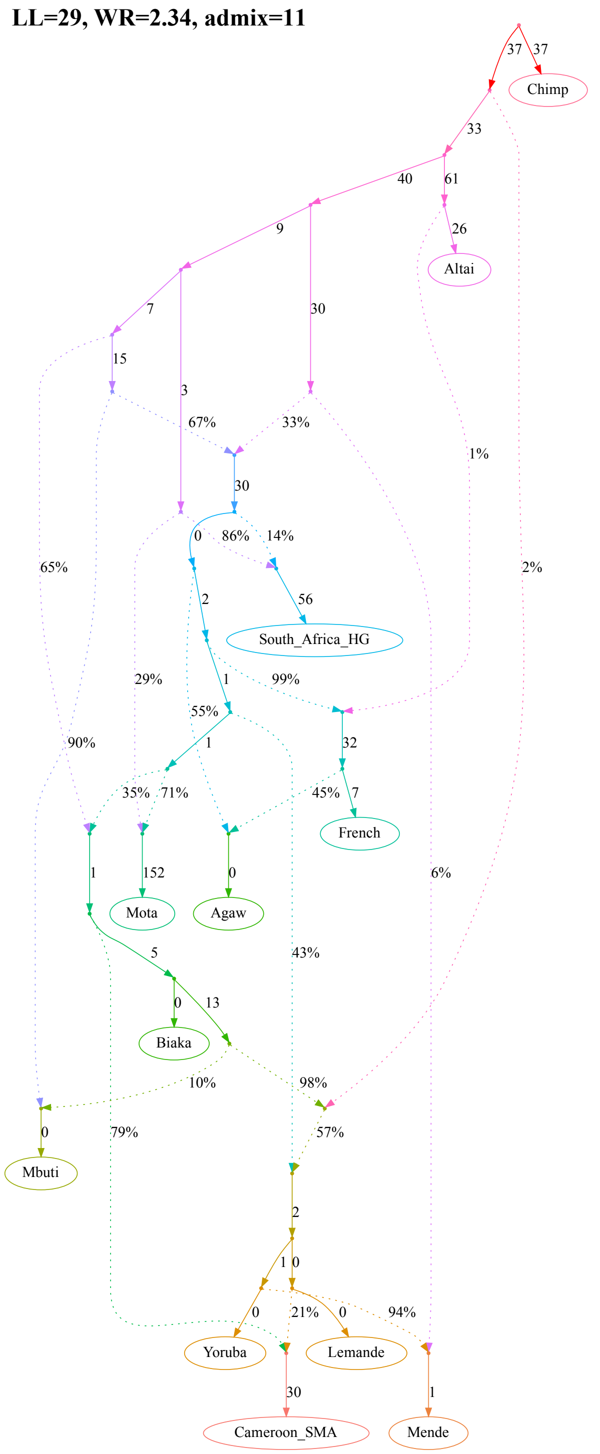

**support for claim 1** partial<br>**support for claim 2** partial<br>**support for claim 3** partial<br>**support for claim 4** partial	**support for claim 1** partial<br>**support for claim 2** partial<br>**support for claim 3** partial<br>**support for claim 4** partial
The original set of SNPs and individuals and the corrected algorithm for calculating f_3-statistics (211,738 variable site with no missing data at the group level)	The original set of SNPs and individuals and the original algorithm for calculating f_3-statistics (363,131 variable site with no missing data at the group level)

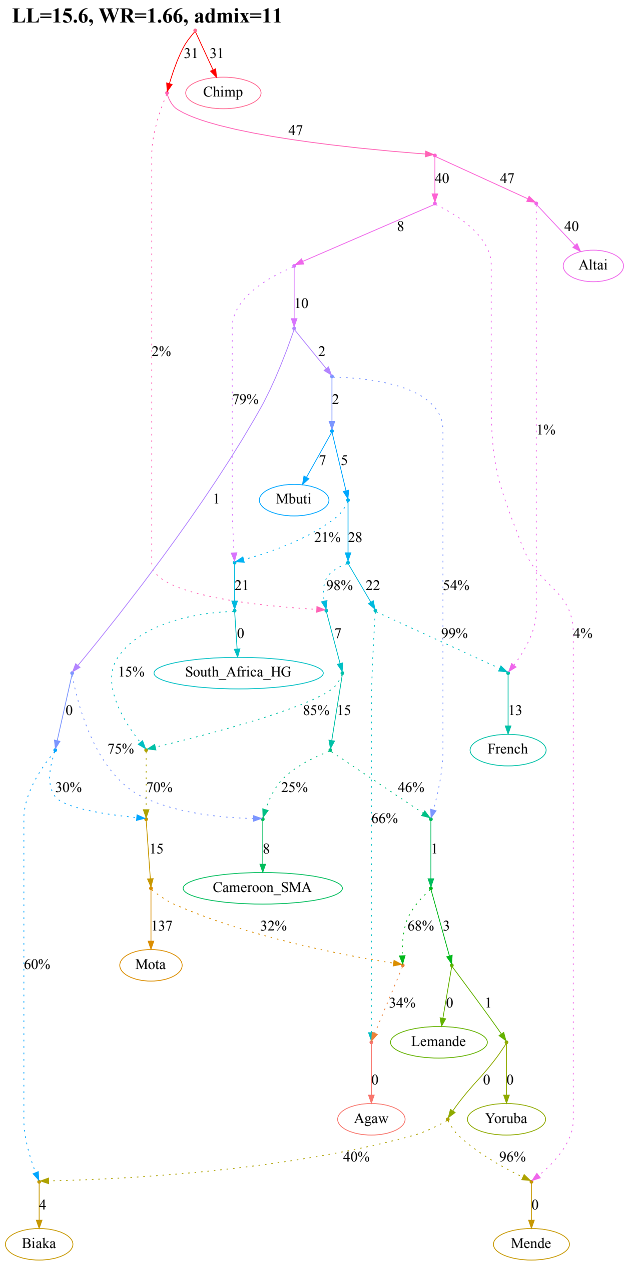

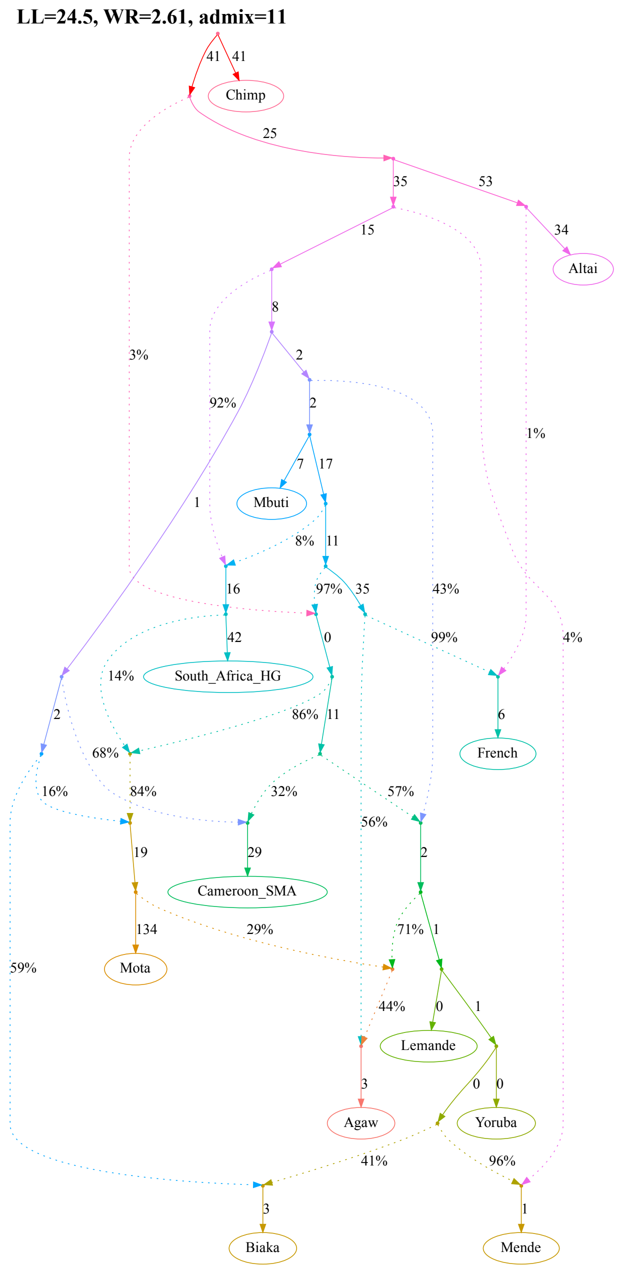

n, selected alternative models fitting nominally better than the published one

**support for claim 1** partial<br>**support for claim 2** partial<br>**support for claim 3** supported<br>**support for claim 4** partial	**support for claim 1** partial<br>**support for claim 2** partial<br>**support for claim 3** supported<br>**support for claim 4** partial
The original set of SNPs and individuals and the corrected algorithm for calculating *f*₃-statistics (211,738 variable site with no missing data at the group level)	The original set of SNPs and individuals and the original algorithm for calculating *f*₃-statistics (363,131 variable site with no missing data at the group level)

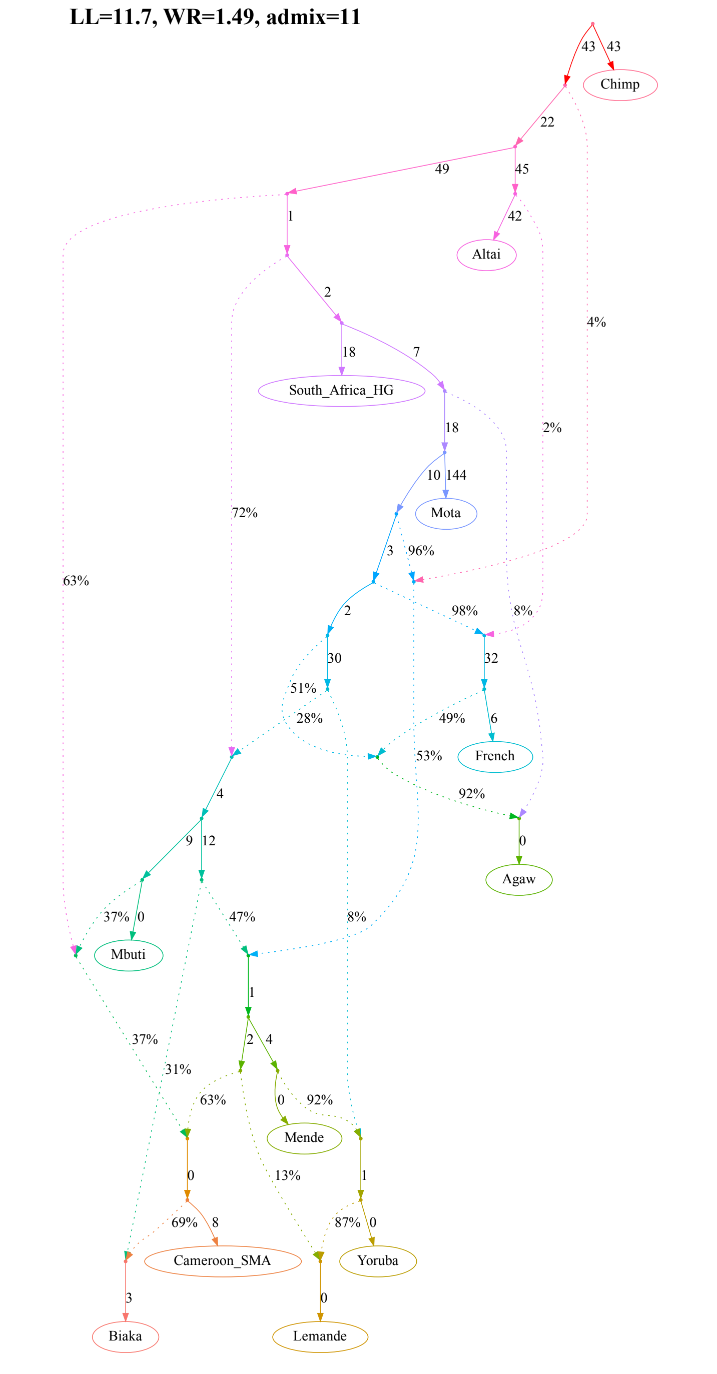

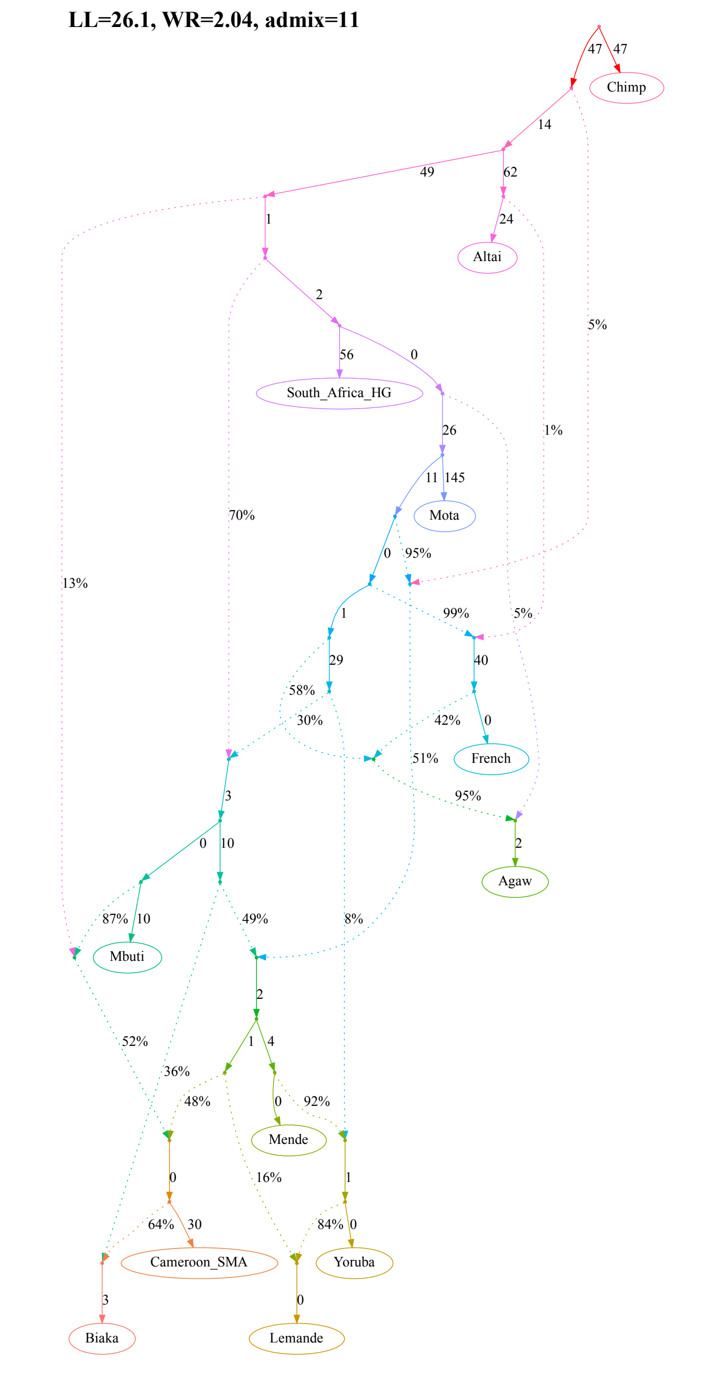

**support for claim 1**	supported
**support for claim 2**	partial
**support for claim 3**	partial
**support for claim 4**	partial

The original set of SNPs and individuals and the corrected algorithm for calculating f_3-statistics (211,738 variable site with no missing data at the group level)

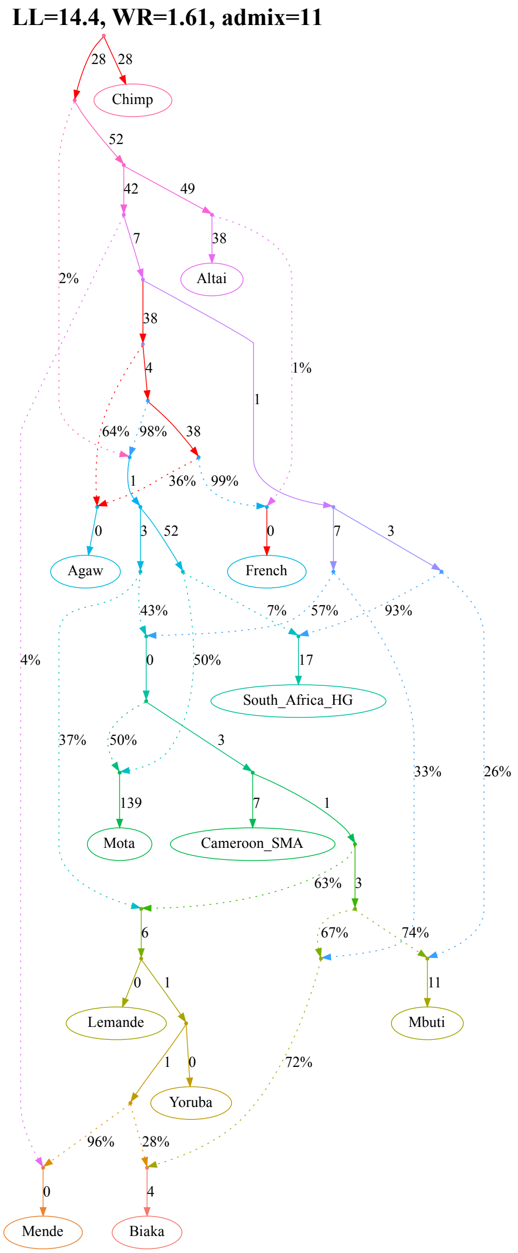

**support for claim 1**	supported
**support for claim 2**	partial
**support for claim 3**	partial
**support for claim 4**	partial

The original set of SNPs and individuals and the original algorithm for calculating f_3-statistics (363,131 variable site with no missing data at the group level)

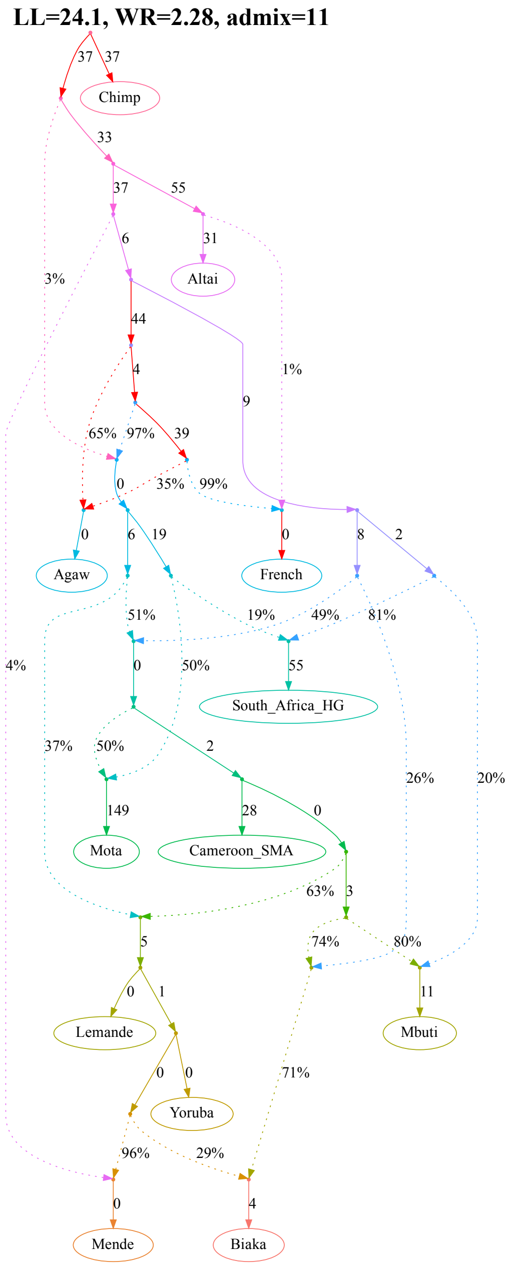

o, selected alternative models fitting nominally better than the published one

**support for claim 1** partial<br>**support for claim 2** partial<br>**support for claim 3** supported<br>**support for claim 4** partial	**support for claim 1** partial<br>**support for claim 2** partial<br>**support for claim 3** supported<br>**support for claim 4** partial
The original set of SNPs and individuals and the corrected algorithm for calculating f_3-statistics (211,738 variable site with no missing data at the group level)	The original set of SNPs and individuals and the original algorithm for calculating f_3-statistics (363,131 variable site with no missing data at the group level)

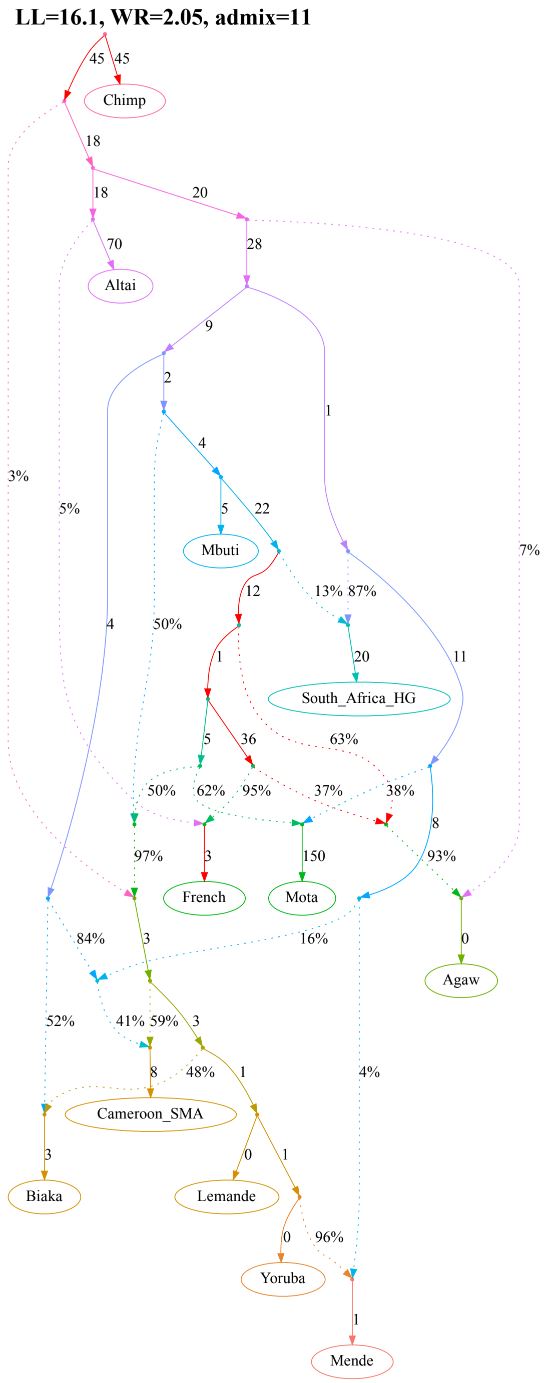

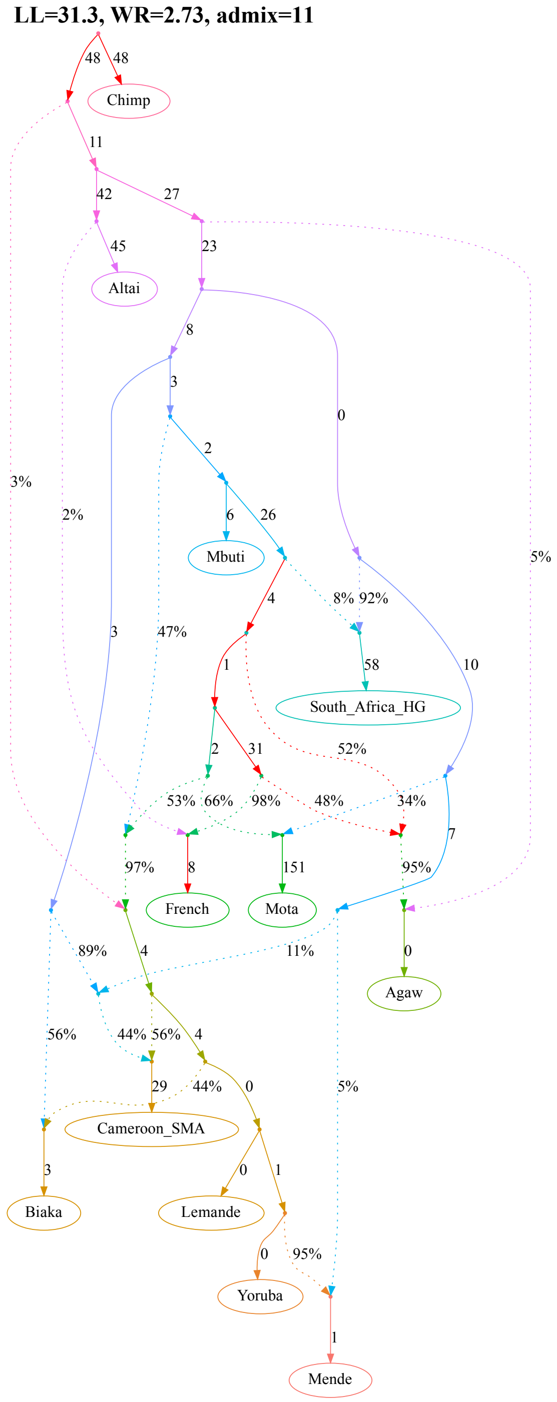

<table>
<tr>
<td>

**support for claim 1**	supported
**support for claim 2**	partial
**support for claim 3**	supported
**support for claim 4**	partial

</td>
<td>

**support for claim 1**	supported
**support for claim 2**	partial
**support for claim 3**	supported
**support for claim 4**	partial

</td>
</tr>
<tr>
<td>The original set of SNPs and individuals and the corrected algorithm for calculating f_3-statistics (211,738 variable site with no missing data at the group level)</td>
<td>The original set of SNPs and individuals and the original algorithm for calculating f_3-statistics (363,131 variable site with no missing data at the group level)</td>
</tr>
<tr>
<td>
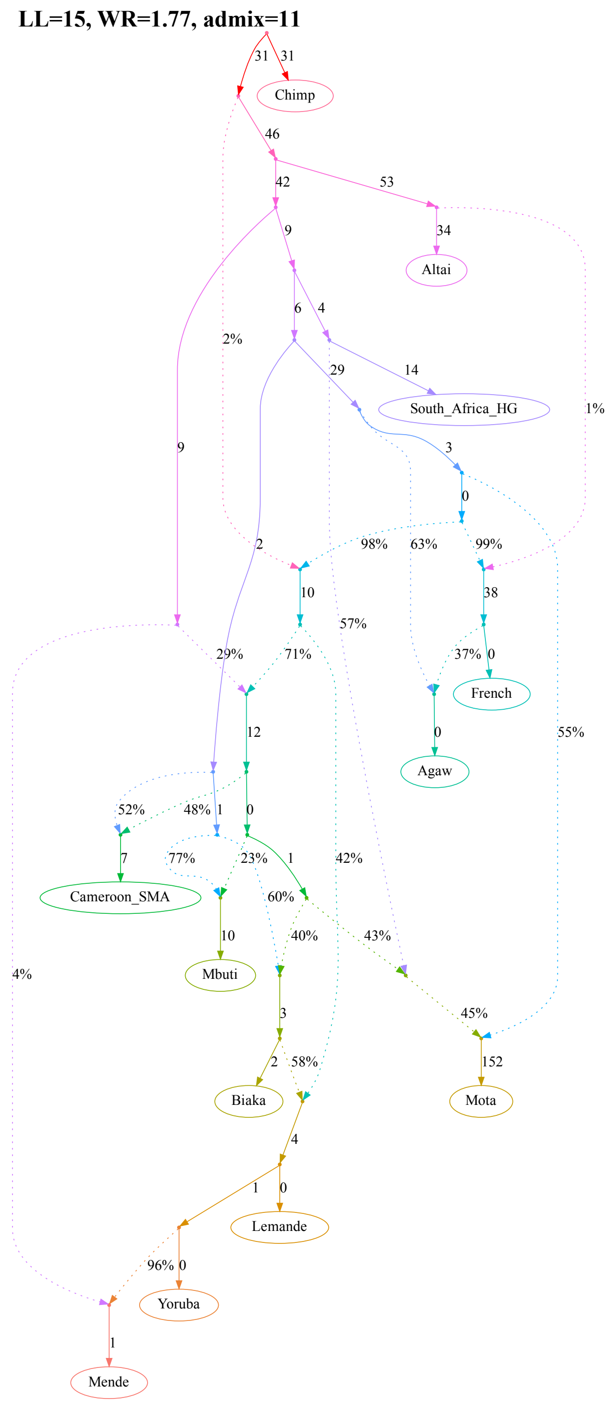

</td>
<td>
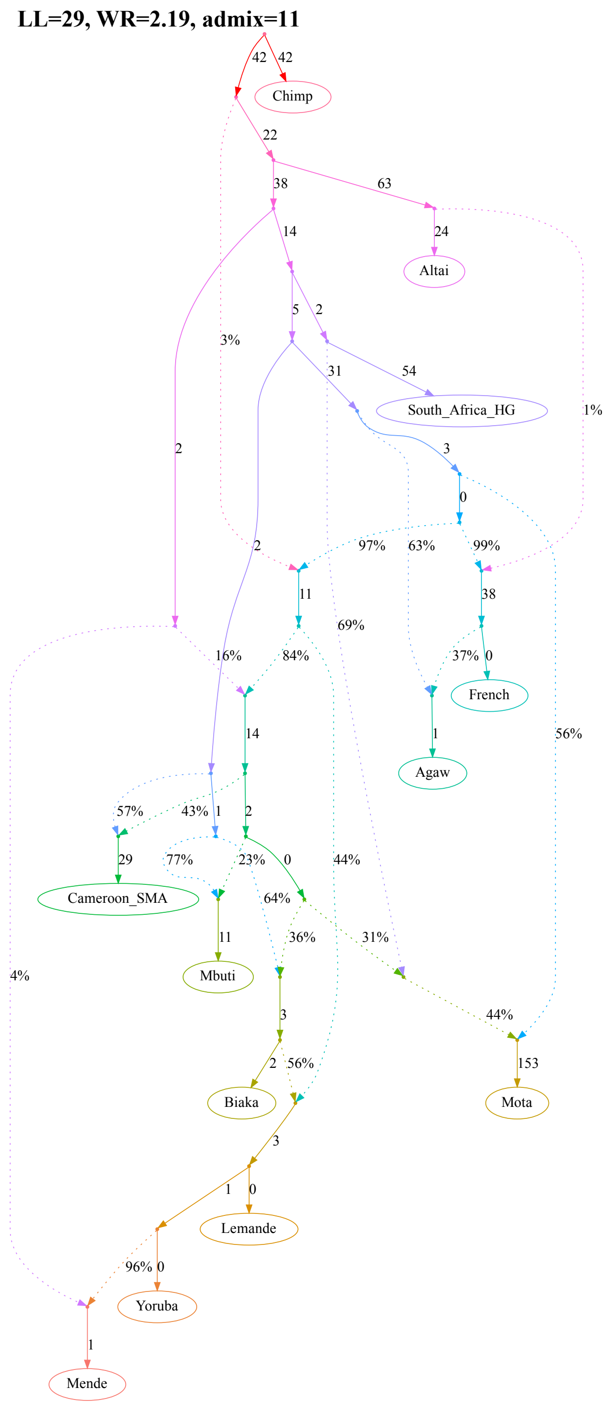

</td>
</tr>
</table>

p, selected alternative models fitting nominally better than the published one

<table>
<tr>
<td>support for claim 1 supported
support for claim 2 partial
support for claim 3 supported
support for claim 4 partial</td>
<td>support for claim 1 supported
support for claim 2 partial
support for claim 3 supported
support for claim 4 partial</td>
</tr>
<tr>
<td>The original set of SNPs and individuals and the corrected algorithm for calculating f₃-statistics (211,738 variable site with no missing data at the group level)</td>
<td>The original set of SNPs and individuals and the original algorithm for calculating f₃-statistics (363,131 variable site with no missing data at the group level)</td>
</tr>
<tr>
<td>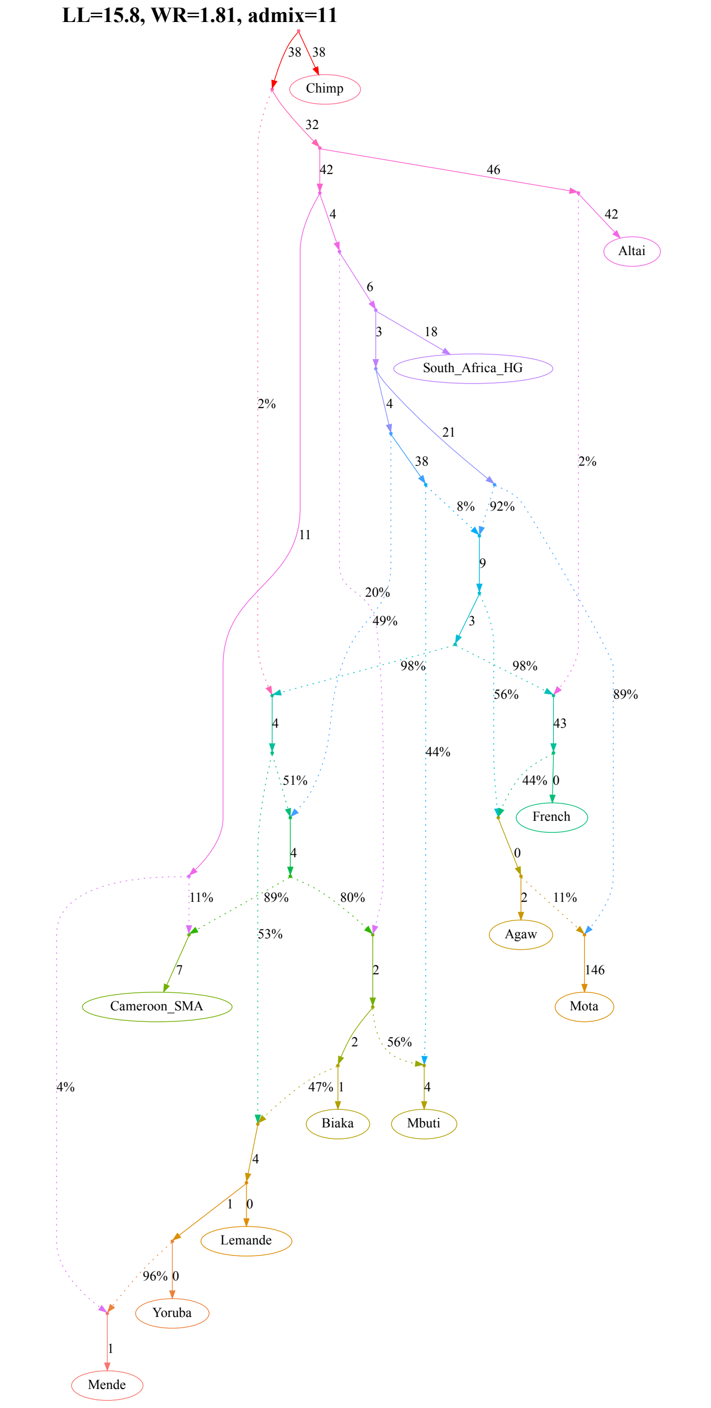
</td>
<td>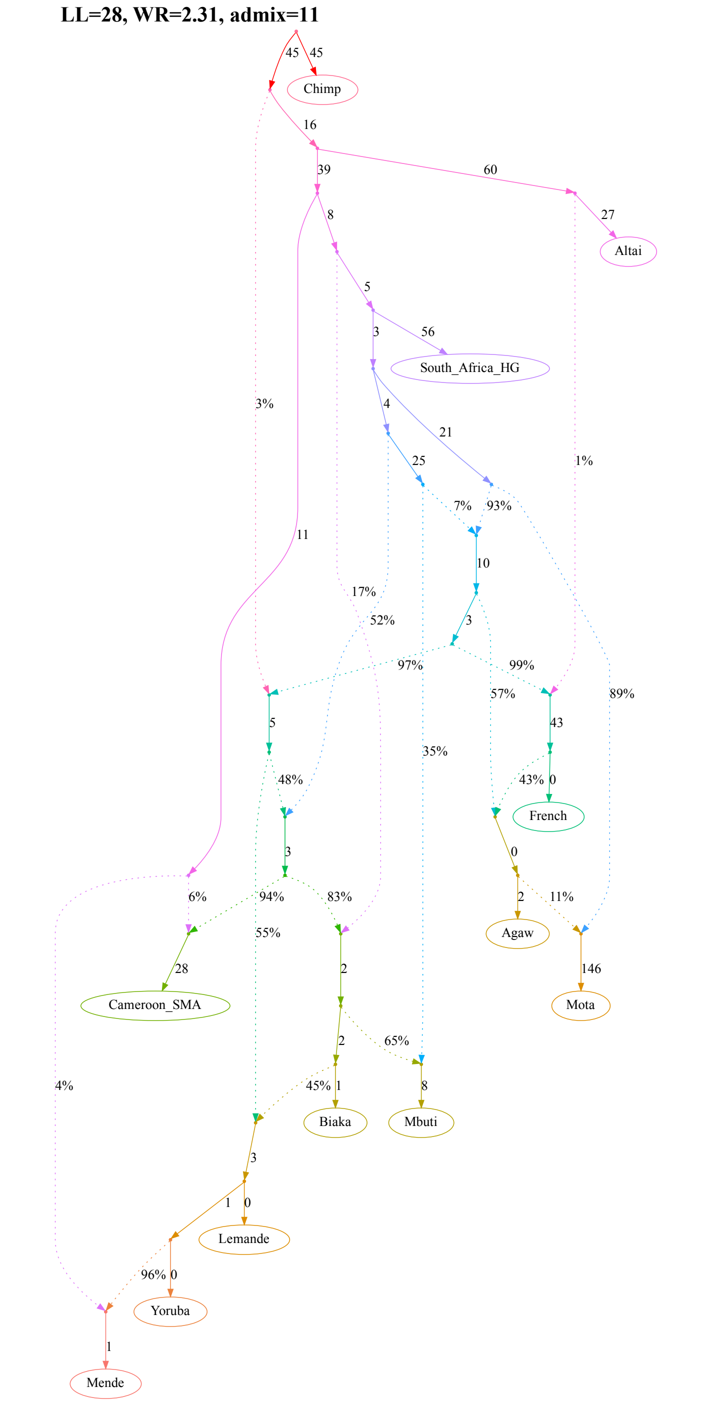
</td>
</tr>
</table>

**support for claim 1**	supported
**support for claim 2**	partial
**support for claim 3**	supported
**support for claim 4**	partial

The original set of SNPs and individuals and the corrected algorithm for calculating f_3-statistics (211,738 variable site with no missing data at the group level)

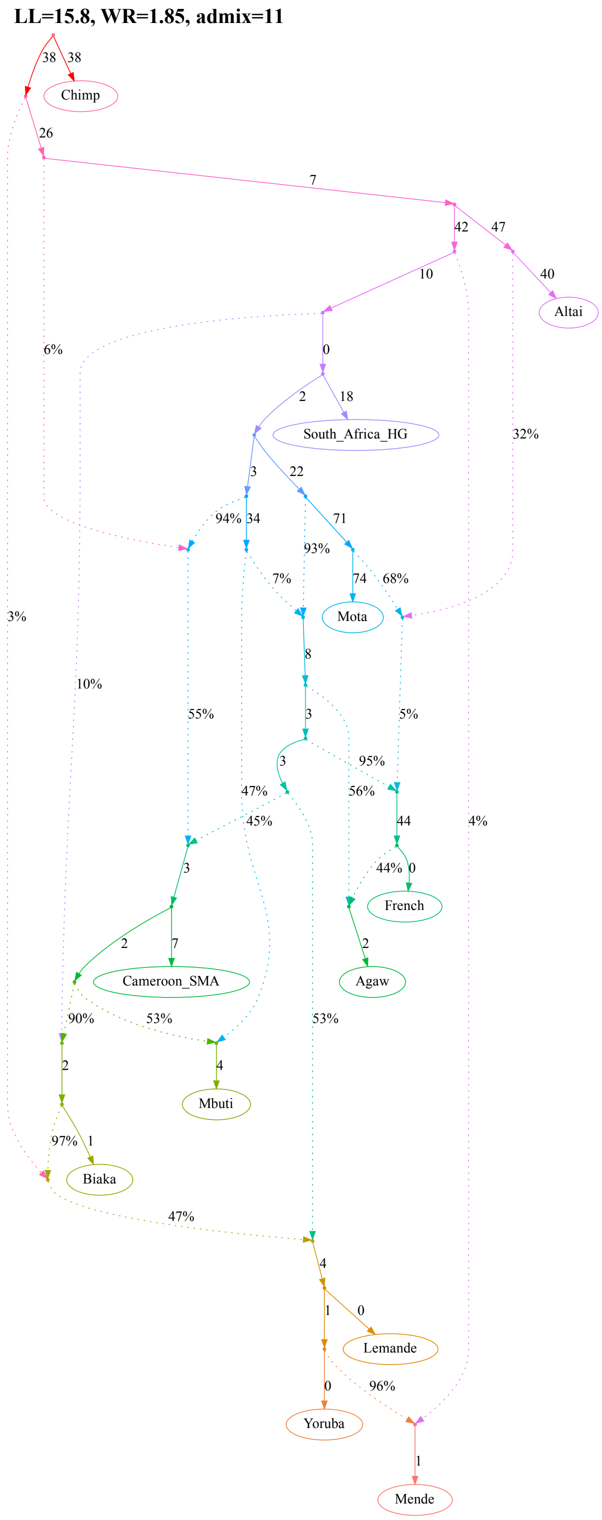

**support for claim 1**	supported
**support for claim 2**	partial
**support for claim 3**	supported
**support for claim 4**	partial

The original set of SNPs and individuals and the original algorithm for calculating f_3-statistics (363,131 variable site with no missing data at the group level)

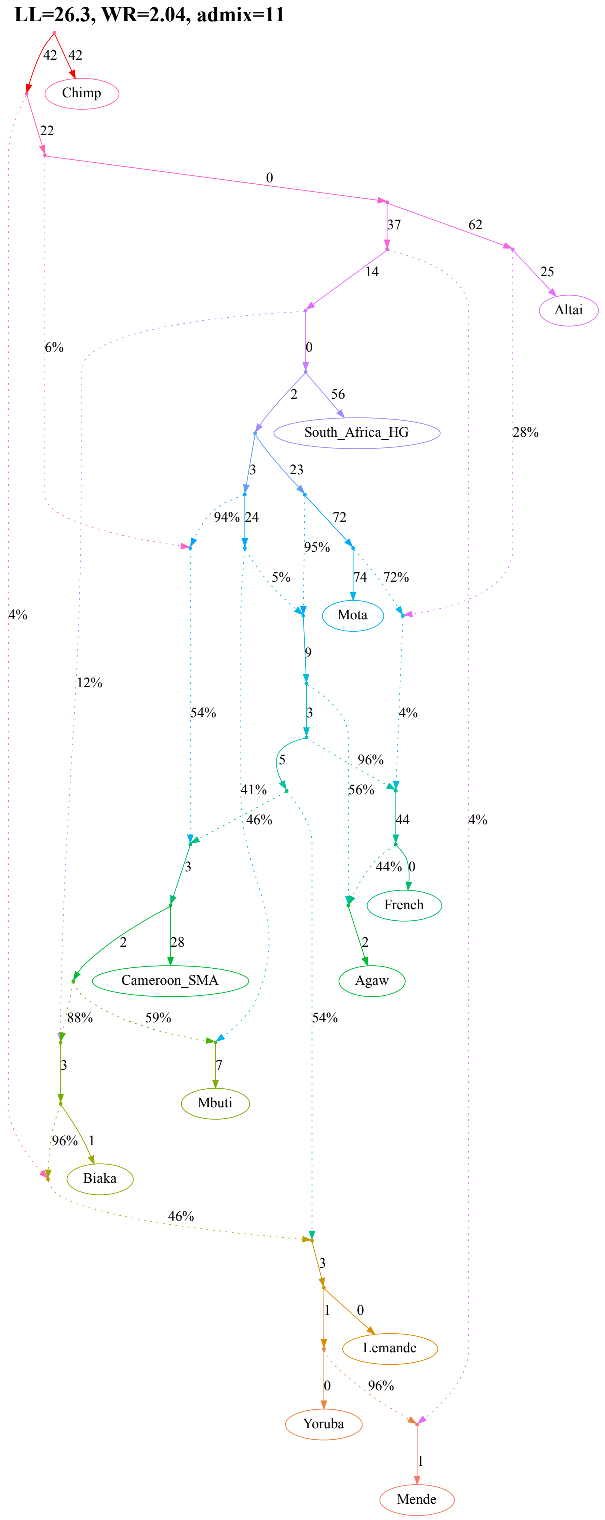

q, selected alternative models fitting nominally better than the published one

**support for claim 1** supported<br>**support for claim 2** partial<br>**support for claim 3** supported<br>**support for claim 4** supported	**support for claim 1** supported<br>**support for claim 2** partial<br>**support for claim 3** supported<br>**support for claim 4** supported
The original set of SNPs and individuals and the corrected algorithm for calculating *f*$_3$-statistics (211,738 variable site with no missing data at the group level)	The original set of SNPs and individuals and the original algorithm for calculating *f*$_3$-statistics (363,131 variable site with no missing data at the group level)
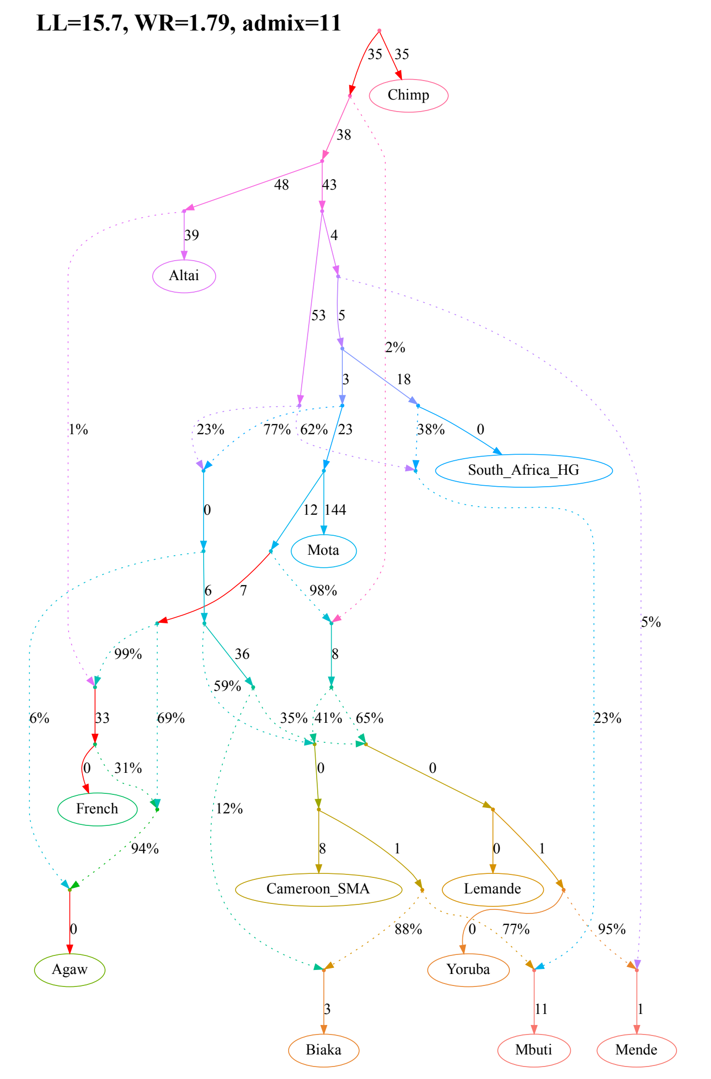	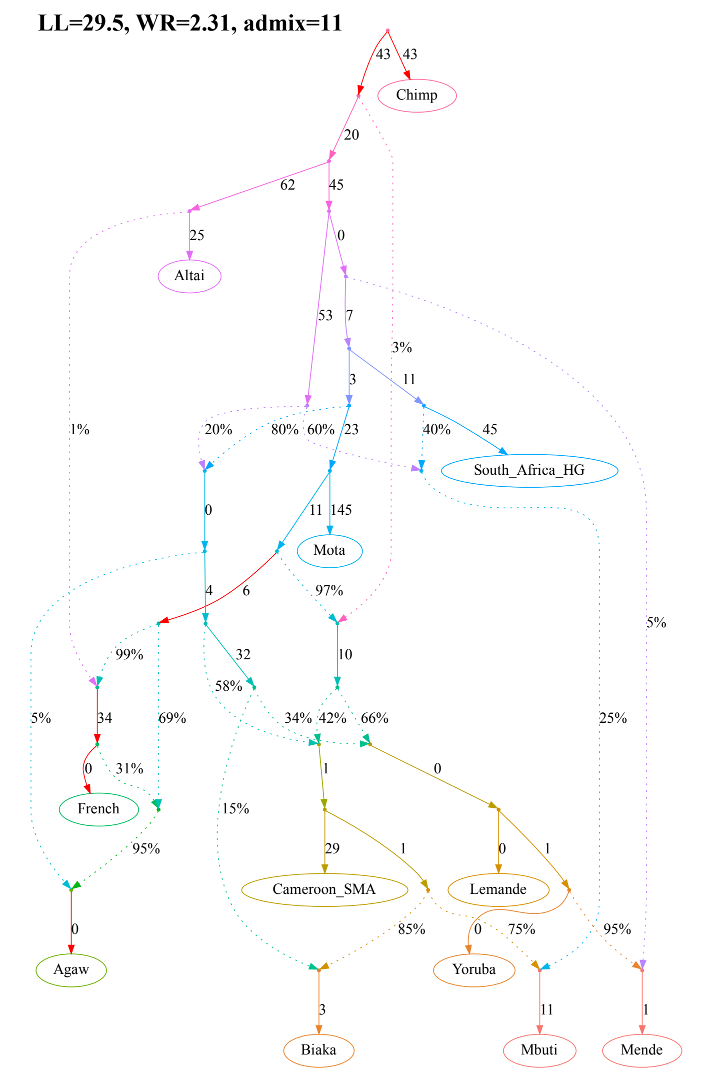

**support for claim 1** supported<br>**support for claim 2** supported<br>**support for claim 3** supported<br>**support for claim 4** supported	**support for claim 1** supported<br>**support for claim 2** supported<br>**support for claim 3** supported<br>**support for claim 4** supported
The original set of SNPs and individuals and the corrected algorithm for calculating f_3-statistics (211,738 variable site with no missing data at the group level)	The original set of SNPs and individuals and the original algorithm for calculating f_3-statistics (363,131 variable site with no missing data at the group level)

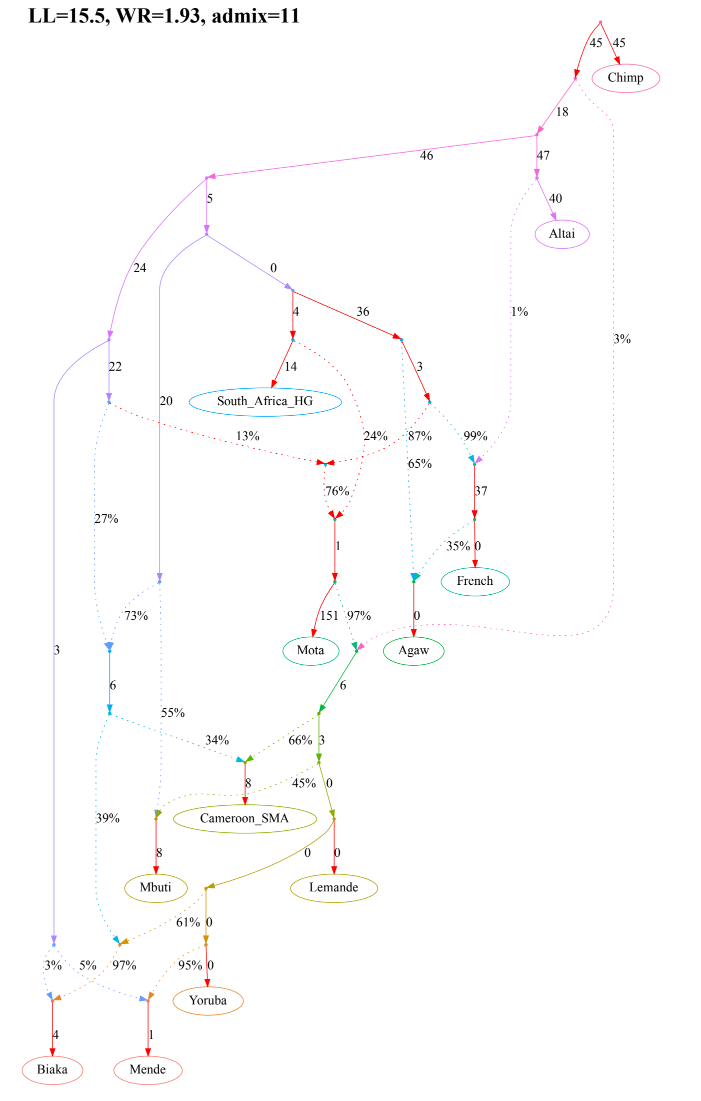

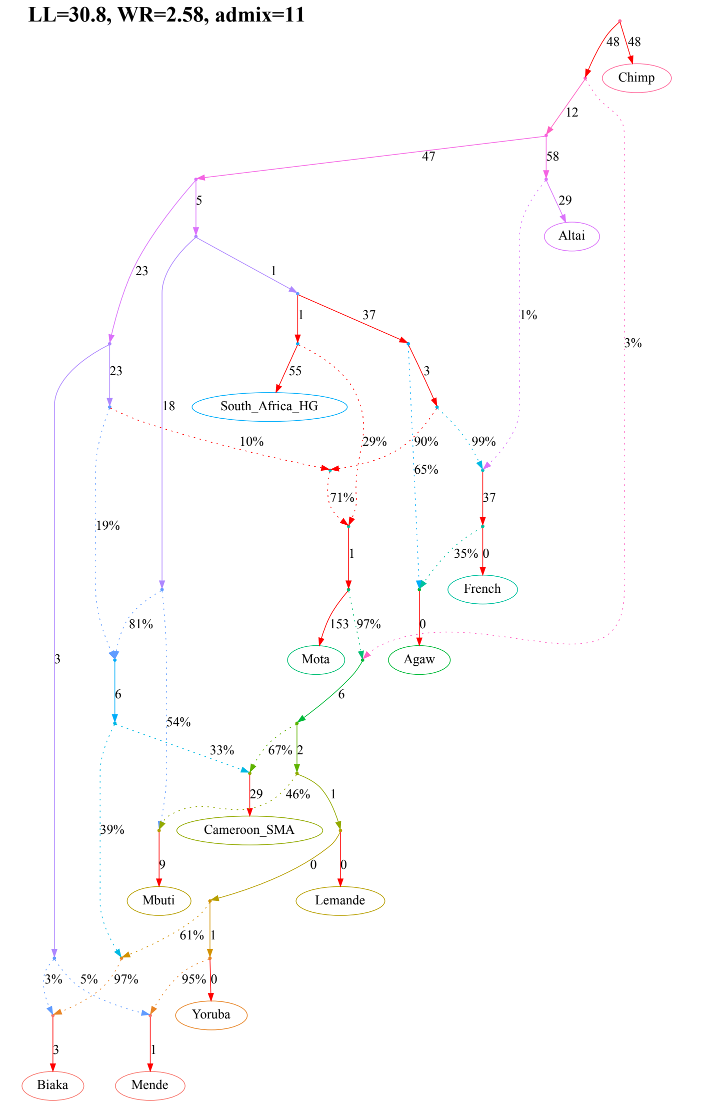

Supplement: Figure 4—source data 1. — The graphs were also re-fitted using the original algorithm for calculating f-statistics. Model parameters (graph edges) that were inferred to be unidentifiable are plotted in red. (a, b) The published model and its simplified representation. Below we list selected claims by Lipson et al., 2020b relying on the admixture graph: (1) A lineage maximized in present-day West African groups (Lemande, Mende, and Yoruba) also contributed some ancestry to the ancient Shum Laka individual, and present-day Biaka and Mbuti; (2) another ancestry component in Shum Laka is a deep-branching lineage maximized in rainforest hunter–gatherers Biaka and Mbuti; (3) ‘super-archaic’ ancestry (i.e., diverging at the modern human/Neanderthal split point or deeper) contributed to Biaka, Shum Laka, Mbuti, Lemande, Mende, and Yoruba; (4) a ghost modern human lineage (or lineages) contributed to Agaw, Mota, Biaka, Shum Laka, Mbuti, Lemande, Mende, and Yoruba. (c-q) Selected alternative models fitting nominally better than the published one. [file elife-85492-fig4-data1.pdf]
