# Supplementary material for: On the limits of fitting complex models of population history to f-statistics
Source: eLife. 2023 Jun 29;12:e85492. doi: 10.7554/eLife.85492 (PMC10310323; doi:10.7554/eLife.85492)

Figure 4–source data 2. Published admixture graph from Wang *et al*. (2021) and alternative graphs found with *findGraphs* (12 populations, 8 admixture events) using the updated algorithm for calculating *f*-statistics. The graphs were also re-fitted using the original algorithm for calculating *f*-statistics. Model parameters (graph edges) that were inferred to be unidentifiable are plotted in red.

a, published model

no. of East Asian groups with Onge-related ancestry	6
no. of East Asian groups with European-related ancestry	1
direction of gene flow China_Island_EN > Taiwan_IA	forward
Mongolia_East is basal with respect to China_Upper_YR and Nepal_Chokhopani	yes
Taiwan_IA is basal with respect to Mongolia_East and Nepal_Chokhopani	yes

The original set of SNPs and individuals and the corrected algorithm for calculating f_3-statistics (203,753 variable site with no missing data at the group level)

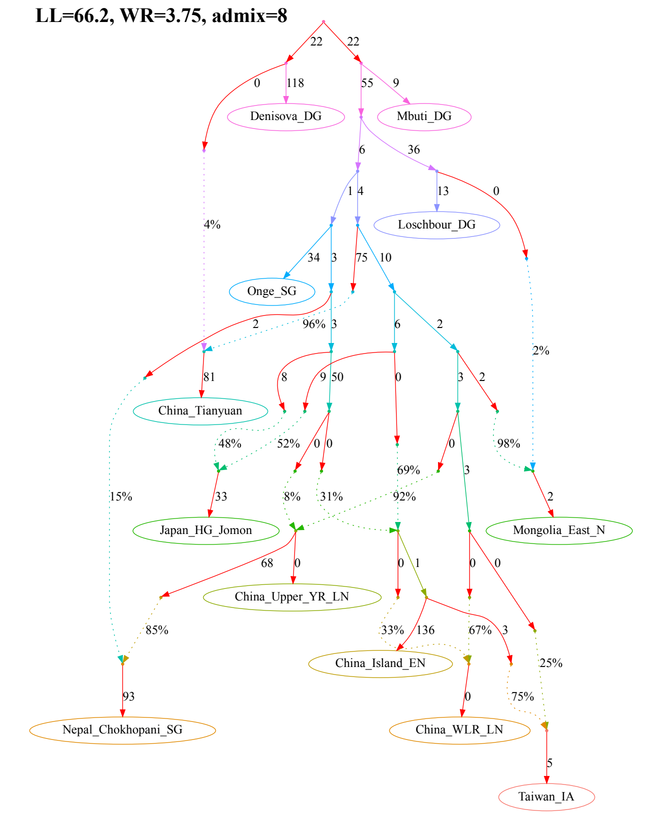

no. of East Asian groups with Onge-related ancestry	6
no. of East Asian groups with European-related ancestry	1
direction of gene flow China_Island_EN > Taiwan_IA	forward
Mongolia_East is basal with respect to China_Upper_YR and Nepal_Chokhopani	yes
Taiwan_IA is basal with respect to Mongolia_East and Nepal_Chokhopani	yes

The original set of SNPs and individuals and the original algorithm for calculating f_3-statistics (496,233 variable site with no missing data at the group level)

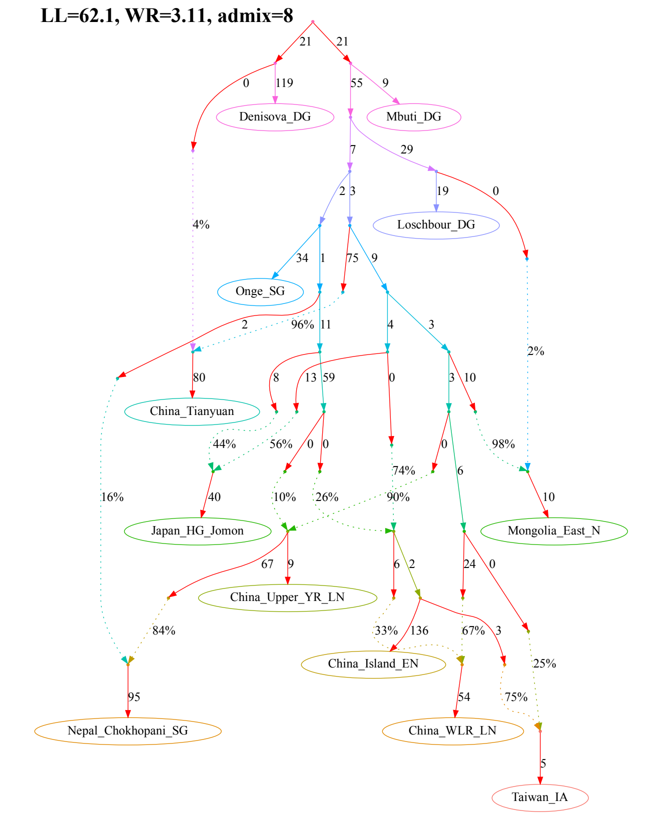

b, published model, simplified

no. of East Asian groups with Onge-related ancestry	6
no. of East Asian groups with European-related ancestry	1
direction of gene flow China_Island_EN > Taiwan_IA	forward
Mongolia_East is basal with respect to China_Upper_YR and Nepal_Chokhopani	yes
Taiwan_IA is basal with respect to Mongolia_East and Nepal_Chokhopani	yes

The original set of SNPs and individuals and the corrected algorithm for calculating *f*3-statistics (203,753 variable site with no missing data at the group level)

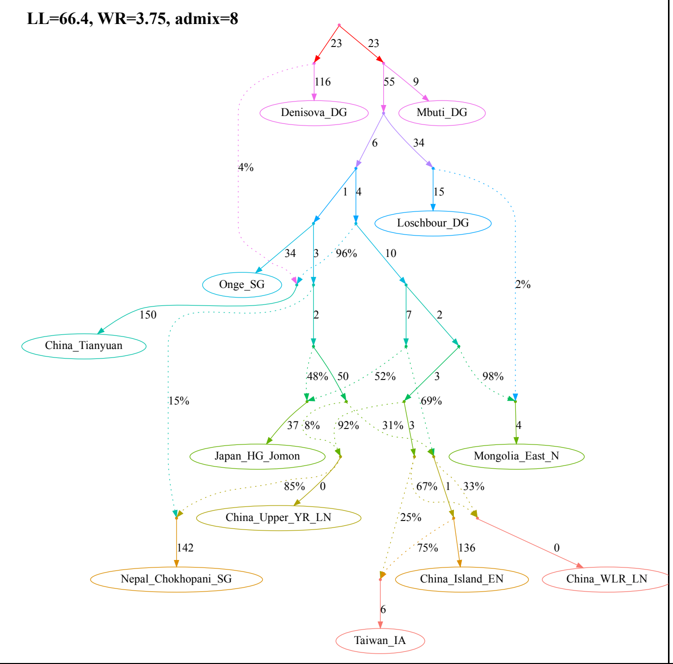

no. of East Asian groups with Onge-related ancestry	6
no. of East Asian groups with European-related ancestry	1
direction of gene flow China_Island_EN > Taiwan_IA	forward
Mongolia_East is basal with respect to China_Upper_YR and Nepal_Chokhopani	yes
Taiwan_IA is basal with respect to Mongolia_East and Nepal_Chokhopani	yes

The original set of SNPs and individuals and the original algorithm for calculating *f*3-statistics (496,233 variable site with no missing data at the group level)

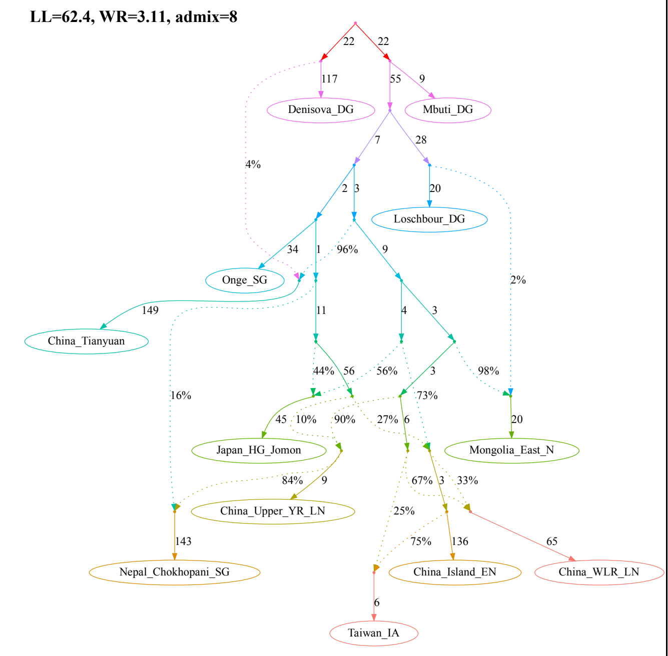

A claim by Wang *et al*. 2021 relying on the admixture graph:

There is Onge-related admixture in the following lineages: Jomon (Japan_HG_Jomon), Tibetan (Nepal_Chokhopani_SG), Upper Yellow River Late Neolithic (China_Upper_YR_LN), West Liao River Late Neolithic (China_WLR_LN), Taiwan Iron Age (Taiwan_IA), China Island Early Neolithic (Liangdao, China_Island_EN).

c, alternative models fitting significantly better than the published one

no. of East Asian groups with Onge-related ancestry 1<br>no. of East Asian groups with European-related ancestry 8<br>direction of gene flow China_Island_EN > Taiwan_IA forward<br>Mongolia_East is basal with respect to China_Upper_YR and Nepal_Chokhopani yes<br>Taiwan_IA is basal with respect to Mongolia_East and Nepal_Chokhopani yes	no. of East Asian groups with Onge-related ancestry 1<br>no. of East Asian groups with European-related ancestry 8<br>direction of gene flow China_Island_EN > Taiwan_IA forward<br>Mongolia_East is basal with respect to China_Upper_YR and Nepal_Chokhopani yes<br>Taiwan_IA is basal with respect to Mongolia_East and Nepal_Chokhopani yes
The original set of SNPs and individuals and the corrected algorithm for calculating f_3-statistics (203,753 variable site with no missing data at the group level)	The original set of SNPs and individuals and the original algorithm for calculating f_3-statistics (496,233 variable site with no missing data at the group level)
**LL=35.4, WR=2.59, admix=8**<br>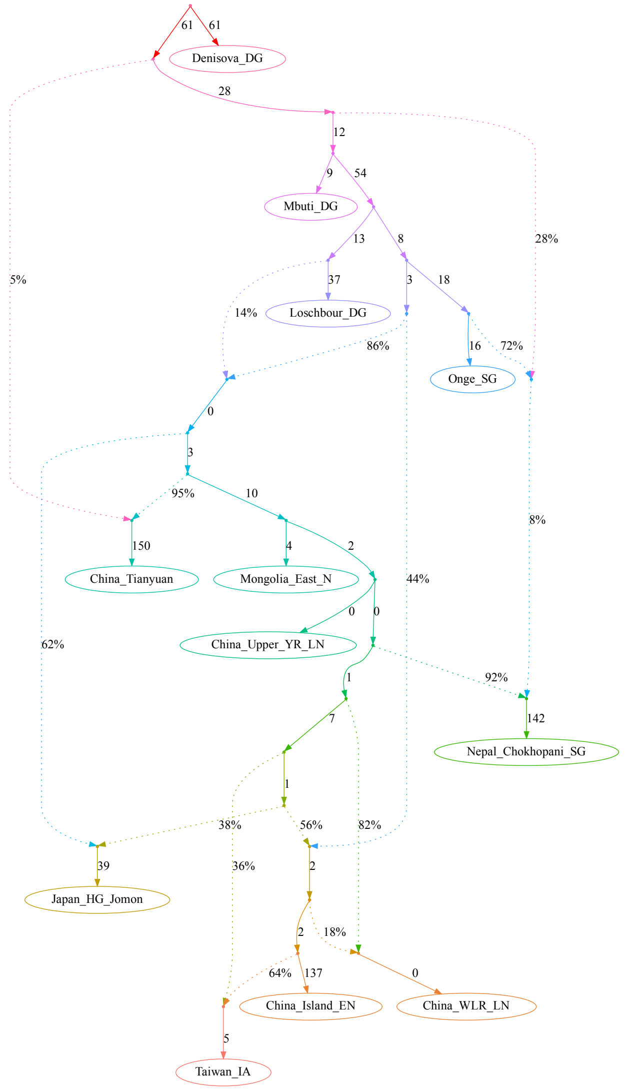	**LL=40.5, WR=3.26, admix=8**<br>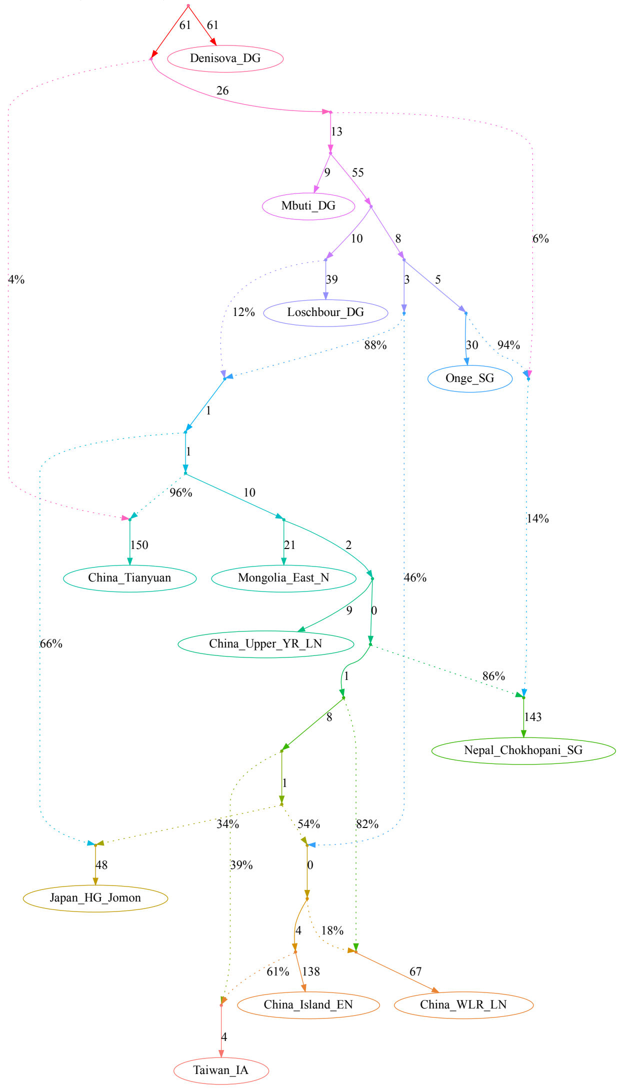

no. of East Asian groups with Onge-related ancestry: 2<br>no. of East Asian groups with European-related ancestry: 8<br>direction of gene flow China_Island_EN > Taiwan_IA: forward<br>Mongolia_East is basal with respect to China_Upper_YR and Nepal_Chokhopani: yes<br>Taiwan_IA is basal with respect to Mongolia_East and Nepal_Chokhopani: yes	no. of East Asian groups with Onge-related ancestry: 2<br>no. of East Asian groups with European-related ancestry: 8<br>direction of gene flow China_Island_EN > Taiwan_IA: forward<br>Mongolia_East is basal with respect to China_Upper_YR and Nepal_Chokhopani: yes<br>Taiwan_IA is basal with respect to Mongolia_East and Nepal_Chokhopani: yes
The original set of SNPs and individuals and the corrected algorithm for calculating f_3-statistics (203,753 variable site with no missing data at the group level)	The original set of SNPs and individuals and the original algorithm for calculating f_3-statistics (496,233 variable site with no missing data at the group level)
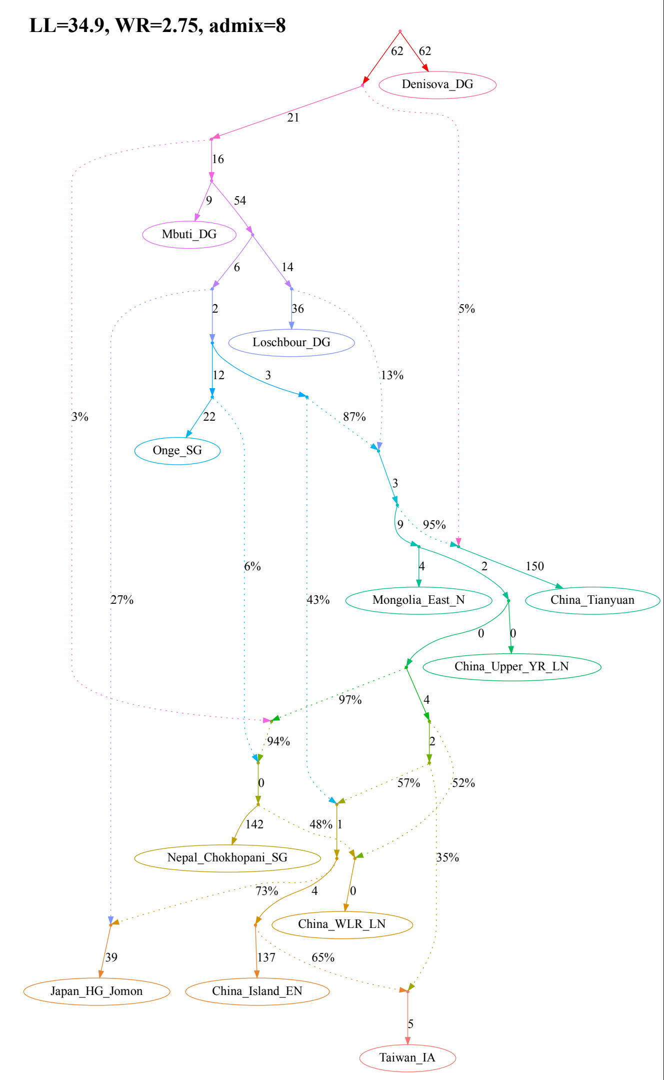	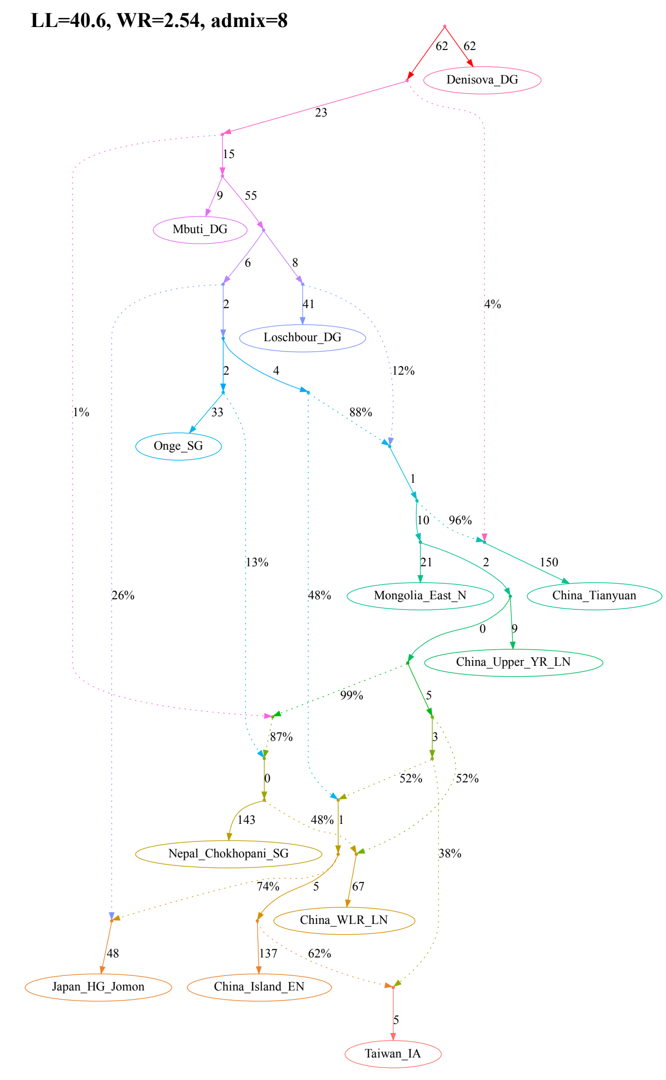

d, alternative models fitting significantly better than the published one

	Left model	Right model
no. of East Asian groups with Onge-related ancestry	1	1
no. of East Asian groups with European-related ancestry	8	8
direction of gene flow China_Island_EN > Taiwan_IA	no gene flow	no gene flow
Mongolia_East is basal with respect to China_Upper_YR and Nepal_Chokhopani	yes	yes
Taiwan_IA is basal with respect to Mongolia_East and Nepal_Chokhopani	yes	yes
	The original set of SNPs and individuals and the corrected algorithm for calculating f_3-statistics (203,753 variable site with no missing data at the group level)	The original set of SNPs and individuals and the original algorithm for calculating f_3-statistics (496,233 variable site with no missing data at the group level)

LL=34.3, WR=2.63, admix=8

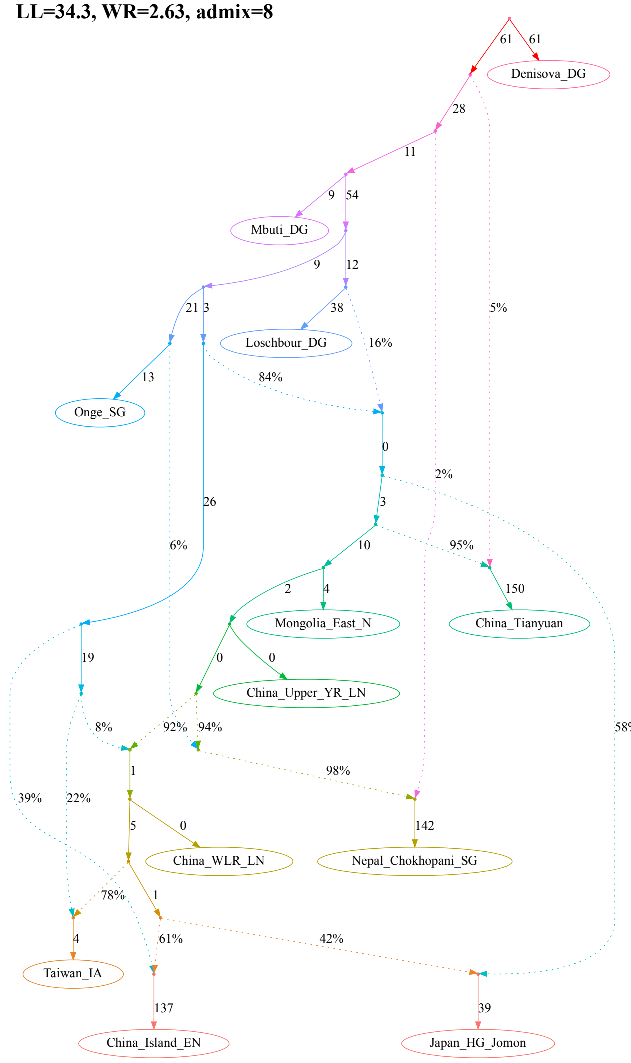

LL=39, WR=3.28, admix=8

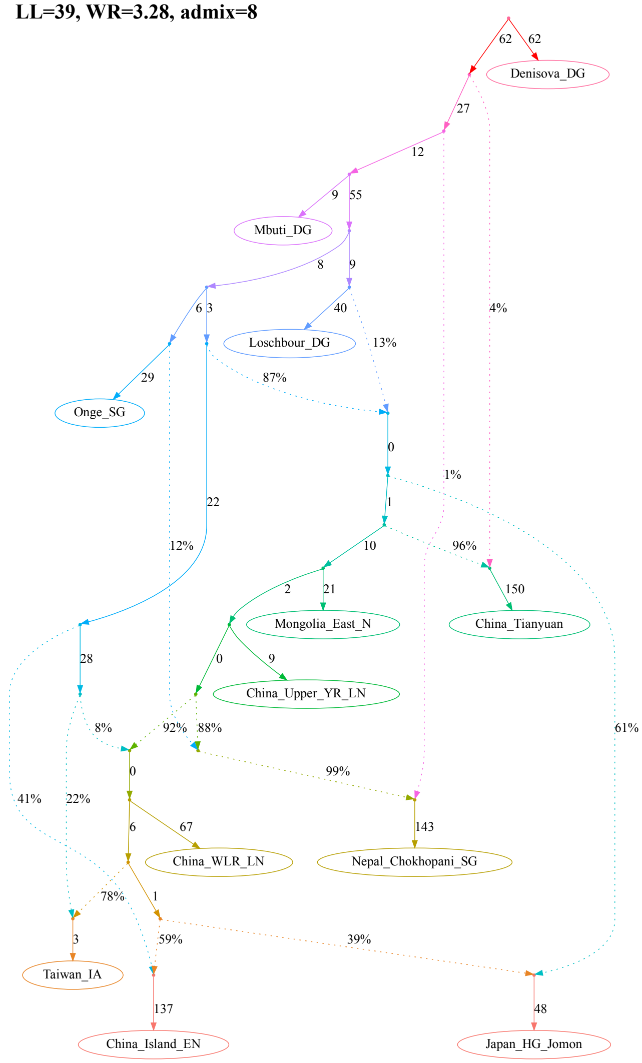

	The original set of SNPs and individuals and the corrected algorithm for calculating f_3-statistics (203,753 variable site with no missing data at the group level)	The original set of SNPs and individuals and the original algorithm for calculating f_3-statistics (496,233 variable site with no missing data at the group level)
no. of East Asian groups with Onge-related ancestry	1	1
no. of East Asian groups with European-related ancestry	8	8
direction of gene flow China_Island_EN > Taiwan_IA	reverse	reverse
Mongolia_East is basal with respect to China_Upper_YR and Nepal_Chokhopani	yes	yes
Taiwan_IA is basal with respect to Mongolia_East and Nepal_Chokhopani	yes	yes

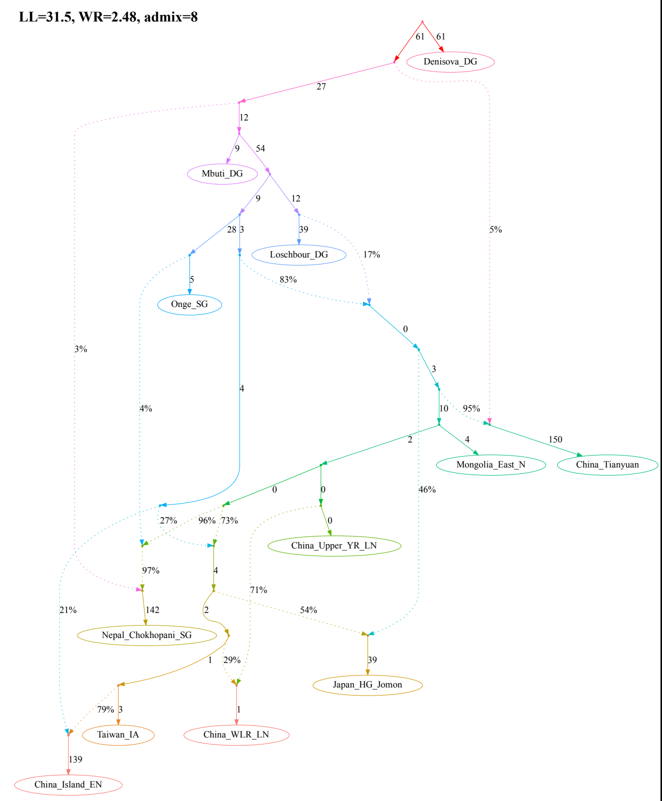

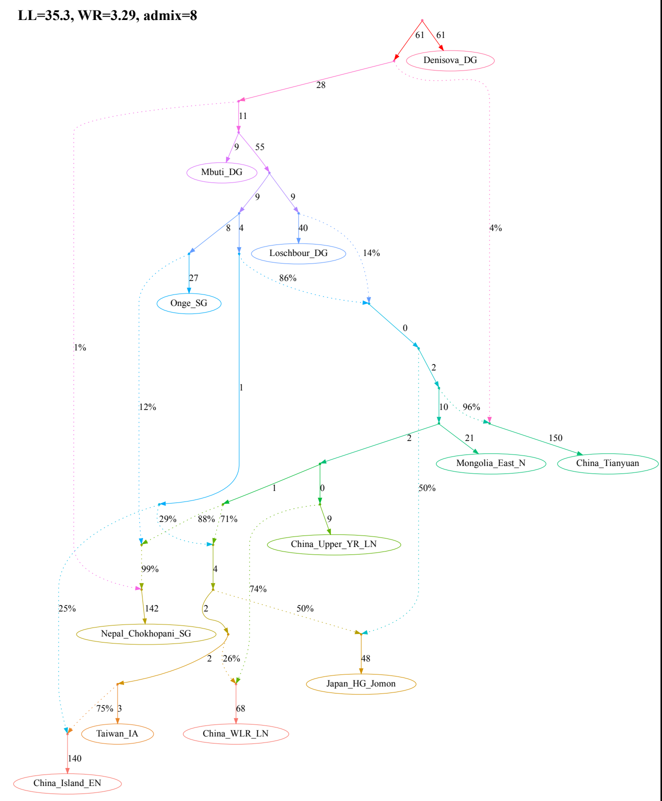

e, alternative models fitting significantly better than the published one

no. of East Asian groups with Onge-related ancestry	1
no. of East Asian groups with European-related ancestry	8
direction of gene flow China_Island_EN > Taiwan_IA	reverse
Mongolia_East is basal with respect to China_Upper_YR and Nepal_Chokhopani	yes
Taiwan_IA is basal with respect to Mongolia_East and Nepal_Chokhopani	yes

The original set of SNPs and individuals and the corrected algorithm for calculating f_3-statistics (203,753 variable site with no missing data at the group level)

LL=32.6, WR=2.49, admix=8

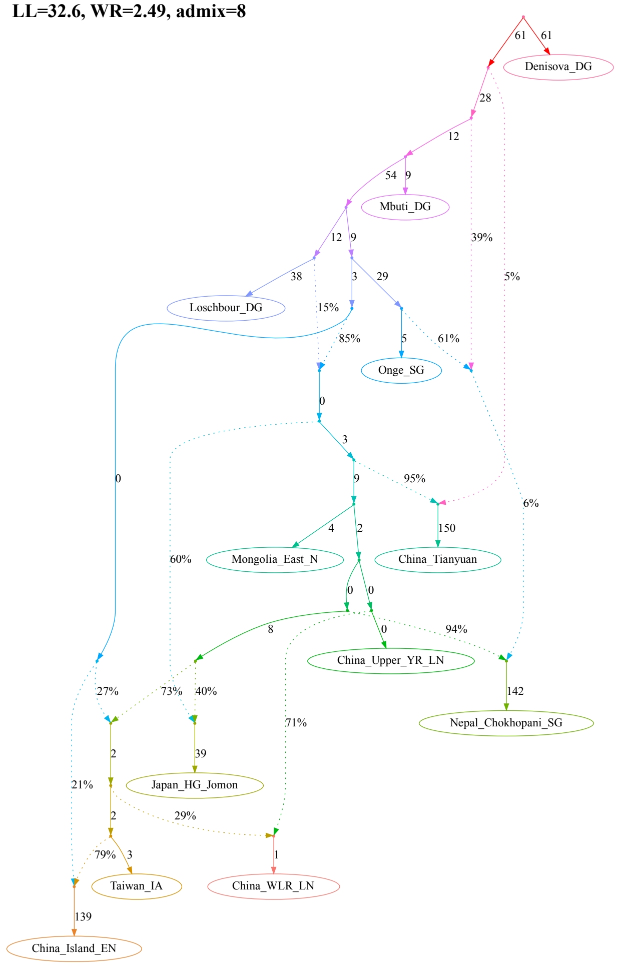

no. of East Asian groups with Onge-related ancestry	1
no. of East Asian groups with European-related ancestry	8
direction of gene flow China_Island_EN > Taiwan_IA	reverse
Mongolia_East is basal with respect to China_Upper_YR and Nepal_Chokhopani	yes
Taiwan_IA is basal with respect to Mongolia_East and Nepal_Chokhopani	yes

The original set of SNPs and individuals and the original algorithm for calculating f_3-statistics (496,233 variable site with no missing data at the group level)

LL=36, WR=3.23, admix=8

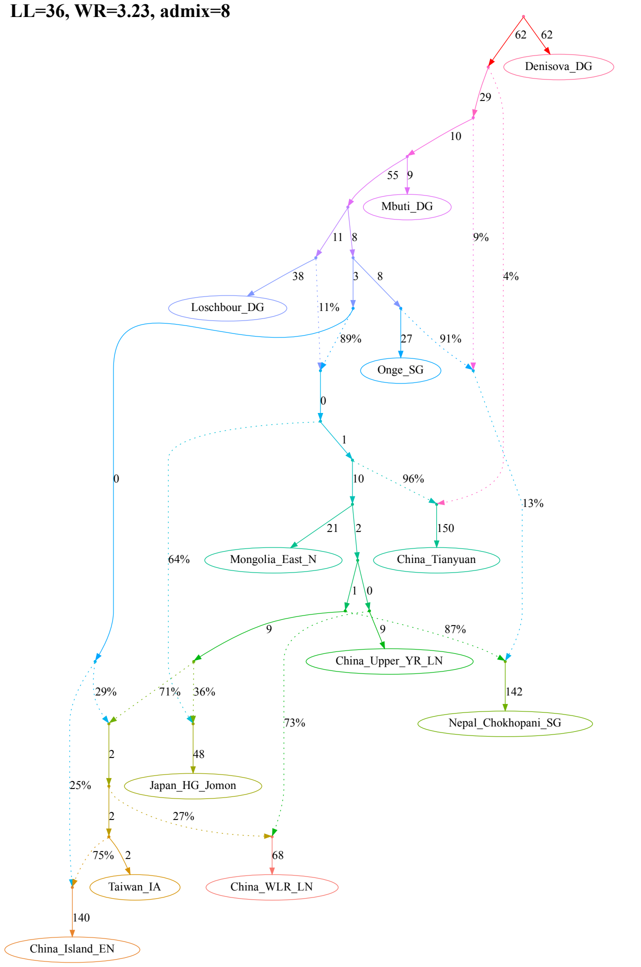

	The original set of SNPs and individuals and the corrected algorithm for calculating f_3-statistics (203,753 variable site with no missing data at the group level)	The original set of SNPs and individuals and the original algorithm for calculating f_3-statistics (496,233 variable site with no missing data at the group level)
no. of East Asian groups with Onge-related ancestry	1	1
no. of East Asian groups with European-related ancestry	8	8
direction of gene flow China_Island_EN > Taiwan_IA	reverse	reverse
Mongolia_East is basal with respect to China_Upper_YR and Nepal_Chokhopani	yes	yes
Taiwan_IA is basal with respect to Mongolia_East and Nepal_Chokhopani	yes	yes

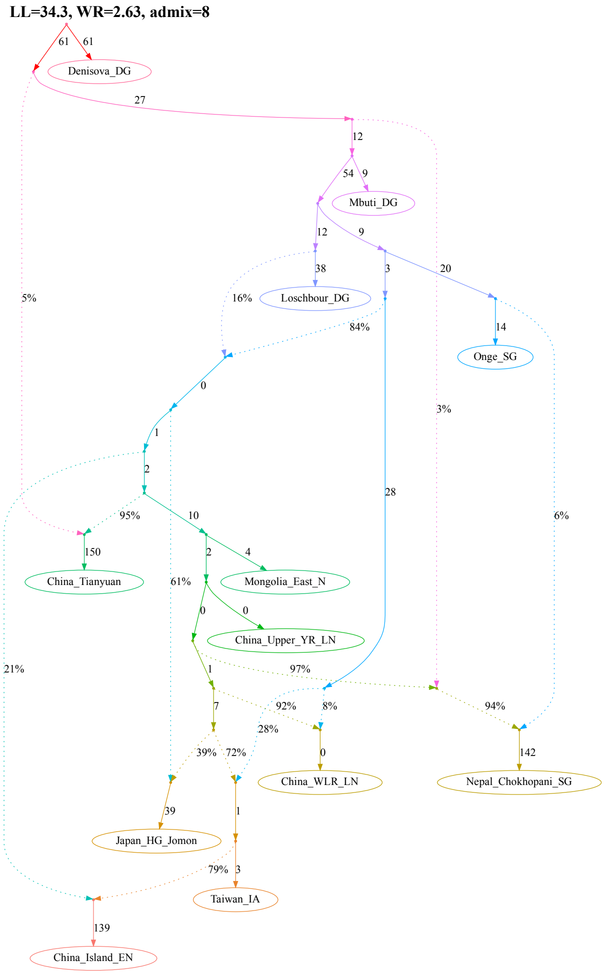

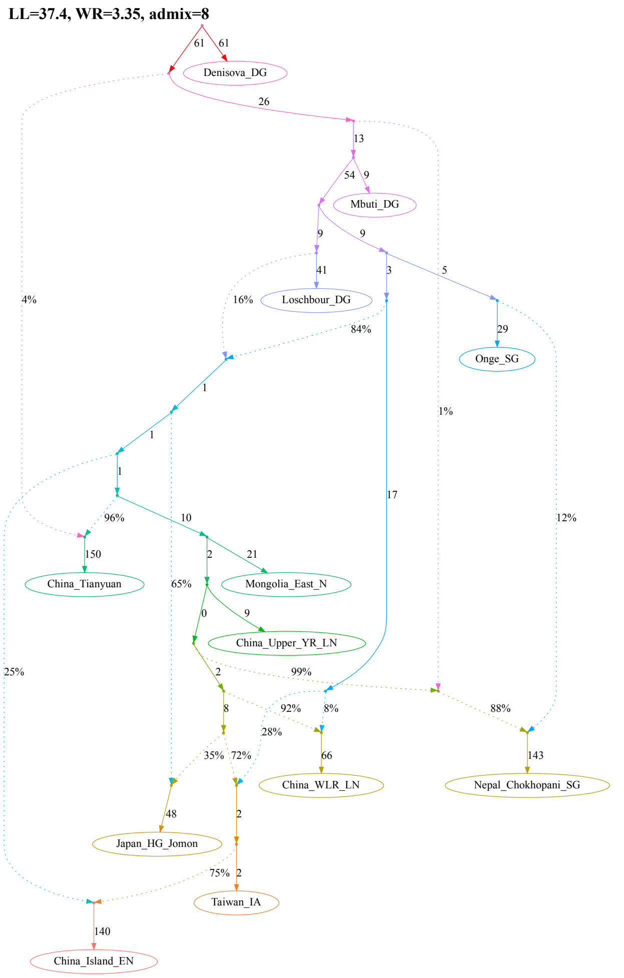

f, alternative models fitting significantly better than the published one

no. of East Asian groups with Onge-related ancestry	1	no. of East Asian groups with Onge-related ancestry	1
no. of East Asian groups with European-related ancestry	8	no. of East Asian groups with European-related ancestry	8
direction of gene flow China_Island_EN > Taiwan_IA	reverse	direction of gene flow China_Island_EN > Taiwan_IA	reverse
Mongolia_East is basal with respect to China_Upper_YR and Nepal_Chokhopani	yes	Mongolia_East is basal with respect to China_Upper_YR and Nepal_Chokhopani	yes
Taiwan_IA is basal with respect to Mongolia_East and Nepal_Chokhopani	yes	Taiwan_IA is basal with respect to Mongolia_East and Nepal_Chokhopani	yes
The original set of SNPs and individuals and the corrected algorithm for calculating f_3-statistics (203,753 variable site with no missing data at the group level)		The original set of SNPs and individuals and the original algorithm for calculating f_3-statistics (496,233 variable site with no missing data at the group level)	

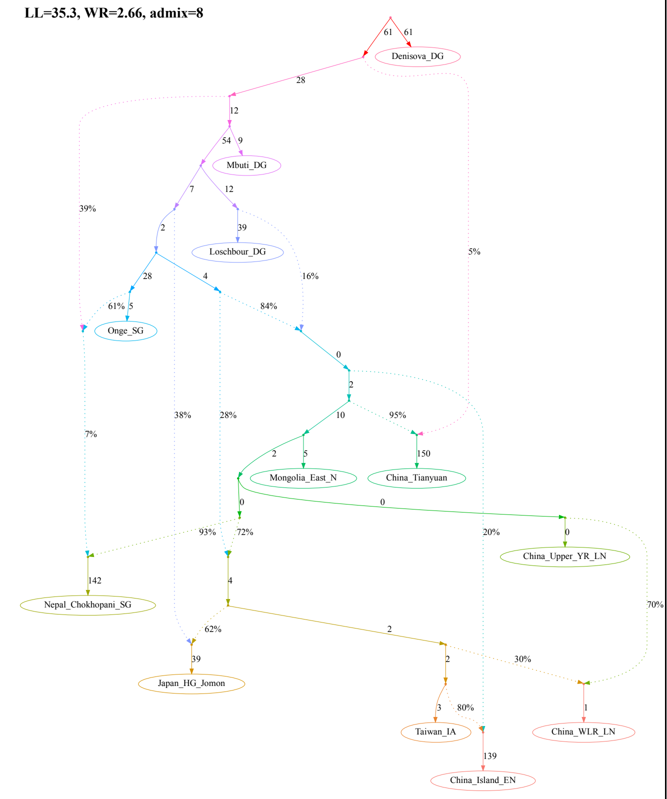

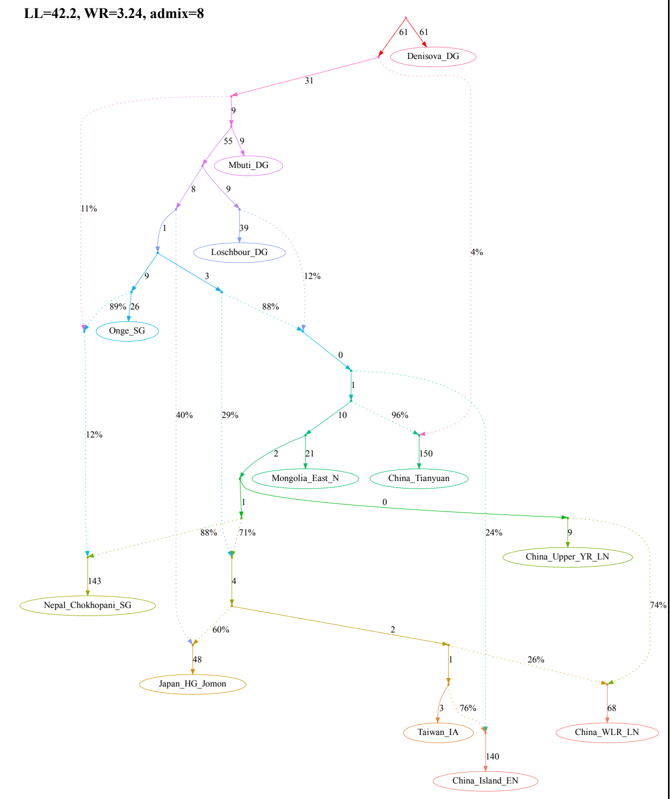

no. of East Asian groups with Onge-related ancestry	1
no. of East Asian groups with European-related ancestry	8
direction of gene flow China_Island_EN > Taiwan_IA	reverse
Mongolia_East is basal with respect to China_Upper_YR and Nepal_Chokhopani	yes
Taiwan_IA is basal with respect to Mongolia_East and Nepal_Chokhopani	yes

The original set of SNPs and individuals and the corrected algorithm for calculating f_3-statistics (203,753 variable site with no missing data at the group level)

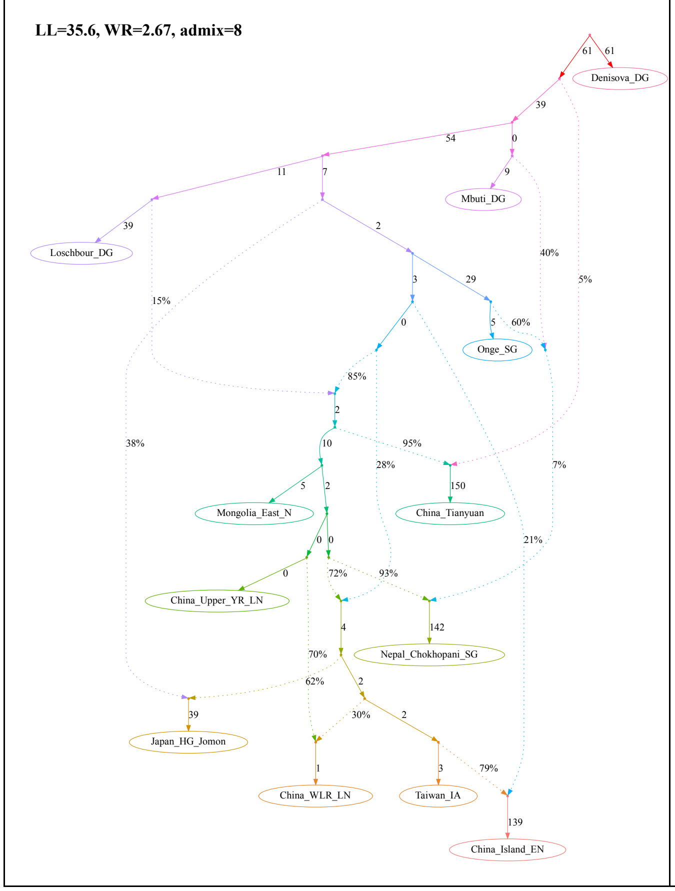

no. of East Asian groups with Onge-related ancestry	1
no. of East Asian groups with European-related ancestry	8
direction of gene flow China_Island_EN > Taiwan_IA	reverse
Mongolia_East is basal with respect to China_Upper_YR and Nepal_Chokhopani	yes
Taiwan_IA is basal with respect to Mongolia_East and Nepal_Chokhopani	yes

The original set of SNPs and individuals and the original algorithm for calculating f_3-statistics (496,233 variable site with no missing data at the group level)

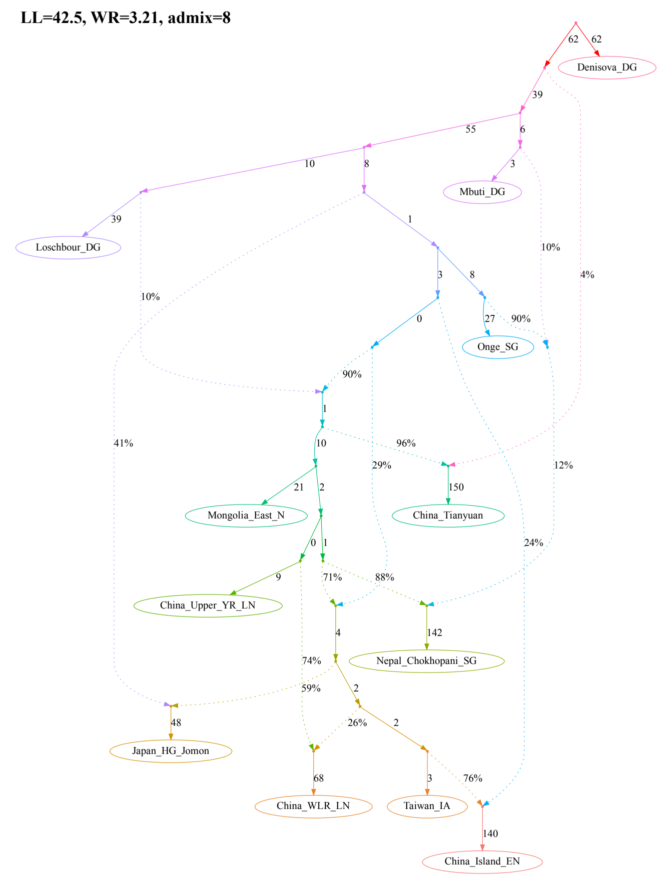

g, alternative models fitting significantly better than the published one

no. of East Asian groups with Onge-related ancestry: 0<br>no. of East Asian groups with European-related ancestry: 8<br>direction of gene flow China_Island_EN > Taiwan_IA: no gene flow<br>Mongolia_East is basal with respect to China_Upper_YR and Nepal_Chokhopani: yes<br>Taiwan_IA is basal with respect to Mongolia_East and Nepal_Chokhopani: yes	no. of East Asian groups with Onge-related ancestry: 0<br>no. of East Asian groups with European-related ancestry: 8<br>direction of gene flow China_Island_EN > Taiwan_IA: no gene flow<br>Mongolia_East is basal with respect to China_Upper_YR and Nepal_Chokhopani: yes<br>Taiwan_IA is basal with respect to Mongolia_East and Nepal_Chokhopani: yes
The original set of SNPs and individuals and the corrected algorithm for calculating f_3-statistics (203,753 variable site with no missing data at the group level)	The original set of SNPs and individuals and the original algorithm for calculating f_3-statistics (496,233 variable site with no missing data at the group level)

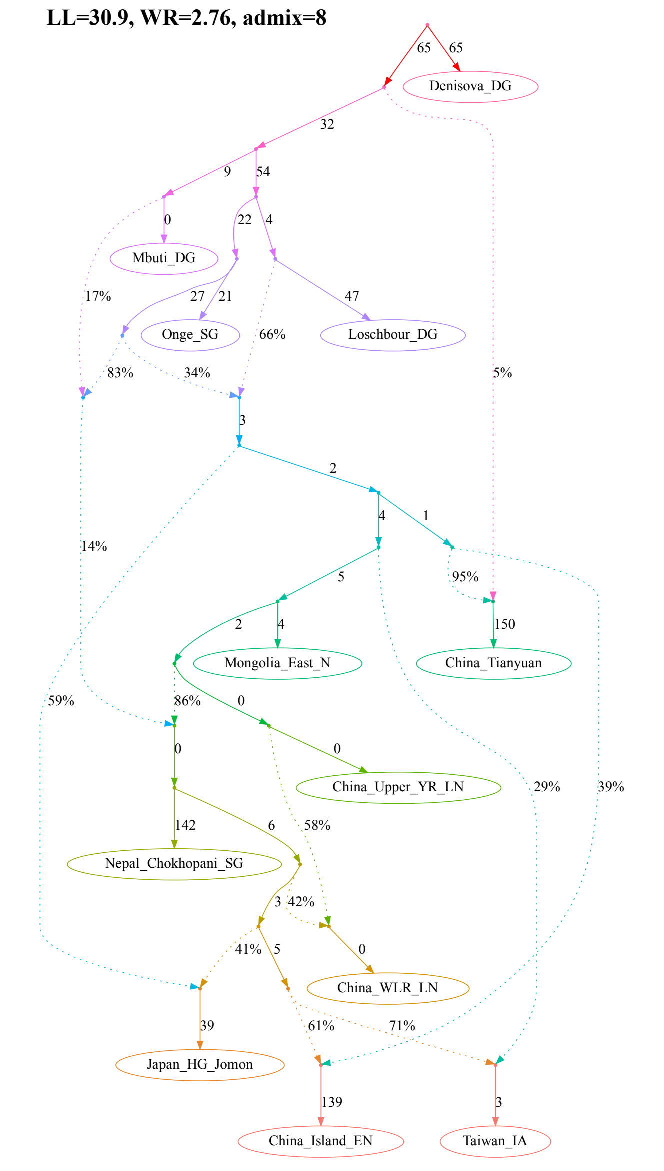

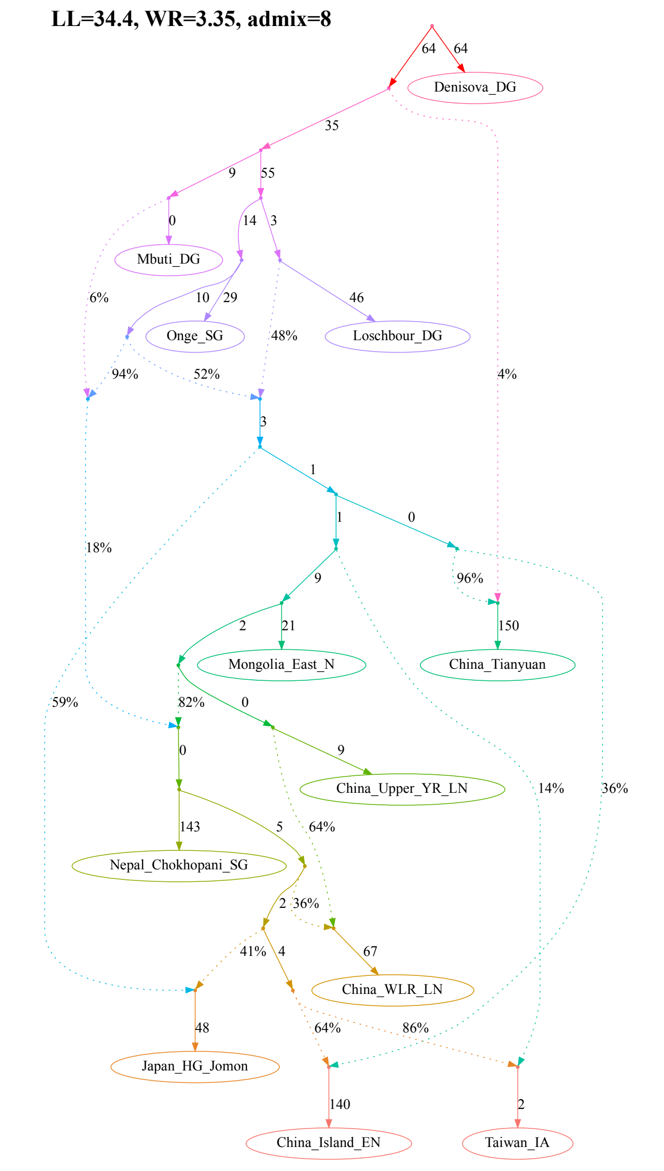

	The original set of SNPs and individuals and the corrected algorithm for calculating f_3-statistics (203,753 variable site with no missing data at the group level)	The original set of SNPs and individuals and the original algorithm for calculating f_3-statistics (496,233 variable site with no missing data at the group level)
no. of East Asian groups with Onge-related ancestry	0	0
no. of East Asian groups with European-related ancestry	8	8
direction of gene flow China_Island_EN > Taiwan_IA	no gene flow	no gene flow
Mongolia_East is basal with respect to China_Upper_YR and Nepal_Chokhopani	yes	yes
Taiwan_IA is basal with respect to Mongolia_East and Nepal_Chokhopani	yes	yes

LL=33.7, WR=3.06, admix=8

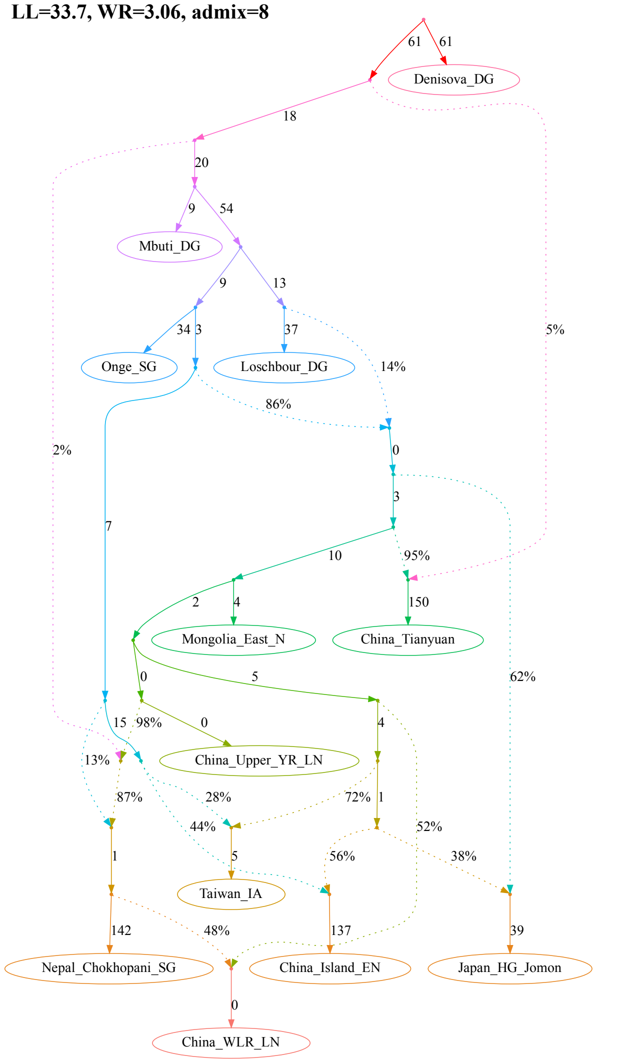

LL=36.1, WR=2.68, admix=8

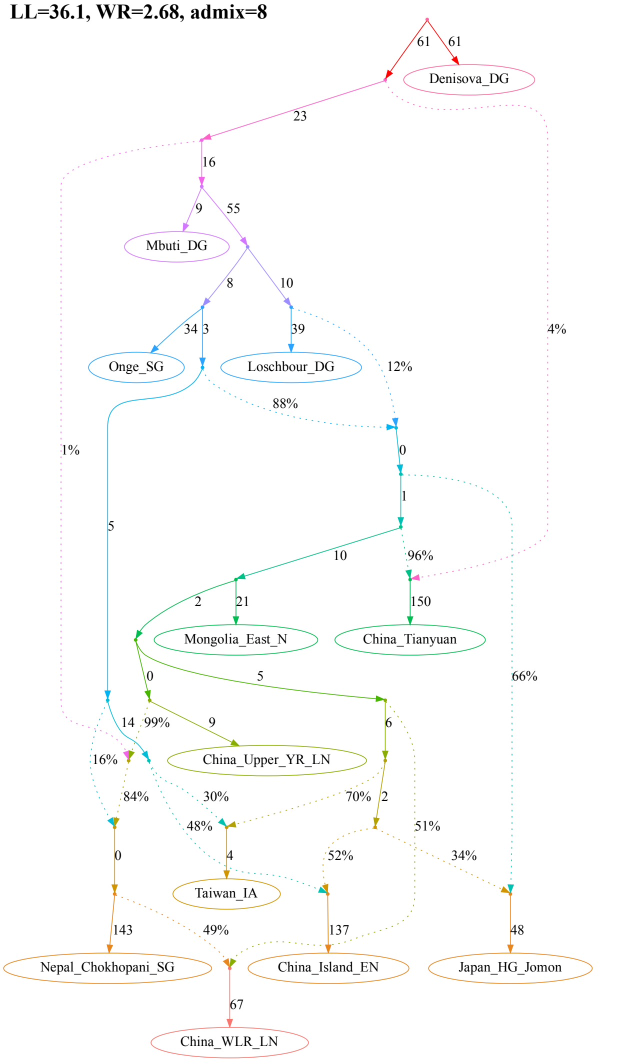

h, alternative models fitting significantly better than the published one

no. of East Asian groups with Onge-related ancestry: 0<br>no. of East Asian groups with European-related ancestry: 8<br>direction of gene flow China_Island_EN > Taiwan_IA: reverse<br>Mongolia_East is basal with respect to China_Upper_YR and Nepal_Chokhopani: yes<br>Taiwan_IA is basal with respect to Mongolia_East and Nepal_Chokhopani: unclear	no. of East Asian groups with Onge-related ancestry: 0<br>no. of East Asian groups with European-related ancestry: 8<br>direction of gene flow China_Island_EN > Taiwan_IA: reverse<br>Mongolia_East is basal with respect to China_Upper_YR and Nepal_Chokhopani: yes<br>Taiwan_IA is basal with respect to Mongolia_East and Nepal_Chokhopani: unclear
The original set of SNPs and individuals and the corrected algorithm for calculating f_3-statistics (203,753 variable site with no missing data at the group level)	The original set of SNPs and individuals and the original algorithm for calculating f_3-statistics (496,233 variable site with no missing data at the group level)

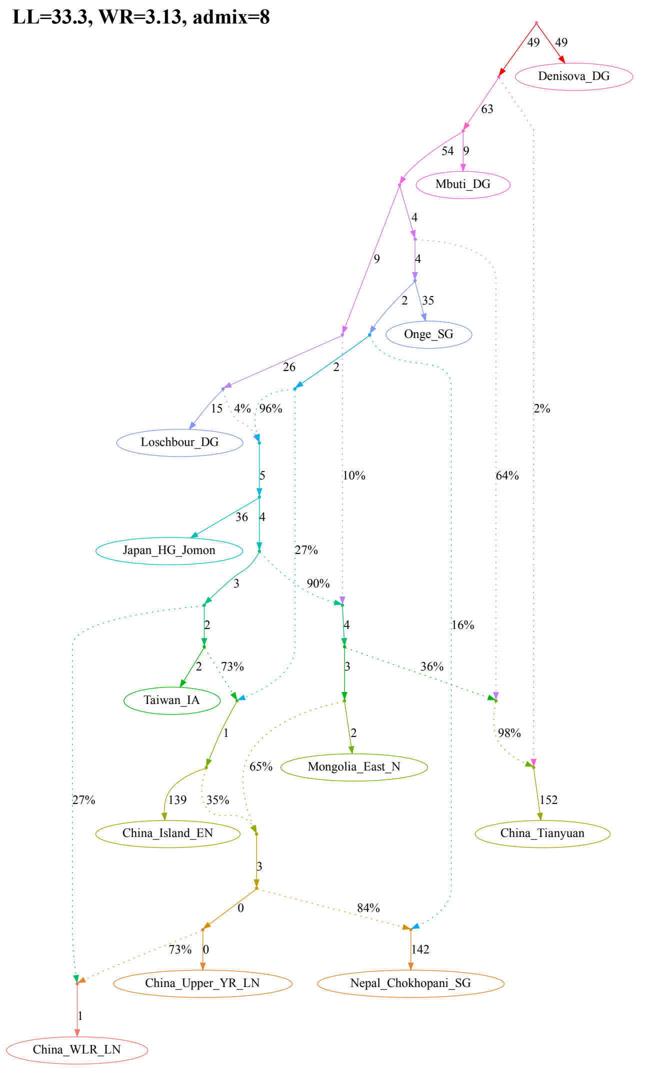

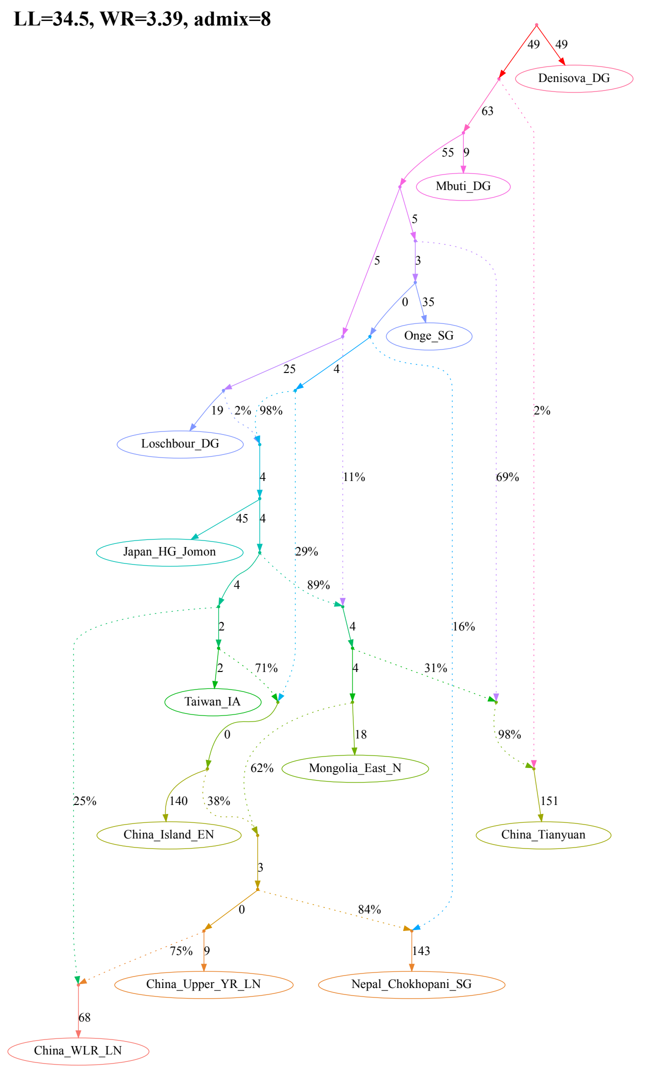

no. of East Asian groups with Onge-related ancestry	0
no. of East Asian groups with European-related ancestry	8
direction of gene flow China_Island_EN > Taiwan_IA	reverse
Mongolia_East is basal with respect to China_Upper_YR and Nepal_Chokhopani	yes
Taiwan_IA is basal with respect to Mongolia_East and Nepal_Chokhopani	yes

The original set of SNPs and individuals and the corrected algorithm for calculating f_3-statistics (203,753 variable site with no missing data at the group level)

LL=30.3, WR=2.27, admix=8

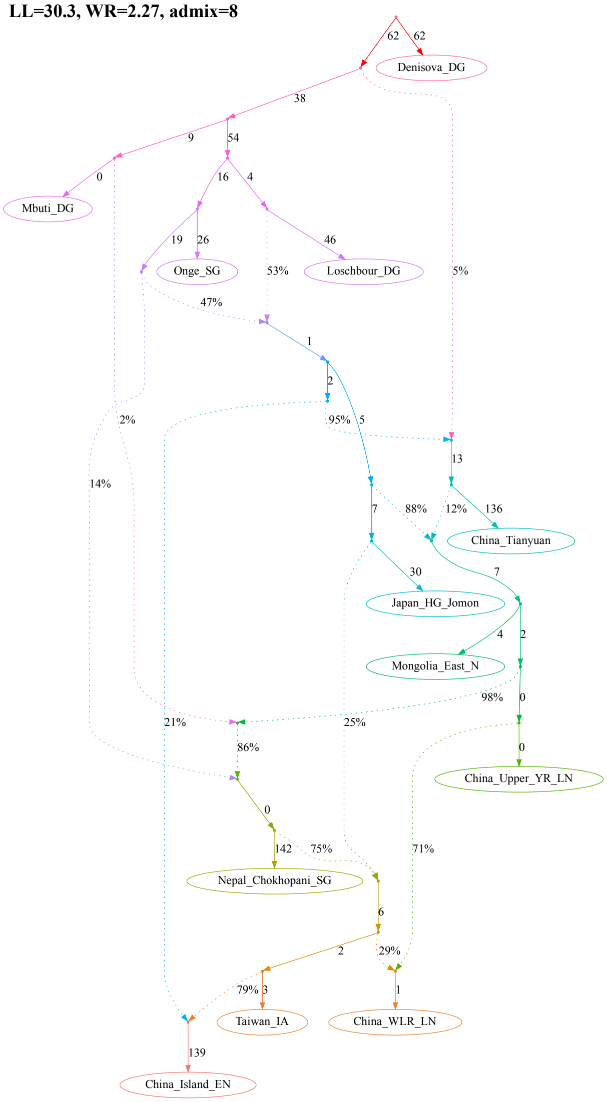

no. of East Asian groups with Onge-related ancestry	0
no. of East Asian groups with European-related ancestry	8
direction of gene flow China_Island_EN > Taiwan_IA	reverse
Mongolia_East is basal with respect to China_Upper_YR and Nepal_Chokhopani	yes
Taiwan_IA is basal with respect to Mongolia_East and Nepal_Chokhopani	yes

The original set of SNPs and individuals and the original algorithm for calculating f_3-statistics (496,233 variable site with no missing data at the group level)

LL=29.7, WR=3.34, admix=8

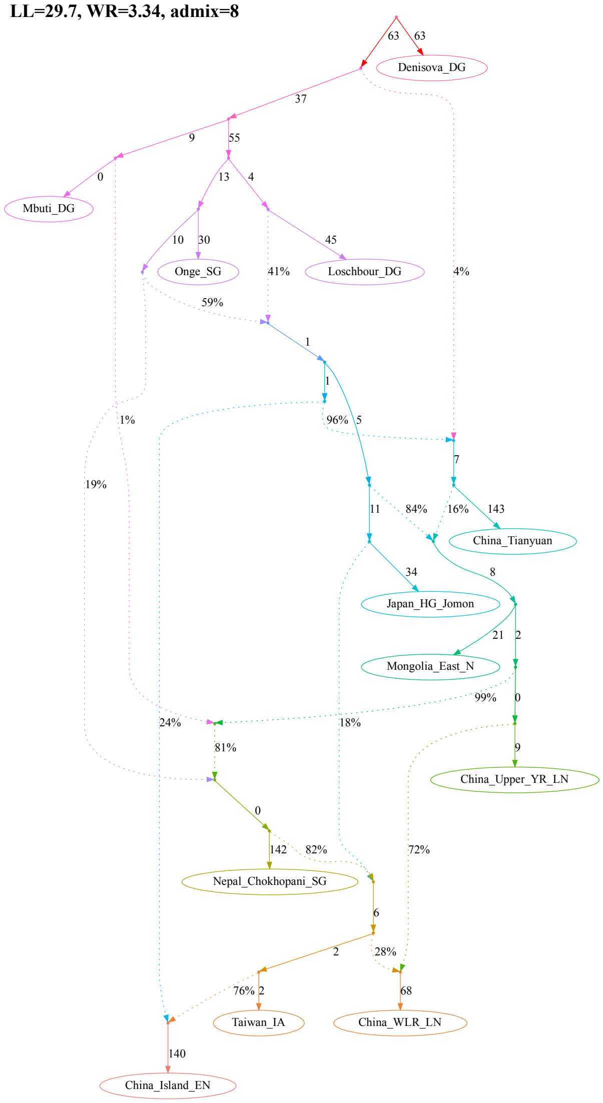

i, alternative models fitting significantly better than the published one

no. of East Asian groups with Onge-related ancestry	0	no. of East Asian groups with Onge-related ancestry	0
no. of East Asian groups with European-related ancestry	8	no. of East Asian groups with European-related ancestry	8
direction of gene flow China_Island_EN > Taiwan_IA	reverse	direction of gene flow China_Island_EN > Taiwan_IA	reverse
Mongolia_East is basal with respect to China_Upper_YR and Nepal_Chokhopani	yes	Mongolia_East is basal with respect to China_Upper_YR and Nepal_Chokhopani	yes
Taiwan_IA is basal with respect to Mongolia_East and Nepal_Chokhopani	yes	Taiwan_IA is basal with respect to Mongolia_East and Nepal_Chokhopani	yes

The original set of SNPs and individuals and the corrected algorithm for calculating f_3-statistics (203,753 variable site with no missing data at the group level)	The original set of SNPs and individuals and the original algorithm for calculating f_3-statistics (496,233 variable site with no missing data at the group level)

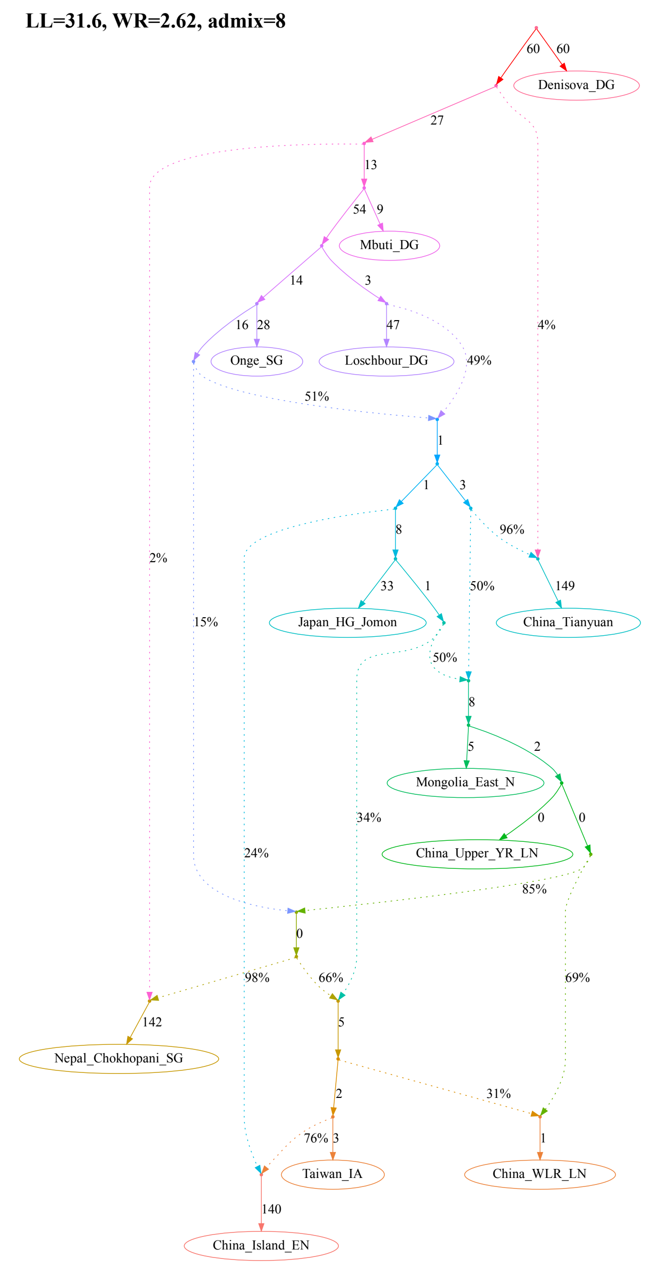

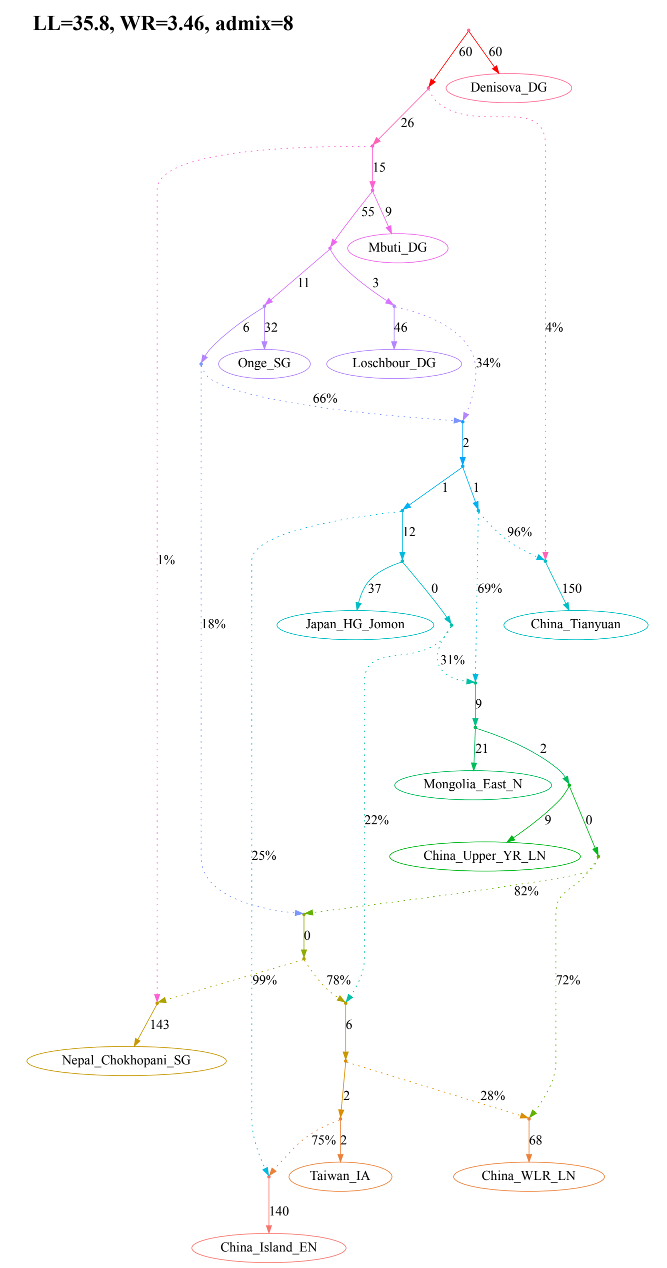

no. of East Asian groups with Onge-related ancestry	0
no. of East Asian groups with European-related ancestry	8
direction of gene flow China_Island_EN > Taiwan_IA	reverse
Mongolia_East is basal with respect to China_Upper_YR and Nepal_Chokhopani	yes
Taiwan_IA is basal with respect to Mongolia_East and Nepal_Chokhopani	yes

The original set of SNPs and individuals and the corrected algorithm for calculating f_3-statistics (203,753 variable site with no missing data at the group level)

LL=33.2, WR=2.61, admix=8

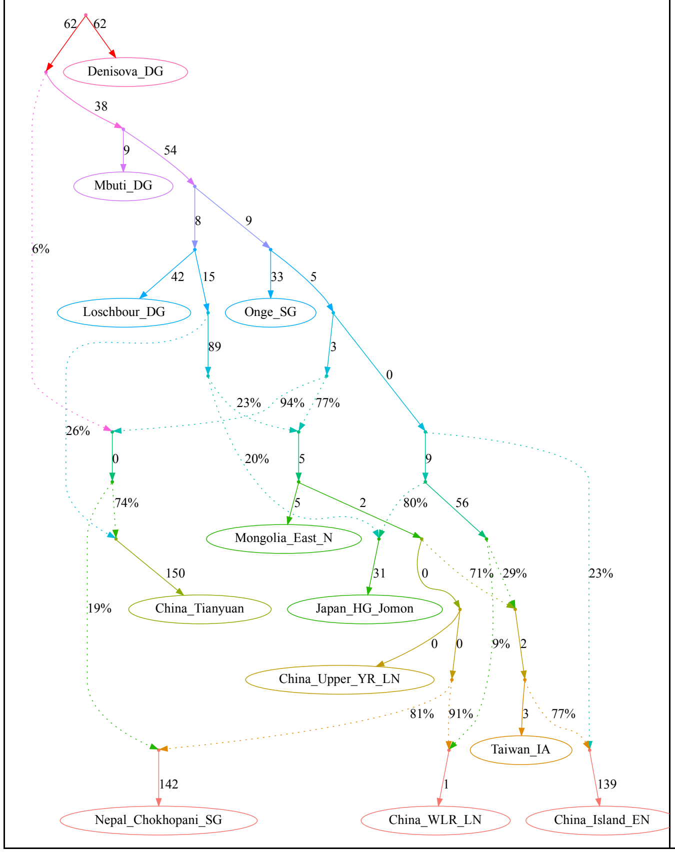

no. of East Asian groups with Onge-related ancestry	0
no. of East Asian groups with European-related ancestry	8
direction of gene flow China_Island_EN > Taiwan_IA	reverse
Mongolia_East is basal with respect to China_Upper_YR and Nepal_Chokhopani	yes
Taiwan_IA is basal with respect to Mongolia_East and Nepal_Chokhopani	yes

The original set of SNPs and individuals and the original algorithm for calculating f_3-statistics (496,233 variable site with no missing data at the group level)

LL=40.1, WR=3.43, admix=8

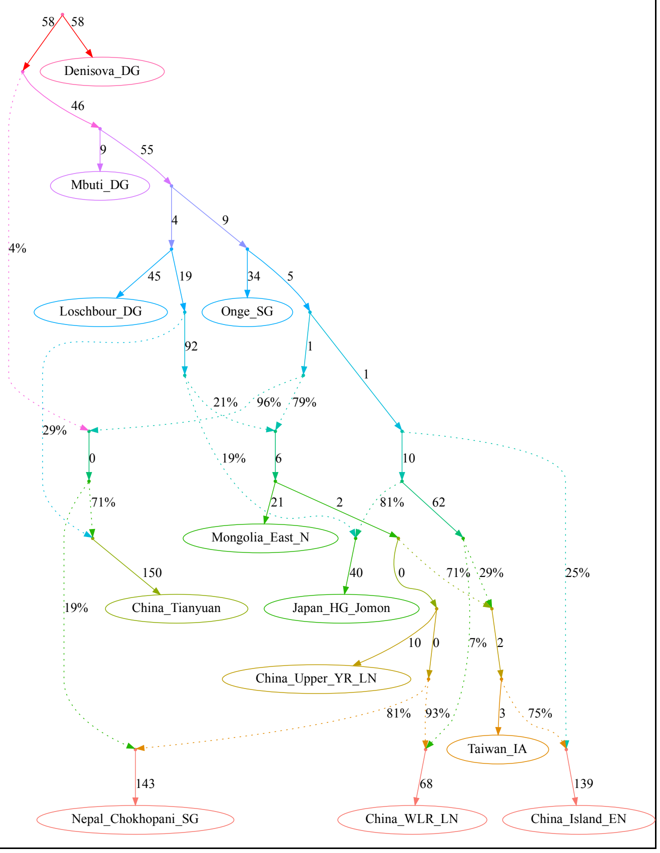

j, alternative models fitting significantly better than the published one

no. of East Asian groups with Onge-related ancestry 0<br>no. of East Asian groups with European-related ancestry 8<br>direction of gene flow China_Island_EN > Taiwan_IA reverse<br>Mongolia_East is basal with respect to China_Upper_YR and Nepal_Chokhopani yes<br>Taiwan_IA is basal with respect to Mongolia_East and Nepal_Chokhopani yes	no. of East Asian groups with Onge-related ancestry 0<br>no. of East Asian groups with European-related ancestry 8<br>direction of gene flow China_Island_EN > Taiwan_IA reverse<br>Mongolia_East is basal with respect to China_Upper_YR and Nepal_Chokhopani yes<br>Taiwan_IA is basal with respect to Mongolia_East and Nepal_Chokhopani yes
The original set of SNPs and individuals and the corrected algorithm for calculating f_3-statistics (203,753 variable site with no missing data at the group level)	The original set of SNPs and individuals and the original algorithm for calculating f_3-statistics (496,233 variable site with no missing data at the group level)

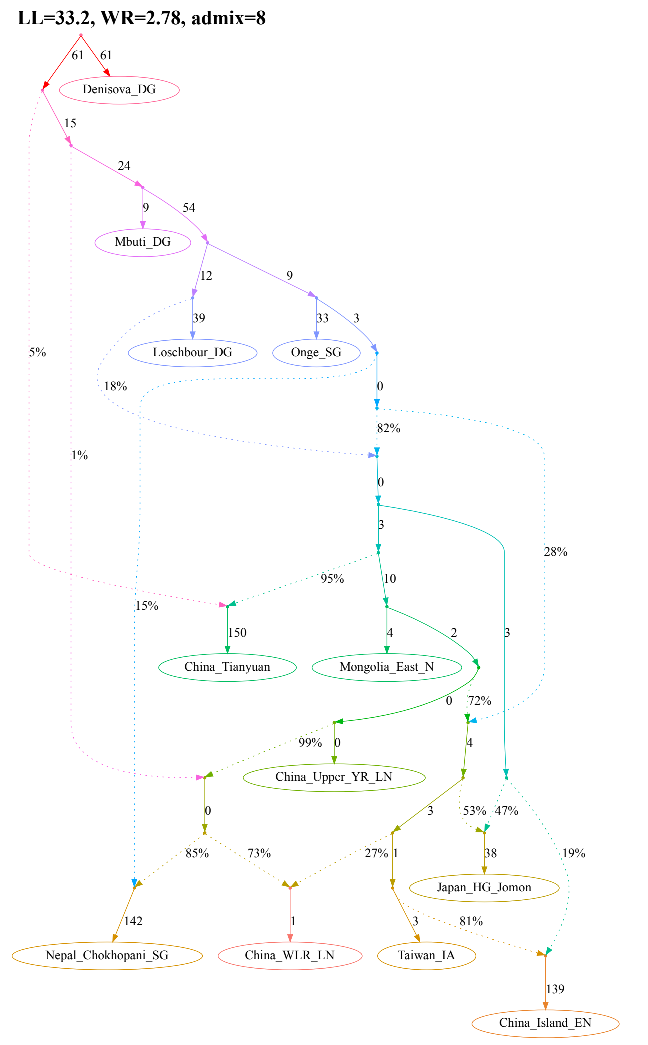

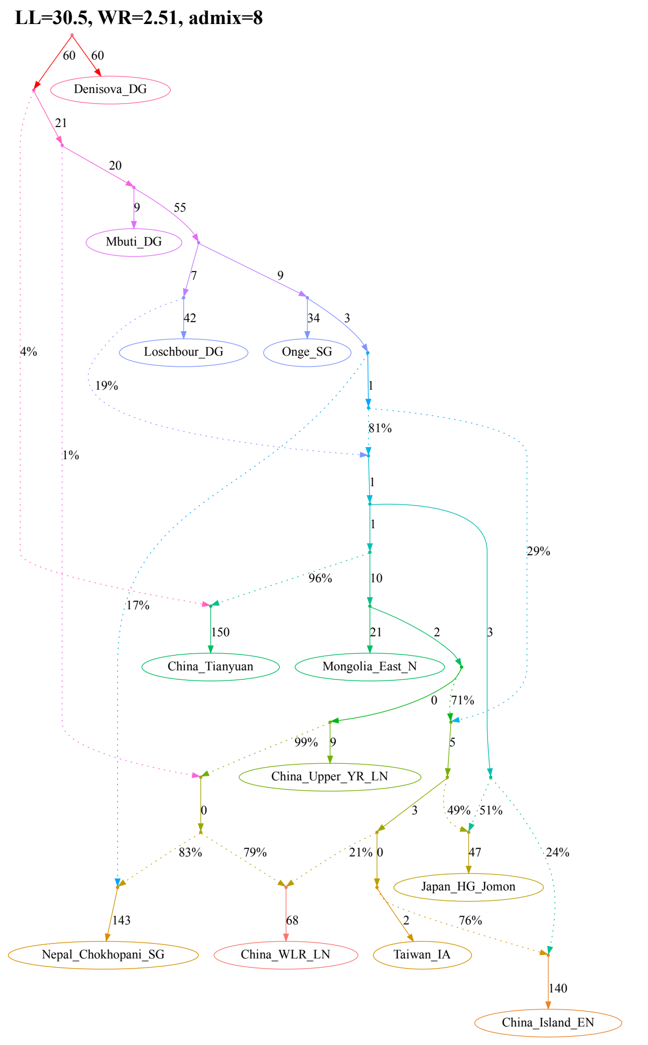

no. of East Asian groups with Onge-related ancestry	0
no. of East Asian groups with European-related ancestry	8
direction of gene flow China_Island_EN > Taiwan_IA	reverse
Mongolia_East is basal with respect to China_Upper_YR and Nepal_Chokhopani	yes
Taiwan_IA is basal with respect to Mongolia_East and Nepal_Chokhopani	yes

The original set of SNPs and individuals and the corrected algorithm for calculating f_3-statistics (203,753 variable site with no missing data at the group level)

LL=34.3, WR=3.17, admix=8

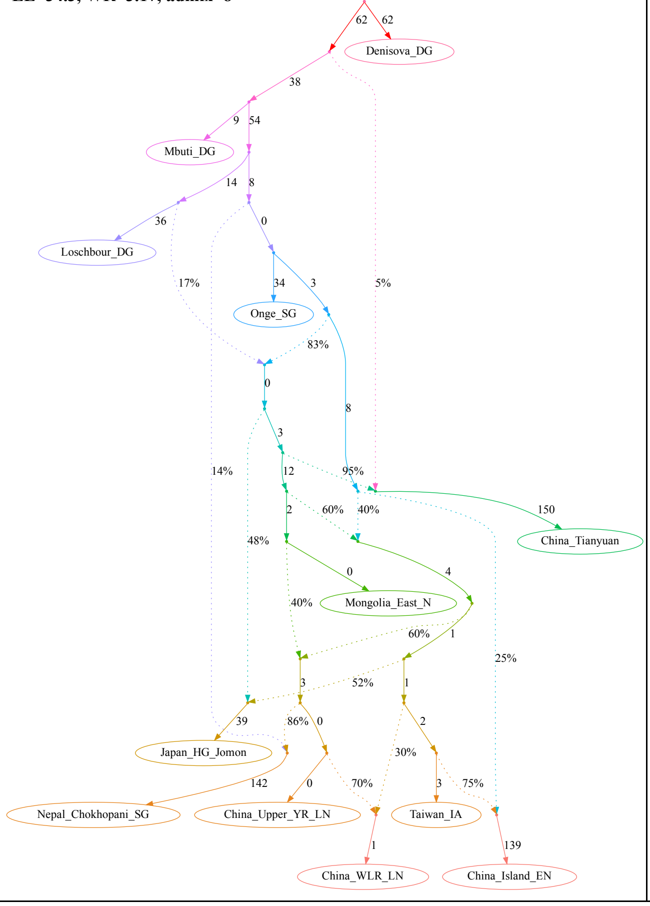

no. of East Asian groups with Onge-related ancestry	0
no. of East Asian groups with European-related ancestry	8
direction of gene flow China_Island_EN > Taiwan_IA	reverse
Mongolia_East is basal with respect to China_Upper_YR and Nepal_Chokhopani	yes
Taiwan_IA is basal with respect to Mongolia_East and Nepal_Chokhopani	yes

The original set of SNPs and individuals and the original algorithm for calculating f_3-statistics (496,233 variable site with no missing data at the group level)

LL=31.9, WR=3.2, admix=8

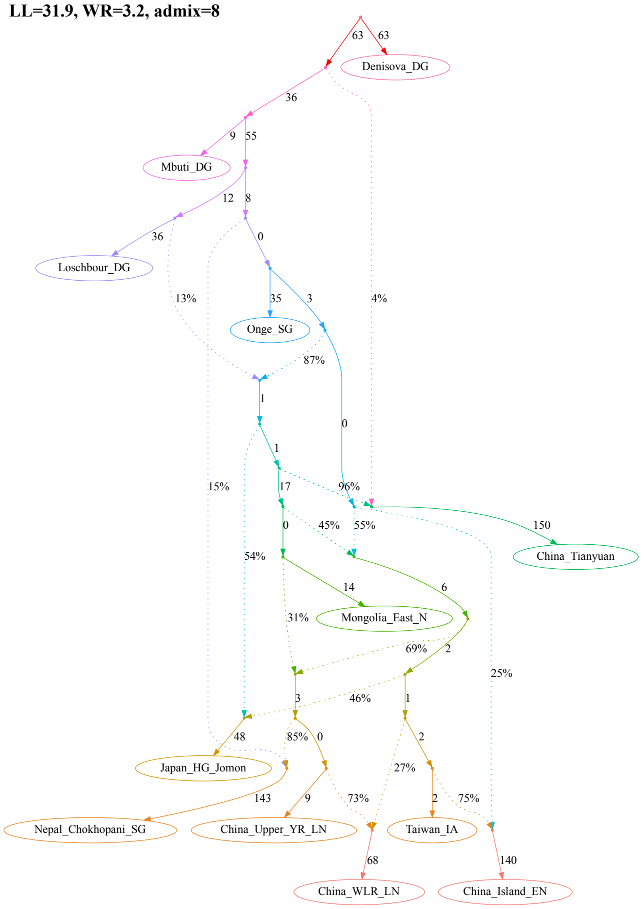

k, alternative models fitting significantly better than the published one

no. of East Asian groups with Onge-related ancestry	0
no. of East Asian groups with European-related ancestry	8
direction of gene flow China_Island_EN > Taiwan_IA	reverse
Mongolia_East is basal with respect to China_Upper_YR and Nepal_Chokhopani	yes
Taiwan_IA is basal with respect to Mongolia_East and Nepal_Chokhopani	yes

The original set of SNPs and individuals and the corrected algorithm for calculating f_3-statistics (203,753 variable site with no missing data at the group level)

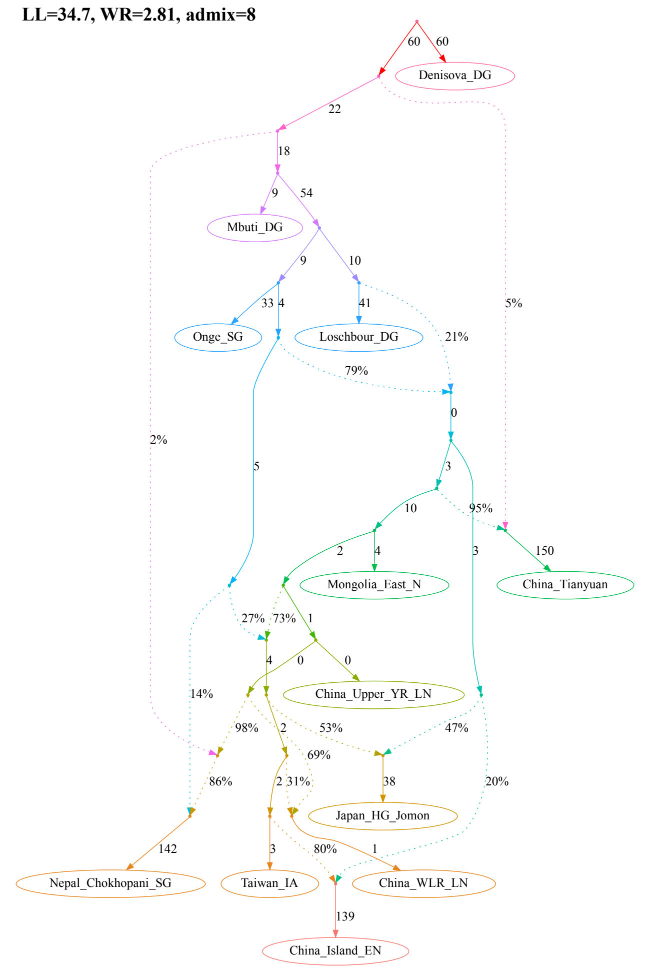

no. of East Asian groups with Onge-related ancestry	0
no. of East Asian groups with European-related ancestry	8
direction of gene flow China_Island_EN > Taiwan_IA	reverse
Mongolia_East is basal with respect to China_Upper_YR and Nepal_Chokhopani	yes
Taiwan_IA is basal with respect to Mongolia_East and Nepal_Chokhopani	yes

The original set of SNPs and individuals and the original algorithm for calculating f_3-statistics (496,233 variable site with no missing data at the group level)

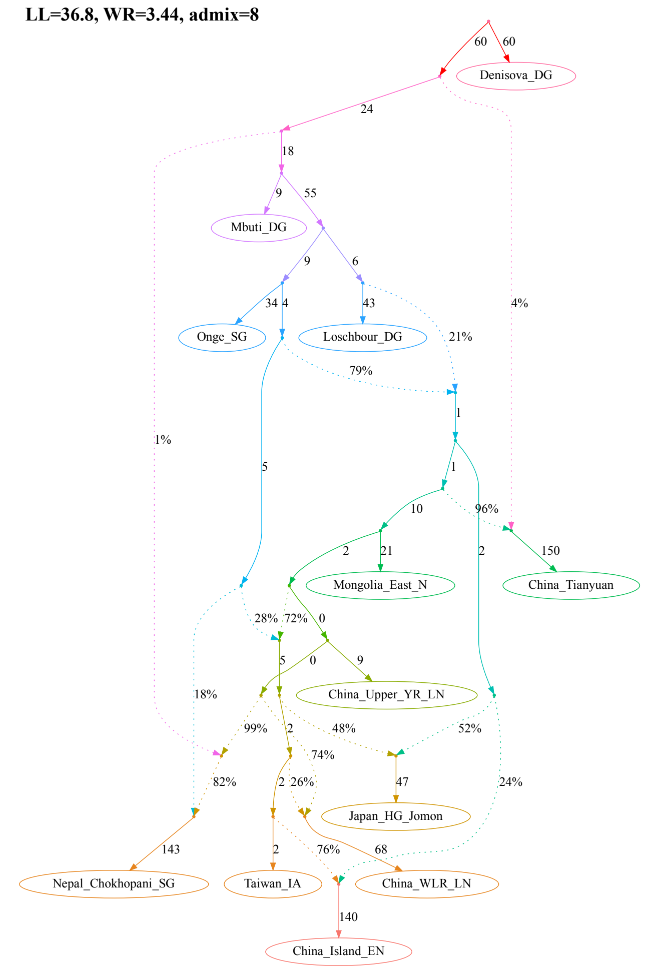

no. of East Asian groups with Onge-related ancestry: 0<br>no. of East Asian groups with European-related ancestry: 8<br>direction of gene flow China_Island_EN > Taiwan_IA: reverse<br>Mongolia_East is basal with respect to China_Upper_YR and Nepal_Chokhopani: yes<br>Taiwan_IA is basal with respect to Mongolia_East and Nepal_Chokhopani: yes	no. of East Asian groups with Onge-related ancestry: 0<br>no. of East Asian groups with European-related ancestry: 8<br>direction of gene flow China_Island_EN > Taiwan_IA: reverse<br>Mongolia_East is basal with respect to China_Upper_YR and Nepal_Chokhopani: yes<br>Taiwan_IA is basal with respect to Mongolia_East and Nepal_Chokhopani: yes
The original set of SNPs and individuals and the corrected algorithm for calculating f_3-statistics (203,753 variable site with no missing data at the group level)	The original set of SNPs and individuals and the original algorithm for calculating f_3-statistics (496,233 variable site with no missing data at the group level)

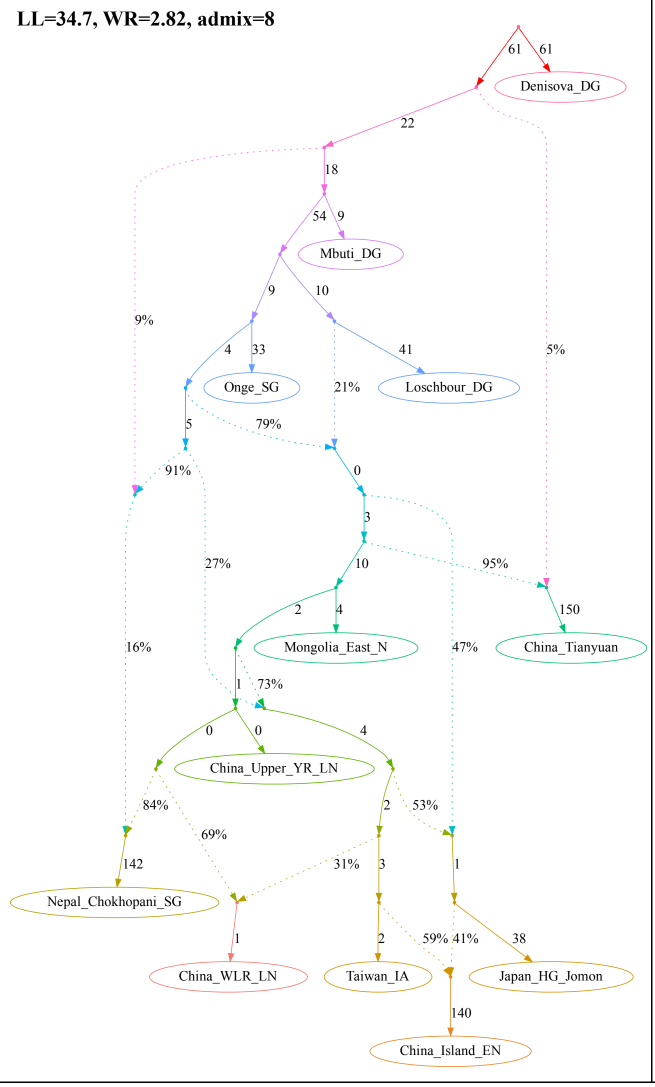

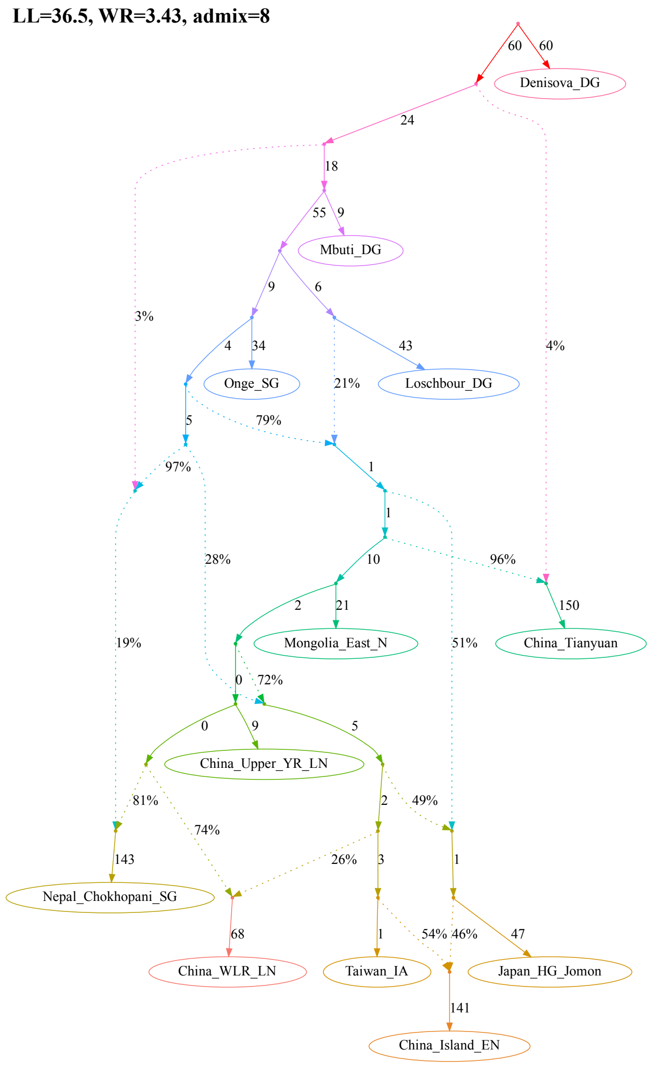

I, alternative models fitting significantly better than the published one

no. of East Asian groups with Onge-related ancestry	0	no. of East Asian groups with Onge-related ancestry	0
no. of East Asian groups with European-related ancestry	8	no. of East Asian groups with European-related ancestry	8
direction of gene flow China_Island_EN > Taiwan_IA	reverse	direction of gene flow China_Island_EN > Taiwan_IA	reverse
Mongolia_East is basal with respect to China_Upper_YR and Nepal_Chokhopani	yes	Mongolia_East is basal with respect to China_Upper_YR and Nepal_Chokhopani	yes
Taiwan_IA is basal with respect to Mongolia_East and Nepal_Chokhopani	yes	Taiwan_IA is basal with respect to Mongolia_East and Nepal_Chokhopani	yes

The original set of SNPs and individuals and the corrected algorithm for calculating f_3-statistics (203,753 variable site with no missing data at the group level)

LL=35.3, WR=2.91, admix=8

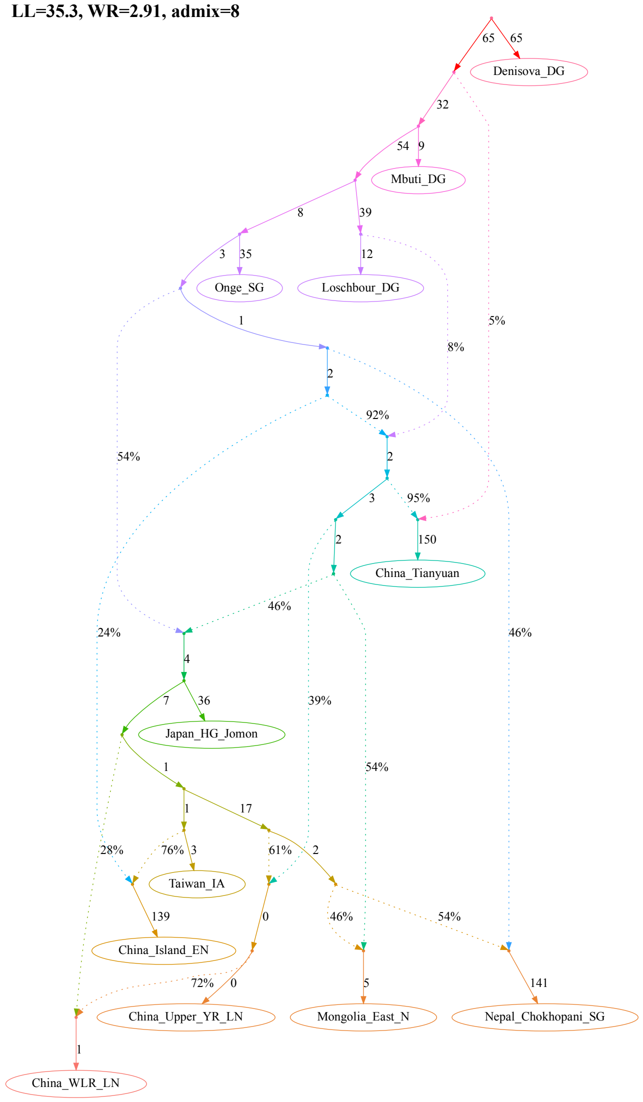

The original set of SNPs and individuals and the original algorithm for calculating f_3-statistics (496,233 variable site with no missing data at the group level)

LL=31.6, WR=3.21, admix=8

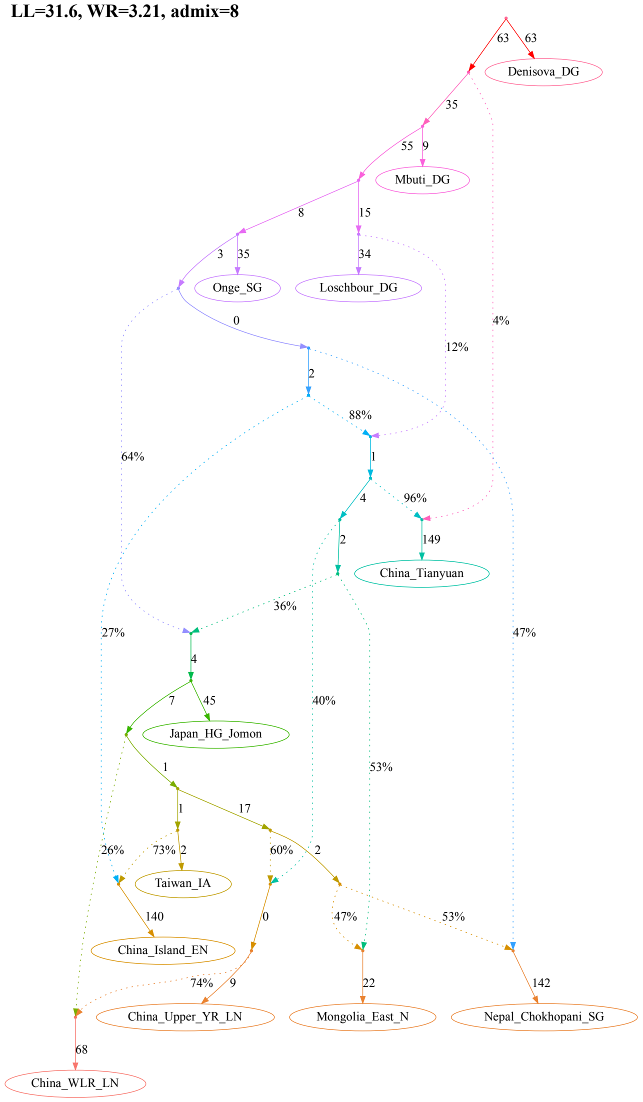

no. of East Asian groups with Onge-related ancestry	0
no. of East Asian groups with European-related ancestry	5
direction of gene flow China_Island_EN > Taiwan_IA	no gene flow
Mongolia_East is basal with respect to China_Upper_YR and Nepal_Chokhopani	yes
Taiwan_IA is basal with respect to Mongolia_East and Nepal_Chokhopani	unclear

The original set of SNPs and individuals and the corrected algorithm for calculating f_3-statistics (203,753 variable site with no missing data at the group level)

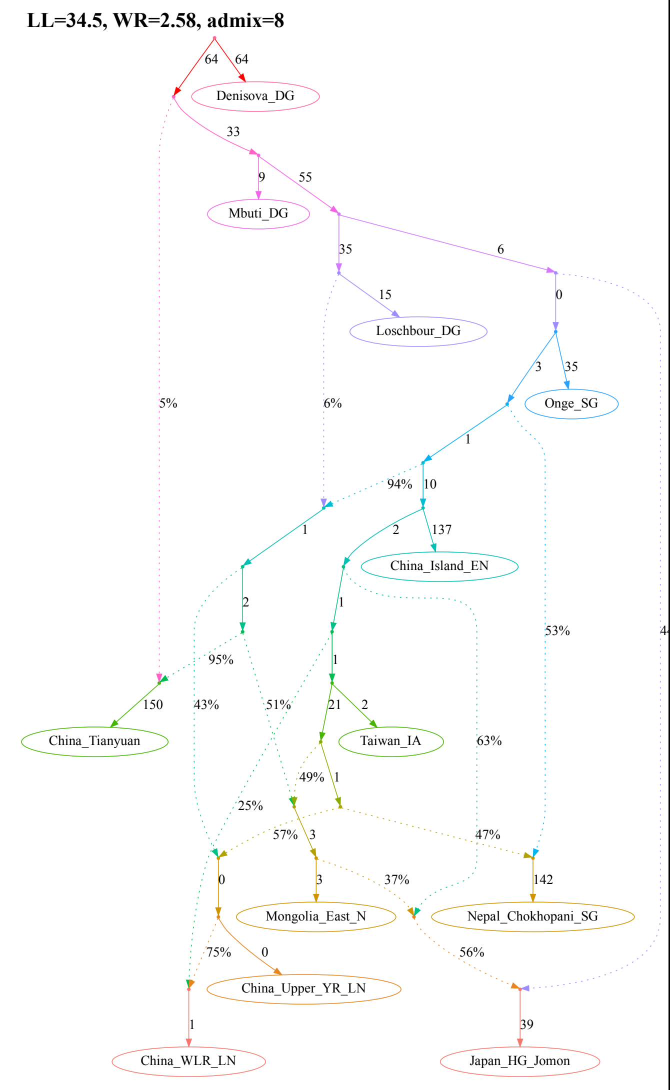

no. of East Asian groups with Onge-related ancestry	0
no. of East Asian groups with European-related ancestry	5
direction of gene flow China_Island_EN > Taiwan_IA	no gene flow
Mongolia_East is basal with respect to China_Upper_YR and Nepal_Chokhopani	yes
Taiwan_IA is basal with respect to Mongolia_East and Nepal_Chokhopani	unclear

The original set of SNPs and individuals and the original algorithm for calculating f_3-statistics (496,233 variable site with no missing data at the group level)

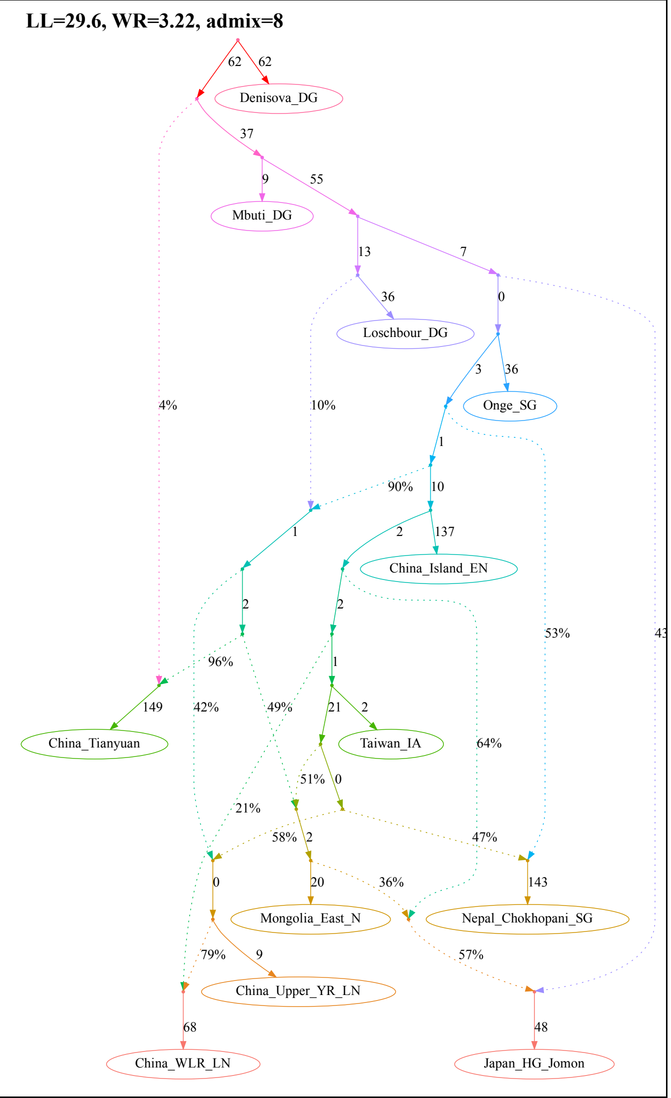

Supplement: Figure 4—source data 2. — The graphs were also re-fitted using the original algorithm for calculating f-statistics. Model parameters (graph edges) that were inferred to be unidentifiable are plotted in red. (a, b) The published model and its simplified representation. The following claim in Wang et al., 2021 relies on the admixture graph: There is Onge-related admixture in the Jomon (Japan_HG_Jomon), Tibetan (Nepal_Chokhopani_SG), Upper Yellow River Late Neolithic (China_Upper_YR_LN), West Liao River Late Neolithic (China_WLR_LN), Taiwan Iron Age (Taiwan_IA), and China Island Early Neolithic (Liangdao, China_Island_EN) lineages. (c-l) Alternative models fitting significantly better than the published one. [file elife-85492-fig4-data2.pdf]
