# Supplementary material for: On the limits of fitting complex models of population history to f-statistics
Source: eLife. 2023 Jun 29;12:e85492. doi: 10.7554/eLife.85492 (PMC10310323; doi:10.7554/eLife.85492)

Figure 4–source data 3. Simplified published admixture graph for West Eurasian groups from Sikora *et al*. (2019) and alternative graphs found with *findGraphs* (13 populations, 6 admixture events). Model parameters (graph edges) that were inferred to be unidentifiable are plotted in red.

a, published model, with chimpanzee added as an outgroup and 4 low-level gene flows from the Neanderthal lineage dropped

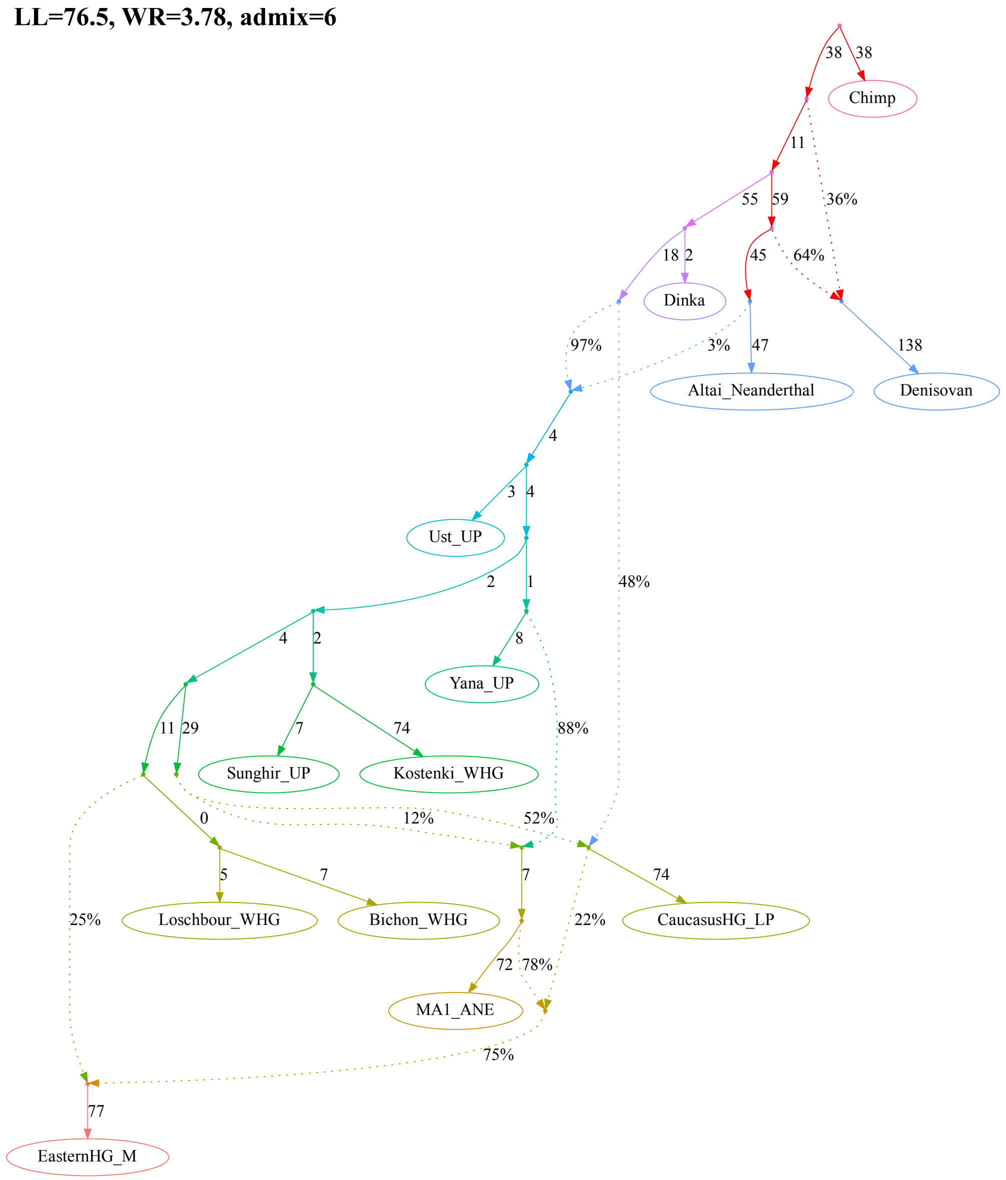

A claim by Sikora *et al*. 2019 relying on the admixture graph above:

The Mal'ta (MA1_ANE) lineage receives a gene flow from the Caucasus hunter-gatherer (CaucasusHG_LP) lineage.

b, models fitting nominally better than the published one and not supporting the claim

LL=65.9, WR=3.32, admix=6

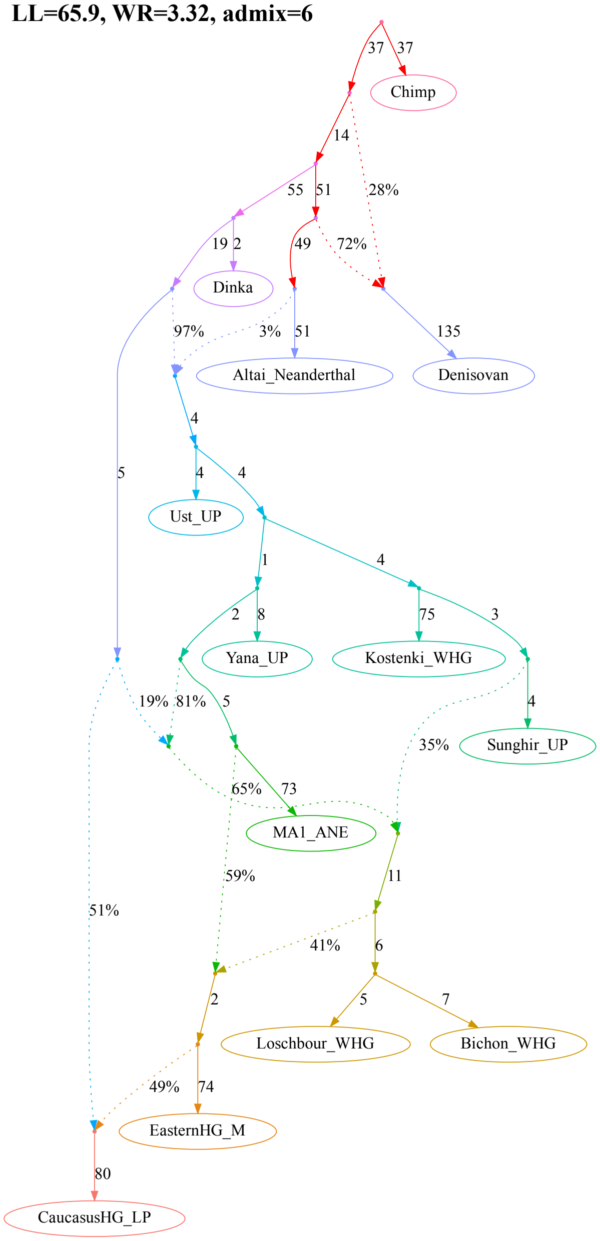

LL=67.6, WR=3.51, admix=6

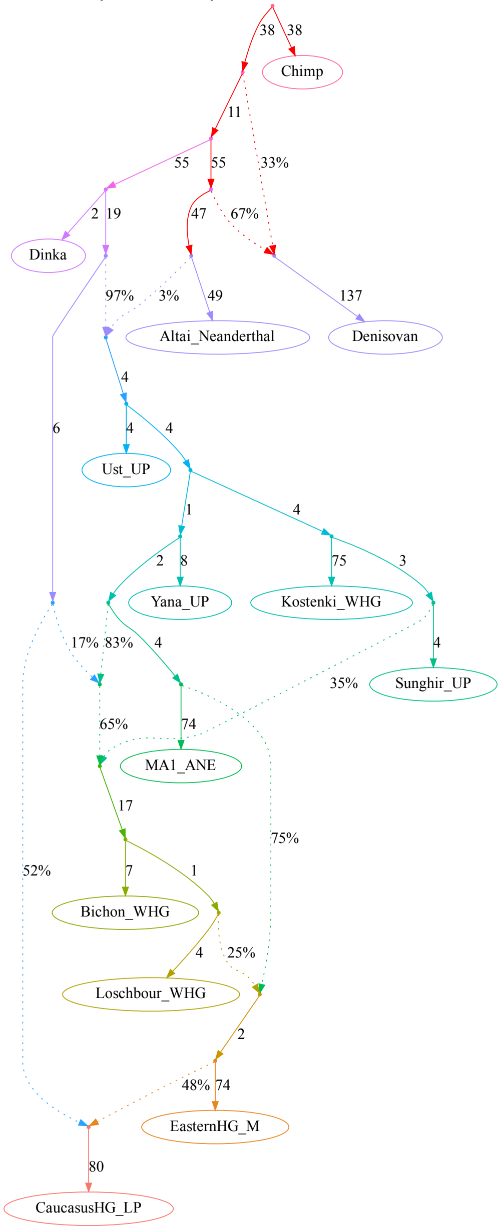

LL=67.6, WR=3.51, admix=6

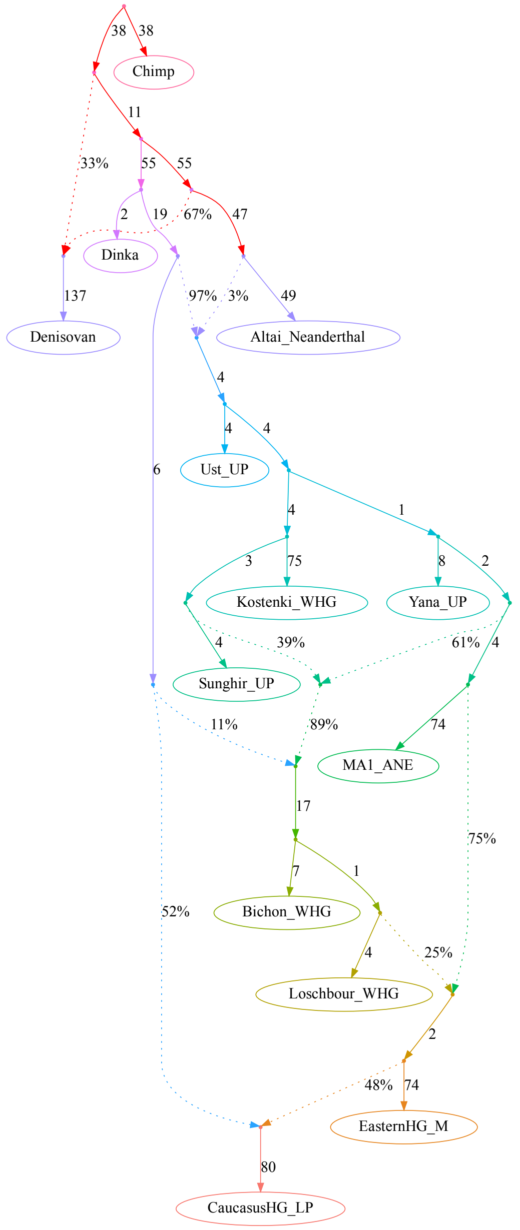

LL=68.3, WR=3.78, admix=6

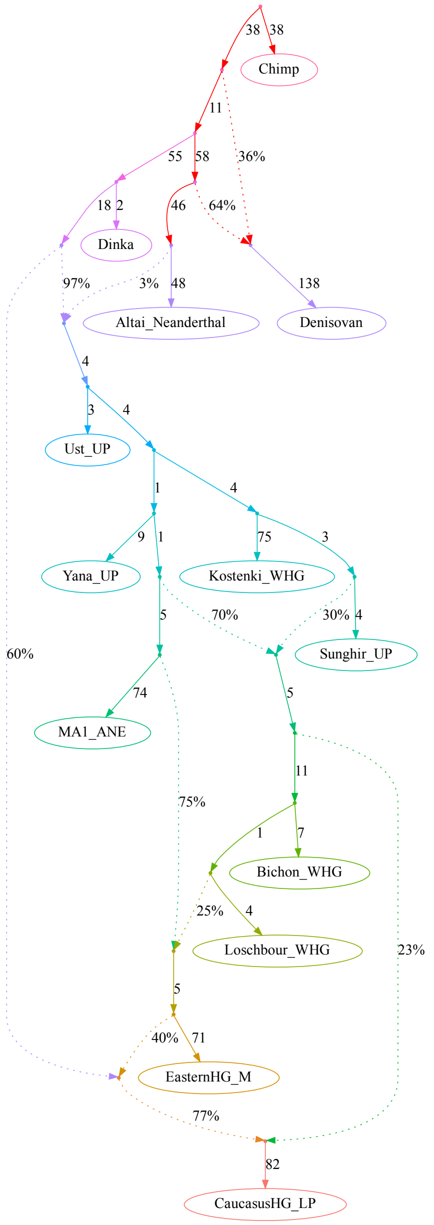

c, models fitting nominally better than the published one and not supporting the claim

LL=63.6, WR=3.57, admix=6

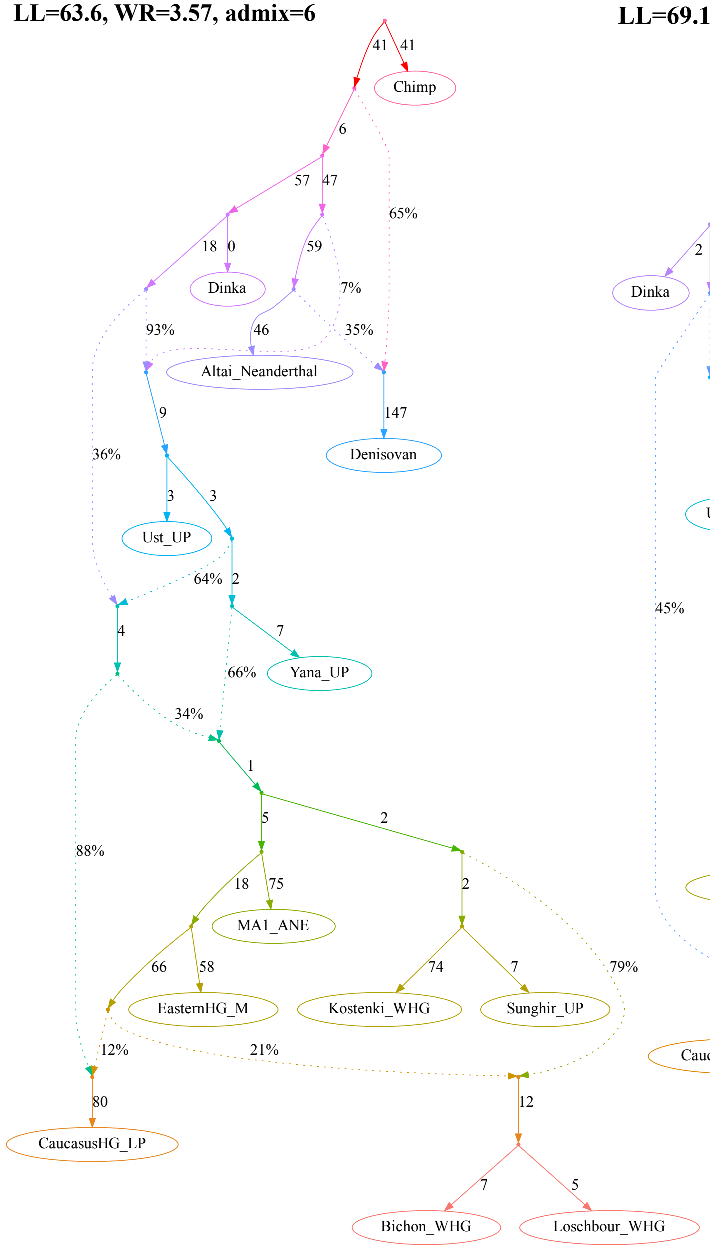

LL=69.1, WR=3.78, admix=6

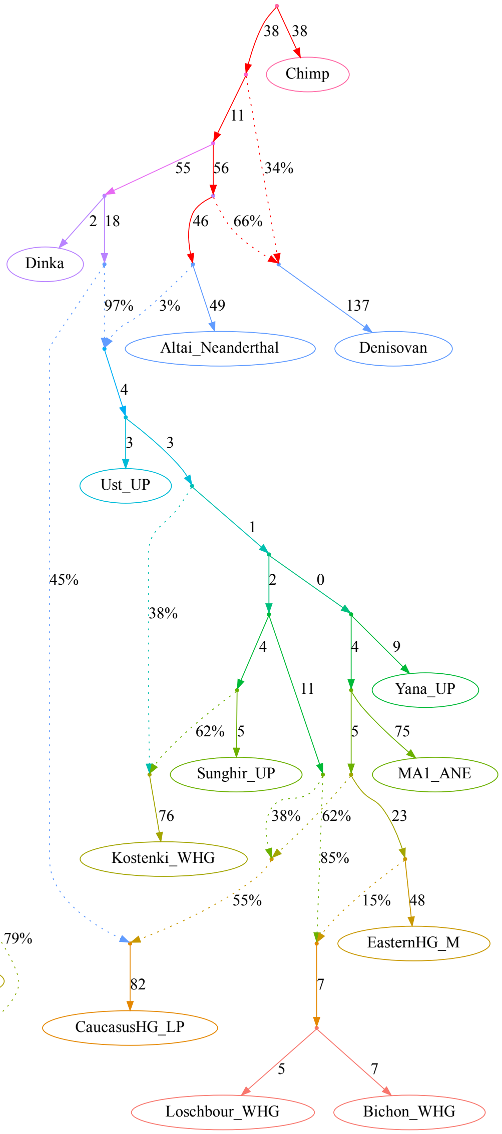

LL=69.2, WR=3.78, admix=6

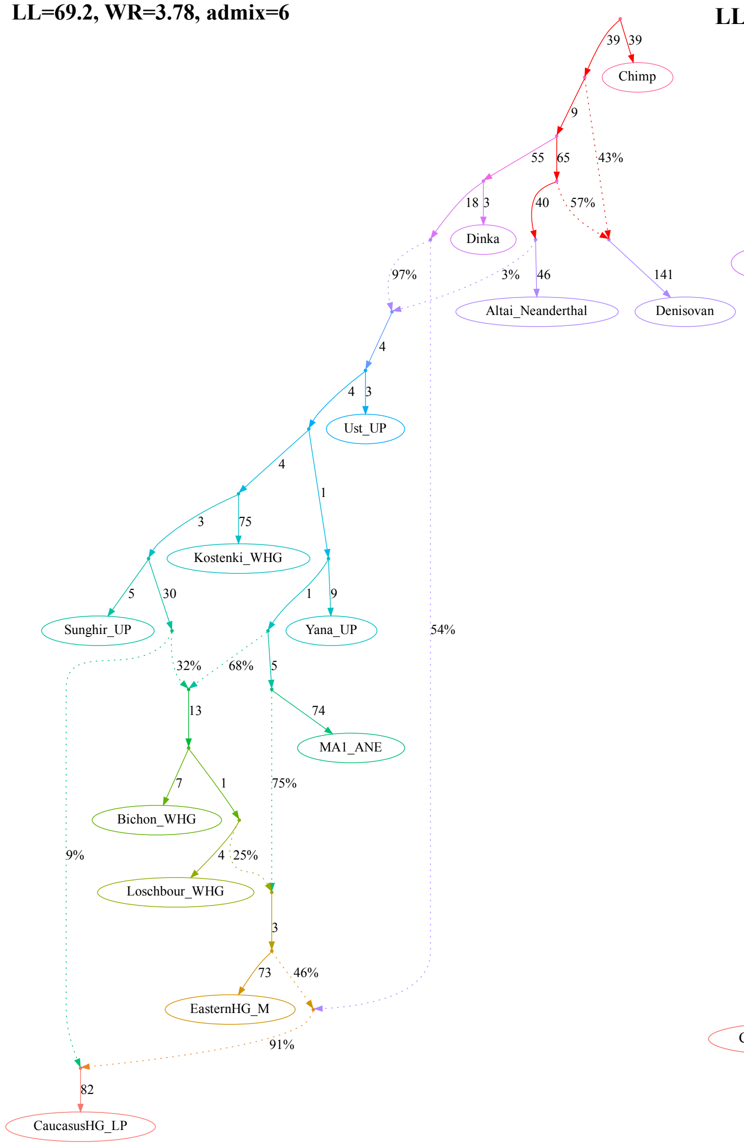

LL=69.7, WR=3.72, admix=6

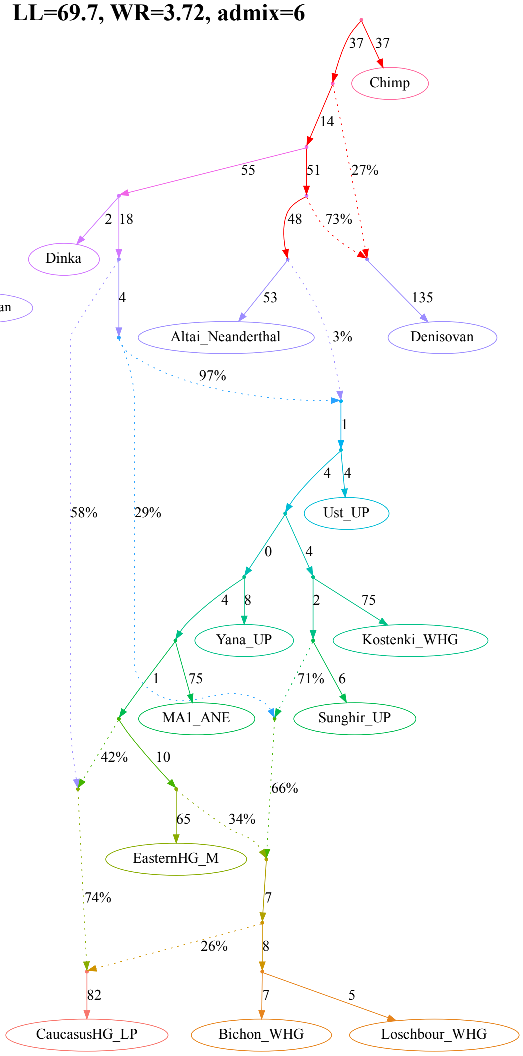

d, models fitting nominally better than the published one and not supporting the claim

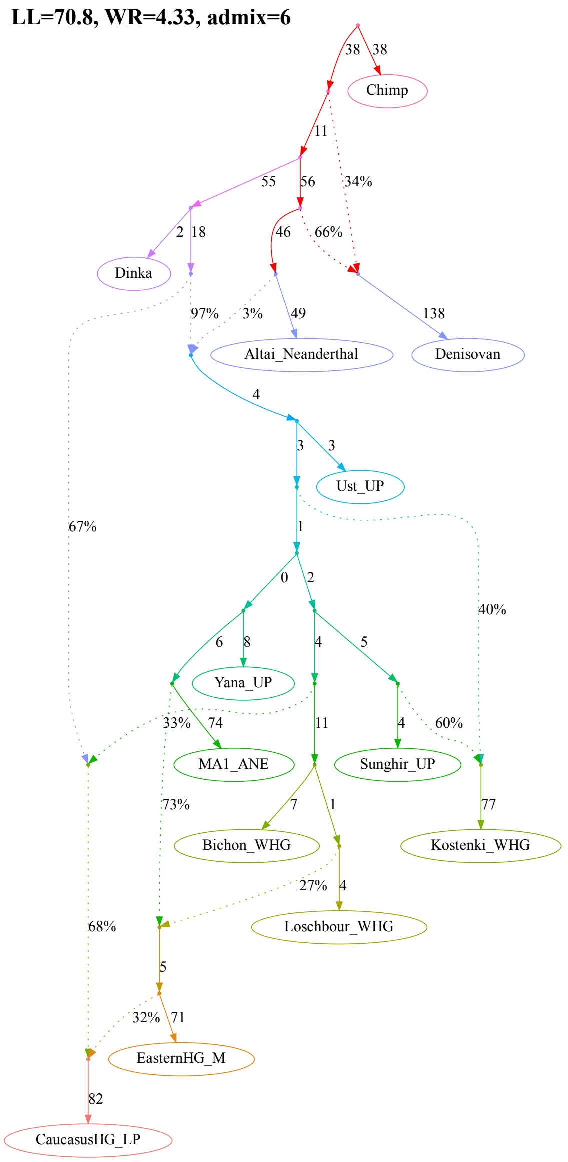

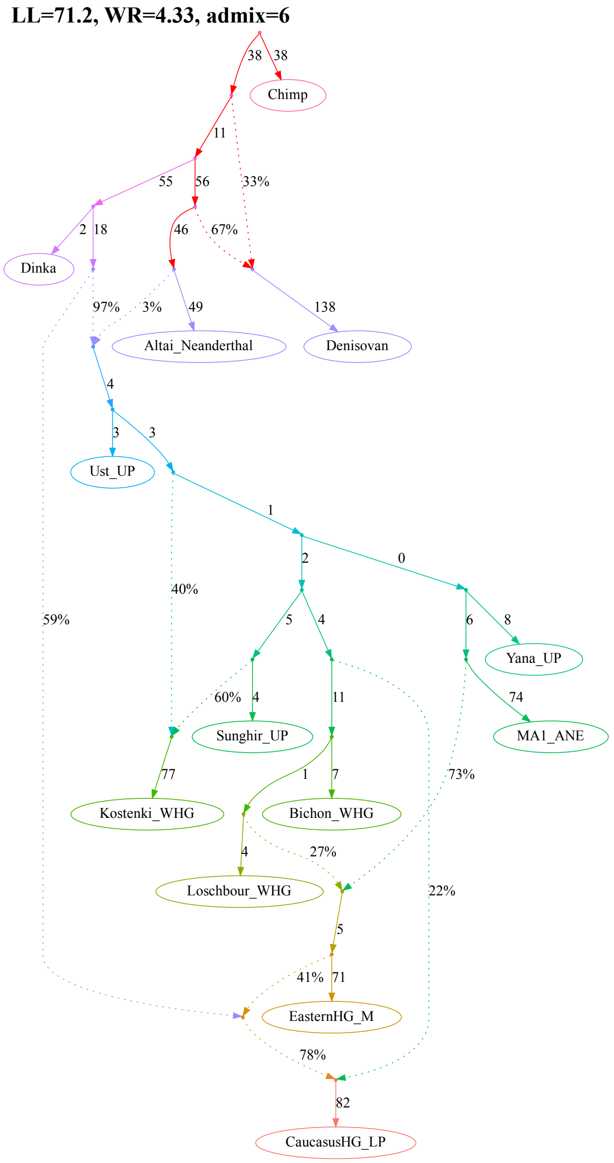

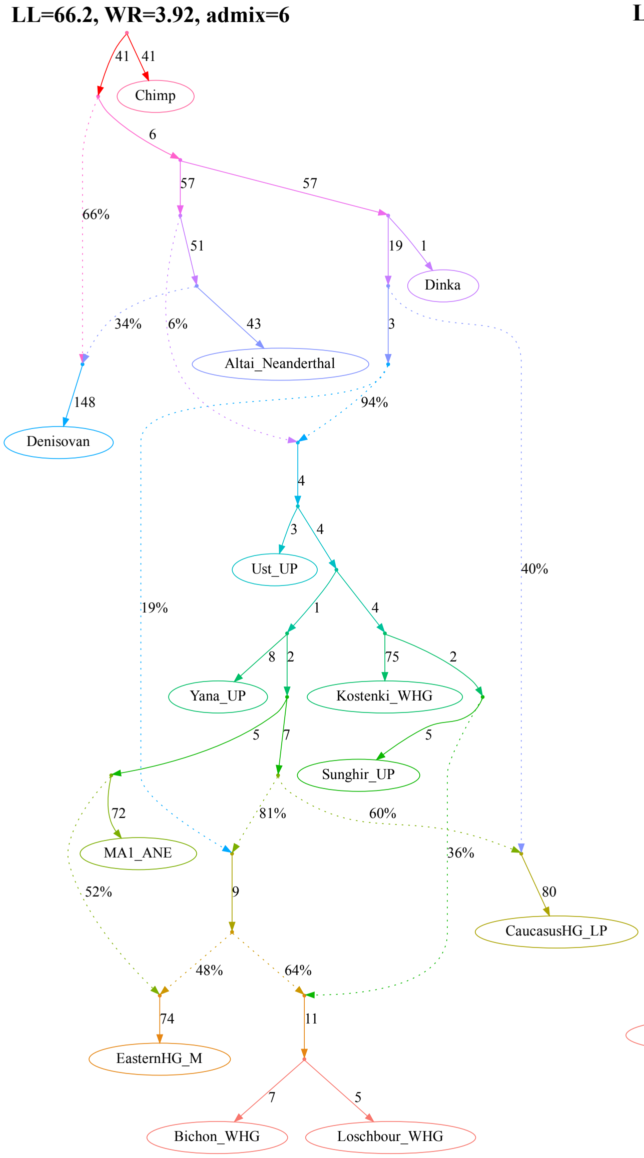
LL=66.2, WR=3.92, admix=6
Chimp
Dinka
Altai_Neanderthal
Denisovan
Ust_UP
Yana_UP
Kostenki_WHG
Sunghir_UP
MA1_ANE
CaucasusHG_LP
EasternHG_M
Bichon_WHG
Loschbour_WHG
66%
34%
6%
94%
19%
40%
81%
60%
36%
52%
48%
64%

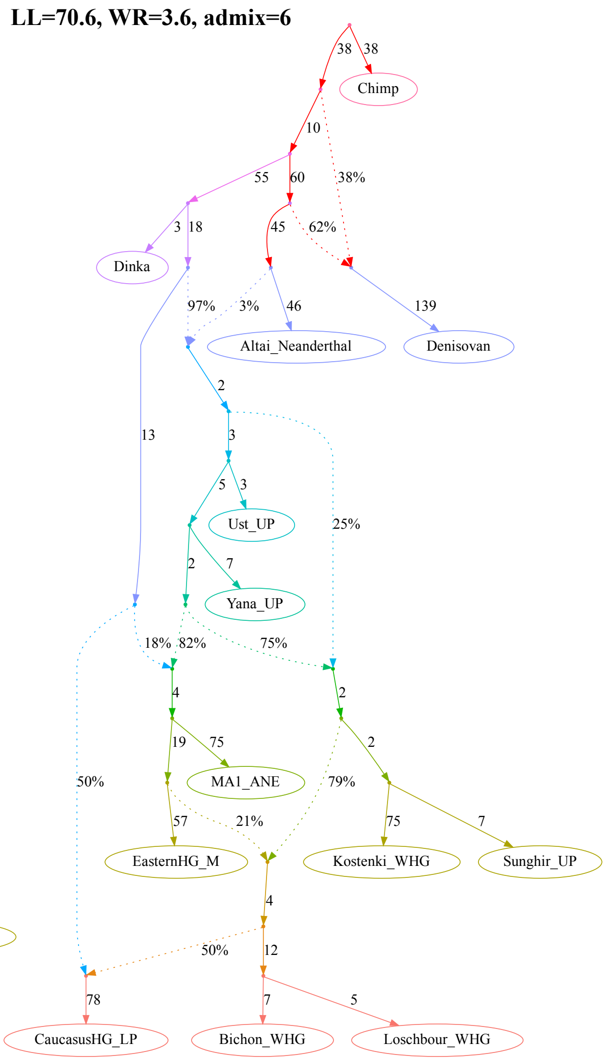
LL=70.6, WR=3.6, admix=6
Chimp
Dinka
Altai_Neanderthal
Denisovan
Ust_UP
Yana_UP
MA1_ANE
EasternHG_M
Kostenki_WHG
Sunghir_UP
CaucasusHG_LP
Bichon_WHG
Loschbour_WHG
38%
62%
97%
3%
25%
18%
82%
75%
50%
21%
79%
50%

e, models fitting nominally better than the published one and not supporting the claim

LL=67, WR=3.78, admix=6

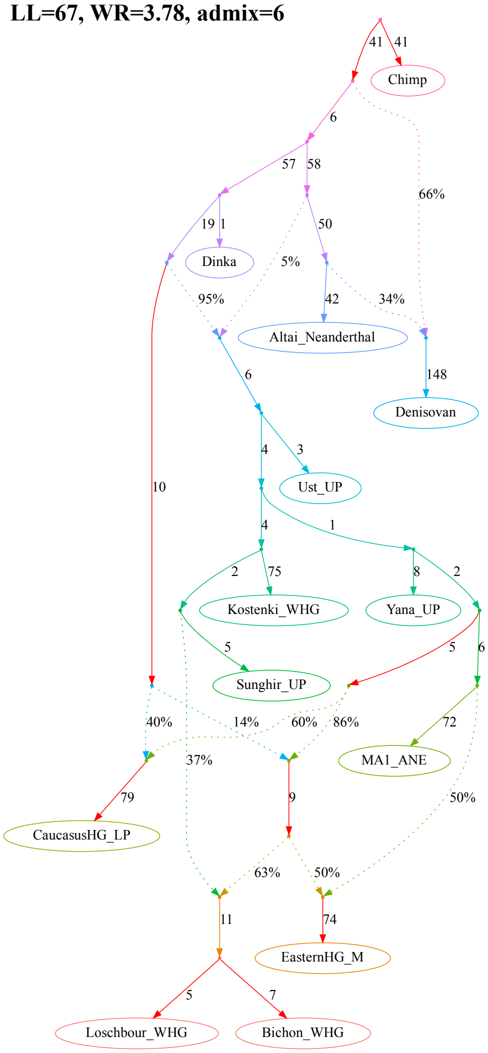

LL=67, WR=3.78, admix=6

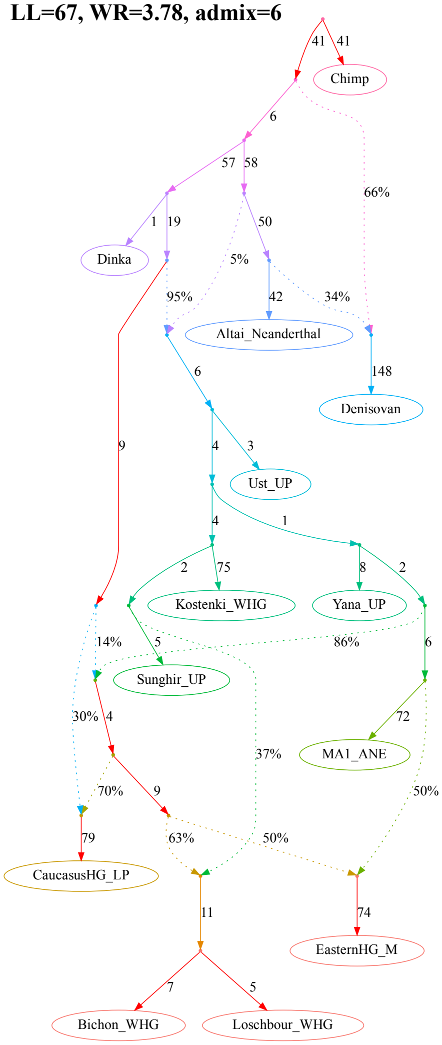

LL=71.2, WR=4.33, admix=6

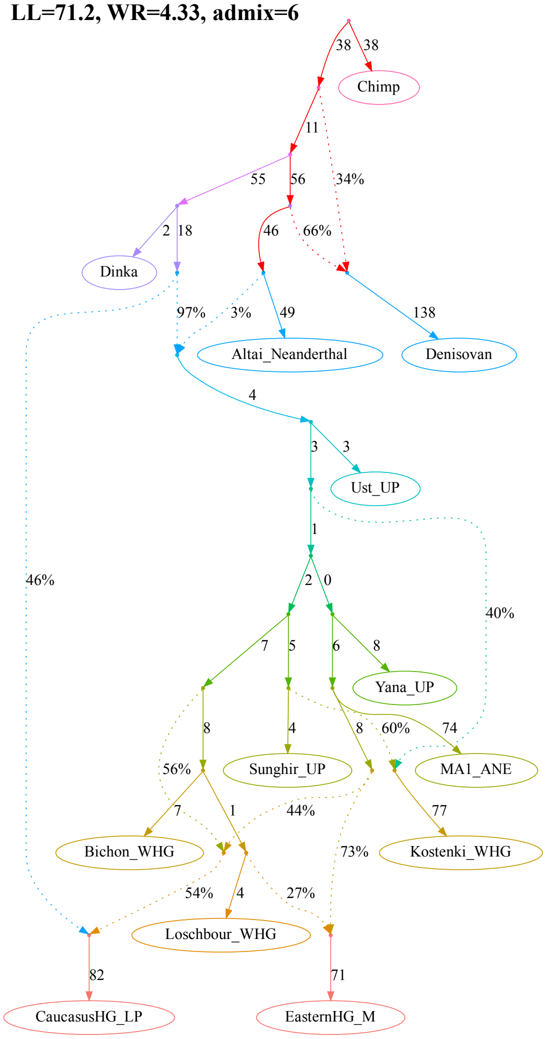

LL=70.7, WR=4.33, admix=6

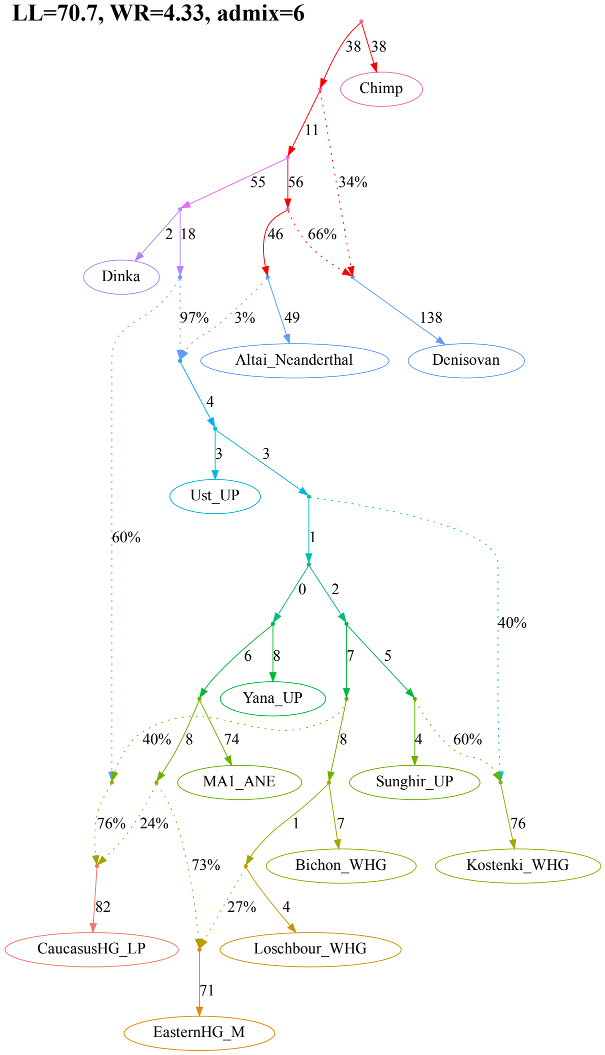

f, models fitting nominally better than the published one and not supporting the claim

LL=70.7, WR=4.33, admix=6

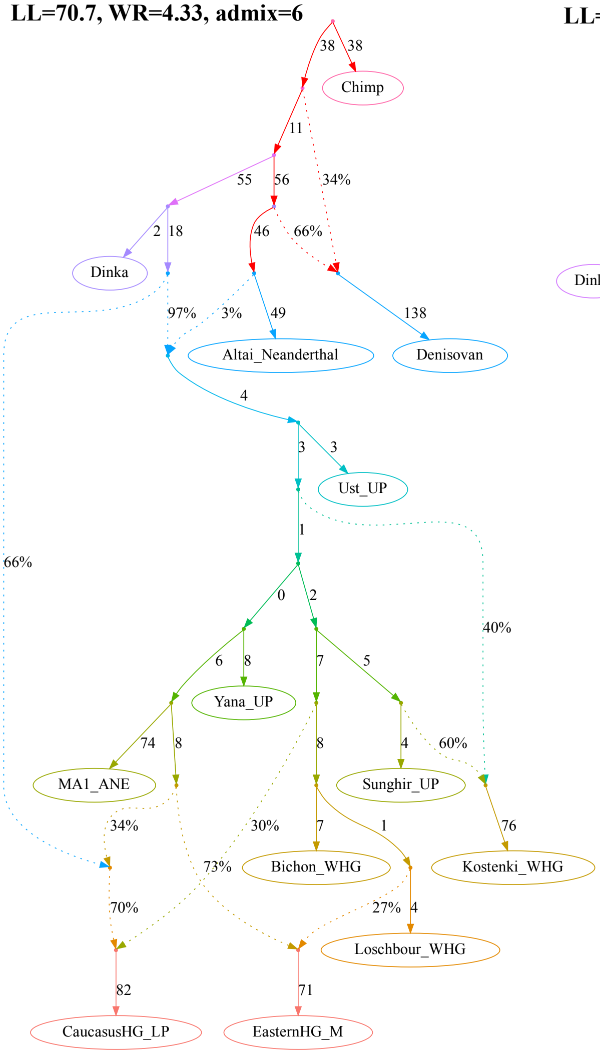

LL=71.1, WR=4.33, admix=6

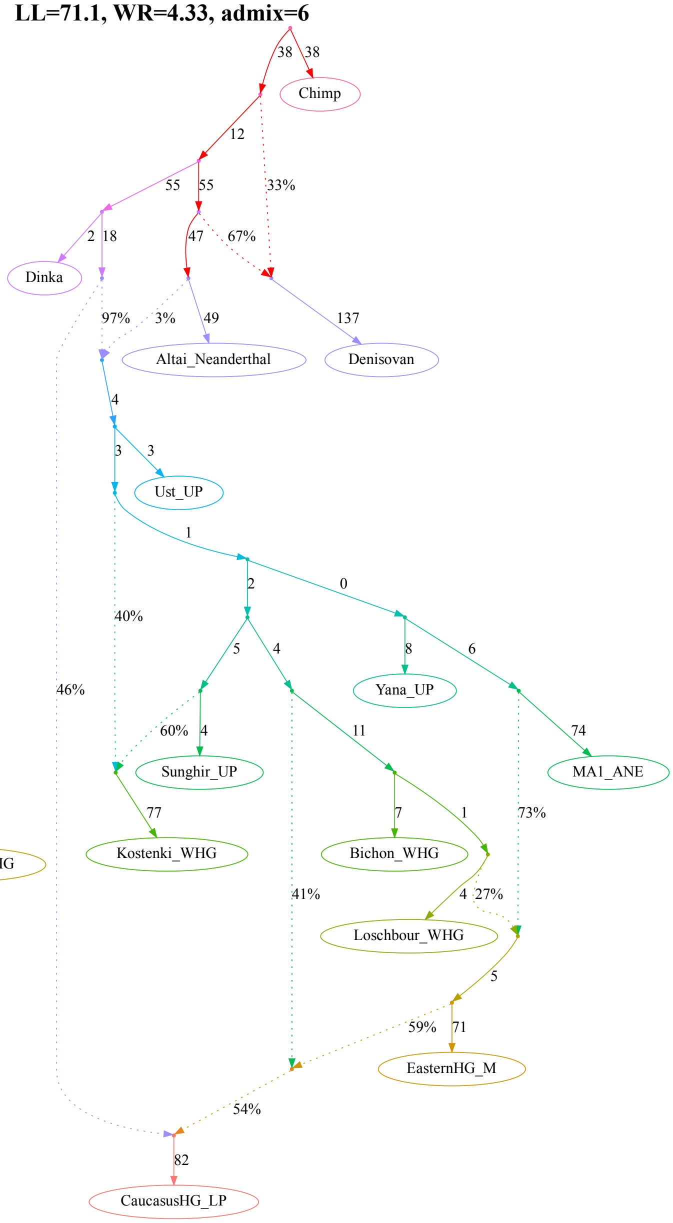

LL=71.4, WR=3.78, admix=6

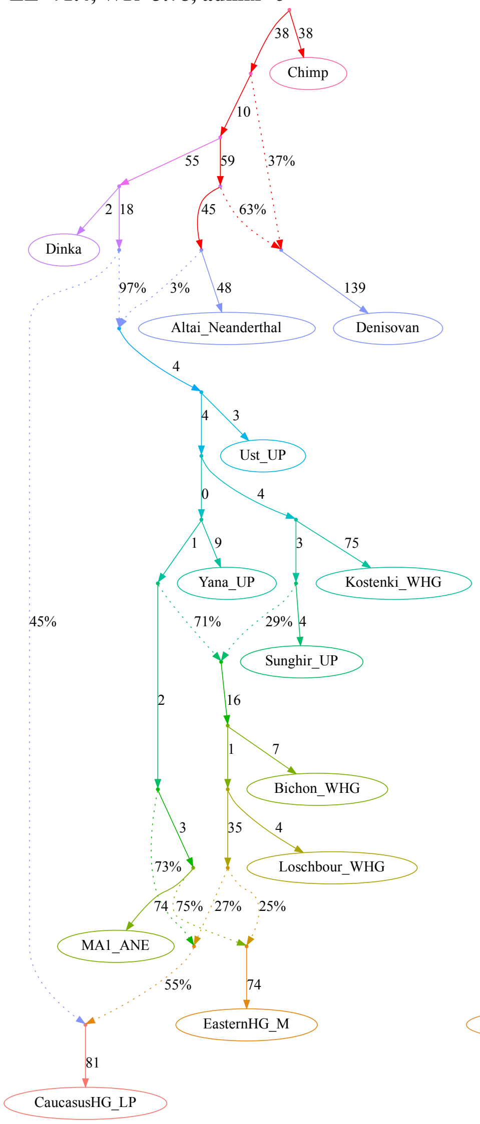

LL=72.2, WR=3.78, admix=6

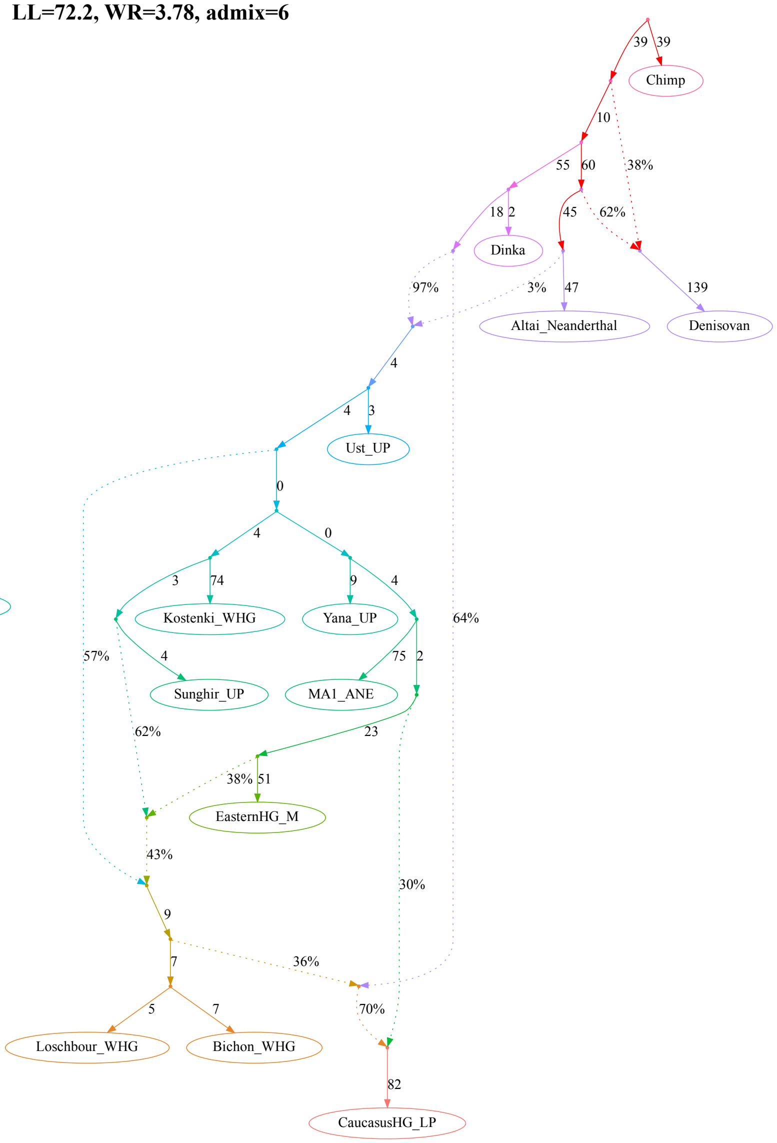

g, models fitting nominally better than the published one and not supporting the claim

LL=72.7, WR=3.78, admix=6

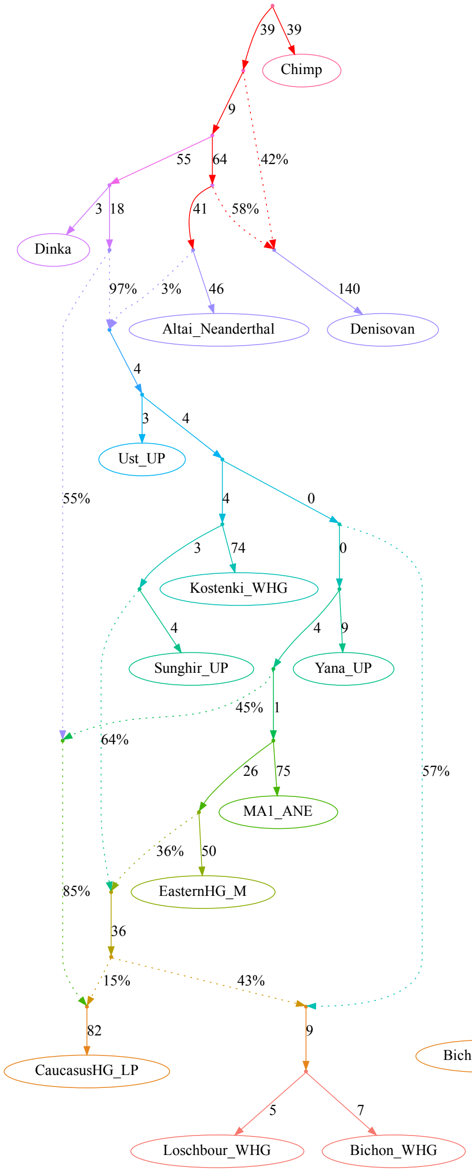

LL=73.2, WR=3.78, admix=6

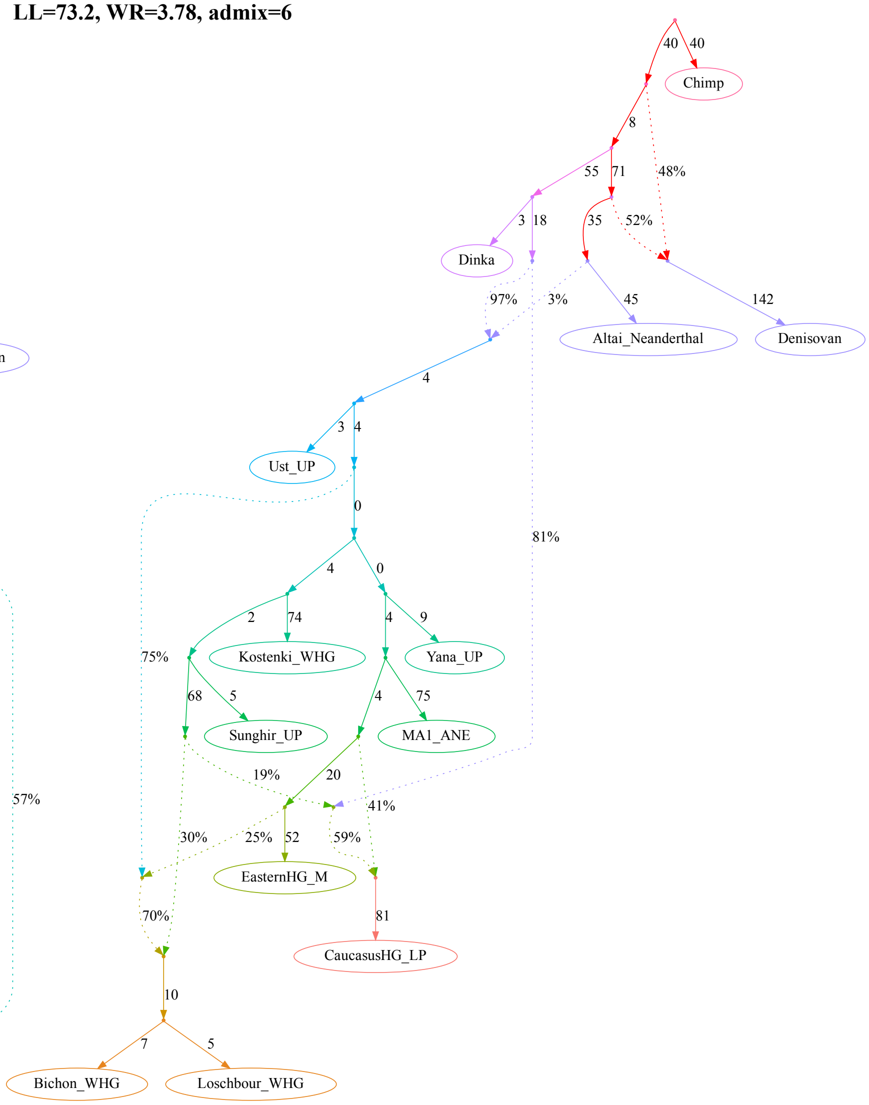

LL=73.4, WR=4.33, admix=6

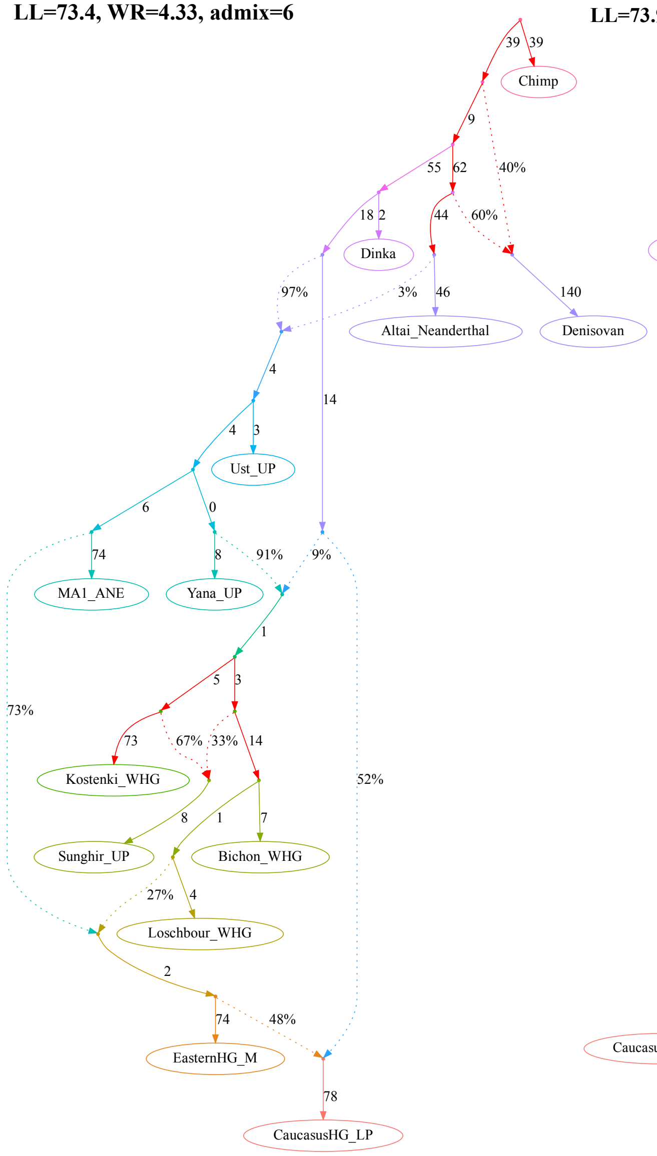

LL=73.9, WR=3.39, admix=6

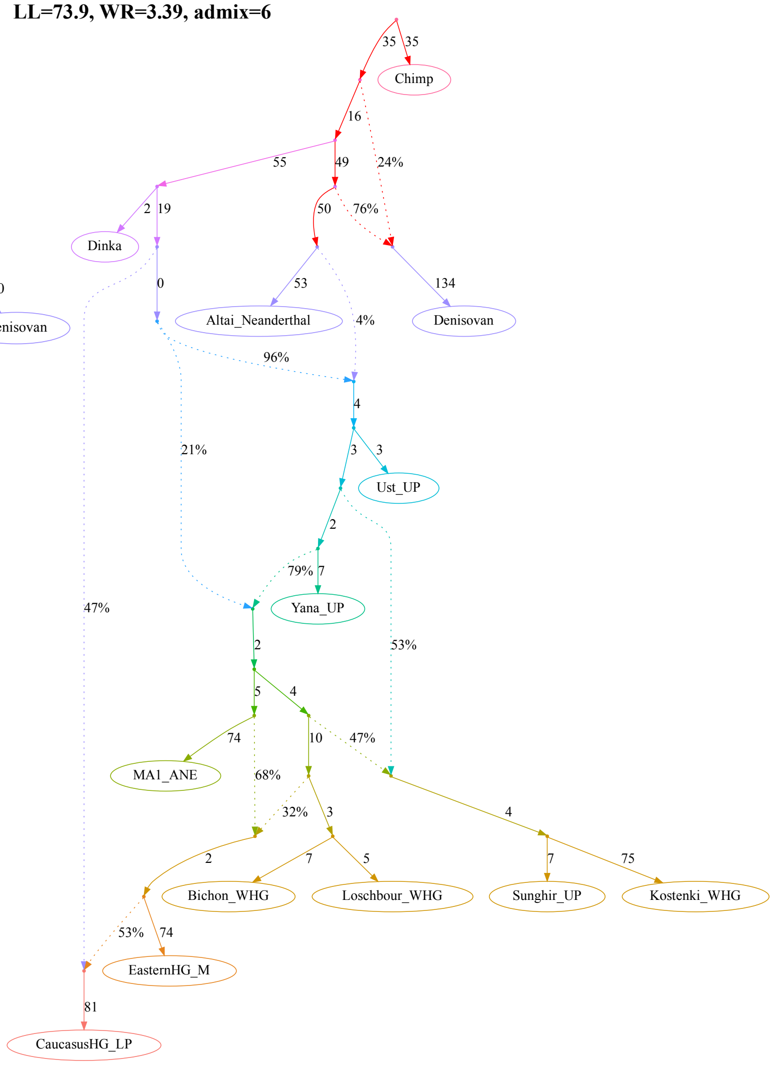

h, models fitting nominally better than the published one and not supporting the claim

LL=74.2, WR=4.33, admix=6

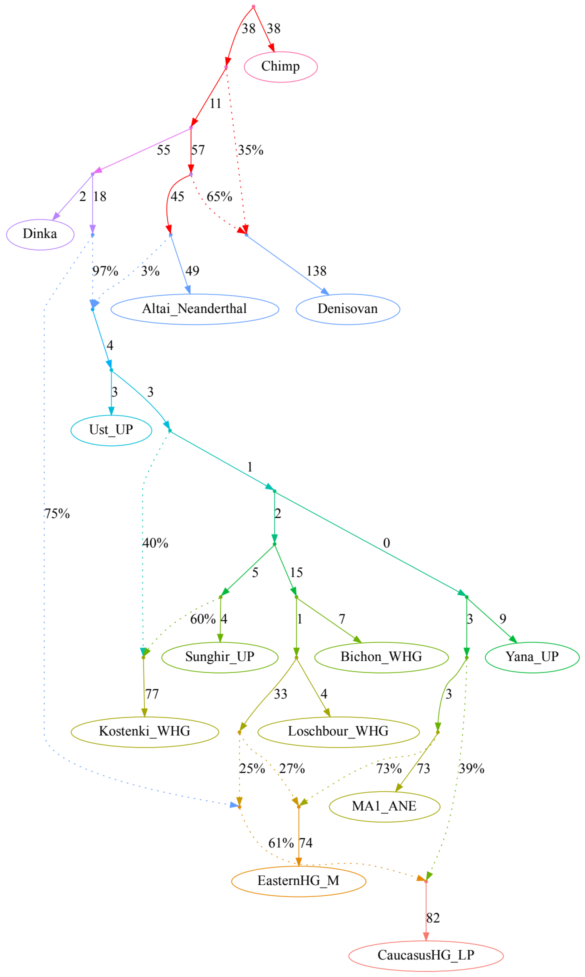

LL=74.2, WR=4.33, admix=6

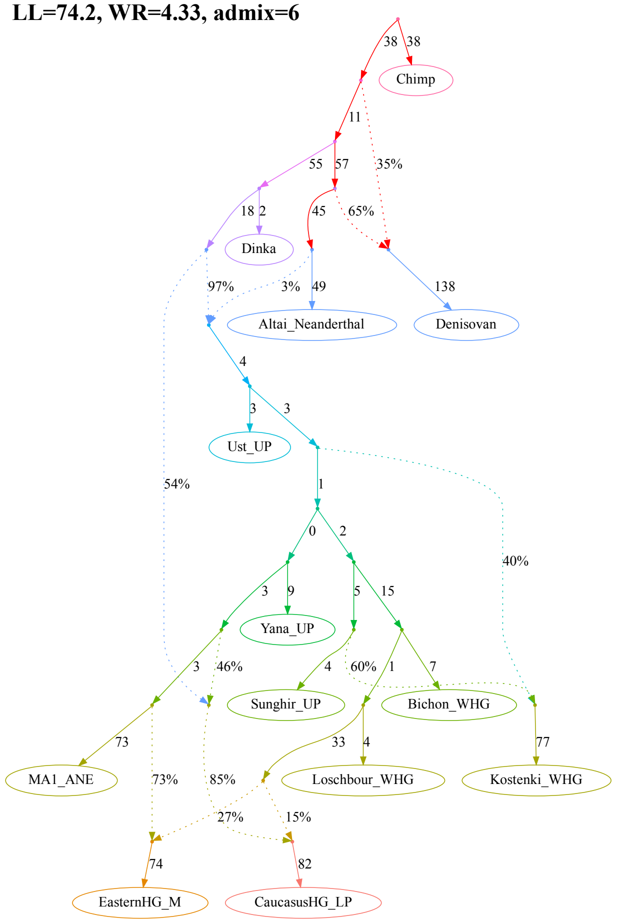

LL=75.1, WR=4.33, admix=6

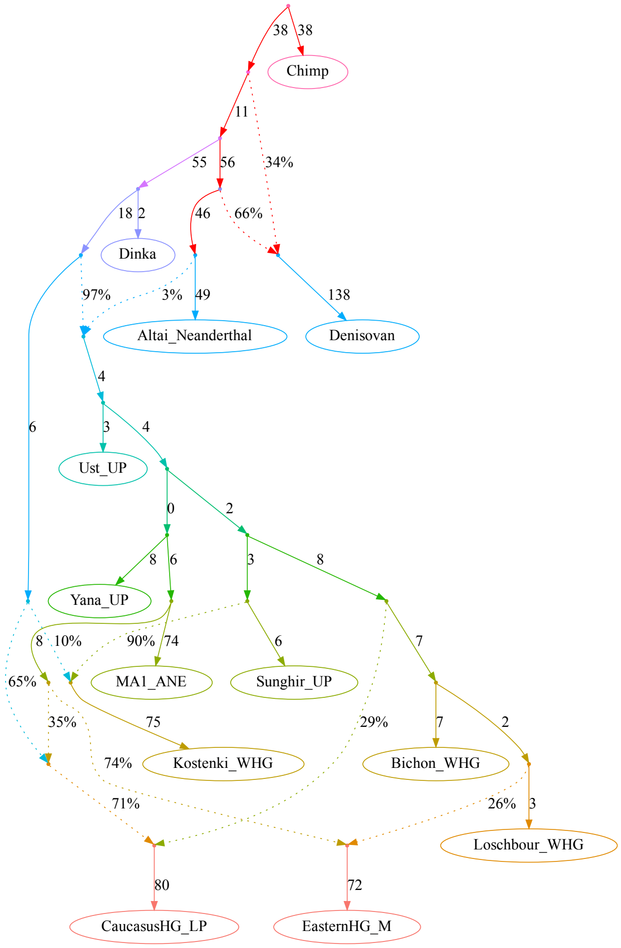

i, models fitting nominally better than the published one and <u>supporting</u> the claim

LL=75.1, WR=3.78, admix=6

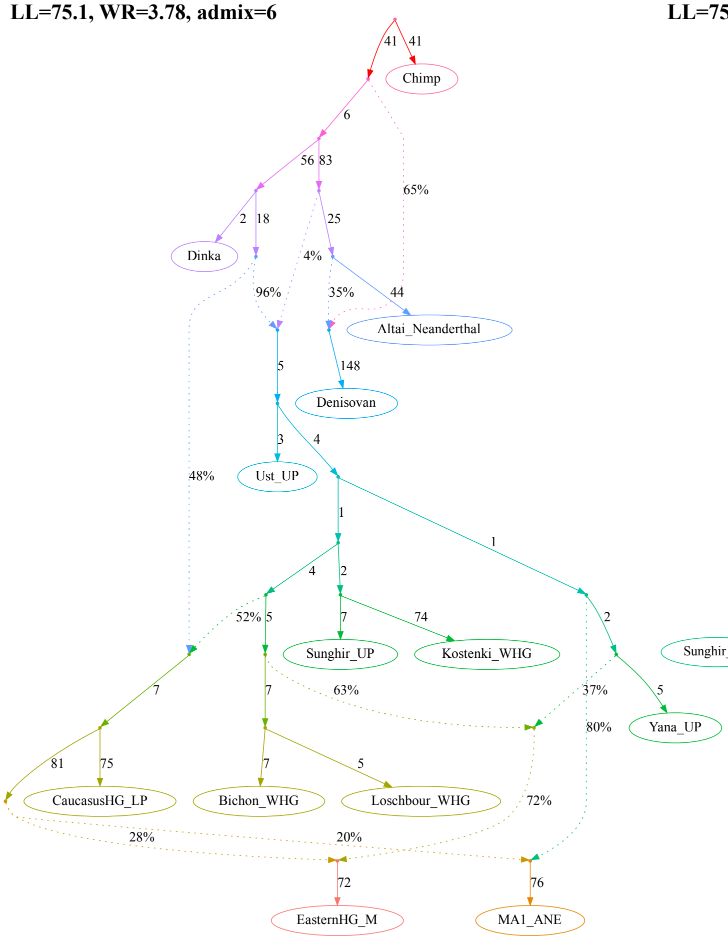

LL=75.9, WR=3.78, admix=6

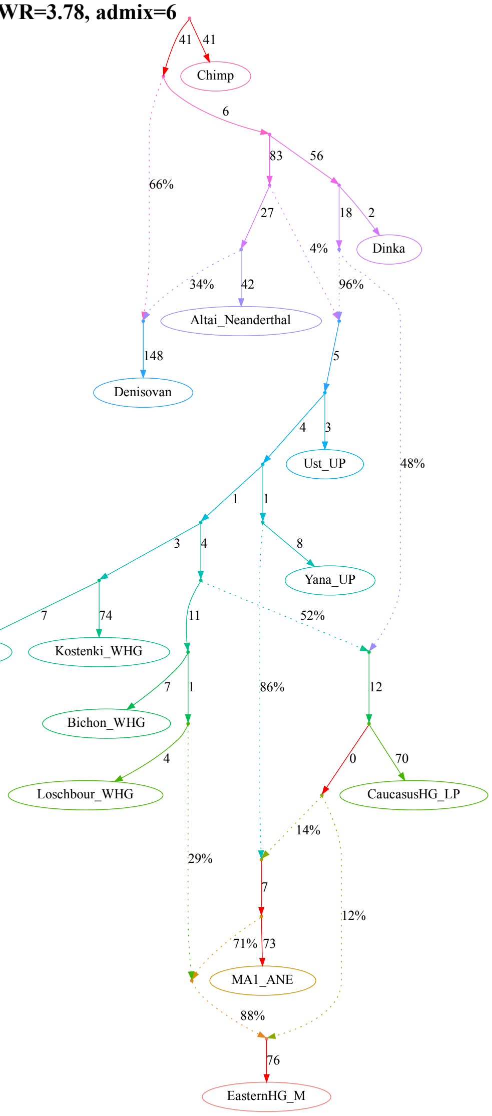

Supplement: Figure 4—source data 3. — Model parameters (graph edges) that were inferred to be unidentifiable are plotted in red. (a) The published model, with chimpanzee added as an outgroup and four low-level gene flows from the Neanderthal lineage dropped. The following claim in Sikora et al., 2019 relies on the admixture graph: The Mal'ta (MA1_ANE) lineage receives a gene flow from the Caucasus hunter-gatherer (CaucasusHG_LP) lineage. (b-h) Models fitting nominally better than the published one and not supporting the claim, (i) models fitting nominally better than the published one and supporting the claim. [file elife-85492-fig4-data3.pdf]
