# Supplementary material for: On the limits of fitting complex models of population history to f-statistics
Source: eLife. 2023 Jun 29;12:e85492. doi: 10.7554/eLife.85492 (PMC10310323; doi:10.7554/eLife.85492)

Figure 4–source data 4. Simplified published admixture graph for East Eurasian groups from Sikora *et al*. (2019) and alternative graphs found with *findGraphs* (14 populations, 6 admixture events). Model parameters (graph edges) that were inferred to be unidentifiable are plotted in red.

a, published model, with chimpanzee added as an outgroup and 4 low-level gene flows from the Neanderthal lineage dropped. **b**, the same model as above, simplified by dropping unidentifiable edges

a **b**

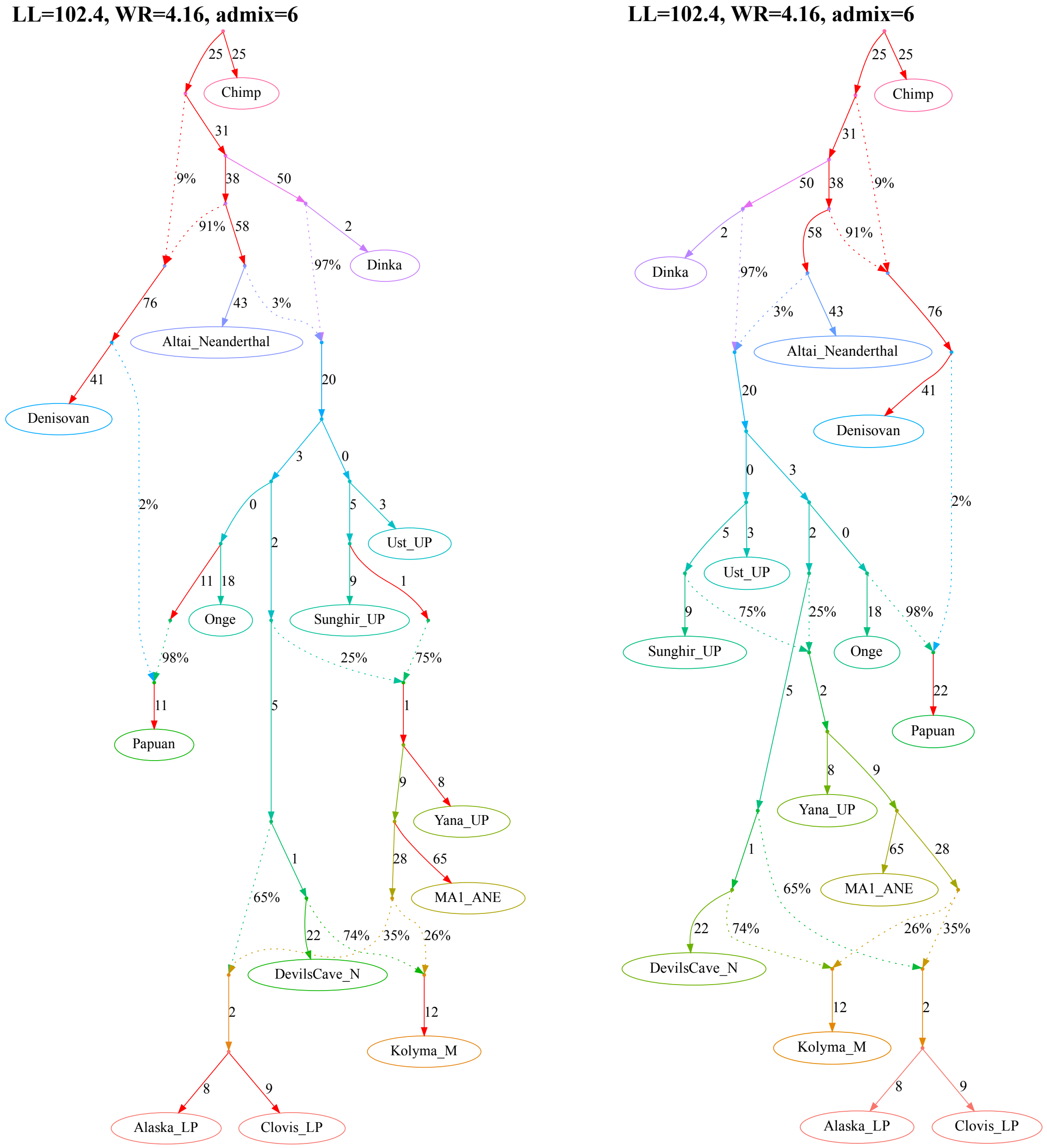

Claims by Sikora *et al*. 2019 relying on the admixture graph above:

1) The Mal’ta (MA1_ANE) and Yana (Yana_UP) lineages receive a gene flow from an Asian source diverging before the Devil’s Cave (DevilsCave_N), Kolyma (Kolyma_M), USR1 (Alaska_LP), and Clovis (Clovis_LP) lineages;
2) European ancestry in the Kolyma, USR1, and Clovis lineages is closer to Mal’ta than to Yana;
3) The Devil’s Cave lineage receives no European-related gene flows, and Kolyma has less European-related ancestry than ancient Americans (USR1 and Clovis).

c, an alternative model fitting nominally better than the published one and supporting all 3 claims

LL=95.1, WR=4.44, admix=6

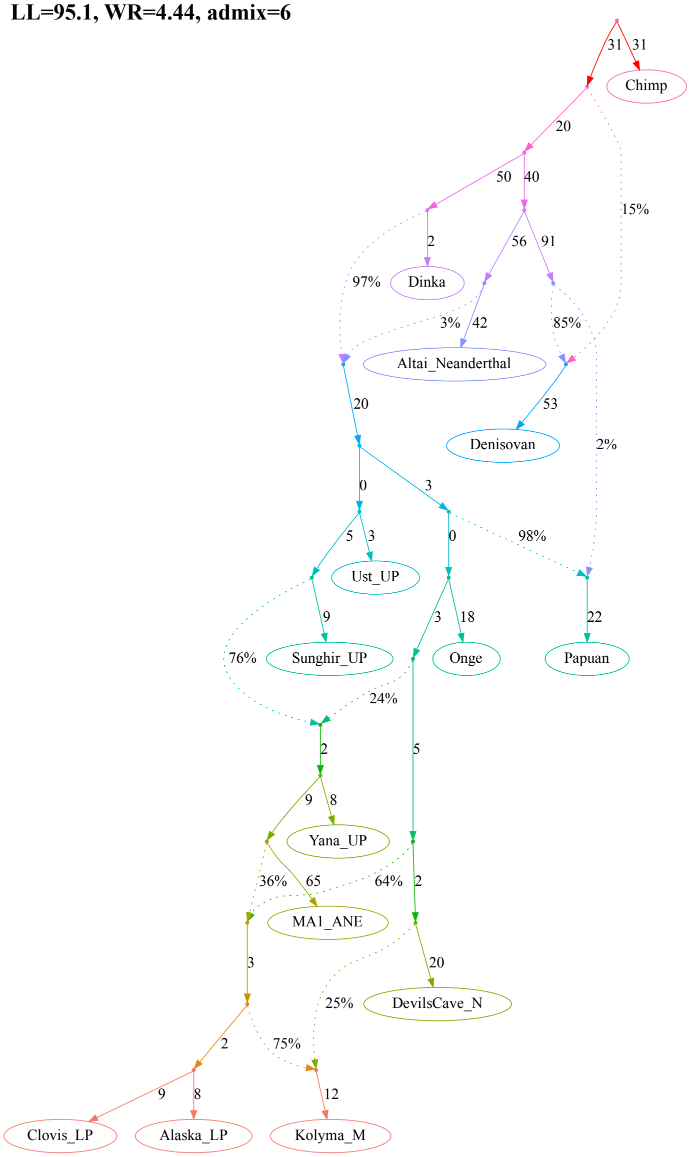

d, alternative models fitting not significantly worse than the published model

claims 1&3 not supported, claim 2 supported

LL=104.3, WR=4.05, admix=6

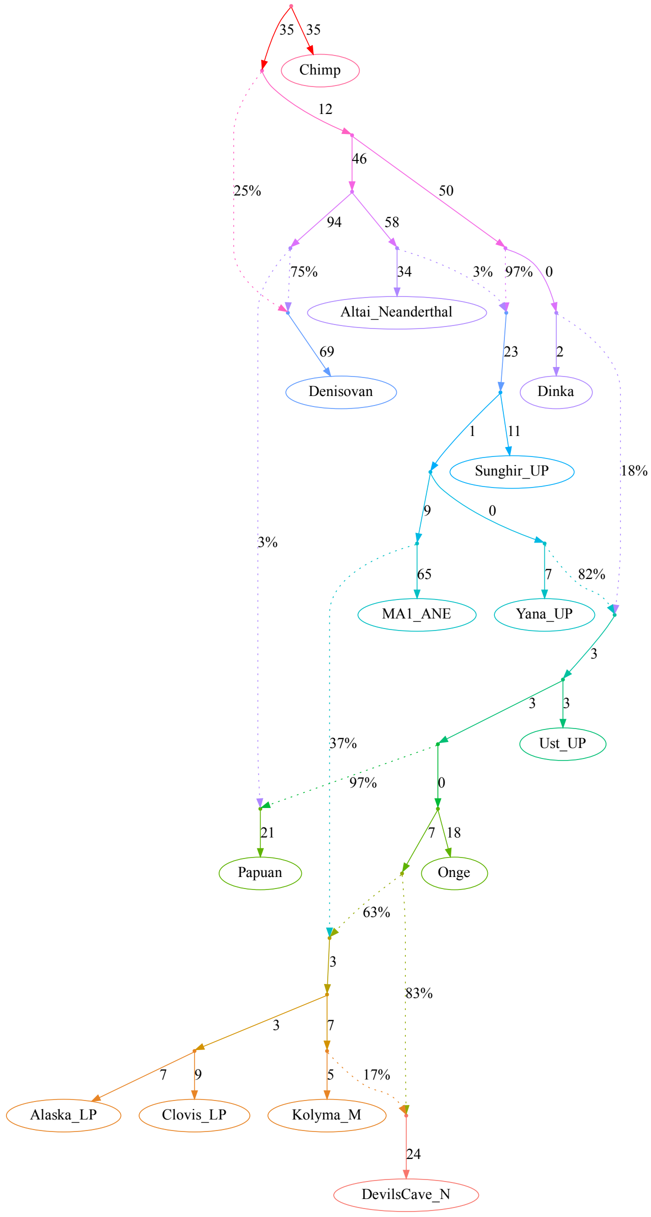

claims 1 not supported, claims 2&3 supported

LL=105.8, WR=4.78, admix=6

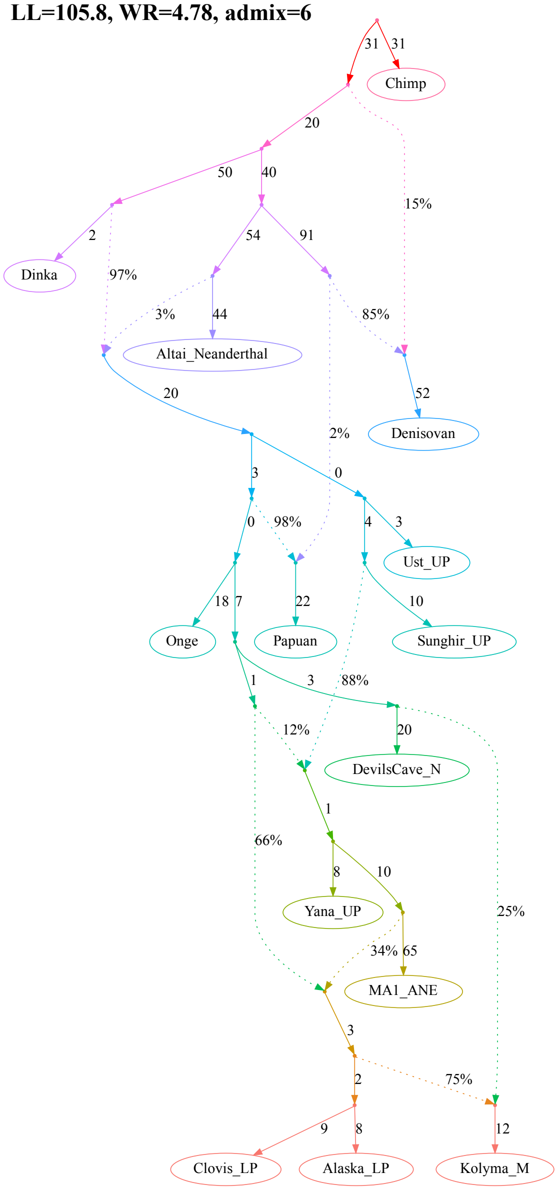

claim 1 not supported, claims 2&3 supported

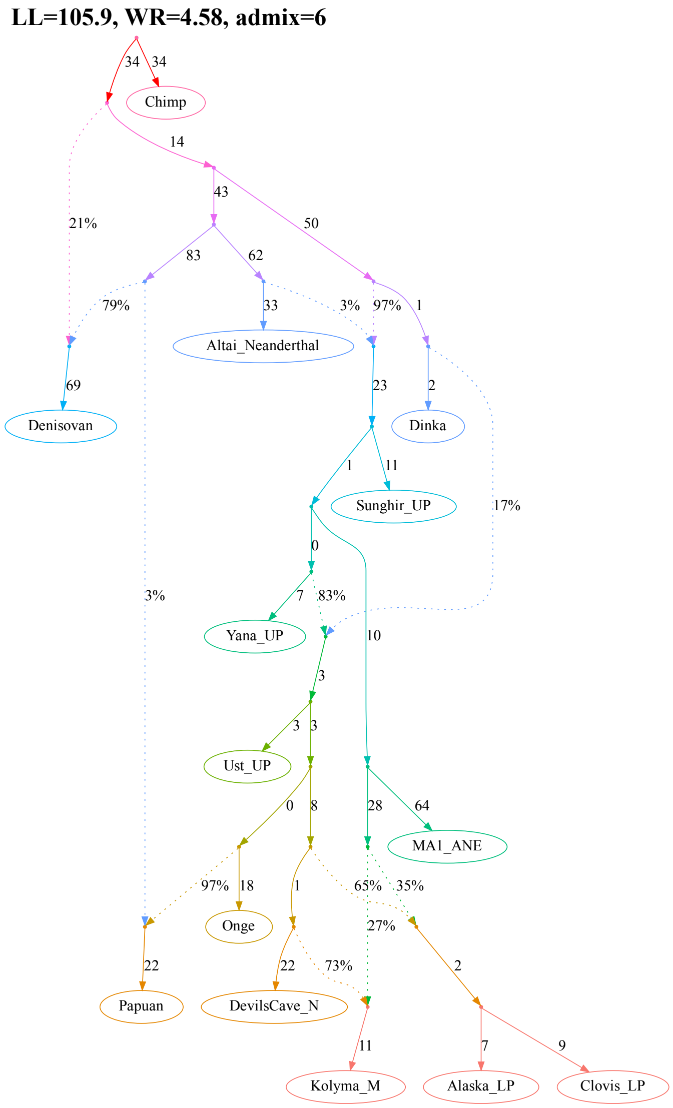

claims 1&2 supported, claim 3 not supported

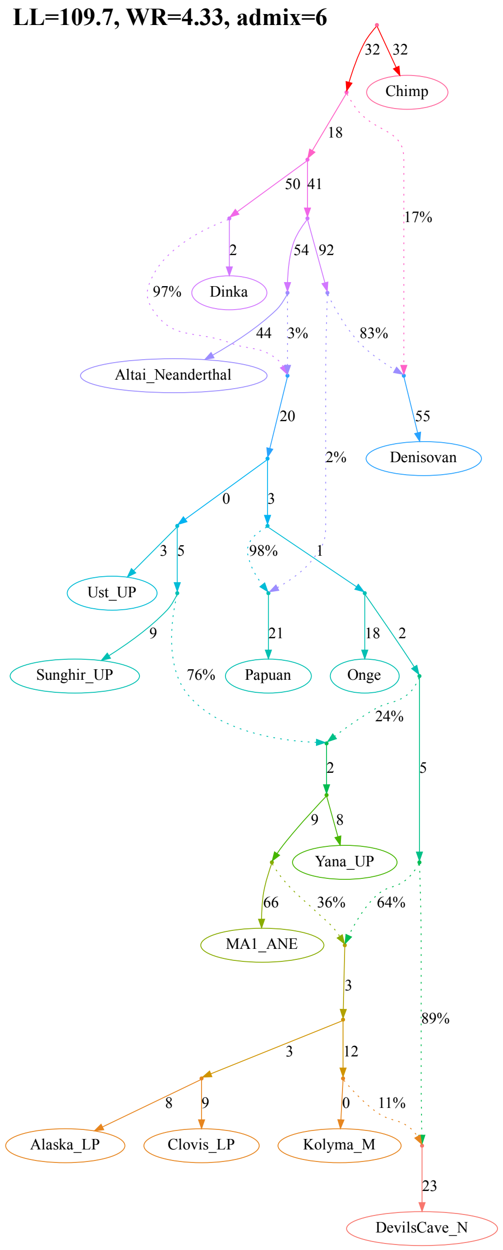

e, alternative models fitting not significantly worse than the published model

claim 1 not supported, claims 2&3 supported

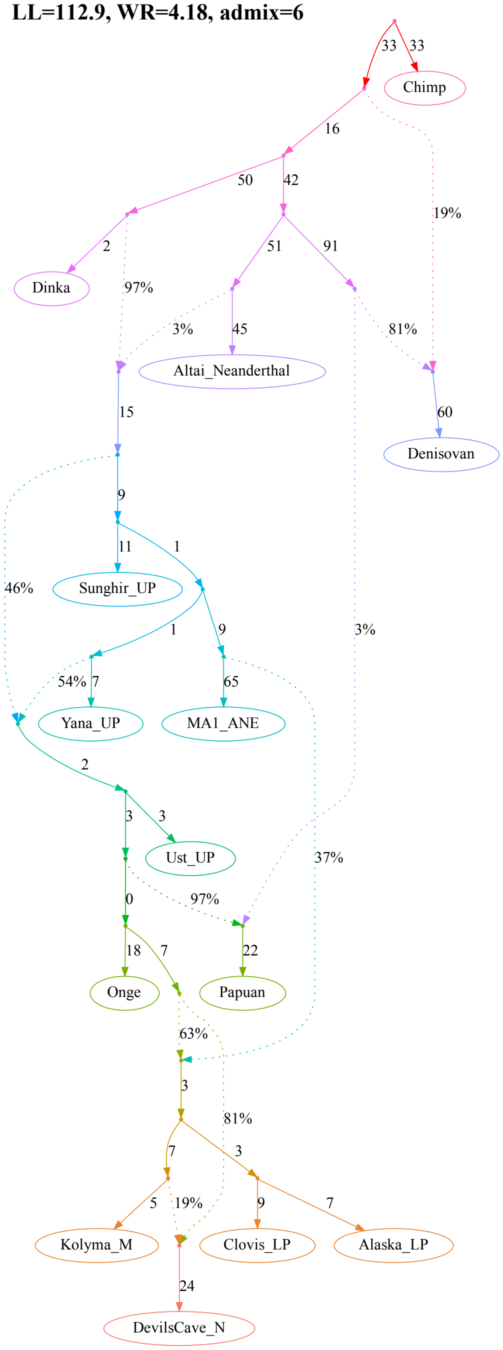

claims 1&3 not supported, claim 2 supported

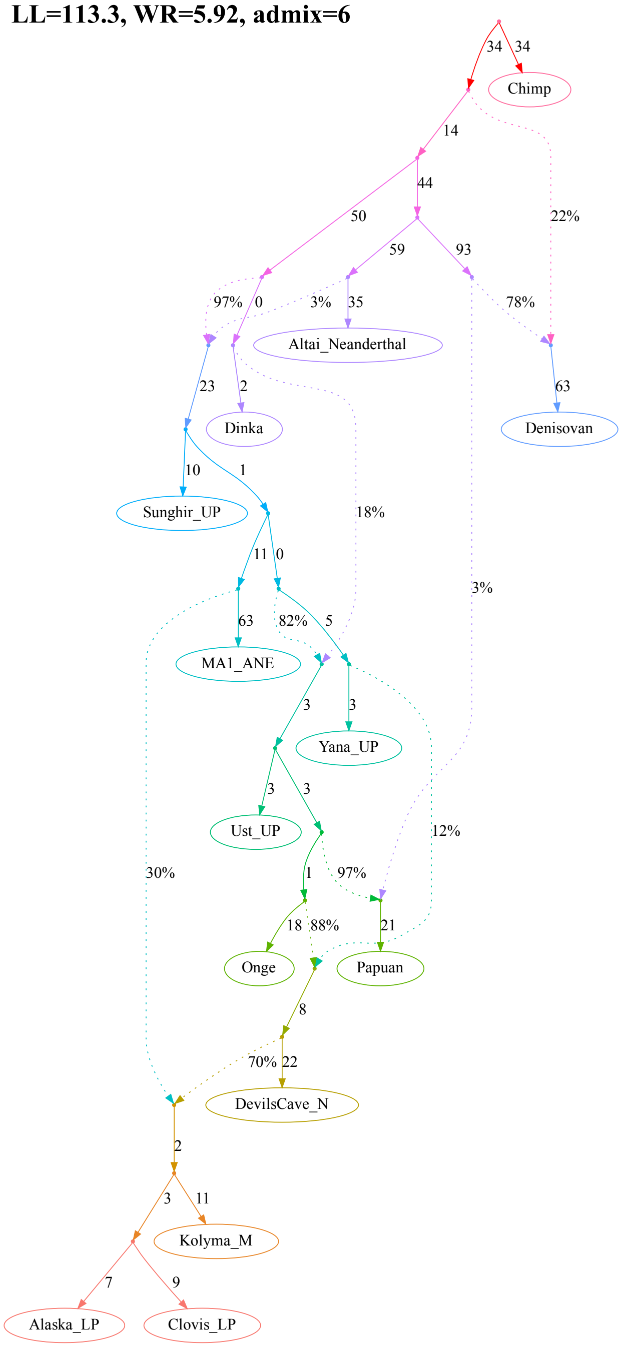

claim 1 not supported, claims 2&3 supported

LL=119.6, WR=5.01, admix=6

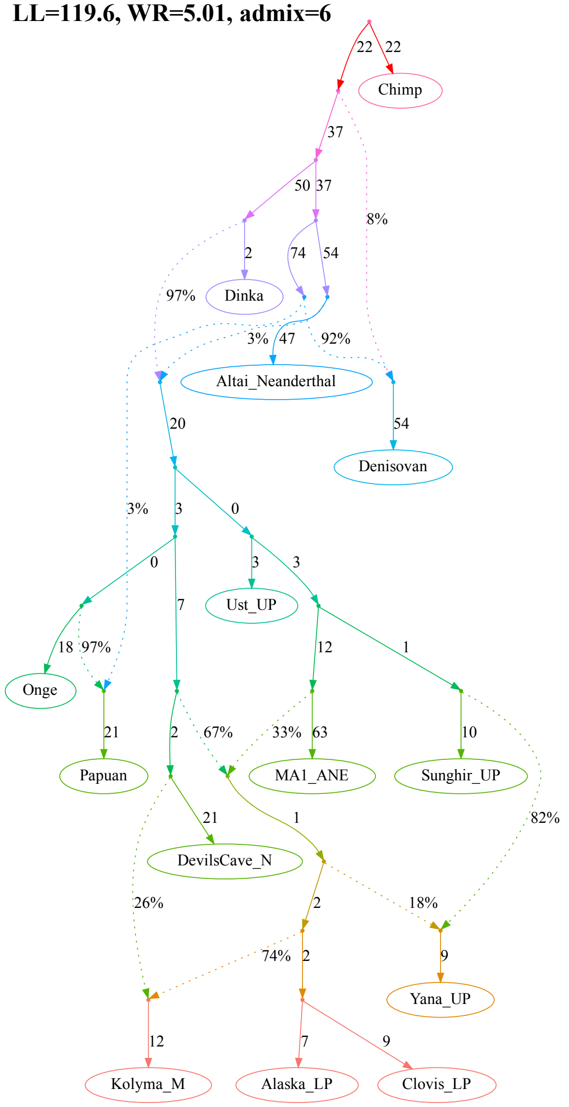

claim 1 not supported, claims 2&3 supported

LL=120.9, WR=5.38, admix=6

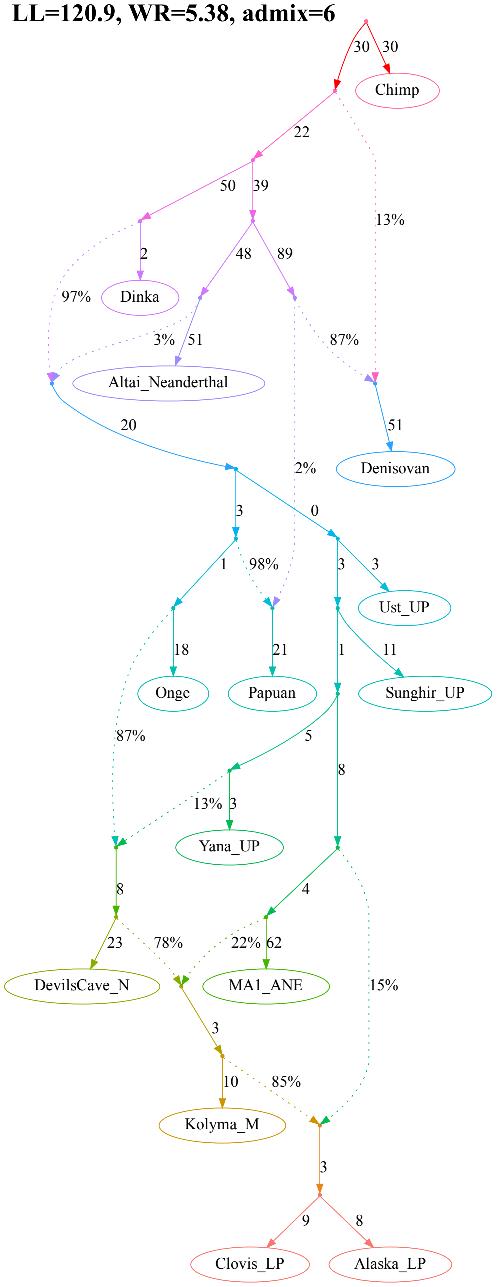

f, alternative models fitting not significantly worse than the published model

claims 1&3 not supported, claim 2 supported

claims 1&3 not supported, claim 2 supported

LL=116.6, WR=4.69, admix=6

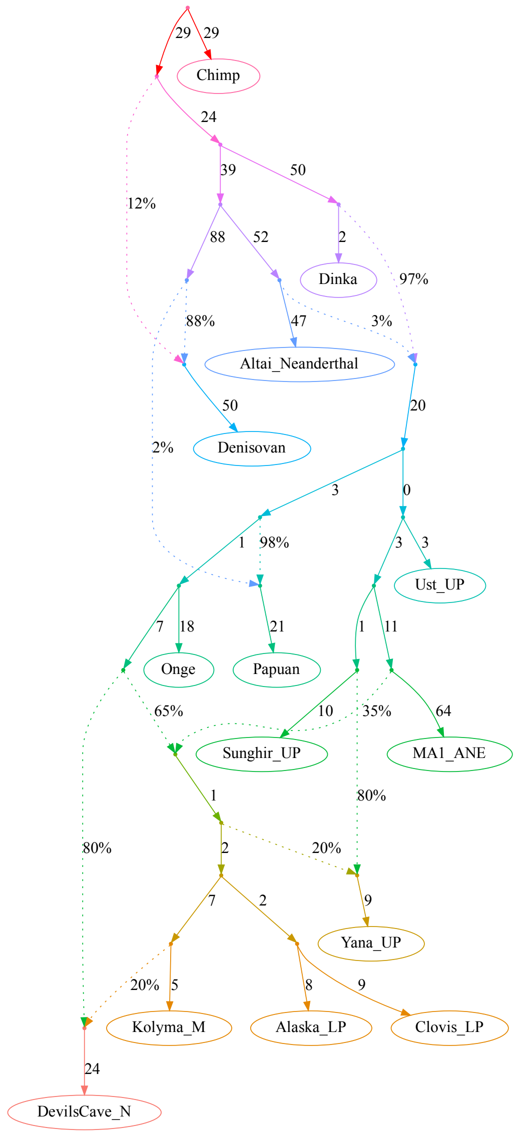

LL=119.1, WR=4.42, admix=6

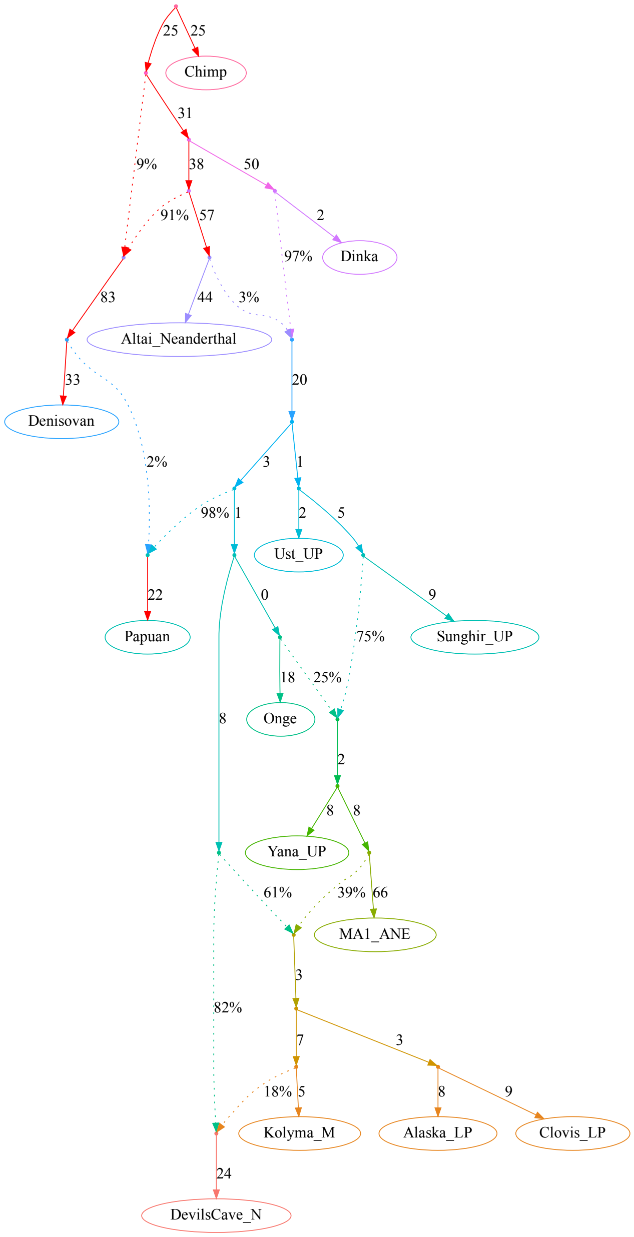

claims 1&2 supported, claim 3 not supported

LL=119.3, WR=4.31, admix=6

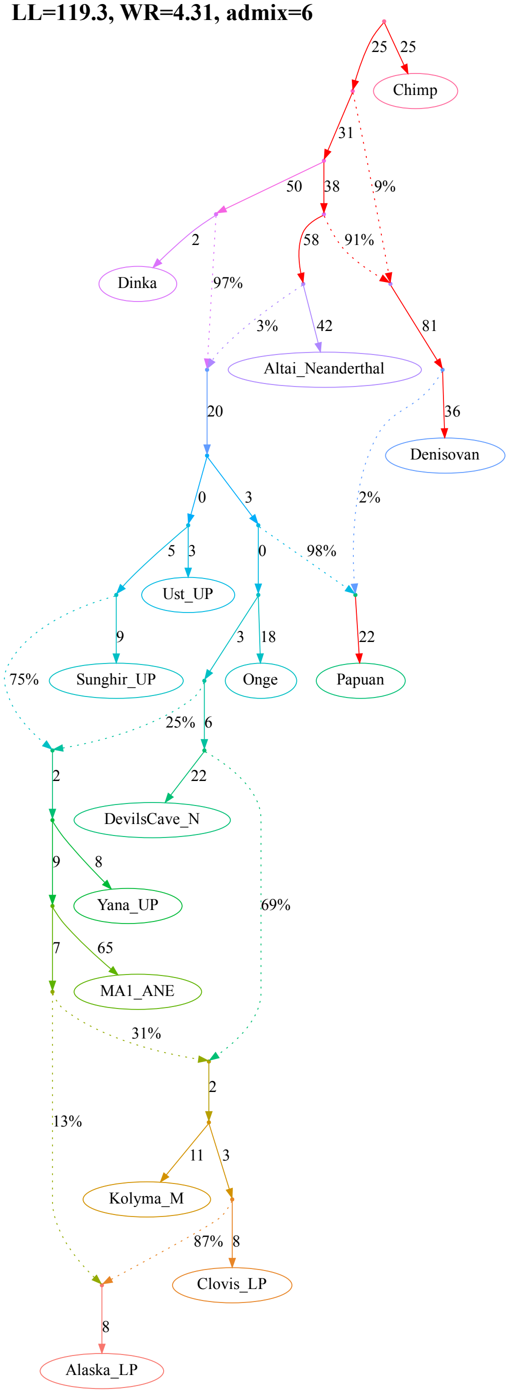

claims 1&2 supported, claim 3 not supported

LL=119.9, WR=4.24, admix=6

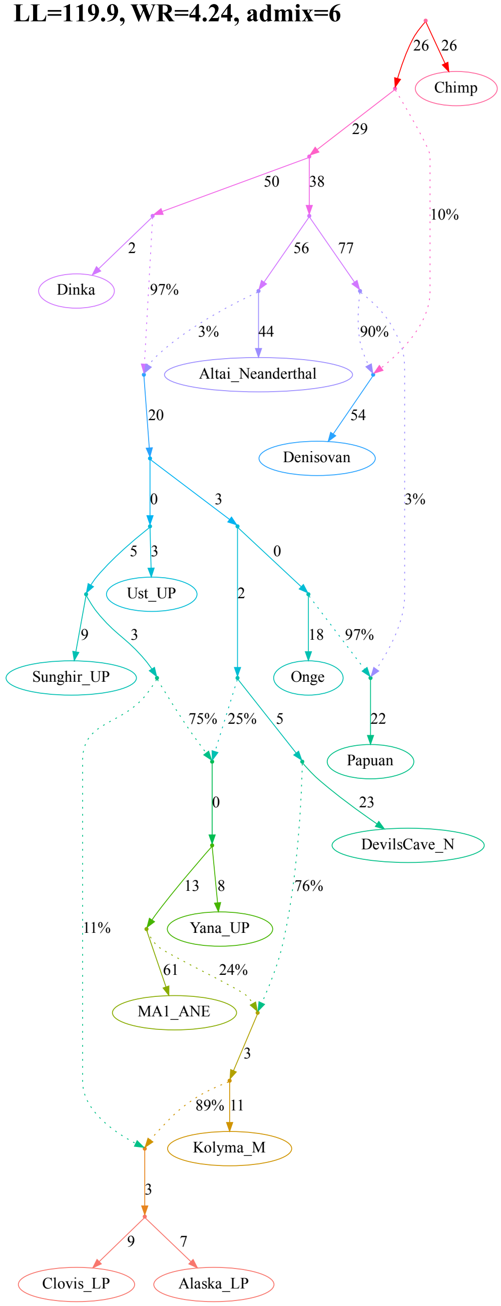

g, alternative models fitting not significantly worse than the published model

claims 1&3 not supported, claim 2 supported

LL=120, WR=4.94, admix=6

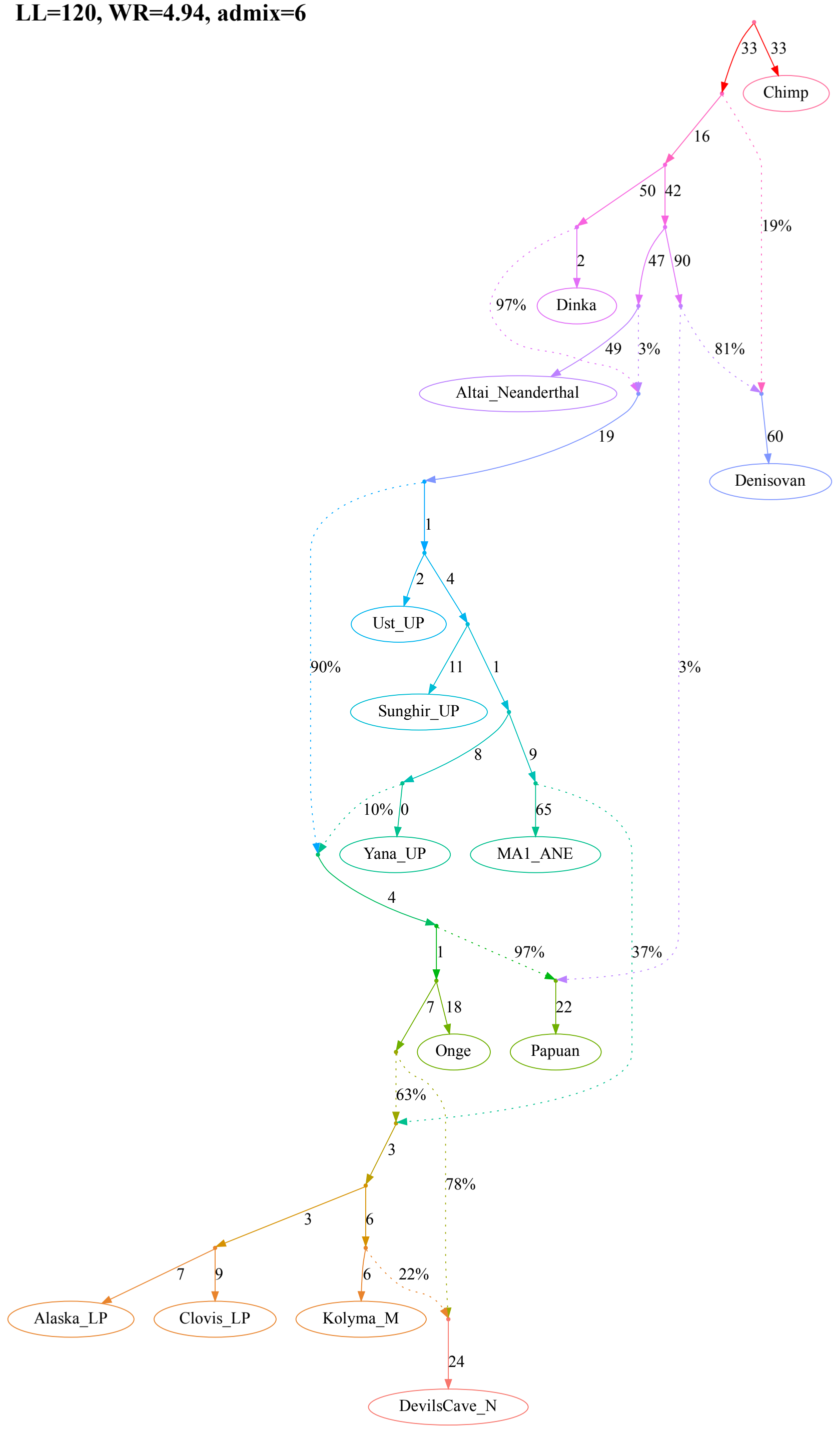

Supplement: Figure 4—source data 4. — Model parameters (graph edges) that were inferred to be unidentifiable are plotted in red. (a) The published model, with chimpanzee added as an outgroup and four low-level gene flows from the Neanderthal lineage dropped. (b) The published model simplified by dropping unidentifiable edges. The following claims in Sikora et al., 2019 rely on the admixture graph: (1) The Mal’ta (MA1_ANE) and Yana (Yana_UP) lineages receive a gene flow from an Asian source diverging before the Devil’s Cave (DevilsCave_N), Kolyma (Kolyma_M), USR1 (Alaska_LP), and Clovis (Clovis_LP) lineages; (2) European ancestry in the Kolyma, USR1, and Clovis lineages is closer to Mal’ta than to Yana; (3) The Devil’s Cave lineage receives no European-related gene flows, and Kolyma has less European-related ancestry than ancient Americans (USR1 and Clovis). (c) An alternative model fitting nominally better than the published one and supporting all three claims, (d-g) alternative models fitting not significantly worse than the published model. [file elife-85492-fig4-data4.pdf]
